# Integrated Single-Cell and RNA Sequencing Analysis Identifies Key Immune Cell and Dendritic Cells Associated Genes Participated in Myocarditis

**DOI:** 10.1155/2022/8655343

**Published:** 2022-10-03

**Authors:** Qiang Gong, Jianfeng Huang, Qicai Wu

**Affiliations:** Department of Cardiac Surgery, Jiangxi Academy of Clinical Medical Sciences, The First Affiliated Hospital of Nanchang University, Nanchang, 330006 Jiangxi, China

## Abstract

Myocarditis is a complex disease characterized by myocardial inflammatory cell infiltration. The purpose of our study was to investigate the gene and single-cell signature to explore the involvement of immune cells in myocarditis. Gene expressions merged from GSE35182 and GSE35182 datasets were subjected to differential expression gene (DEG) analysis and PPI network construction. The correlation analysis of DEGs with immune cell infiltration was performed. Single-cell RNA sequencing (scRNA-seq) was downloaded from GSE174458. A total of 58 DEGs were identified, including 51 DEGs upregulated and 7 DEGs downregulated in the myocarditis group compared with the control group. GO and KEGG enrichment analyses revealed that myocarditis triggered DEGs mainly involved in immune-related processes and pathways. PPI network analysis identified 20 central hub genes. Occurrence of myocarditis induced significant enrichment of conventional dendritic cell 2 (cDC2), plasmacytoid DC, and plasma cell in myocardial tissue. Mmp12, Gpnmb, and Atp6v0d2 expressions were positively correlated with cDC abundance, of which only Mmp1 and Gpnmb were shared with hub gene list. A total of 20972 cells in scRNA-seq yielded 26 cell clusters and annotated 9 cell types, including fibroblasts, neutrophils, stromal cells, monocytes, basophils, B cells, natural killer T cells, innate lymphoid cells, and T cells, and only proportion of natural killer T cells and monocytes were higher in the myocarditis than in control. Monocytes annotated 3 subclusters including DC, macrophage, and monocytes. Hub genes of Ctss, Mpeg1, Cybb, H2-Ab1, Ly86, CD74, and Lgals3 were highly expressed in monocytes cluster. Among DC-correlated DEGs, Mmp12 was mainly expressed in monocyte cluster, and Gpnmb was mainly expressed in fibroblast cluster, whereas Atp6v0d2 expression has a weaker signal and weaker cell preference. In conclusion, DC infiltration and its associated pivotal genes may be responsible for progression of myocarditis. Our study expands and provides novel information on the immune cell engagement of myocarditis.

## 1. Introduction

Myocarditis is a widespread disease with cardiac dysfunction in which complex inflammatory cell infiltration (may be focal or diffuse) of myocardial tissue occurs with or without myocardial cell damage [1]. Myocarditis affects people of all ages, although its symptoms usually onset between the ages of 20 and 50, and causes a wide range of clinical manifestations, which makes its diagnosis challenging [2]. Myocarditis continues to threaten human health with a prevalence ranging from 0.06% to 2.4%. In a possible underestimation instance, just in England, myocarditis initial diagnosis occupied 0.04% of all hospital admissions in the period 1998 to 2017 [3]. However, the diagnosis and treatment of myocarditis still face great challenges. These challenges result from a considerable knowledge blank between therapeutic management and alteration in the cardiac immune environment from the autoimmune-induced acute inflammatory phase to the myopathic phase.

Immune cell infiltration exerts a nonnegligible role in the progression of myocarditis. Unlike other myocardial injury mechanisms, the inflammatory state of myocarditis may be secondary to infection, autoimmune-mediated, toxic substance exposure, or maybe idiopathic [4]. According to the type of cellular inflammatory infiltrate, myocarditis can be classified into four types: lymphocytic, eosinophilic, giant-cell myocarditis, and granulomatous. Numerous studies have demonstrated the important role of different immune cells in the prevention of myocarditis. For example, with CD28 superagonists, targeting Treg cells could be used to treat and prevent autoimmune myocarditis [5]. Restraining autoreactive T cells entered the peripheral tissues, and recognizing cardiac peptide by maintaining T cells in an anergic state through PD-1 signaling may lead to cardiogenic shock in patients with myocarditis [6]. Mono-macrophage-derived neurotrophic factor could alleviate bacterial myocarditis by increasing M1 macrophages [7]. Therefore, a greater understanding of the immune cell infiltration in the heart muscle can yield insight into the molecular mechanisms of myocarditis.

In this study, we mined differentially expressed genes (DEGs) by analyzing the RNA-seq and array expression profile data of myocarditis mice and then analyzed the correlation between DEGs and immune cell infiltration. Finally, we obtained the immune cell landscape of myocarditis mice at a single-cell resolution. These results will help reveal in detail the distribution of immune cells in myocarditis and help us understand the value of DEG-related immune cells in the progression of myocarditis.

## 2. Materials and Methods

### 2.1. Gene Expression Omnibus (GEO) Dataset Processing

The GSE35182 (3 + 3, only males included) and GSE53607 (5 + 5, only data in 60-day group included) datasets are expression profile array data and are based on GPL6246 (MoGene-1_0-st) Affymetrix Mouse Gene 1.0 ST Array (transcript (gene) version) platform. In the GSE35182 dataset, mice were infected with coxsackievirus B3 to construct a myocarditis model. In the GSE53607 dataset, C3H mice were infected with TMEV intraperitoneally to construct a myocarditis model. The GSE35182 and GSE35182 dataset were downloaded from the GEO database (https://www.ncbi.nlm.nih.gov/geo/). These two array data were merged and standardized and corrected batch effects by using sva package (3.20) in R. After that, genes were subjected to identify differentially expressed genes (DEGs) between the control group and the myocarditis group. The screening criteria were set as ∣log2FC | >0.585 and adjust *P* < 0.05. Next, DEGs were enriched by GO and KEGG databases to explore the biological function of DEGs.

### 2.2. Protein-Protein Interaction (PPI) Network Construction

All of the DEGs were subjected to the development of PPI network construction using the STRING database (https://string-db.org/). The interaction of a default comprehensive score of more than 0.4 was set as significant. The gene having a molecular complex detection (MCODE) score of more than 3 and an edge number of more than 4 was identified as hub gene. The PPI network between DEGs was visualized by using the Cytoscape 3.6.1 software.

### 2.3. Infiltration of Immune Cells in Myocarditis

Infiltration of immune cells and abundance of each immune cell in myocarditis were accessed by ImmuCellAI database (http://bioinfo.life.hust.edu.cn/ImmuCellAI-mouse/#!/). The correlation of DEGs with immune cells was determined upon the criteria *P* < 0.05. For the correlation between DEGs and immune cells, the “BiocManager” R package was used for analysis, and the correlation coefficient filter criterion was 0.1.

### 2.4. The Single-Cell RNA Sequencing (scRNA-seq) Dataset Processing

The GSE174458 (7 + 7) dataset is expression profiling by high-throughput sequencing and is based on the GPL21103 Illumina HiSeq 4000 platform. In the GSE174458 dataset, mice were infected with coxsackievirus B3 to construct a myocarditis model. R package Seurat (version 4.1.1) was used to process the scRNA-seq dataset. After GSE174458 data was imported into Seurat, quality control was firstly carried out, and the criteria were as follows: (1) genes expressed in less than 3 cells were removed; (2) cells expressing fewer than 50 genes were removed; (3) cells with more than 20% mitochondrial gene expression intensity were removed. Next, scRNA-seq data was standardized by using NormalizeData function, and then, the 1500 genes with the most fluctuations in expression data were then picked for subsequent cluster analysis. Cell annotation was performed by SingR (version 1.8.1) and Celldex (version 1.4.0) package. DEGs were identified for each cell type using the FindAllMarkers function.

## 3. Results

### 3.1. Identification and Functional Enrichment of DEGs in Myocarditis

**Figure 1:**
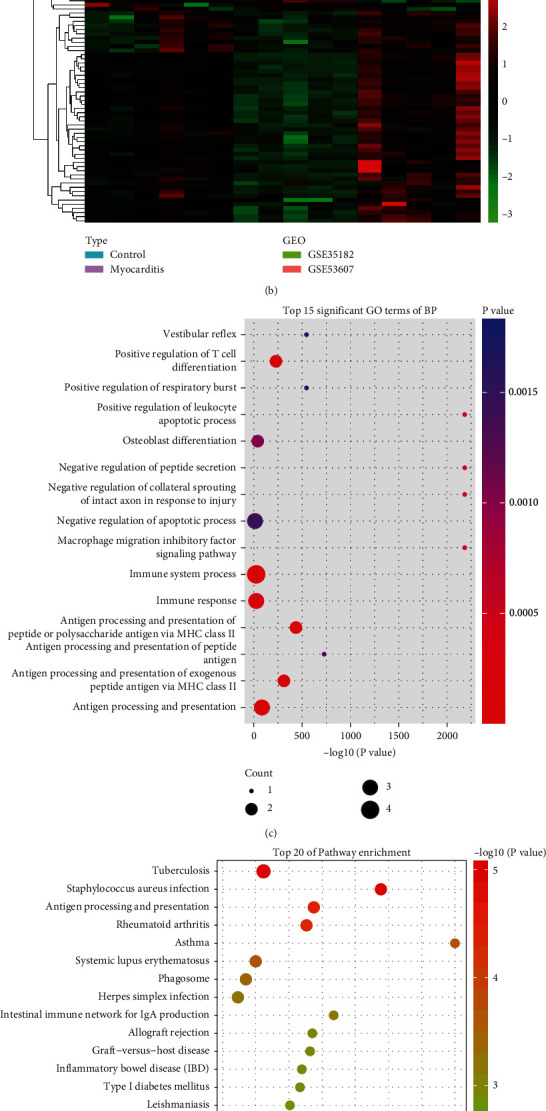
Identification and functional enrichment of DEGs in myocarditis. (a) Volcano plot of DEGs. Red and blue represent upregulated and downregulated DEGs in the myocarditis group relative to the control group. (b) Heatmap plot of DEGs. (c) Top 15 significant GO terms of biological process. (d) Top 20 KEGG enrichment pathways of DEGs.

Gene expression from the merged GSE35182 and GSE35182 datasets was normalized and batch corrected for DEG analysis. A total of 58 DEGs were identified upon the criteria of ∣log2FC | >0.585 and adjust *P* < 0.05, among which 51 DEGs were upregulated and 7 DEGs were downregulated in the myocarditis group compared with the control group (Figure 1(a)). Among the upregulated DEGs, the top three DEGs with the largest fold change were Mmp12, Gpnmb, and Atp6v0d2, respectively (Figure 1(a)). A distinctly different expression pattern was observed between the myocarditis group and the control group in the heatmap (Figure 1(b)). All of these 58 DEGs were enrolled for subsequent analysis.

To obtain insights into the biological functions of DEGs, GO and KEGG enrichment analyses were performed. The result of biological process in GO category revealed that these DEGs were mainly against immune-related processes, including positive regulation of T cell differentiation, immune system process, positive regulation of leukocyte apoptotic process, immune response, macrophage migration inhibitory factor signaling pathway, and regulation of the immune system, as well as antigen presentation-related processes, including antigen processing and presentation of peptide antigen, antigen processing and presentation of peptide or polysaccharide antigen via MHC class II, antigen processing and presentation of exogenous peptide antigen via MHC class II, and antigen processing and presentation (Figure 1(c)). Among the top 15 biological process items, these immune-related and antigen presentation-related processes account for 60% (9/15), suggesting that the DEGs induced by myocarditis were associated with the immune abnormalities of myocarditis patients. Not unexpectedly, KEGG enrichment revealed that immune-related pathways were enriched, such as antigen processing and presentation, cell adhesion molecules (CAMs), and intestinal immune network for IgA production (Figure 1(d)). Collectively, myocarditis-triggered DEGs may be involved in the progression of myocarditis through immune regulation.

### 3.2. Identification of the Hub Genes in Myocarditis

**Figure 2:**
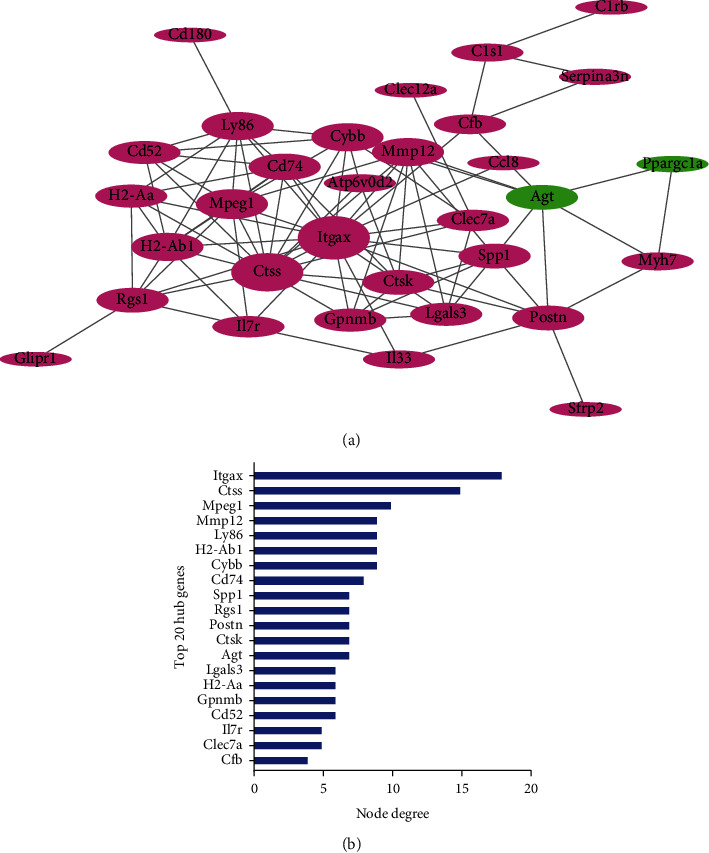
Identification of the hub genes in myocarditis. (a) PPI network of DEGs in STRING database. Red and green represent upregulated and downregulated DEGs in the myocarditis group relative to the control group. Larger ellipse sizes indicate more interacting proteins. (b) Identified the top 20 hub genes. The abscissa represents the number of edges.

To investigate the DEG “hub genes” associated with myocarditis, we constructed a PPI network using all the DEGs based on the STRING database. As shown in Figure 2(a), 32 nodes and 182 edges were identified in the PPI network. Moreover, the DEGs with the number of edges > 4 and MCODE score > 3 were selected, including Itgax, Mmp12, and Gpnmb (Figure 2(b)). Therefore, these 20 central node genes were considered hub genes for further analyses.

### 3.3. Dendritic Cell Was Enriched in the Myocardial Tissue

**Figure 3:**
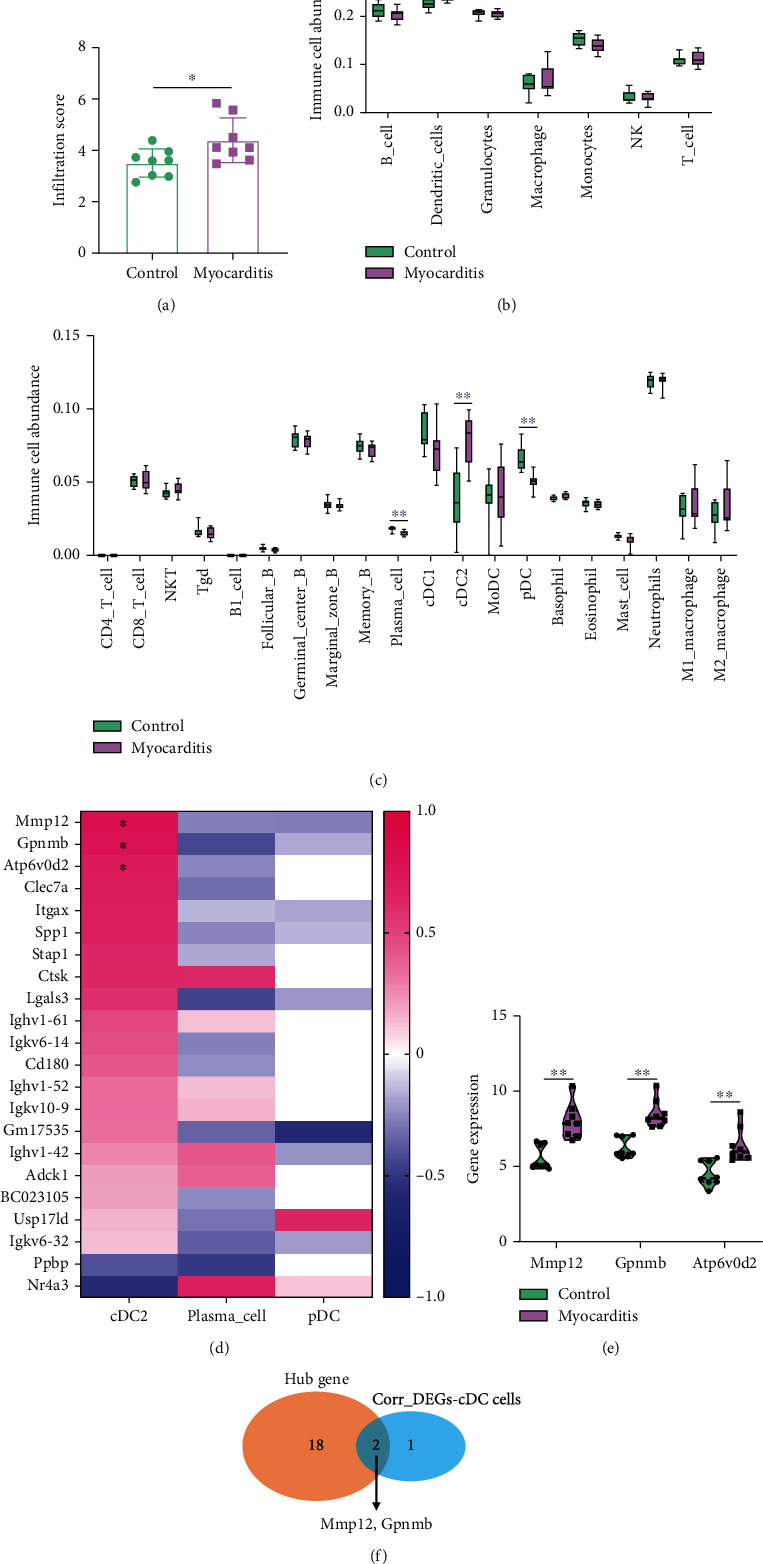
Dendritic cell was enriched in the myocardial tissue. (a) Immune infiltration scores in the myocardial tissue and controls according to the ImmuCellAI database. (b) The abundance of 7 types of immune cells. (c) The abundance of 20 types of immune cells. (d) A heatmap of correlation between DEGs with cDC2, pDC, and plasma cell. (e) The expressions of Mmp12, Gpnmb, and Atp6v0d2 in the myocarditis group. (f) Mmp12 and Gpnmb were overlapped between hub gene list and DC-correlated DEGs. ∗ means *P* < 0.05, and ∗∗ means *P* < 0.01.

To explore the possible effect of myocarditis on different immune cell infiltration, the ImmuCellAI database was used to access the ingredients of 36 immune cells at three layers [8] in the myocardial tissue. Expectedly, myocardial tissue had significantly higher immune infiltration scores than controls (Figure 3(a)). In the first layer composed of 7 types of immune cells, compared with the control group, only the abundance of dendritic cell (DC) was significantly upregulated in the myocarditis tissue, and the abundance of the other 6 types of immune cells had no difference between the two groups (Figure 3(b)). Furthermore, in the second layer composed of 20 types of immune cells, we observed that the occurrence of myocarditis induced significant enrichment of conventional DC2 (cDC2), plasmacytoid DC (pDC), and plasma cells in myocardial tissue (Figure 3(c)). Subsequently, we performed a correlation analysis between DEGs with cDC2, pDC, and plasma cells. Only 22 of the 58 DEGs were associated with these three immune cells upon a filter criterion with an *r* value of less than 0.1 (Figure 3(d)). The expression levels of Mmp12, Gpnmb, and Atp6v0d2 were positively correlated with the abundance of cDC (Figure 3(d)). The expressions of Mmp12, Gpnmb, and Atp6v0d2 were all enhanced in the myocarditis group compared to the control group (Figure 3(e)), of which only Mmp1 and Gpnmb were shared with the hub gene list (Figure 3(f)). Taken together, cDC was enriched in the myocardial tissue and positively correlated with the expression of Mmp12, Gpnmb, and Atp6v0d2.

### 3.4. Quality Control of scRNA-seq Data

**Figure 4:**
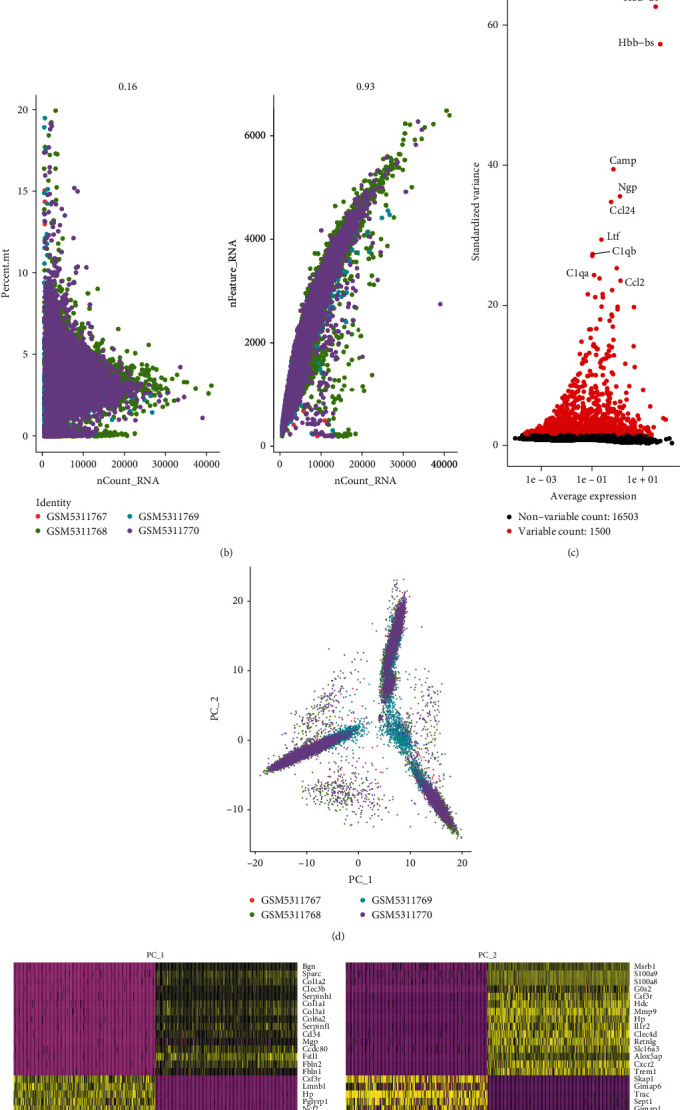
Quality control of scRNA-seq data. (a) After quality control, 2013 nonconforming cells were deleted and remained 20972 cells for further analysis. (b) Correlation analysis of scRNA-seq depth and mitochondrial gene expression. (c) A total of 16,503 genes were analyzed, of which 1,500 genes with high expression variation were selected for subsequent cluster analysis. (d) Principal component analysis for dimensionality reduction for scRNA-seq. (e) Analysis for the top 20 significant difference principal components.

In the present study, a total of 22,985 cells were obtained from the GSE174458 dataset, of which 9,734 cells were from healthy control and 13,251 cells were from myocarditic mice. After quality control using Seurat, the remaining 20,972 single-cell transcriptomes were retained for subsequent analysis (Figure 4(a)). Cells with more than 20% mitochondrial gene expression intensity were removed thereby mitochondrial genes did not affect the sequencing depth (Figure 4(b)). A total of 16,503 genes were analyzed, of which 1,500 genes with high expression variation were selected for subsequent cluster analysis (Figure 4(c)). Preliminary dimensionality reduction was carried out by principal component analysis (PCA) for scRNA-seq data, and we found no clear segregation among cardiac muscle cells (Figure 4(d)); we then performed further analysis for the top 20 significant difference principal components (Figure 4(e)). These results indicated that scRNA-seq data could be used in subsequent analysis.

### 3.5. Comprehensive Analysis of scRNA-seq Data of Myocarditis

**Figure 5:**
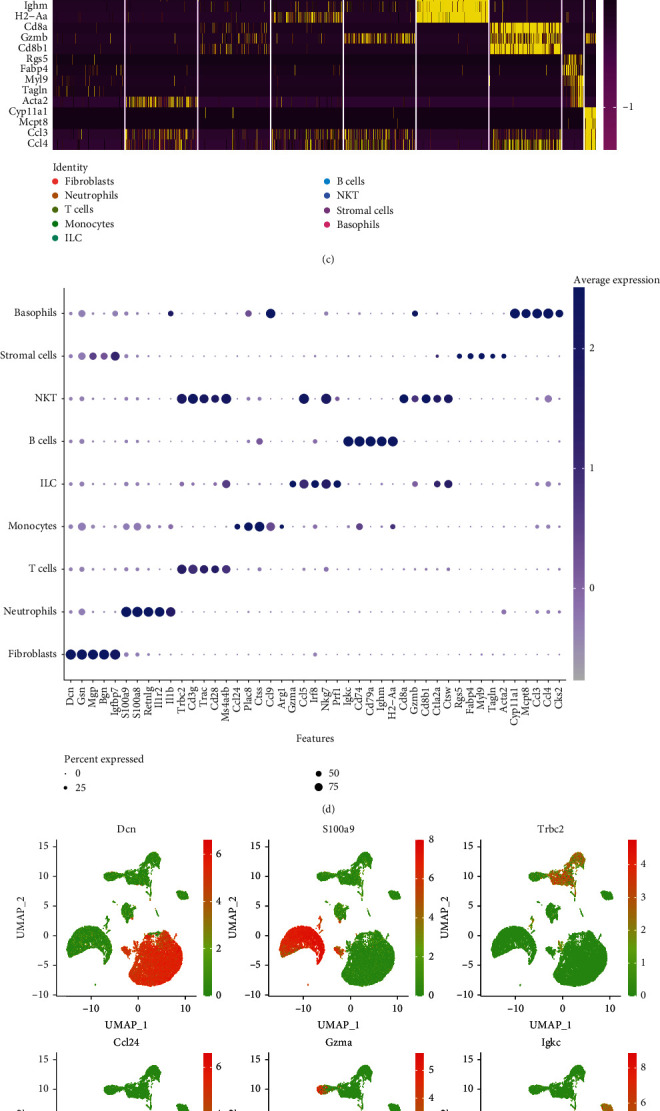
Comprehensive analysis of scRNA-seq data of myocarditis. (a) Mapping of cell clusters using an unbiased nonlinear dimension reduction of UMAP cluster based on scRNA-seq data. (b) Annotating cell types. Nine major cell types were annotated based on the canonical cell markers. (c) A total of 9136 marker genes were aggregated into 26 clusters, and the top 5 marker genes in each cell type were exhibited. (d) The expression of the top 5 marker genes in each cell type was visualized by bubble plot. (e) The distribution of the most abundantly expressed marker gene in each cell type was visualized on the UMAP plot. (f) Relative distribution of 9 cell types in the myocarditis group and the control group.

To visualize the distribution of the scRNA-seq data, an unbiased nonlinear dimension reduction of uniform manifold approximation and projection (UMAP) yielded 26 cell clusters (Figure 5(a)). Next, using canonical markers for indicated cell types (Supplemental Table 1), we annotated 9 cell types, including fibroblasts, neutrophils, stromal cells, monocytes, basophils, B cells, natural killer T cells, innate lymphoid cells, and T cells (Figure 5(b)). A total of 9,136 marker genes were recognized, and the expressions of the top 5 marker genes in the 9 cell types were visualized as heatmap (Figure 5(c)) and bubble plot (Figure 5(d)). As we can see, fibroblasts mainly express Dcn, Gsn, Mgp, and Bgn, and monocytes mainly express Ccl4, Plac8, Ctss, and Arg1. Furthermore, the scatter UMAP plots presented the distribution of the most abundantly expressed marker gene in each cell type (Figure 5(e)). Intriguingly, the proportion of monocytes and natural killer T cells in the myocarditis group was clearly higher than that in the control group, and the remaining cell types showed no significant difference (Figure 5(f)). Taken together, the results of scRNA-seq reemphasized the important role of immune cells in myocarditis.

### 3.6. Integration Analysis of Monocyte Cluster and Hub Gene Reveals DC-Related Mmp12 Is a Key Gene in Myocarditis

**Figure 6:**
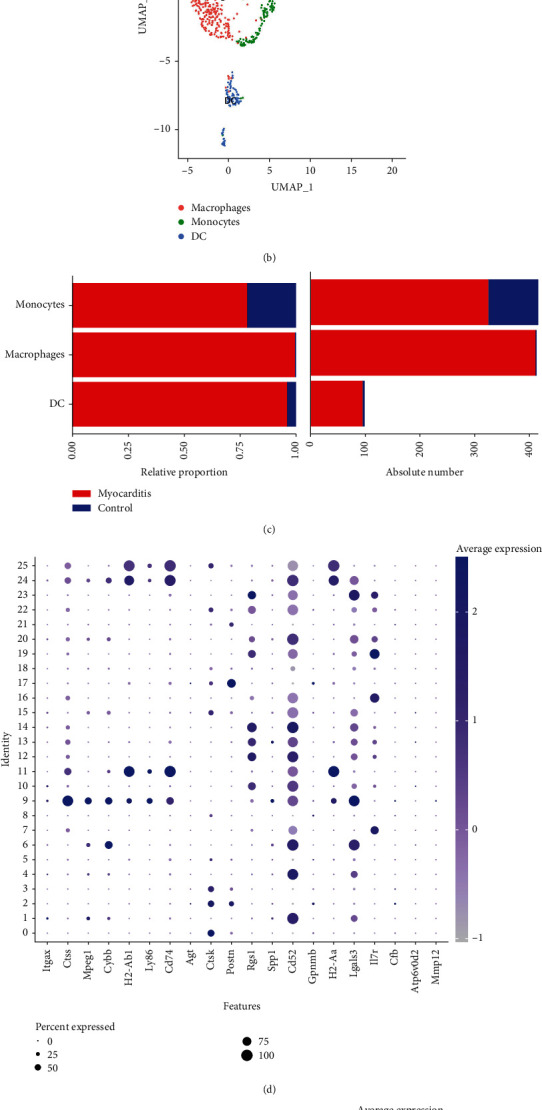
Integration analysis of monocyte cluster and hub gene reveals that DC-related Mmp12 is a key gene in myocarditis. (a) The expression of top 10 marker genes of monocyte cluster 9 in 26 clusters was displayed on the heatmap. (b) UMAP of monocytes identified in heart infiltrates was determined 3 subclusters. (c) Relative distribution of 3 monocyte subclusters in the myocarditis group and the control group. (d) The expression and distribution of the hub genes in the 26 clusters were visualized on the bubble plot. (e) The expression and distribution of Mmp12, Gpnmb, and Atp6v0d2 in the 26 clusters were visualized on the bubble plot.

Since the above analysis found that cDC was enriched in myocarditis tissues (Figure 3), we focused on cluster 9 of monocytes, which mainly expressed Ctss, Ccl9, and Tgfbi (Figure 6(a)). In dissecting the monocyte complexity, we determined 3 subclusters including DC, macrophage, and monocytes by using the above indicated markers (Figure 6(b)). The abundance of these three cells was significantly higher in the myocarditis group than in the control tissue (Figure 6(c)). Furthermore, we characterized the single-cell expression profiles of hub genes displayed in scatter UMAP plots, as well as three DEGs significantly correlated with DC (Supplemental Figure 1). The results showed that hub genes of Ctss, Mpeg1, Cybb, H2-Ab1, Ly86, CD74, and Lgals3 were highly expressed in the monocytes cluster (Figure 6(d)). In addition, among three DC-correlated DEGs, Mmp12 was mainly expressed in monocyte cluster 9, and Gpnmb was mainly expressed in fibroblast clusters 2, 8, and 17, whereas the expression of Atp6v0d2 has a weaker signal and weaker cell preference in the single-cell expression profile (Figure 6(e) and Supplemental Figure 2). Taken together, these results suggested that Mmp12 is a key gene involved in the progression of myocarditis due to DC infiltration.

## 4. Discussion

Myocarditis is a complex inflammatory disease accompanied by immune dysfunction that causes cardiogenic shock in 3.2% (11/337) of cases [9]. Myocarditis exhibited a wide spectrum of clinical manifestations, with the vast majority presenting with nonspecific systemic symptoms such as gastroenteritis, myalgia, fever, or respiratory symptoms [10], in addition to the possibility of arrhythmias, palpitations, and exertional dyspnea [11, 12] in patients with myocarditis. This varies widely in clinical presentation leading to the diagnosis being sometimes difficult to establish, and numerous therapies for myocarditis have not demonstrated survival benefits [13]. Therefore, a lot of research is still needed to deeply analyze the underlying mechanism of myocarditis infiltration to provide the basis for the diagnosis and treatment of myocarditis. In this study, the combined analysis of DEGs and immune cell infiltration in myocarditic tissue found that Mmp12, Atp6v0d2, and Gpnmb were significantly positively correlated with DC cell infiltration; subsequently, scRNA-seq data analysis confirmed that the enrichment of DC in myocarditic tissue was related to Mmp12.

The scRNA-seq is a powerful and practical technique that can characterize cell diversity and heterogeneity in unprecedented detail to dissect the complexity of diseases. Recently, scRNA-seq has uncovered the complexity of tumor-infiltrating myeloid cells in several different cancers, such as DC and tumor-associated macrophages [14–16]. However, the immune infiltration of myocardial tissue under different physiological and pathological conditions is rarely studied. In experimental autoimmune myocarditis (EAM) model at different phases, scRNA-seq identified 26 cell subtypes among 34665 cells and found that Hif1a contributes to the inflammatory response mainly through the regulation of macrophage and T-helper 17 cells [17]. A recent study revealed that myeloid cells, T cells, and fibroblasts play a critical role in the cytotoxic functions and inflammation and immune response in viral myocarditis [18]. Pathogenic immune cell subsets in checkpoint inhibitor-induced myocarditis model [19] and peripheral immune landscape in BNT162b2 mRNA vaccine-induced myocarditis model [20] were described by scRNA-seq. These reports dissect the cellular landscape and transcriptome of four myocarditis models and initially reveal the unique roles of different immune cell types in inflammation and immune responses in myocarditis. However, there are only four studies about scRNA-seq in myocarditis in the PubMed database, and this huge blank urges the advancement of more scRNA-seq work. In this context, we reanalyzed scRNA-seq data (GSE174458) from the aforementioned viral myocarditis model combined with hub gene analysis. We found that monocyte cluster 9 had signatures critical for immune responses, particularly of DC, and Mmp12 contributes to the DC infiltration in the pathogenesis of viral myocarditis. Our research is not only a reuse of existing resources but also a supplement to the original data.

DC originates from monocytes and is intermediate mediator of adaptive immunity and can be subdivided into 4 subtypes: cDC1, cDC2, pDC, and monocyte-derived DC [21]. In recent years, the function of DC cells in myocarditis has gradually been recognized. For example, the modulating DC function by targeting NLRP3 inflammasome through miR-223-3p can ameliorate the EAM [22]. Inhibition of the accumulation of DC in the inflamed myocardium by MCS-18 treatment could mitigate the EAM [23]. Driving DC activation and Th17 differentiation by tenascin-C aggravates EAM progression through Toll-like receptor 4 [24]. These results affirm the contribution of DC function in the pathological progression of myocarditis. Our results are concordant with this, and we disclose that DC is significantly enriched in myocarditis tissue both in ImmuCellAI database and scRNA-seq data. In conclusion, our study reemphasizes the abnormal enrichment and promoting role of DC in myocarditis. Interestingly, in the present study, KEGG enrichment revealed that DEGs were mainly enriched in immune-related pathways, such as antigen processing and presentation. It is well known that DC is the most important antigen presenting cells [25]. A study observed that infiltration of DC and monocyte in the heart and self-antigen presentation by cDC2 is induced by myocarditis [26]. Impaired antigen-presentation capacity of DC was also observed in enterovirus myocarditis [27]. These results suggest that DEG-involved antigen processing and presentation pathway may couple DC functions to participate in myocarditis disease progression.

Moreover, KEGG enrichment also found that DEGs were mainly enriched in the CAMs pathway, implicating that CAMs may have a potential role in the progression of myocarditis. For example, the mediation role of CAMs in ventricular pacing associated with myocardial inflammatory responses has been uncovered [28]. Of note, vascular CAM-1 has been shown to be a biomarker of experimental autoimmune myocarditis [29]. These studies support our results. DEGs are also enriched in the intestinal immune network for IgA production pathway. Interestingly, intravenous immunoglobulin may effectively improve pediatric myocarditis [30], suggesting that immunoglobulin has the possibility of becoming a medication for myocarditis.

In this study, we identified 51 DEGs were upregulated in the myocarditis group compared with the control group, and the top three DEGs with the largest fold change were Mmp12, Gpnmb, and Atp6v0d2. All three genes were significantly positively correlated with the abundance of DC in myocarditic tissues, but only Mmp12 and Gpnmb were identified as hub genes. Furthermore, according to the scRNA-seq data, only Mmp12 was expressed in monocyte cluster 9, while Gpnmb was expressed in fibroblast clusters 2, 8, and 17. Therefore, we concluded that Mmp12 is a key gene involved in the progression of myocarditis due to DC infiltration. Mmp12 was the most abundant matrix metallopeptidase in the conditioned medium of DC revealed by proteome profile [24]. Moreover, Mmp12 knockout mice lose the immune surveillance ability characterized by immature myeloid cells accumulated and cannot differentiate to DC, macrophages, or neutrophils [31]. These results support our conclusion that Mmp12 plays an important role in DC function. MMPs also contributed to medication of myocarditis; for example, inhibition of MMP activity contributed to early clarithromycin treatment in rat autoimmune myocarditis [32] or nonbacterial myocarditis [33].

## 5. Conclusions

In conclusion, we profiled 58 DEGs and predicted immune cell infiltration and characterized single-cell profile in myocarditic tissues. Based on integrated DEGs, DC infiltration, and scRNA-seq data analysis, we revealed that the DC-related gene such as Mmp12 expression signature is an important clue for understanding the pathology of myocarditis. Our study provides evidence for immunotherapy and provides a research direction for the future DC-targeted therapy of myocarditis.

## Data Availability

All data used to support the findings of this study can be found in the article.

## Supplementary Materials

Supplementary Materials See Figures S1-S2 and Table S1in the file of Supplementary Material.
