# Supplementary material for: Integrated Single-Cell and RNA Sequencing Analysis Identifies Key Immune Cell and Dendritic Cells Associated Genes Participated in Myocarditis
Source: J Immunol Res. 2022 Oct 3;2022:8655343. doi: 10.1155/2022/8655343 (PMC9550476; doi:10.1155/2022/8655343)
Supplement: Supplementary Materials — See Figures S1-S2 and Table S1in the file of Supplementary Material. [file 8655343.f1.zip › Supplemental Table 1.pdf]

| p_val | avg_log2F | pct.1 | pct.2 | p_val_adj | cluster | gene |
|---|---|---|---|---|---|---|
| 0 | 6.241712 | 0.991 | 0.273 | 0 | Fibroblas | Dcn |
| 0 | 5.791857 | 0.992 | 0.537 | 0 | Fibroblas | Gsn |
| 0 | 5.09981 | 0.967 | 0.148 | 0 | Fibroblas | Mgp |
| 0 | 4.755996 | 0.978 | 0.082 | 0 | Fibroblas | Bgn |
| 0 | 4.682293 | 0.984 | 0.135 | 0 | Fibroblas | Igfbp7 |
| 0 | 4.574651 | 0.972 | 0.07 | 0 | Fibroblas | Htra3 |
| 0 | 4.458437 | 0.962 | 0.068 | 0 | Fibroblas | Sparc |
| 0 | 4.401816 | 0.954 | 0.048 | 0 | Fibroblas | Clec3b |
| 0 | 4.273037 | 0.891 | 0.046 | 0 | Fibroblas | Lpl |
| 0 | 4.233677 | 0.969 | 0.071 | 0 | Fibroblas | Sparcl1 |
| 0 | 4.187381 | 0.913 | 0.069 | 0 | Fibroblas | Col3a1 |
| 0 | 4.18565 | 0.96 | 0.057 | 0 | Fibroblas | Lum |
| 0 | 4.177863 | 0.966 | 0.036 | 0 | Fibroblas | Serping1 |
| 0 | 4.000315 | 0.941 | 0.041 | 0 | Fibroblas | Col1a2 |
| 0 | 3.859724 | 0.919 | 0.036 | 0 | Fibroblas | Serpinh1 |
| 0 | 3.857957 | 0.939 | 0.044 | 0 | Fibroblas | Gpx3 |
| 0 | 3.829328 | 0.915 | 0.039 | 0 | Fibroblas | Col1a1 |
| 0 | 3.829208 | 0.908 | 0.035 | 0 | Fibroblas | Fbln1 |
| 0 | 3.732133 | 0.869 | 0.19 | 0 | Fibroblas | Mt1 |
| 0 | 3.713823 | 0.945 | 0.035 | 0 | Fibroblas | Pcolce |
| 0 | 3.670387 | 0.953 | 0.051 | 0 | Fibroblas | Ltbp4 |
| 0 | 3.637268 | 0.965 | 0.327 | 0 | Fibroblas | Cebpd |
| 0 | 3.625733 | 0.756 | 0.054 | 0 | Fibroblas | Hspb1 |
| 0 | 3.483854 | 0.95 | 0.029 | 0 | Fibroblas | Cygb |
| 0 | 3.476809 | 0.913 | 0.159 | 0 | Fibroblas | Egr1 |
| 0 | 3.38306 | 0.935 | 0.327 | 0 | Fibroblas | Jun |
| 0 | 3.352701 | 0.87 | 0.028 | 0 | Fibroblas | Smoc2 |
| 0 | 3.290302 | 0.902 | 0.028 | 0 | Fibroblas | Col6a1 |
| 0 | 3.257051 | 0.92 | 0.03 | 0 | Fibroblas | Cd34 |
| 0 | 3.23749 | 0.792 | 0.033 | 0 | Fibroblas | Mfap5 |
| 0 | 3.201867 | 0.879 | 0.027 | 0 | Fibroblas | Ccdc80 |
| 0 | 3.185358 | 0.896 | 0.027 | 0 | Fibroblas | Serpinf1 |
| 0 | 3.182546 | 0.914 | 0.028 | 0 | Fibroblas | Mmp2 |
| 0 | 3.182187 | 0.887 | 0.022 | 0 | Fibroblas | Col6a2 |
| 0 | 3.173664 | 0.619 | 0.041 | 0 | Fibroblas | Cxcl1 |
| 0 | 3.155207 | 0.862 | 0.026 | 0 | Fibroblas | Col15a1 |
| 0 | 3.152484 | 0.896 | 0.04 | 0 | Fibroblas | Cfh |
| 0 | 3.151253 | 0.883 | 0.025 | 0 | Fibroblas | Fbln2 |
| 0 | 3.151136 | 0.948 | 0.032 | 0 | Fibroblas | Rarres2 |
| 0 | 3.073758 | 0.958 | 0.116 | 0 | Fibroblas | Cd81 |
| 0 | 3.070649 | 0.317 | 0.016 | 0 | Fibroblas | Cxcl14 |
| 0 | 3.026673 | 0.732 | 0.018 | 0 | Fibroblas | Meg3 |
| 0 | 3.007128 | 0.738 | 0.019 | 0 | Fibroblas | Dpep1 |
| 0 | 2.993733 | 0.844 | 0.108 | 0 | Fibroblas | Klf4 |
| 0 | 2.957574 | 0.881 | 0.03 | 0 | Fibroblas | Fstl1 |
| 0 | 2.929779 | 0.901 | 0.023 | 0 | Fibroblas | Dpt |
| 0 | 2.922159 | 0.929 | 0.068 | 0 | Fibroblas | Gstm1 |
| 0 | 2.885499 | 0.943 | 0.115 | 0 | Fibroblas | Igfbp4 |
| 0 | 2.869017 | 0.872 | 0.023 | 0 | Fibroblas | Nid1 |
| 0 | 2.820109 | 0.593 | 0.019 | 0 | Fibroblas | Cyr61 |
| 0 | 2.792105 | 0.822 | 0.018 | 0 | Fibroblas | Gas1 |
| 0 | 2.790571 | 0.926 | 0.025 | 0 | Fibroblas | Lhfp |
| 0 | 2.779679 | 0.95 | 0.182 | 0 | Fibroblas | Ctsl |

| | | | | | | |
|---|---|---|---|---|---|---|
| 0 | 2.769986 | 0.867 | 0.282 | 0 | Fibroblas | Pi16 |
| 0 | 2.734558 | 0.895 | 0.024 | 0 | Fibroblas | Pmp22 |
| 0 | 2.726921 | 0.934 | 0.114 | 0 | Fibroblas | Zbtb20 |
| 0 | 2.67426 | 0.873 | 0.023 | 0 | Fibroblas | Nfix |
| 0 | 2.668038 | 0.86 | 0.022 | 0 | Fibroblas | Hspg2 |
| 0 | 2.661412 | 0.875 | 0.023 | 0 | Fibroblas | Rbp1 |
| 0 | 2.661101 | 0.839 | 0.021 | 0 | Fibroblas | Col4a1 |
| 0 | 2.657951 | 0.786 | 0.098 | 0 | Fibroblas | Ly6a |
| 0 | 2.656257 | 0.929 | 0.038 | 0 | Fibroblas | Selenom |
| 0 | 2.640362 | 0.876 | 0.034 | 0 | Fibroblas | Axl |
| 0 | 2.635108 | 0.832 | 0.088 | 0 | Fibroblas | Id3 |
| 0 | 2.622129 | 0.824 | 0.015 | 0 | Fibroblas | Pcsk6 |
| 0 | 2.606813 | 0.9 | 0.049 | 0 | Fibroblas | Lrp1 |
| 0 | 2.582505 | 0.778 | 0.033 | 0 | Fibroblas | Ckb |
| 0 | 2.574819 | 0.857 | 0.017 | 0 | Fibroblas | Loxl1 |
| 0 | 2.560858 | 0.97 | 0.21 | 0 | Fibroblas | Cd63 |
| 0 | 2.534107 | 0.848 | 0.017 | 0 | Fibroblas | Prelp |
| 0 | 2.518151 | 0.83 | 0.017 | 0 | Fibroblas | Plpp3 |
| 0 | 2.515695 | 0.896 | 0.17 | 0 | Fibroblas | Crispld2 |
| 0 | 2.496984 | 0.84 | 0.02 | 0 | Fibroblas | Tnxb |
| 0 | 2.493252 | 0.871 | 0.04 | 0 | Fibroblas | Lamc1 |
| 0 | 2.488346 | 0.856 | 0.026 | 0 | Fibroblas | Slc43a3 |
| 0 | 2.476219 | 0.943 | 0.249 | 0 | Fibroblas | Anxa5 |
| 0 | 2.467021 | 0.77 | 0.015 | 0 | Fibroblas | Ogn |
| 0 | 2.461419 | 0.802 | 0.018 | 0 | Fibroblas | Lamb1 |
| 0 | 2.453686 | 0.869 | 0.02 | 0 | Fibroblas | Sdc2 |
| 0 | 2.423712 | 0.857 | 0.02 | 0 | Fibroblas | Mxra8 |
| 0 | 2.416875 | 0.845 | 0.024 | 0 | Fibroblas | Abca8a |
| 0 | 2.416216 | 0.805 | 0.061 | 0 | Fibroblas | Ecm1 |
| 0 | 2.401188 | 0.435 | 0.015 | 0 | Fibroblas | Mfap4 |
| 0 | 2.396018 | 0.903 | 0.154 | 0 | Fibroblas | Mtch1 |
| 0 | 2.383411 | 0.777 | 0.01 | 0 | Fibroblas | Islr |
| 0 | 2.381006 | 0.884 | 0.063 | 0 | Fibroblas | Klf9 |
| 0 | 2.373134 | 0.851 | 0.05 | 0 | Fibroblas | Crip2 |
| 0 | 2.365872 | 0.857 | 0.015 | 0 | Fibroblas | Nfib |
| 0 | 2.364037 | 0.825 | 0.021 | 0 | Fibroblas | Gpm6b |
| 0 | 2.359321 | 0.866 | 0.032 | 0 | Fibroblas | Rcn3 |
| 0 | 2.353622 | 0.787 | 0.236 | 0 | Fibroblas | Socs3 |
| 0 | 2.311493 | 0.898 | 0.056 | 0 | Fibroblas | Nenf |
| 0 | 2.302893 | 0.86 | 0.302 | 0 | Fibroblas | Hspa1b |
| 0 | 2.297163 | 0.798 | 0.01 | 0 | Fibroblas | Tcf21 |
| 0 | 2.294079 | 0.664 | 0.151 | 0 | Fibroblas | Apoe |
| 0 | 2.293227 | 0.894 | 0.066 | 0 | Fibroblas | Rnase4 |
| 0 | 2.291328 | 0.929 | 0.331 | 0 | Fibroblas | Tuba1a |
| 0 | 2.287531 | 0.711 | 0.012 | 0 | Fibroblas | Col8a1 |
| 0 | 2.275409 | 0.808 | 0.016 | 0 | Fibroblas | Olfml3 |
| 0 | 2.272729 | 0.868 | 0.064 | 0 | Fibroblas | Tcf4 |
| 0 | 2.272397 | 0.842 | 0.016 | 0 | Fibroblas | Rhoj |
| 0 | 2.265369 | 0.898 | 0.138 | 0 | Fibroblas | Sptbn1 |
| 0 | 2.258854 | 0.872 | 0.046 | 0 | Fibroblas | Cd302 |
| 0 | 2.249675 | 0.783 | 0.027 | 0 | Fibroblas | Spry1 |
| 0 | 2.236685 | 0.849 | 0.083 | 0 | Fibroblas | Serpinb6a |
| 0 | 2.223061 | 0.821 | 0.017 | 0 | Fibroblas | Cpq |
| 0 | 2.221877 | 0.909 | 0.636 | 0 | Fibroblas | Fos |

| | | | | | | |
|---|---|---|---|---|---|---|
| 0 | 2.206061 | 0.932 | 0.18 | 0 | Fibroblas | Cyb5a |
| 0 | 2.204785 | 0.643 | 0.01 | 0 | Fibroblas | Spon2 |
| 0 | 2.188465 | 0.508 | 0.017 | 0 | Fibroblas | Ctgf |
| 0 | 2.187738 | 0.733 | 0.011 | 0 | Fibroblas | Adamts5 |
| 0 | 2.155388 | 0.691 | 0.018 | 0 | Fibroblas | Timp3 |
| 0 | 2.148102 | 0.764 | 0.015 | 0 | Fibroblas | Oaf |
| 0 | 2.141603 | 0.542 | 0.038 | 0 | Fibroblas | Mt2 |
| 0 | 2.135343 | 0.647 | 0.017 | 0 | Fibroblas | Aspn |
| 0 | 2.131067 | 0.788 | 0.013 | 0 | Fibroblas | Ptgis |
| 0 | 2.126569 | 0.852 | 0.051 | 0 | Fibroblas | Nedd4 |
| 0 | 2.113845 | 0.692 | 0.011 | 0 | Fibroblas | Pcolce2 |
| 0 | 2.104526 | 0.752 | 0.02 | 0 | Fibroblas | Pam |
| 0 | 2.10435 | 0.934 | 0.2 | 0 | Fibroblas | Selenop |
| 0 | 2.091383 | 0.708 | 0.012 | 0 | Fibroblas | Col6a3 |
| 0 | 2.084352 | 0.785 | 0.016 | 0 | Fibroblas | Plxdc2 |
| 0 | 2.08411 | 0.711 | 0.035 | 0 | Fibroblas | Atf5 |
| 0 | 2.082043 | 0.801 | 0.009 | 0 | Fibroblas | Bicc1 |
| 0 | 2.073528 | 0.939 | 0.271 | 0 | Fibroblas | Laptm4a |
| 0 | 2.07214 | 0.763 | 0.012 | 0 | Fibroblas | Ramp2 |
| 0 | 2.068852 | 0.788 | 0.015 | 0 | Fibroblas | Cavin3 |
| 0 | 2.029463 | 0.777 | 0.012 | 0 | Fibroblas | Lama2 |
| 0 | 2.015071 | 0.616 | 0.012 | 0 | Fibroblas | Nbl1 |
| 0 | 1.993474 | 0.811 | 0.016 | 0 | Fibroblas | Fxyd1 |
| 0 | 1.992633 | 0.768 | 0.092 | 0 | Fibroblas | Errfi1 |
| 0 | 1.988165 | 0.812 | 0.177 | 0 | Fibroblas | Pmepa1 |
| 0 | 1.968313 | 0.762 | 0.038 | 0 | Fibroblas | Nupr1 |
| 0 | 1.96818 | 0.985 | 0.696 | 0 | Fibroblas | Cst3 |
| 0 | 1.967625 | 0.754 | 0.012 | 0 | Fibroblas | Col5a1 |
| 0 | 1.962545 | 0.791 | 0.299 | 0 | Fibroblas | Hspa1a |
| 0 | 1.961406 | 0.777 | 0.02 | 0 | Fibroblas | Ddah2 |
| 0 | 1.950349 | 0.743 | 0.01 | 0 | Fibroblas | Adamts2 |
| 0 | 1.94081 | 0.633 | 0.012 | 0 | Fibroblas | Gfpt2 |
| 0 | 1.937923 | 0.705 | 0.011 | 0 | Fibroblas | Fbn1 |
| 0 | 1.935616 | 0.734 | 0.01 | 0 | Fibroblas | Fbln5 |
| 0 | 1.920891 | 0.751 | 0.009 | 0 | Fibroblas | Entpd2 |
| 0 | 1.902007 | 0.574 | 0.151 | 0 | Fibroblas | C3 |
| 0 | 1.888182 | 0.805 | 0.076 | 0 | Fibroblas | Camk2n1 |
| 0 | 1.867108 | 0.966 | 0.546 | 0 | Fibroblas | Vim |
| 0 | 1.866808 | 0.732 | 0.013 | 0 | Fibroblas | Col5a2 |
| 0 | 1.866036 | 0.749 | 0.015 | 0 | Fibroblas | Fhl1 |
| 0 | 1.857884 | 0.755 | 0.01 | 0 | Fibroblas | Pdgfra |
| 0 | 1.854655 | 0.623 | 0.012 | 0 | Fibroblas | Eln |
| 0 | 1.842787 | 0.51 | 0.01 | 0 | Fibroblas | Ackr3 |
| 0 | 1.842069 | 0.779 | 0.052 | 0 | Fibroblas | Nfic |
| 0 | 1.835874 | 0.707 | 0.016 | 0 | Fibroblas | Ace |
| 0 | 1.832779 | 0.806 | 0.082 | 0 | Fibroblas | Rhoc |
| 0 | 1.827911 | 0.789 | 0.249 | 0 | Fibroblas | Fosb |
| 0 | 1.819579 | 0.604 | 0.008 | 0 | Fibroblas | Gsta3 |
| 0 | 1.808015 | 0.701 | 0.008 | 0 | Fibroblas | Medag |
| 0 | 1.806922 | 0.907 | 0.318 | 0 | Fibroblas | Rhob |
| 0 | 1.804007 | 0.681 | 0.023 | 0 | Fibroblas | Selenbp1 |
| 0 | 1.797572 | 0.745 | 0.023 | 0 | Fibroblas | Nfia |
| 0 | 1.797403 | 0.653 | 0.071 | 0 | Fibroblas | Uap1 |
| 0 | 1.79475 | 0.979 | 0.425 | 0 | Fibroblas | Ifitm3 |

| | | | | | | |
|---|---|---|---|---|---|---|
| 0 | 1.790589 | 0.667 | 0.017 | 0 | Fibroblas | Serpine2 |
| 0 | 1.788294 | 0.763 | 0.013 | 0 | Fibroblas | S100a16 |
| 0 | 1.78161 | 0.709 | 0.015 | 0 | Fibroblas | Sod3 |
| 0 | 1.777742 | 0.708 | 0.02 | 0 | Fibroblas | Emilin1 |
| 0 | 1.773558 | 0.644 | 0.01 | 0 | Fibroblas | Htra1 |
| 0 | 1.768221 | 0.747 | 0.018 | 0 | Fibroblas | Pkd2 |
| 0 | 1.752505 | 0.747 | 0.026 | 0 | Fibroblas | Itgb5 |
| 0 | 1.750936 | 0.85 | 0.126 | 0 | Fibroblas | Tmed3 |
| 0 | 1.748781 | 0.673 | 0.014 | 0 | Fibroblas | Ppp1r14a |
| 0 | 1.742074 | 0.751 | 0.049 | 0 | Fibroblas | Slc29a1 |
| 0 | 1.739542 | 0.81 | 0.055 | 0 | Fibroblas | Fcgrt |
| 0 | 1.729884 | 0.75 | 0.014 | 0 | Fibroblas | Fkbp9 |
| 0 | 1.724576 | 0.716 | 0.01 | 0 | Fibroblas | Mxra7 |
| 0 | 1.70444 | 0.858 | 0.173 | 0 | Fibroblas | Ybx3 |
| 0 | 1.702481 | 0.711 | 0.138 | 0 | Fibroblas | Pnp |
| 0 | 1.702189 | 0.693 | 0.01 | 0 | Fibroblas | Lama4 |
| 0 | 1.699645 | 0.902 | 0.223 | 0 | Fibroblas | Slc25a4 |
| 0 | 1.699528 | 0.559 | 0.084 | 0 | Fibroblas | Ugdh |
| 0 | 1.682795 | 0.674 | 0.006 | 0 | Fibroblas | Aebp1 |
| 0 | 1.675365 | 0.694 | 0.02 | 0 | Fibroblas | Nav1 |
| 0 | 1.671557 | 0.601 | 0.009 | 0 | Fibroblas | Col5a3 |
| 0 | 1.655779 | 0.738 | 0.016 | 0 | Fibroblas | Cald1 |
| 0 | 1.655614 | 0.721 | 0.01 | 0 | Fibroblas | Fermt2 |
| 0 | 1.650679 | 0.66 | 0.02 | 0 | Fibroblas | Angptl2 |
| 0 | 1.642508 | 0.597 | 0.006 | 0 | Fibroblas | Sfrp1 |
| 0 | 1.628557 | 0.765 | 0.047 | 0 | Fibroblas | Vkorc1 |
| 0 | 1.627836 | 0.62 | 0.019 | 0 | Fibroblas | Pla1a |
| 0 | 1.620069 | 0.923 | 0.295 | 0 | Fibroblas | Bsg |
| 0 | 1.617172 | 0.581 | 0.036 | 0 | Fibroblas | Bag3 |
| 0 | 1.612252 | 0.778 | 0.077 | 0 | Fibroblas | Txndc5 |
| 0 | 1.609236 | 0.764 | 0.064 | 0 | Fibroblas | Calu |
| 0 | 1.608903 | 0.911 | 0.427 | 0 | Fibroblas | Calr |
| 0 | 1.602703 | 0.899 | 0.343 | 0 | Fibroblas | Hsp90b1 |
| 0 | 1.595889 | 0.565 | 0.008 | 0 | Fibroblas | Fibin |
| 0 | 1.595851 | 0.915 | 0.339 | 0 | Fibroblas | Aldh2 |
| 0 | 1.595444 | 0.748 | 0.054 | 0 | Fibroblas | Rras |
| 0 | 1.589339 | 0.731 | 0.169 | 0 | Fibroblas | Phlda1 |
| 0 | 1.58096 | 0.674 | 0.014 | 0 | Fibroblas | Il11ra1 |
| 0 | 1.578209 | 0.928 | 0.344 | 0 | Fibroblas | Timp2 |
| 0 | 1.577842 | 0.776 | 0.109 | 0 | Fibroblas | Emp1 |
| 0 | 1.567125 | 0.702 | 0.084 | 0 | Fibroblas | Itm2a |
| 0 | 1.565724 | 0.653 | 0.006 | 0 | Fibroblas | Vwa1 |
| 0 | 1.565349 | 0.657 | 0.009 | 0 | Fibroblas | Dpysl3 |
| 0 | 1.564932 | 0.449 | 0.015 | 0 | Fibroblas | Tnfaip6 |
| 0 | 1.558169 | 0.521 | 0.007 | 0 | Fibroblas | Fmo2 |
| 0 | 1.557529 | 0.627 | 0.011 | 0 | Fibroblas | Col4a2 |
| 0 | 1.554954 | 0.704 | 0.016 | 0 | Fibroblas | Fgfr1 |
| 0 | 1.55275 | 0.65 | 0.017 | 0 | Fibroblas | Tm4sf1 |
| 0 | 1.546501 | 0.886 | 0.331 | 0 | Fibroblas | Pdia3 |
| 0 | 1.544517 | 0.861 | 0.2 | 0 | Fibroblas | App |
| 0 | 1.542733 | 0.913 | 0.33 | 0 | Fibroblas | Ptms |
| 0 | 1.54208 | 0.684 | 0.046 | 0 | Fibroblas | Socs2 |
| 0 | 1.536654 | 0.667 | 0.009 | 0 | Fibroblas | Mmp23 |
| 0 | 1.533409 | 0.587 | 0.01 | 0 | Fibroblas | Fxyd6 |

| | | | | | | |
|---|---|---|---|---|---|---|
| 0 | 1.527816 | 0.705 | 0.013 | 0 | Fibroblas | Fkbp7 |
| 0 | 1.524393 | 0.924 | 0.327 | 0 | Fibroblas | Selenof |
| 0 | 1.521298 | 0.554 | 0.013 | 0 | Fibroblas | Adamts1 |
| 0 | 1.50969 | 0.684 | 0.01 | 0 | Fibroblas | P3h3 |
| 0 | 1.501883 | 0.68 | 0.016 | 0 | Fibroblas | Cavin1 |
| 0 | 1.500308 | 0.581 | 0.008 | 0 | Fibroblas | Scara5 |
| 0 | 1.494355 | 0.55 | 0.011 | 0 | Fibroblas | Gas6 |
| 0 | 1.485883 | 0.468 | 0.01 | 0 | Fibroblas | Pgf |
| 0 | 1.47772 | 0.84 | 0.187 | 0 | Fibroblas | Ddost |
| 0 | 1.470783 | 0.625 | 0.014 | 0 | Fibroblas | Ndrg2 |
| 0 | 1.46604 | 0.808 | 0.139 | 0 | Fibroblas | Arl1 |
| 0 | 1.447906 | 0.571 | 0.008 | 0 | Fibroblas | Frzb |
| 0 | 1.445454 | 0.556 | 0.009 | 0 | Fibroblas | Col14a1 |
| 0 | 1.444861 | 0.574 | 0.008 | 0 | Fibroblas | Loxl2 |
| 0 | 1.443228 | 0.647 | 0.008 | 0 | Fibroblas | Lpar1 |
| 0 | 1.440829 | 0.639 | 0.01 | 0 | Fibroblas | Ndn |
| 0 | 1.434848 | 0.642 | 0.009 | 0 | Fibroblas | Snhg18 |
| 0 | 1.431173 | 0.94 | 0.43 | 0 | Fibroblas | Ppib |
| 0 | 1.429669 | 0.545 | 0.134 | 0 | Fibroblas | Gadd45g |
| 0 | 1.421921 | 0.816 | 0.208 | 0 | Fibroblas | Pdia6 |
| 0 | 1.417932 | 0.623 | 0.106 | 0 | Fibroblas | Gem |
| 0 | 1.417806 | 0.505 | 0.009 | 0 | Fibroblas | Vcam1 |
| 0 | 1.410271 | 0.58 | 0.011 | 0 | Fibroblas | Maged2 |
| 0 | 1.406565 | 0.588 | 0.008 | 0 | Fibroblas | Aldh1a1 |
| 0 | 1.402019 | 0.685 | 0.04 | 0 | Fibroblas | Mfge8 |
| 0 | 1.397301 | 0.654 | 0.018 | 0 | Fibroblas | Eng |
| 0 | 1.395619 | 0.505 | 0.042 | 0 | Fibroblas | Tppp3 |
| 0 | 1.395088 | 0.723 | 0.055 | 0 | Fibroblas | Tspan3 |
| 0 | 1.394742 | 0.643 | 0.012 | 0 | Fibroblas | Npdc1 |
| 0 | 1.394569 | 0.665 | 0.01 | 0 | Fibroblas | Gpx8 |
| 0 | 1.390653 | 0.914 | 0.341 | 0 | Fibroblas | Lamp1 |
| 0 | 1.389308 | 0.558 | 0.008 | 0 | Fibroblas | Cp |
| 0 | 1.387811 | 0.625 | 0.012 | 0 | Fibroblas | Steap3 |
| 0 | 1.385059 | 0.623 | 0.009 | 0 | Fibroblas | Plpp1 |
| 0 | 1.384601 | 0.673 | 0.034 | 0 | Fibroblas | Thra |
| 0 | 1.383178 | 0.684 | 0.042 | 0 | Fibroblas | Rcn1 |
| 0 | 1.379902 | 0.73 | 0.094 | 0 | Fibroblas | Dap |
| 0 | 1.377184 | 0.65 | 0.021 | 0 | Fibroblas | Tpst1 |
| 0 | 1.373765 | 0.608 | 0.01 | 0 | Fibroblas | Ppic |
| 0 | 1.370798 | 0.398 | 0.008 | 0 | Fibroblas | Meox1 |
| 0 | 1.367856 | 0.567 | 0.01 | 0 | Fibroblas | Tbx20 |
| 0 | 1.36566 | 0.588 | 0.006 | 0 | Fibroblas | Matn2 |
| 0 | 1.357587 | 0.618 | 0.013 | 0 | Fibroblas | Colec12 |
| 0 | 1.355696 | 0.601 | 0.007 | 0 | Fibroblas | Kdelr3 |
| 0 | 1.355236 | 0.592 | 0.007 | 0 | Fibroblas | C1ra |
| 0 | 1.350062 | 0.972 | 0.618 | 0 | Fibroblas | Gnas |
| 0 | 1.349395 | 0.642 | 0.009 | 0 | Fibroblas | Sgce |
| 0 | 1.346461 | 0.58 | 0.007 | 0 | Fibroblas | Abi3bp |
| 0 | 1.345447 | 0.619 | 0.009 | 0 | Fibroblas | Bmp1 |
| 0 | 1.344792 | 0.804 | 0.157 | 0 | Fibroblas | Lmna |
| 0 | 1.340968 | 0.664 | 0.035 | 0 | Fibroblas | Cnn3 |
| 0 | 1.335175 | 0.566 | 0.149 | 0 | Fibroblas | Hsd11b1 |
| 0 | 1.332485 | 0.498 | 0.005 | 0 | Fibroblas | Thbs2 |
| 0 | 1.331349 | 0.816 | 0.181 | 0 | Fibroblas | Itm2c |

| | | | | | | |
|---|---|---|---|---|---|---|
| 0 | 1.327512 | 0.515 | 0.005 | 0 | Fibroblas | Ms4a4d |
| 0 | 1.3246 | 0.418 | 0.04 | 0 | Fibroblas | Igfbp6 |
| 0 | 1.318823 | 0.607 | 0.005 | 0 | Fibroblas | Mrc2 |
| 0 | 1.318022 | 0.627 | 0.013 | 0 | Fibroblas | Maged1 |
| 0 | 1.316795 | 0.606 | 0.006 | 0 | Fibroblas | Gpr153 |
| 0 | 1.30713 | 0.706 | 0.069 | 0 | Fibroblas | Comt |
| 0 | 1.300822 | 0.631 | 0.039 | 0 | Fibroblas | Vat1 |
| 0 | 1.294805 | 0.671 | 0.051 | 0 | Fibroblas | Lman1 |
| 0 | 1.290627 | 0.571 | 0.013 | 0 | Fibroblas | Akap12 |
| 0 | 1.286099 | 0.512 | 0.006 | 0 | Fibroblas | Cpxm1 |
| 0 | 1.283153 | 0.737 | 0.132 | 0 | Fibroblas | Kdelr2 |
| 0 | 1.279517 | 0.585 | 0.014 | 0 | Fibroblas | Mmp14 |
| 0 | 1.2764 | 0.39 | 0.009 | 0 | Fibroblas | Serpine1 |
| 0 | 1.275882 | 0.57 | 0.012 | 0 | Fibroblas | Lamb2 |
| 0 | 1.270822 | 0.616 | 0.044 | 0 | Fibroblas | Cyth3 |
| 0 | 1.270229 | 0.568 | 0.022 | 0 | Fibroblas | Ar |
| 0 | 1.263907 | 0.556 | 0.009 | 0 | Fibroblas | Ecm2 |
| 0 | 1.263869 | 0.68 | 0.085 | 0 | Fibroblas | Dpysl2 |
| 0 | 1.251698 | 0.49 | 0.006 | 0 | Fibroblas | Bmp4 |
| 0 | 1.251387 | 0.504 | 0.004 | 0 | Fibroblas | Podn |
| 0 | 1.244585 | 0.555 | 0.023 | 0 | Fibroblas | Atp1a2 |
| 0 | 1.243395 | 0.611 | 0.047 | 0 | Fibroblas | Heg1 |
| 0 | 1.238472 | 0.624 | 0.014 | 0 | Fibroblas | Gng11 |
| 0 | 1.236154 | 0.556 | 0.006 | 0 | Fibroblas | Scn7a |
| 0 | 1.232757 | 0.503 | 0.007 | 0 | Fibroblas | C1s1 |
| 0 | 1.232402 | 0.478 | 0.007 | 0 | Fibroblas | Igf1 |
| 0 | 1.231434 | 0.337 | 0.046 | 0 | Fibroblas | Mbd1 |
| 0 | 1.228588 | 0.616 | 0.05 | 0 | Fibroblas | Clic4 |
| 0 | 1.222543 | 0.579 | 0.014 | 0 | Fibroblas | 8-Sep |
| 0 | 1.219417 | 0.543 | 0.006 | 0 | Fibroblas | C1qtnf7 |
| 0 | 1.218426 | 0.668 | 0.056 | 0 | Fibroblas | Ptov1 |
| 0 | 1.2165 | 0.65 | 0.054 | 0 | Fibroblas | Prdx4 |
| 0 | 1.210715 | 0.524 | 0.181 | 0 | Fibroblas | Gm12840 |
| 0 | 1.210013 | 0.802 | 0.2 | 0 | Fibroblas | Rpn2 |
| 0 | 1.207656 | 0.44 | 0.045 | 0 | Fibroblas | Hk2 |
| 0 | 1.207055 | 0.798 | 0.172 | 0 | Fibroblas | Hmgn1 |
| 0 | 1.206015 | 0.598 | 0.015 | 0 | Fibroblas | Ryk |
| 0 | 1.197432 | 0.539 | 0.013 | 0 | Fibroblas | Phlda3 |
| 0 | 1.191716 | 0.786 | 0.229 | 0 | Fibroblas | Dbi |
| 0 | 1.185744 | 0.914 | 0.366 | 0 | Fibroblas | Prdx1 |
| 0 | 1.182384 | 0.446 | 0.004 | 0 | Fibroblas | Rgs17 |
| 0 | 1.180121 | 0.581 | 0.014 | 0 | Fibroblas | Ehd2 |
| 0 | 1.179228 | 0.555 | 0.009 | 0 | Fibroblas | Sash1 |
| 0 | 1.178483 | 0.53 | 0.028 | 0 | Fibroblas | Vcan |
| 0 | 1.177223 | 0.706 | 0.099 | 0 | Fibroblas | Atraid |
| 0 | 1.172894 | 0.762 | 0.26 | 0 | Fibroblas | Cdkn1a |
| 0 | 1.168426 | 0.562 | 0.005 | 0 | Fibroblas | Naalad2 |
| 0 | 1.161531 | 0.57 | 0.012 | 0 | Fibroblas | Ltbp3 |
| 0 | 1.161399 | 0.782 | 0.176 | 0 | Fibroblas | Oat |
| 0 | 1.158707 | 0.796 | 0.199 | 0 | Fibroblas | Kdelr1 |
| 0 | 1.154112 | 0.576 | 0.019 | 0 | Fibroblas | Crtap |
| 0 | 1.152266 | 0.567 | 0.009 | 0 | Fibroblas | Gpx7 |
| 0 | 1.152055 | 0.514 | 0.035 | 0 | Fibroblas | Sulf2 |
| 0 | 1.149671 | 0.619 | 0.051 | 0 | Fibroblas | Cnpy2 |

| | | | | | | |
|---|---|---|---|---|---|---|
| 0 | 1.145847 | 0.862 | 0.284 | 0 | Fibroblas | Fkbp1a |
| 0 | 1.142201 | 0.56 | 0.016 | 0 | Fibroblas | Tceal8 |
| 0 | 1.141605 | 0.528 | 0.007 | 0 | Fibroblas | Khdrbs3 |
| 0 | 1.134742 | 0.561 | 0.023 | 0 | Fibroblas | Mast4 |
| 0 | 1.129232 | 0.496 | 0.005 | 0 | Fibroblas | Gstm2 |
| 0 | 1.129168 | 0.82 | 0.225 | 0 | Fibroblas | Swi5 |
| 0 | 1.1266 | 0.679 | 0.092 | 0 | Fibroblas | Pea15a |
| 0 | 1.125165 | 0.866 | 0.285 | 0 | Fibroblas | Krtcap2 |
| 0 | 1.120126 | 0.56 | 0.012 | 0 | Fibroblas | Copz2 |
| 0 | 1.116018 | 0.435 | 0.009 | 0 | Fibroblas | Ndufa4l2 |
| 0 | 1.114781 | 0.423 | 0.106 | 0 | Fibroblas | Ly6c1 |
| 0 | 1.1143 | 0.682 | 0.095 | 0 | Fibroblas | Lrpap1 |
| 0 | 1.113896 | 0.686 | 0.136 | 0 | Fibroblas | Pdlim2 |
| 0 | 1.110876 | 0.94 | 0.491 | 0 | Fibroblas | Dynll1 |
| 0 | 1.108039 | 0.453 | 0.009 | 0 | Fibroblas | Cryab |
| 0 | 1.106903 | 0.479 | 0.006 | 0 | Fibroblas | Pdgfrl |
| 0 | 1.106575 | 0.647 | 0.086 | 0 | Fibroblas | Tceal9 |
| 0 | 1.102457 | 0.765 | 0.164 | 0 | Fibroblas | Map1lc3a |
| 0 | 1.100558 | 0.652 | 0.081 | 0 | Fibroblas | Il6st |
| 0 | 1.100442 | 0.424 | 0.005 | 0 | Fibroblas | Efemp1 |
| 0 | 1.100313 | 0.815 | 0.206 | 0 | Fibroblas | Dstn |
| 0 | 1.099326 | 0.572 | 0.034 | 0 | Fibroblas | Ece1 |
| 0 | 1.098376 | 0.733 | 0.145 | 0 | Fibroblas | Ergic3 |
| 0 | 1.093919 | 0.519 | 0.012 | 0 | Fibroblas | Lima1 |
| 0 | 1.092263 | 0.549 | 0.011 | 0 | Fibroblas | Efemp2 |
| 0 | 1.091881 | 0.554 | 0.054 | 0 | Fibroblas | F2r |
| 0 | 1.090672 | 0.535 | 0.005 | 0 | Fibroblas | Tmem45a |
| 0 | 1.090162 | 0.557 | 0.085 | 0 | Fibroblas | Kcnq1ot1 |
| 0 | 1.088483 | 0.509 | 0.007 | 0 | Fibroblas | Fam198b |
| 0 | 1.087311 | 0.758 | 0.174 | 0 | Fibroblas | Prdx2 |
| 0 | 1.086669 | 0.549 | 0.046 | 0 | Fibroblas | Lrrc8a |
| 0 | 1.083143 | 0.555 | 0.049 | 0 | Fibroblas | Tubb6 |
| 0 | 1.081926 | 0.452 | 0.008 | 0 | Fibroblas | Plat |
| 0 | 1.08138 | 0.991 | 0.911 | 0 | Fibroblas | Itm2b |
| 0 | 1.080727 | 0.478 | 0.005 | 0 | Fibroblas | Srpx2 |
| 0 | 1.080634 | 0.881 | 0.534 | 0 | Fibroblas | Hsp90aa1 |
| 0 | 1.079686 | 0.806 | 0.268 | 0 | Fibroblas | Rrbp1 |
| 0 | 1.074027 | 0.607 | 0.045 | 0 | Fibroblas | Gna11 |
| 0 | 1.072953 | 0.655 | 0.177 | 0 | Fibroblas | Glul |
| 0 | 1.069932 | 0.855 | 0.272 | 0 | Fibroblas | Tuba1b |
| 0 | 1.069669 | 0.517 | 0.015 | 0 | Fibroblas | Acvrl1 |
| 0 | 1.068908 | 0.979 | 0.895 | 0 | Fibroblas | Jund |
| 0 | 1.065489 | 0.574 | 0.031 | 0 | Fibroblas | Tnfsf12 |
| 0 | 1.063523 | 0.839 | 0.283 | 0 | Fibroblas | Ahnak |
| 0 | 1.06318 | 0.681 | 0.246 | 0 | Fibroblas | Atf3 |
| 0 | 1.061739 | 0.43 | 0.007 | 0 | Fibroblas | Heyl |
| 0 | 1.059643 | 0.5 | 0.006 | 0 | Fibroblas | Gstt1 |
| 0 | 1.058982 | 0.812 | 0.237 | 0 | Fibroblas | Pebp1 |
| 0 | 1.057326 | 0.544 | 0.017 | 0 | Fibroblas | Clstn1 |
| 0 | 1.0541 | 0.992 | 0.686 | 0 | Fibroblas | mt-Cytb |
| 0 | 1.05291 | 0.526 | 0.017 | 0 | Fibroblas | Lysmd2 |
| 0 | 1.052315 | 0.597 | 0.062 | 0 | Fibroblas | Lrrc58 |
| 0 | 1.047788 | 0.636 | 0.155 | 0 | Fibroblas | Ppp1r14b |
| 0 | 1.044529 | 0.475 | 0.005 | 0 | Fibroblas | Clmp |

| | | | | | | |
|---|---|---|---|---|---|---|
| 0 | 1.04338 | 0.545 | 0.032 | 0 | Fibroblas | Cd151 |
| 0 | 1.040424 | 0.51 | 0.008 | 0 | Fibroblas | Creb3l1 |
| 0 | 1.038844 | 0.511 | 0.007 | 0 | Fibroblas | Arhgef40 |
| 0 | 1.035635 | 0.538 | 0.051 | 0 | Fibroblas | Raph1 |
| 0 | 1.033291 | 0.418 | 0.007 | 0 | Fibroblas | Prss23 |
| 0 | 1.032382 | 0.494 | 0.006 | 0 | Fibroblas | Ghr |
| 0 | 1.032085 | 0.517 | 0.014 | 0 | Fibroblas | Dst |
| 0 | 1.025316 | 0.764 | 0.253 | 0 | Fibroblas | Manf |
| 0 | 1.023468 | 0.579 | 0.046 | 0 | Fibroblas | Fndc3b |
| 0 | 1.022313 | 0.395 | 0.004 | 0 | Fibroblas | Sox9 |
| 0 | 1.017164 | 0.515 | 0.007 | 0 | Fibroblas | Parva |
| 0 | 1.015259 | 0.454 | 0.02 | 0 | Fibroblas | Itih5 |
| 0 | 1.012197 | 0.624 | 0.114 | 0 | Fibroblas | Slc1a5 |
| 0 | 1.012142 | 0.718 | 0.155 | 0 | Fibroblas | Ssr2 |
| 0 | 1.011812 | 0.769 | 0.19 | 0 | Fibroblas | P4hb |
| 0 | 1.010982 | 0.484 | 0.014 | 0 | Fibroblas | Creb3l2 |
| 0 | 1.00959 | 0.876 | 0.385 | 0 | Fibroblas | Arf4 |
| 0 | 1.009584 | 0.474 | 0.016 | 0 | Fibroblas | Cyp27a1 |
| 0 | 1.006788 | 0.497 | 0.029 | 0 | Fibroblas | Nrp1 |
| 0 | 1.00382 | 0.42 | 0.006 | 0 | Fibroblas | Spon1 |
| 0 | 1.002337 | 0.553 | 0.047 | 0 | Fibroblas | Rbpms |
| 0 | -1.01658 | 0.591 | 0.622 | 0 | Fibroblas | Msn |
| 0 | -1.01783 | 0.909 | 0.904 | 0 | Fibroblas | Cfl1 |
| 0 | -1.02813 | 0.974 | 0.969 | 0 | Fibroblas | H3f3b |
| 0 | -1.03097 | 0.066 | 0.278 | 0 | Fibroblas | Gng2 |
| 0 | -1.05025 | 0.018 | 0.273 | 0 | Fibroblas | Ikzf1 |
| 0 | -1.05566 | 0.04 | 0.265 | 0 | Fibroblas | B4galnt1 |
| 0 | -1.05778 | 0.756 | 0.764 | 0 | Fibroblas | Rap1b |
| 0 | -1.09321 | 0.021 | 0.279 | 0 | Fibroblas | Arhgap9 |
| 0 | -1.10657 | 0.022 | 0.284 | 0 | Fibroblas | Tbc1d10c |
| 0 | -1.11386 | 0.02 | 0.278 | 0 | Fibroblas | Pstpip1 |
| 0 | -1.1391 | 0.814 | 0.767 | 0 | Fibroblas | Pkm |
| 0 | -1.13923 | 0.813 | 0.812 | 0 | Fibroblas | Pnrc1 |
| 0 | -1.1482 | 0.99 | 0.993 | 0 | Fibroblas | Fau |
| 0 | -1.15539 | 0.088 | 0.324 | 0 | Fibroblas | Rasgrp2 |
| 0 | -1.16585 | 0.48 | 0.581 | 0 | Fibroblas | Supt4a |
| 0 | -1.16728 | 0.067 | 0.277 | 0 | Fibroblas | Skap2 |
| 0 | -1.16766 | 0.151 | 0.345 | 0 | Fibroblas | Atp11b |
| 0 | -1.18777 | 0.104 | 0.331 | 0 | Fibroblas | Arl4c |
| 0 | -1.19532 | 0.076 | 0.272 | 0 | Fibroblas | Syk |
| 0 | -1.20152 | 0.873 | 0.796 | 0 | Fibroblas | Map1lc3b |
| 0 | -1.22314 | 0.27 | 0.451 | 0 | Fibroblas | Apbb1ip |
| 0 | -1.2283 | 0.038 | 0.281 | 0 | Fibroblas | Retreg1 |
| 0 | -1.23457 | 0.807 | 0.805 | 0 | Fibroblas | Arpc3 |
| 0 | -1.24589 | 0.074 | 0.322 | 0 | Fibroblas | Chd7 |
| 0 | -1.24865 | 0.017 | 0.29 | 0 | Fibroblas | Itgb7 |
| 0 | -1.25147 | 0.072 | 0.296 | 0 | Fibroblas | Rassf3 |
| 0 | -1.25247 | 0.548 | 0.638 | 0 | Fibroblas | Clk1 |
| 0 | -1.2576 | 0.594 | 0.651 | 0 | Fibroblas | H2afj |
| 0 | -1.26164 | 0.029 | 0.331 | 0 | Fibroblas | Arhgap15 |
| 0 | -1.2689 | 0.022 | 0.274 | 0 | Fibroblas | Ptpn22 |
| 0 | -1.27268 | 0.086 | 0.307 | 0 | Fibroblas | 1-Sep |
| 0 | -1.27595 | 0.083 | 0.285 | 0 | Fibroblas | Csf2ra |
| 0 | -1.27888 | 0.024 | 0.344 | 0 | Fibroblas | Arhgap30 |

| | | | | | | |
|---|---|---|---|---|---|---|
| 0 | -1.28199 | 0.586 | 0.598 | 0 | Fibroblas | Sub1 |
| 0 | -1.28575 | 0.33 | 0.506 | 0 | Fibroblas | Ppp1r18 |
| 0 | -1.29291 | 0.022 | 0.252 | 0 | Fibroblas | Themis2 |
| 0 | -1.30132 | 0.021 | 0.258 | 0 | Fibroblas | Nfam1 |
| 0 | -1.30182 | 0.473 | 0.594 | 0 | Fibroblas | Dazap2 |
| 0 | -1.31365 | 0.021 | 0.27 | 0 | Fibroblas | Itgam |
| 0 | -1.31551 | 0.02 | 0.344 | 0 | Fibroblas | Bin2 |
| 0 | -1.32784 | 0.017 | 0.275 | 0 | Fibroblas | Gimap1 |
| 0 | -1.33806 | 0.158 | 0.395 | 0 | Fibroblas | Fmnl1 |
| 0 | -1.35028 | 0.155 | 0.414 | 0 | Fibroblas | Ankrd44 |
| 0 | -1.35194 | 0.572 | 0.674 | 0 | Fibroblas | Actr3 |
| 0 | -1.35723 | 0.351 | 0.49 | 0 | Fibroblas | Ezr |
| 0 | -1.35999 | 0.024 | 0.33 | 0 | Fibroblas | Cd84 |
| 0 | -1.37005 | 0.134 | 0.403 | 0 | Fibroblas | Cd82 |
| 0 | -1.38254 | 0.026 | 0.369 | 0 | Fibroblas | Dock2 |
| 0 | -1.38778 | 0.409 | 0.607 | 0 | Fibroblas | Cnn2 |
| 0 | -1.38836 | 0.027 | 0.34 | 0 | Fibroblas | Inpp5d |
| 0 | -1.38882 | 0.024 | 0.327 | 0 | Fibroblas | Ptpn6 |
| 0 | -1.39065 | 0.611 | 0.668 | 0 | Fibroblas | Cap1 |
| 0 | -1.40436 | 0.067 | 0.33 | 0 | Fibroblas | Lyn |
| 0 | -1.40897 | 0.068 | 0.383 | 0 | Fibroblas | Slc9a3r1 |
| 0 | -1.40965 | 0.024 | 0.276 | 0 | Fibroblas | Cd300lf |
| 0 | -1.41215 | 0.2 | 0.459 | 0 | Fibroblas | Grk2 |
| 0 | -1.41484 | 0.536 | 0.619 | 0 | Fibroblas | Iqgap1 |
| 0 | -1.41698 | 0.027 | 0.341 | 0 | Fibroblas | Myo1f |
| 0 | -1.41943 | 0.779 | 0.845 | 0 | Fibroblas | Arpc2 |
| 0 | -1.44986 | 0.023 | 0.288 | 0 | Fibroblas | Ptprcap |
| 0 | -1.45087 | 0.021 | 0.279 | 0 | Fibroblas | Lck |
| 0 | -1.46184 | 0.022 | 0.279 | 0 | Fibroblas | Trem3 |
| 0 | -1.46199 | 0.023 | 0.261 | 0 | Fibroblas | AC110211.1 |
| 0 | -1.4794 | 0.021 | 0.283 | 0 | Fibroblas | Cd300a |
| 0 | -1.48239 | 0.023 | 0.278 | 0 | Fibroblas | Pilra |
| 0 | -1.48523 | 0.525 | 0.649 | 0 | Fibroblas | Ostf1 |
| 0 | -1.48729 | 0.053 | 0.278 | 0 | Fibroblas | Entpd1 |
| 0 | -1.49091 | 0.114 | 0.404 | 0 | Fibroblas | Vsir |
| 0 | -1.49577 | 0.029 | 0.261 | 0 | Fibroblas | Ankrd33b |
| 0 | -1.49924 | 0.969 | 0.983 | 0 | Fibroblas | Actg1 |
| 0 | -1.50426 | 0.976 | 0.989 | 0 | Fibroblas | H2-D1 |
| 0 | -1.50876 | 0.641 | 0.734 | 0 | Fibroblas | Arpc5 |
| 0 | -1.51875 | 0.212 | 0.484 | 0 | Fibroblas | Lrrfip1 |
| 0 | -1.52048 | 0.669 | 0.773 | 0 | Fibroblas | Sh3bgrl3 |
| 0 | -1.52416 | 0.99 | 0.995 | 0 | Fibroblas | Actb |
| 0 | -1.53501 | 0.119 | 0.358 | 0 | Fibroblas | Tnfaip3 |
| 0 | -1.53681 | 0.074 | 0.359 | 0 | Fibroblas | Dusp5 |
| 0 | -1.5387 | 0.329 | 0.515 | 0 | Fibroblas | Rhog |
| 0 | -1.542 | 0.946 | 0.958 | 0 | Fibroblas | H3f3a |
| 0 | -1.54632 | 0.041 | 0.324 | 0 | Fibroblas | 2310001H17Rik |
| 0 | -1.55277 | 0.016 | 0.269 | 0 | Fibroblas | Bcl2a1b |
| 0 | -1.56588 | 0.025 | 0.268 | 0 | Fibroblas | Cd93 |
| 0 | -1.56854 | 0.412 | 0.556 | 0 | Fibroblas | Adgre5 |
| 0 | -1.57485 | 0.027 | 0.311 | 0 | Fibroblas | Pira2 |
| 0 | -1.57632 | 0.031 | 0.357 | 0 | Fibroblas | Glipr1 |
| 0 | -1.58406 | 0.026 | 0.379 | 0 | Fibroblas | AB124611 |
| 0 | -1.58598 | 0.021 | 0.3 | 0 | Fibroblas | Cd2 |

| | | | | | | |
|---|---|---|---|---|---|---|
| 0 | -1.59592 | 0.251 | 0.47 | 0 | Fibroblas | Efhd2 |
| 0 | -1.5978 | 0.08 | 0.315 | 0 | Fibroblas | Satb1 |
| 0 | -1.60268 | 0.517 | 0.591 | 0 | Fibroblas | Zyx |
| 0 | -1.6072 | 0.182 | 0.471 | 0 | Fibroblas | Man2b1 |
| 0 | -1.61126 | 0.147 | 0.41 | 0 | Fibroblas | Dennd4a |
| 0 | -1.61481 | 0.022 | 0.321 | 0 | Fibroblas | Ms4a6b |
| 0 | -1.62028 | 0.549 | 0.625 | 0 | Fibroblas | Prr13 |
| 0 | -1.62259 | 0.052 | 0.318 | 0 | Fibroblas | Slc15a3 |
| 0 | -1.63437 | 0.025 | 0.265 | 0 | Fibroblas | Ptafr |
| 0 | -1.64965 | 0.022 | 0.273 | 0 | Fibroblas | Il1rn |
| 0 | -1.65261 | 0.026 | 0.315 | 0 | Fibroblas | Slpi |
| 0 | -1.66198 | 0.037 | 0.297 | 0 | Fibroblas | Gimap6 |
| 0 | -1.66834 | 0.021 | 0.304 | 0 | Fibroblas | Gm5150 |
| 0 | -1.67732 | 0.022 | 0.27 | 0 | Fibroblas | Cd300ld |
| 0 | -1.6841 | 0.029 | 0.29 | 0 | Fibroblas | Gcnt2 |
| 0 | -1.69567 | 0.023 | 0.294 | 0 | Fibroblas | Arg2 |
| 0 | -1.69725 | 0.535 | 0.604 | 0 | Fibroblas | Cdk2ap2 |
| 0 | -1.70341 | 0.035 | 0.409 | 0 | Fibroblas | Itgal |
| 0 | -1.71201 | 0.042 | 0.451 | 0 | Fibroblas | Fyb |
| 0 | -1.71501 | 0.797 | 0.823 | 0 | Fibroblas | Cyba |
| 0 | -1.71602 | 0.044 | 0.3 | 0 | Fibroblas | Sh2d3c |
| 0 | -1.72784 | 0.349 | 0.61 | 0 | Fibroblas | Gpsm3 |
| 0 | -1.74699 | 0.216 | 0.4 | 0 | Fibroblas | Ets1 |
| 0 | -1.76798 | 0.062 | 0.335 | 0 | Fibroblas | Pygl |
| 0 | -1.76899 | 0.303 | 0.599 | 0 | Fibroblas | Vasp |
| 0 | -1.77535 | 0.648 | 0.776 | 0 | Fibroblas | Lsp1 |
| 0 | -1.77641 | 0.029 | 0.335 | 0 | Fibroblas | Ncf1 |
| 0 | -1.80353 | 0.216 | 0.484 | 0 | Fibroblas | Glrx |
| 0 | -1.82136 | 0.029 | 0.335 | 0 | Fibroblas | Cd33 |
| 0 | -1.83807 | 0.213 | 0.555 | 0 | Fibroblas | Samhd1 |
| 0 | -1.84196 | 0.03 | 0.399 | 0 | Fibroblas | Snx20 |
| 0 | -1.85055 | 0.122 | 0.559 | 0 | Fibroblas | Fam49b |
| 0 | -1.85545 | 0.026 | 0.368 | 0 | Fibroblas | Jaml |
| 0 | -1.86517 | 0.039 | 0.356 | 0 | Fibroblas | Cd24a |
| 0 | -1.87325 | 0.315 | 0.682 | 0 | Fibroblas | Ucp2 |
| 0 | -1.87914 | 0.053 | 0.538 | 0 | Fibroblas | Arhgap45 |
| 0 | -1.89123 | 0.036 | 0.495 | 0 | Fibroblas | Cd37 |
| 0 | -1.89602 | 0.897 | 0.963 | 0 | Fibroblas | Pfn1 |
| 0 | -1.89729 | 0.036 | 0.504 | 0 | Fibroblas | Hcls1 |
| 0 | -1.91436 | 0.02 | 0.252 | 0 | Fibroblas | Rgs1 |
| 0 | -1.93725 | 0.03 | 0.359 | 0 | Fibroblas | Slfn1 |
| 0 | -1.95148 | 0.081 | 0.535 | 0 | Fibroblas | Fam107b |
| 0 | -1.95934 | 0.445 | 0.678 | 0 | Fibroblas | Pim1 |
| 0 | -1.96198 | 0.029 | 0.316 | 0 | Fibroblas | Slc7a11 |
| 0 | -2.01825 | 0.545 | 0.729 | 0 | Fibroblas | Zfp36l2 |
| 0 | -2.02815 | 0.725 | 0.824 | 0 | Fibroblas | Mcl1 |
| 0 | -2.04703 | 0.13 | 0.362 | 0 | Fibroblas | Stx11 |
| 0 | -2.05198 | 0.042 | 0.406 | 0 | Fibroblas | Plek |
| 0 | -2.05557 | 0.032 | 0.369 | 0 | Fibroblas | Pla2g7 |
| 0 | -2.0667 | 0.843 | 0.748 | 0 | Fibroblas | S100a11 |
| 0 | -2.0701 | 0.042 | 0.534 | 0 | Fibroblas | Itgb2 |
| 0 | -2.07329 | 0.102 | 0.439 | 0 | Fibroblas | Tpd52 |
| 0 | -2.0812 | 0.038 | 0.526 | 0 | Fibroblas | Ptpn18 |
| 0 | -2.12637 | 0.033 | 0.365 | 0 | Fibroblas | Dusp2 |

| | | | | | | |
|---|---|---|---|---|---|---|
| 0 | -2.1283 | 0.038 | 0.501 | 0 | Fibroblas | Ncf4 |
| 0 | -2.13781 | 0.044 | 0.402 | 0 | Fibroblas | Adam8 |
| 0 | -2.14753 | 0.033 | 0.432 | 0 | Fibroblas | Cd3d |
| 0 | -2.16498 | 0.054 | 0.468 | 0 | Fibroblas | Sorl1 |
| 0 | -2.19567 | 0.032 | 0.424 | 0 | Fibroblas | Fcgr3 |
| 0 | -2.21789 | 0.04 | 0.536 | 0 | Fibroblas | Tspan13 |
| 0 | -2.2275 | 0.043 | 0.429 | 0 | Fibroblas | Lilr4b |
| 0 | -2.24134 | 0.04 | 0.506 | 0 | Fibroblas | Sell |
| 0 | -2.24972 | 0.194 | 0.461 | 0 | Fibroblas | Ets2 |
| 0 | -2.25107 | 0.032 | 0.336 | 0 | Fibroblas | Rdh12 |
| 0 | -2.25422 | 0.045 | 0.495 | 0 | Fibroblas | Cxcr4 |
| 0 | -2.2551 | 0.026 | 0.29 | 0 | Fibroblas | Cd3g |
| 0 | -2.27306 | 0.037 | 0.384 | 0 | Fibroblas | Cxcr2 |
| 0 | -2.2971 | 0.607 | 0.694 | 0 | Fibroblas | Taldo1 |
| 0 | -2.29777 | 0.209 | 0.477 | 0 | Fibroblas | Rnf149 |
| 0 | -2.29921 | 0.036 | 0.411 | 0 | Fibroblas | Cyp4f18 |
| 0 | -2.30133 | 0.026 | 0.262 | 0 | Fibroblas | Acod1 |
| 0 | -2.30565 | 0.022 | 0.32 | 0 | Fibroblas | Ms4a4b |
| 0 | -2.30654 | 0.086 | 0.516 | 0 | Fibroblas | Neurl3 |
| 0 | -2.31033 | 0.094 | 0.635 | 0 | Fibroblas | Limd2 |
| 0 | -2.32578 | 0.072 | 0.541 | 0 | Fibroblas | Rgs2 |
| 0 | -2.32658 | 0.041 | 0.599 | 0 | Fibroblas | Hcst |
| 0 | -2.33297 | 0.035 | 0.398 | 0 | Fibroblas | Mcemp1 |
| 0 | -2.33984 | 0.028 | 0.29 | 0 | Fibroblas | Hcar2 |
| 0 | -2.34369 | 0.268 | 0.659 | 0 | Fibroblas | Hmgb2 |
| 0 | -2.34968 | 0.022 | 0.3 | 0 | Fibroblas | Trbc2 |
| 0 | -2.37487 | 0.039 | 0.547 | 0 | Fibroblas | Ltb |
| 0 | -2.38361 | 0.113 | 0.536 | 0 | Fibroblas | Cd44 |
| 0 | -2.41118 | 0.035 | 0.416 | 0 | Fibroblas | C5ar1 |
| 0 | -2.41853 | 0.053 | 0.618 | 0 | Fibroblas | Emb |
| 0 | -2.46246 | 0.038 | 0.466 | 0 | Fibroblas | Spi1 |
| 0 | -2.49743 | 0.667 | 0.878 | 0 | Fibroblas | Fxyd5 |
| 0 | -2.50458 | 0.324 | 0.785 | 0 | Fibroblas | Cotl1 |
| 0 | -2.5198 | 0.874 | 0.804 | 0 | Fibroblas | Cebpb |
| 0 | -2.54281 | 0.05 | 0.576 | 0 | Fibroblas | Slfn2 |
| 0 | -2.627 | 0.039 | 0.446 | 0 | Fibroblas | Ccr1 |
| 0 | -2.63142 | 0.04 | 0.398 | 0 | Fibroblas | Clec4e |
| 0 | -2.64416 | 0.044 | 0.546 | 0 | Fibroblas | Samsn1 |
| 0 | -2.6454 | 0.038 | 0.409 | 0 | Fibroblas | Trem1 |
| 0 | -2.65665 | 0.056 | 0.742 | 0 | Fibroblas | Cd53 |
| 0 | -2.65993 | 0.148 | 0.482 | 0 | Fibroblas | Plaur |
| 0 | -2.68702 | 0.097 | 0.526 | 0 | Fibroblas | Lgals3 |
| 0 | -2.71317 | 0.047 | 0.489 | 0 | Fibroblas | Ncf2 |
| 0 | -2.72417 | 0.033 | 0.338 | 0 | Fibroblas | Mmp8 |
| 0 | -2.7257 | 0.052 | 0.728 | 0 | Fibroblas | Gmfg |
| 0 | -2.7631 | 0.061 | 0.773 | 0 | Fibroblas | Laptm5 |
| 0 | -2.77453 | 0.054 | 0.722 | 0 | Fibroblas | Cytip |
| 0 | -2.8239 | 0.196 | 0.832 | 0 | Fibroblas | Arhgdib |
| 0 | -2.83753 | 0.081 | 0.733 | 0 | Fibroblas | Selplg |
| 0 | -2.84707 | 0.077 | 0.5 | 0 | Fibroblas | Lmnb1 |
| 0 | -2.85299 | 0.988 | 1 | 0 | Fibroblas | Tmsb4x |
| 0 | -2.88347 | 0.043 | 0.442 | 0 | Fibroblas | Clec4d |
| 0 | -2.90978 | 0.041 | 0.409 | 0 | Fibroblas | Mmp9 |
| 0 | -2.9162 | 0.132 | 0.582 | 0 | Fibroblas | Vps37b |

| | | | | | | |
|---|---|---|---|---|---|---|
| 0 | -2.91658 | 0.145 | 0.454 | 0 | Fibroblas | Slc16a3 |
| 0 | -2.92085 | 0.035 | 0.358 | 0 | Fibroblas | Wfdc21 |
| 0 | -2.95804 | 0.064 | 0.78 | 0 | Fibroblas | Ptprc |
| 0 | -2.98074 | 0.064 | 0.326 | 0 | Fibroblas | Cd14 |
| 0 | -2.98119 | 0.145 | 0.788 | 0 | Fibroblas | Stk17b |
| 0 | -2.99869 | 0.06 | 0.52 | 0 | Fibroblas | Pglyrp1 |
| 0 | -3.04776 | 0.047 | 0.461 | 0 | Fibroblas | Csf3r |
| 0 | -3.05736 | 0.095 | 0.5 | 0 | Fibroblas | Lst1 |
| 0 | -3.1012 | 0.041 | 0.296 | 0 | Fibroblas | Lcn2 |
| 0 | -3.15752 | 0.026 | 0.26 | 0 | Fibroblas | Ccl4 |
| 0 | -3.20593 | 0.054 | 0.533 | 0 | Fibroblas | Alox5ap |
| 0 | -3.2229 | 0.06 | 0.739 | 0 | Fibroblas | Lcp1 |
| 0 | -3.25481 | 0.05 | 0.465 | 0 | Fibroblas | Hp |
| 0 | -3.35006 | 0.048 | 0.446 | 0 | Fibroblas | Hdc |
| 0 | -3.35451 | 0.135 | 0.397 | 0 | Fibroblas | Ccrl2 |
| 0 | -3.37071 | 0.131 | 0.603 | 0 | Fibroblas | Mxd1 |
| 0 | -3.40126 | 0.569 | 0.955 | 0 | Fibroblas | Btg1 |
| 0 | -3.41154 | 0.073 | 0.875 | 0 | Fibroblas | Rac2 |
| 0 | -3.44219 | 0.071 | 0.88 | 0 | Fibroblas | Coro1a |
| 0 | -3.53499 | 0.072 | 0.618 | 0 | Fibroblas | Fcer1g |
| 0 | -3.59092 | 0.057 | 0.498 | 0 | Fibroblas | Ccl6 |
| 0 | -3.66291 | 0.058 | 0.453 | 0 | Fibroblas | Il1r2 |
| 0 | -3.67188 | 0.353 | 0.592 | 0 | Fibroblas | Msrb1 |
| 0 | -3.81998 | 0.066 | 0.38 | 0 | Fibroblas | Cxcl2 |
| 0 | -3.95078 | 0.066 | 0.468 | 0 | Fibroblas | Il1b |
| 0 | -4.13445 | 0.081 | 0.926 | 0 | Fibroblas | Cd52 |
| 0 | -4.33378 | 0.082 | 0.645 | 0 | Fibroblas | Tyrobp |
| 0 | -4.6103 | 0.104 | 0.525 | 0 | Fibroblas | Lyz2 |
| 0 | -4.73159 | 0.171 | 0.967 | 0 | Fibroblas | Srgn |
| 0 | -4.93352 | 0.156 | 0.509 | 0 | Fibroblas | Retnlg |
| 0 | -5.29074 | 0.366 | 0.646 | 0 | Fibroblas | S100a9 |
| 0 | -5.34894 | 0.31 | 0.633 | 0 | Fibroblas | S100a8 |
| ######## | -1.0893 | 0.077 | 0.254 | ######## | Fibroblas | H2-Q7 |
| ######## | -1.34842 | 0.124 | 0.301 | ######## | Fibroblas | Ogfrl1 |
| ######## | -1.23396 | 0.147 | 0.324 | ######## | Fibroblas | Tnfrsf1b |
| ######## | -1.40194 | 0.459 | 0.536 | ######## | Fibroblas | Adipor1 |
| ######## | -1.67641 | 0.115 | 0.292 | ######## | Fibroblas | Marcksl1 |
| ######## | -1.00974 | 0.105 | 0.279 | ######## | Fibroblas | D16Ertd472e |
| ######## | -1.62779 | 0.217 | 0.381 | ######## | Fibroblas | Ninj1 |
| ######## | -1.52081 | 0.235 | 0.392 | ######## | Fibroblas | Emilin2 |
| ######## | -1.27429 | 0.828 | 0.723 | ######## | Fibroblas | Ctsd |
| ######## | -1.03871 | 0.987 | 0.951 | ######## | Fibroblas | Rps27 |
| ######## | -1.26807 | 0.592 | 0.596 | ######## | Fibroblas | Mrpl33 |
| ######## | -1.09993 | 0.369 | 0.485 | ######## | Fibroblas | Ptbp3 |
| ######## | -1.07004 | 0.091 | 0.257 | ######## | Fibroblas | Abtb1 |
| ######## | -1.83805 | 0.674 | 0.609 | ######## | Fibroblas | Litaf |
| ######## | -1.36763 | 0.287 | 0.425 | ######## | Fibroblas | 7-Mar |
| ######## | -1.15174 | 0.343 | 0.463 | ######## | Fibroblas | Myh9 |
| ######## | -1.71436 | 0.202 | 0.37 | ######## | Fibroblas | Tgfbi |
| ######## | -1.22458 | 0.151 | 0.315 | ######## | Fibroblas | Gpcpd1 |
| ######## | -1.09469 | 0.584 | 0.635 | ######## | Fibroblas | Ier5 |
| ######## | -1.08789 | 0.091 | 0.252 | ######## | Fibroblas | Hopx |
| ######## | -1.48716 | 0.179 | 0.338 | ######## | Fibroblas | Plk3 |
| ######## | -1.15856 | 0.165 | 0.326 | ######## | Fibroblas | Itpkb |

| | | | | | | |
|---|---|---|---|---|---|---|
| ######## | -2.00328 | 0.12 | 0.277 | ######## | Fibroblas | Basp1 |
| ######## | -1.14651 | 0.731 | 0.652 | ######## | Fibroblas | H2afz |
| ######## | -1.52188 | 0.3 | 0.419 | ######## | Fibroblas | Nudt4 |
| ######## | -1.18089 | 0.591 | 0.579 | ######## | Fibroblas | Prdx6 |
| ######## | -1.15211 | 0.149 | 0.304 | ######## | Fibroblas | Sephs2 |
| ######## | -2.06677 | 0.571 | 0.555 | ######## | Fibroblas | Grina |
| ######## | -1.58338 | 0.762 | 0.681 | ######## | Fibroblas | H2-K1 |
| ######## | -1.55267 | 0.675 | 0.609 | ######## | Fibroblas | Prdx5 |
| ######## | -1.17531 | 0.183 | 0.331 | ######## | Fibroblas | Crlf3 |
| ######## | -1.51819 | 0.322 | 0.436 | ######## | Fibroblas | 2810474O19Rik |
| ######## | -1.09111 | 0.996 | 0.982 | ######## | Fibroblas | Ftl1 |
| ######## | -1.13097 | 0.126 | 0.272 | ######## | Fibroblas | Lyst |
| ######## | 2.721554 | 0.663 | 0.675 | ######## | Fibroblas | Hbb-bs |
| ######## | -1.30951 | 0.469 | 0.512 | ######## | Fibroblas | Klf3 |
| ######## | -1.10492 | 0.38 | 0.459 | ######## | Fibroblas | Ptpn1 |
| ######## | -1.12798 | 0.381 | 0.465 | ######## | Fibroblas | Psmb8 |
| ######## | -1.05149 | 0.331 | 0.427 | ######## | Fibroblas | Grb2 |
| ######## | -1.12663 | 0.231 | 0.347 | ######## | Fibroblas | Prkcd |
| ######## | -1.17743 | 0.234 | 0.347 | ######## | Fibroblas | Pgd |
| ######## | -1.32699 | 0.23 | 0.347 | ######## | Fibroblas | Kctd12 |
| ######## | -1.12652 | 0.429 | 0.484 | ######## | Fibroblas | Fosl2 |
| ######## | -1.33249 | 0.475 | 0.495 | ######## | Fibroblas | Picalm |
| ######## | -1.05712 | 0.157 | 0.28 | ######## | Fibroblas | Rilpl2 |
| ######## | -1.39523 | 0.313 | 0.396 | ######## | Fibroblas | Snap23 |
| ######## | -1.9174 | 0.21 | 0.329 | ######## | Fibroblas | Isg15 |
| ######## | -1.12533 | 0.168 | 0.279 | ######## | Fibroblas | Dgat1 |
| ######## | -1.01776 | 0.442 | 0.467 | ######## | Fibroblas | Eno1 |
| ######## | -1.44359 | 0.277 | 0.356 | ######## | Fibroblas | Tmcc1 |
| ######## | -1.11835 | 0.435 | 0.458 | ######## | Fibroblas | Fam32a |
| ######## | -1.24866 | 0.16 | 0.259 | ######## | Fibroblas | Cpne2 |
| ######## | -1.27519 | 0.222 | 0.302 | ######## | Fibroblas | Fbxl5 |
| ######## | -1.0206 | 0.193 | 0.283 | 2.73E-96 | Fibroblas | Hist1h1c |
| ######## | -1.02157 | 0.849 | 0.694 | 1.16E-95 | Fibroblas | Txn1 |
| 4.30E-99 | -1.21089 | 0.366 | 0.41 | 7.75E-95 | Fibroblas | Trib1 |
| 2.94E-97 | -1.16905 | 0.357 | 0.389 | 5.30E-93 | Fibroblas | Card19 |
| 4.31E-92 | -1.02625 | 0.312 | 0.366 | 7.77E-88 | Fibroblas | Gm26532 |
| 1.19E-88 | -1.02331 | 0.386 | 0.403 | 2.15E-84 | Fibroblas | Atg3 |
| 2.44E-87 | -1.00743 | 0.486 | 0.468 | 4.40E-83 | Fibroblas | Cox17 |
| 6.21E-82 | -1.08869 | 0.491 | 0.463 | 1.12E-77 | Fibroblas | Atp2b1 |
| 7.54E-75 | -1.12408 | 0.49 | 0.448 | 1.36E-70 | Fibroblas | Ehd1 |
| 1.23E-72 | 2.700321 | 0.499 | 0.502 | 2.21E-68 | Fibroblas | Hba-a1 |
| 2.19E-63 | -1.62809 | 0.203 | 0.273 | 3.94E-59 | Fibroblas | Ifitm1 |
| 1.70E-45 | -1.07477 | 0.516 | 0.443 | 3.07E-41 | Fibroblas | Ndel1 |
| 1.23E-39 | -1.3361 | 0.563 | 0.335 | 2.21E-35 | Fibroblas | AW112010 |
| 2.83E-15 | -1.03891 | 0.671 | 0.505 | 5.10E-11 | Fibroblas | Uba52 |
| 4.73E-12 | -1.2601 | 0.766 | 0.614 | 8.51E-08 | Fibroblas | Nfkbia |
| 2.08E-06 | -1.05842 | 0.945 | 0.585 | 0.037393 | Fibroblas | Tmsb10 |
| 0 | 6.700779 | 0.996 | 0.363 | 0 | Neutrophi | S100a9 |
| 0 | 6.634196 | 0.997 | 0.318 | 0 | Neutrophi | S100a8 |
| 0 | 6.261991 | 0.931 | 0.157 | 0 | Neutrophi | Retnlg |
| 0 | 4.741311 | 0.931 | 0.058 | 0 | Neutrophi | Il1r2 |
| 0 | 4.506433 | 0.909 | 0.079 | 0 | Neutrophi | Il1b |
| 0 | 4.459004 | 0.997 | 0.32 | 0 | Neutrophi | Msrb1 |
| 0 | 4.323117 | 0.702 | 0.082 | 0 | Neutrophi | Cxcl2 |

| | | | | | |
|---|---|---|---|---|---|
| 0 | 4.320239 | 0.744 | 0.126 | 0 | NeutrophiCcrl2 |
| 0 | 4.16906 | 0.601 | 0.042 | 0 | NeutrophiLcn2 |
| 0 | 4.130272 | 0.905 | 0.068 | 0 | NeutrophiHp |
| 0 | 4.080699 | 0.943 | 0.052 | 0 | NeutrophiCsf3r |
| 0 | 4.060252 | 0.933 | 0.046 | 0 | NeutrophiHdc |
| 0 | 4.031788 | 0.962 | 0.191 | 0 | NeutrophiMxd1 |
| 0 | 3.996888 | 0.999 | 0.174 | 0 | NeutrophiTyrobp |
| 0 | 3.994772 | 0.881 | 0.033 | 0 | NeutrophiMmp9 |
| 0 | 3.902983 | 0.886 | 0.129 | 0 | NeutrophiSlc16a3 |
| 0 | 3.881032 | 0.733 | 0.039 | 0 | NeutrophiWfdc21 |
| 0 | 3.846859 | 0.896 | 0.051 | 0 | NeutrophiClec4d |
| 0 | 3.753147 | 0.389 | 0.019 | 0 | NeutrophiGm5483 |
| 0 | 3.726112 | 0.9 | 0.094 | 0 | NeutrophiCcl6 |
| 0 | 3.703324 | 0.914 | 0.104 | 0 | NeutrophiLmnb1 |
| 0 | 3.692749 | 0.677 | 0.042 | 0 | NeutrophiMmp8 |
| 0 | 3.631827 | 0.886 | 0.114 | 0 | NeutrophiPglyrp1 |
| 0 | 3.626454 | 0.47 | 0.024 | 0 | NeutrophiAsprv1 |
| 0 | 3.61754 | 0.838 | 0.044 | 0 | NeutrophiTrem1 |
| 0 | 3.594053 | 0.913 | 0.116 | 0 | NeutrophiLst1 |
| 0 | 3.447878 | 0.317 | 0.017 | 0 | NeutrophiBC100530 |
| 0 | 3.446827 | 0.772 | 0.057 | 0 | NeutrophiClec4e |
| 0 | 3.444012 | 0.997 | 0.434 | 0 | NeutrophiSrgn |
| 0 | 3.439129 | 0.868 | 0.059 | 0 | NeutrophiCcr1 |
| 0 | 3.434774 | 0.588 | 0.08 | 0 | NeutrophiCd14 |
| 0 | 3.412294 | 0.883 | 0.087 | 0 | NeutrophiNcf2 |
| 0 | 3.381675 | 0.998 | 0.795 | 0 | NeutrophiCebpb |
| 0 | 3.343582 | 0.963 | 0.096 | 0 | NeutrophiAlox5ap |
| 0 | 3.299537 | 0.604 | 0.029 | 0 | NeutrophiHcar2 |
| 0 | 3.275318 | 0.825 | 0.031 | 0 | NeutrophiCxcr2 |
| 0 | 3.270239 | 0.254 | 0.014 | 0 | NeutrophiNgp |
| 0 | 3.233205 | 0.827 | 0.049 | 0 | NeutrophiC5ar1 |
| 0 | 3.228056 | 0.797 | 0.046 | 0 | NeutrophiMcemp1 |
| 0 | 3.207092 | 0.702 | 0.032 | 0 | NeutrophiRdh12 |
| 0 | 3.154955 | 0.934 | 0.459 | 0 | NeutrophiGrina |
| 0 | 3.148833 | 0.797 | 0.174 | 0 | NeutrophiPlaur |
| 0 | 3.121863 | 0.372 | 0.013 | 0 | NeutrophiStfa2l1 |
| 0 | 3.100237 | 0.882 | 0.147 | 0 | NeutrophiLyz2 |
| 0 | 3.099237 | 0.841 | 0.418 | 0 | NeutrophiG0s2 |
| 0 | 3.080824 | 0.557 | 0.023 | 0 | NeutrophiAcod1 |
| 0 | 3.08039 | 0.84 | 0.078 | 0 | NeutrophiSpi1 |
| 0 | 3.074925 | 0.938 | 0.236 | 0 | NeutrophiLcp1 |
| 0 | 3.069408 | 0.819 | 0.184 | 0 | NeutrophiEts2 |
| 0 | 3.052166 | 0.809 | 0.207 | 0 | NeutrophiRnf149 |
| 0 | 3.001 | 0.953 | 0.564 | 0 | NeutrophiTaldo1 |
| 0 | 2.99981 | 0.988 | 0.743 | 0 | NeutrophiS100a11 |
| 0 | 2.98499 | 0.775 | 0.061 | 0 | NeutrophiCyp4f18 |
| 0 | 2.982117 | 0.556 | 0.094 | 0 | NeutrophiBasp1 |
| 0 | 2.933558 | 0.971 | 0.159 | 0 | NeutrophiFcer1g |
| 0 | 2.899048 | 0.667 | 0.123 | 0 | NeutrophiStx11 |
| 0 | 2.886766 | 0.672 | 0.026 | 0 | NeutrophiSlc7a11 |
| 0 | 2.74599 | 0.534 | 0.193 | 0 | NeutrophiIsg15 |
| 0 | 2.744339 | 0.939 | 0.559 | 0 | NeutrophiLitaf |
| 0 | 2.700754 | 0.736 | 0.072 | 0 | NeutrophiAdam8 |
| 0 | 2.687486 | 0.692 | 0.056 | 0 | NeutrophiPla2g7 |

| | | | | | |
|---|---|---|---|---|---|
| 0 | 2.659686 | 0.753 | 0.115 | 0 | NeutrophiSorl1 |
| 0 | 2.641471 | 0.676 | 0.038 | 0 | NeutrophiCd33 |
| 0 | 2.624053 | 0.748 | 0.13 | 0 | NeutrophiTpd52 |
| 0 | 2.613893 | 0.675 | 0.059 | 0 | NeutrophiPygl |
| 0 | 2.600966 | 0.643 | 0.062 | 0 | NeutrophiSlfn1 |
| 0 | 2.551409 | 0.618 | 0.024 | 0 | NeutrophiArg2 |
| 0 | 2.541154 | 0.572 | 0.022 | 0 | NeutrophiCd300ld |
| 0 | 2.53391 | 0.507 | 0.161 | 0 | NeutrophiIfitm1 |
| 0 | 2.518114 | 0.592 | 0.049 | 0 | NeutrophiSh2d3c |
| 0 | 2.509524 | 0.795 | 0.184 | 0 | NeutrophiCd44 |
| 0 | 2.505116 | 0.945 | 0.725 | 0 | NeutrophiMcl1 |
| 0 | 2.502788 | 0.742 | 0.076 | 0 | NeutrophiFcgr3 |
| 0 | 2.491418 | 0.774 | 0.151 | 0 | NeutrophiSamsn1 |
| 0 | 2.491376 | 0.721 | 0.092 | 0 | NeutrophiLilr4b |
| 0 | 2.484397 | 0.744 | 0.182 | 0 | NeutrophiSlfn2 |
| 0 | 2.450125 | 0.544 | 0.028 | 0 | NeutrophiPtafr |
| 0 | 2.445022 | 0.575 | 0.038 | 0 | NeutrophiGcnt2 |
| 0 | 2.440467 | 0.648 | 0.065 | 0 | NeutrophiCd24a |
| 0 | 2.428194 | 0.395 | 0.014 | 0 | NeutrophiLy6g |
| 0 | 2.423938 | 0.618 | 0.053 | 0 | NeutrophiNcf1 |
| 0 | 2.421854 | 0.971 | 0.713 | 0 | NeutrophiFxyd5 |
| 0 | 2.415019 | 0.751 | 0.13 | 0 | NeutrophiSell |
| 0 | 2.411064 | 0.905 | 0.332 | 0 | NeutrophiStk17b |
| 0 | 2.348378 | 0.627 | 0.07 | 0 | NeutrophiJaml |
| 0 | 2.343661 | 0.989 | 0.691 | 0 | NeutrophiBtg1 |
| 0 | 2.338196 | 0.74 | 0.152 | 0 | NeutrophiTspan13 |
| 0 | 2.334896 | 0.601 | 0.063 | 0 | NeutrophiSlc15a3 |
| 0 | 2.327571 | 0.487 | 0.12 | 0 | NeutrophiMarcksl1 |
| 0 | 2.3258 | 0.724 | 0.255 | 0 | NeutrophiNudt4 |
| 0 | 2.318508 | 0.632 | 0.202 | 0 | NeutrophiNinj1 |
| 0 | 2.316585 | 0.552 | 0.026 | 0 | NeutrophiAnkrd33b |
| 0 | 2.314683 | 0.642 | 0.223 | 0 | NeutrophiTmcc1 |
| 0 | 2.310183 | 0.855 | 0.468 | 0 | NeutrophiZyx |
| 0 | 2.300575 | 0.717 | 0.135 | 0 | NeutrophiNcf4 |
| 0 | 2.294068 | 0.561 | 0.02 | 0 | NeutrophiAC110211.1 |
| 0 | 2.290709 | 0.496 | 0.017 | 0 | NeutrophiFpr1 |
| 0 | 2.286833 | 0.584 | 0.286 | 0 | NeutrophiTnfaip2 |
| 0 | 2.286405 | 0.768 | 0.28 | 0 | NeutrophiTrib1 |
| 0 | 2.281161 | 0.489 | 0.019 | 0 | NeutrophiF630028O10Rik |
| 0 | 2.260094 | 0.529 | 0.036 | 0 | NeutrophiIl1rn |
| 0 | 2.25238 | 0.618 | 0.106 | 0 | NeutrophiPlek |
| 0 | 2.244399 | 0.504 | 0.042 | 0 | NeutrophiCd93 |
| 0 | 2.240642 | 0.701 | 0.255 | 0 | NeutrophiSnap23 |
| 0 | 2.23752 | 0.564 | 0.029 | 0 | NeutrophiPilra |
| 0 | 2.236546 | 0.978 | 0.355 | 0 | NeutrophiCd52 |
| 0 | 2.235461 | 0.572 | 0.027 | 0 | NeutrophiTrem3 |
| 0 | 2.233603 | 0.835 | 0.352 | 0 | NeutrophiHmgb2 |
| 0 | 2.217427 | 0.836 | 0.588 | 0 | NeutrophiPrdx5 |
| 0 | 2.19364 | 0.526 | 0.06 | 0 | NeutrophiEntpd1 |
| 0 | 2.190823 | 0.856 | 0.247 | 0 | NeutrophiGmfg |
| 0 | 2.181256 | 0.556 | 0.047 | 0 | NeutrophiGm5150 |
| 0 | 2.169782 | 0.576 | 0.106 | 0 | NeutrophiSnx20 |
| 0 | 2.164266 | 0.593 | 0.102 | 0 | NeutrophiOgfrl1 |
| 0 | 2.156898 | 0.34 | 0.021 | 0 | NeutrophiLrg1 |

| | | | | | | |
|---|---|---|---|---|---|---|
| 0 | 2.134193 | 0.564 | 0.052 | 0 | Neutrophi | Pira2 |
| 0 | 2.129758 | 0.525 | 0.118 | 0 | Neutrophi | Cpne2 |
| 0 | 2.128137 | 0.567 | 0.069 | 0 | Neutrophi | 2310001H17Rik |
| 0 | 2.112875 | 0.683 | 0.192 | 0 | Neutrophi | Rgs2 |
| 0 | 2.105692 | 0.628 | 0.307 | 0 | Neutrophi | 2810474O19Rik |
| 0 | 2.103909 | 0.931 | 0.333 | 0 | Neutrophi | Coro1a |
| 0 | 2.101892 | 0.687 | 0.205 | 0 | Neutrophi | Emilin2 |
| 0 | 2.09195 | 0.787 | 0.414 | 0 | Neutrophi | Adipor1 |
| 0 | 2.090106 | 0.536 | 0.039 | 0 | Neutrophi | Cd300a |
| 0 | 2.089395 | 0.615 | 0.165 | 0 | Neutrophi | Cxcr4 |
| 0 | 2.076643 | 1 | 0.992 | 0 | Neutrophi | Tmsb4x |
| 0 | 2.069745 | 0.423 | 0.042 | 0 | Neutrophi | Osm |
| 0 | 2.066279 | 0.86 | 0.268 | 0 | Neutrophi | Selplg |
| 0 | 2.050706 | 0.358 | 0.01 | 0 | Neutrophi | Mrgpra2b |
| 0 | 2.037734 | 0.516 | 0.042 | 0 | Neutrophi | Cd300lf |
| 0 | 2.033344 | 0.45 | 0.02 | 0 | Neutrophi | Chil1 |
| 0 | 2.032397 | 0.802 | 0.49 | 0 | Neutrophi | Pim1 |
| 0 | 2.026867 | 0.671 | 0.539 | 0 | Neutrophi | Cdk2ap2 |
| 0 | 2.024039 | 0.913 | 0.653 | 0 | Neutrophi | Lsp1 |
| 0 | 2.01401 | 0.512 | 0.19 | 0 | Neutrophi | Fbxl5 |
| 0 | 2.00907 | 0.512 | 0.027 | 0 | Neutrophi | Themis2 |
| 0 | 2.003979 | 0.535 | 0.178 | 0 | Neutrophi | Plk3 |
| 0 | 2.000681 | 0.769 | 0.534 | 0 | Neutrophi | Prr13 |
| 0 | 1.996082 | 0.561 | 0.085 | 0 | Neutrophi | Glipr1 |
| 0 | 1.994022 | 0.777 | 0.391 | 0 | Neutrophi | Gpsm3 |
| 0 | 1.970568 | 0.69 | 0.25 | 0 | Neutrophi | Glrx |
| 0 | 1.966605 | 0.707 | 0.338 | 0 | Neutrophi | Rhog |
| 0 | 1.964844 | 0.906 | 0.516 | 0 | Neutrophi | Cd9 |
| 0 | 1.958638 | 0.935 | 0.807 | 0 | Neutrophi | Map1lc3b |
| 0 | 1.955519 | 0.998 | 0.995 | 0 | Neutrophi | Fth1 |
| 0 | 1.955203 | 0.573 | 0.284 | 0 | Neutrophi | Jdp2 |
| 0 | 1.954349 | 0.718 | 0.414 | 0 | Neutrophi | Ndel1 |
| 0 | 1.942471 | 0.422 | 0.045 | 0 | Neutrophi | Tlr2 |
| 0 | 1.940311 | 0.898 | 0.739 | 0 | Neutrophi | Txn1 |
| 0 | 1.938693 | 0.492 | 0.036 | 0 | Neutrophi | Nfam1 |
| 0 | 1.92912 | 0.717 | 0.419 | 0 | Neutrophi | Picalm |
| 0 | 1.923835 | 0.551 | 0.213 | 0 | Neutrophi | Kctd12 |
| 0 | 1.915737 | 0.575 | 0.051 | 0 | Neutrophi | Slpi |
| 0 | 1.891078 | 0.398 | 0.026 | 0 | Neutrophi | Nlrp3 |
| 0 | 1.883962 | 0.916 | 0.336 | 0 | Neutrophi | Rac2 |
| 0 | 1.874557 | 0.832 | 0.294 | 0 | Neutrophi | Ptprc |
| 0 | 1.871514 | 0.897 | 0.743 | 0 | Neutrophi | Ctsd |
| 0 | 1.851032 | 1 | 0.986 | 0 | Neutrophi | Ftl1 |
| 0 | 1.819539 | 0.422 | 0.023 | 0 | Neutrophi | Fpr2 |
| 0 | 1.812348 | 0.571 | 0.21 | 0 | Neutrophi | Pgd |
| 0 | 1.811237 | 0.753 | 0.208 | 0 | Neutrophi | Emb |
| 0 | 1.810776 | 0.703 | 0.43 | 0 | Neutrophi | Klf3 |
| 0 | 1.806697 | 0.552 | 0.097 | 0 | Neutrophi | AB124611 |
| 0 | 1.805756 | 0.998 | 0.969 | 0 | Neutrophi | Actg1 |
| 0 | 1.802709 | 0.356 | 0.012 | 0 | Neutrophi | Ptgs2os2 |
| 0 | 1.796272 | 0.429 | 0.056 | 0 | Neutrophi | Slc2a3 |
| 0 | 1.792711 | 0.5 | 0.091 | 0 | Neutrophi | Csf2ra |
| 0 | 1.787418 | 0.578 | 0.29 | 0 | Neutrophi | 7-Mar |
| 0 | 1.7848 | 0.419 | 0.089 | 0 | Neutrophi | Rab20 |

| | | | | | |
|---|---|---|---|---|---|
| 0 | 1.783859 | 0.385 | 0.089 | 0 | NeutrophiDusp16 |
| 0 | 1.778226 | 0.625 | 0.161 | 0 | NeutrophiHcls1 |
| 0 | 1.774181 | 0.488 | 0.115 | 0 | NeutrophiLyst |
| 0 | 1.773834 | 0.68 | 0.41 | 0 | NeutrophiEhd1 |
| 0 | 1.772273 | 0.412 | 0.015 | 0 | NeutrophiDhrs9 |
| 0 | 1.767838 | 0.663 | 0.174 | 0 | NeutrophiItgb2 |
| 0 | 1.765643 | 0.579 | 0.314 | 0 | NeutrophiCard19 |
| 0 | 1.763612 | 0.85 | 0.463 | 0 | NeutrophiCotl1 |
| 0 | 1.763167 | 0.441 | 0.032 | 0 | NeutrophiLpcat2 |
| 0 | 1.759436 | 0.278 | 0.041 | 0 | NeutrophiIfitm6 |
| 0 | 1.754142 | 0.377 | 0.037 | 0 | NeutrophiSlc40a1 |
| 0 | 1.748512 | 0.677 | 0.296 | 0 | NeutrophiSamhd1 |
| 0 | 1.748094 | 0.452 | 0.041 | 0 | NeutrophiFgr |
| 0 | 1.745808 | 0.44 | 0.104 | 0 | NeutrophiRabgef1 |
| 0 | 1.742606 | 0.931 | 0.775 | 0 | NeutrophiCyba |
| 0 | 1.739175 | 0.565 | 0.168 | 0 | NeutrophiVsir |
| 0 | 1.73425 | 0.605 | 0.243 | 0 | NeutrophiMan2b1 |
| 0 | 1.733581 | 0.66 | 0.386 | 0 | NeutrophiFam32a |
| 0 | 1.730755 | 0.75 | 0.528 | 0 | NeutrophiIqgap1 |
| 0 | 1.713521 | 0.759 | 0.6 | 0 | NeutrophiRab7 |
| 0 | 1.710324 | 0.532 | 0.203 | 0 | NeutrophiDennd4a |
| 0 | 1.709777 | 0.699 | 0.376 | 0 | NeutrophiVasp |
| 0 | 1.704322 | 0.492 | 0.149 | 0 | NeutrophiSephs2 |
| 0 | 1.703355 | 0.652 | 0.194 | 0 | NeutrophiNeurl3 |
| 0 | 1.700404 | 0.514 | 0.085 | 0 | NeutrophiMyo1f |
| 0 | 1.691825 | 0.605 | 0.288 | 0 | NeutrophiEfhd2 |
| 0 | 1.683851 | 0.484 | 0.159 | 0 | NeutrophiGpcpd1 |
| 0 | 1.67366 | 0.461 | 0.09 | 0 | NeutrophiAbtb1 |
| 0 | 1.669097 | 0.426 | 0.033 | 0 | NeutrophiHck |
| 0 | 1.663112 | 0.408 | 0.043 | 0 | NeutrophiActa2 |
| 0 | 1.661538 | 0.768 | 0.545 | 0 | NeutrophiMrpl33 |
| 0 | 1.661242 | 0.782 | 0.529 | 0 | NeutrophiOstf1 |
| 0 | 1.651516 | 0.802 | 0.593 | 0 | NeutrophiCap1 |
| 0 | 1.651181 | 0.506 | 0.107 | 0 | NeutrophiLyn |
| 0 | 1.649459 | 0.434 | 0.045 | 0 | NeutrophiPirb |
| 0 | 1.644257 | 0.894 | 0.675 | 0 | NeutrophiNeat1 |
| 0 | 1.640784 | 0.833 | 0.413 | 0 | NeutrophiArhgdib |
| 0 | 1.639006 | 0.411 | 0.055 | 0 | NeutrophiRin3 |
| 0 | 1.63866 | 0.419 | 0.086 | 0 | NeutrophiSlc27a4 |
| 0 | 1.636442 | 0.598 | 0.336 | 0 | NeutrophiAtg3 |
| 0 | 1.634965 | 0.42 | 0.049 | 0 | NeutrophiHacd4 |
| 0 | 1.63399 | 0.462 | 0.087 | 0 | NeutrophiSkap2 |
| 0 | 1.632825 | 0.457 | 0.103 | 0 | NeutrophiRassf3 |
| 0 | 1.630393 | 0.758 | 0.272 | 0 | NeutrophiCytip |
| 0 | 1.617709 | 0.774 | 0.281 | 0 | NeutrophiCd53 |
| 0 | 1.613249 | 0.387 | 0.04 | 0 | NeutrophiLrrc25 |
| 0 | 1.611093 | 0.387 | 0.036 | 0 | NeutrophiRab11fip1 |
| 0 | 1.60335 | 0.633 | 0.433 | 0 | NeutrophiDhrs7 |
| 0 | 1.600569 | 0.363 | 0.014 | 0 | NeutrophiTrpm2 |
| 0 | 1.598435 | 0.375 | 0.021 | 0 | NeutrophiCd300lb |
| 0 | 1.592505 | 0.512 | 0.201 | 0 | NeutrophiSirpa |
| 0 | 1.584981 | 0.412 | 0.105 | 0 | NeutrophiFem1c |
| 0 | 1.57819 | 0.59 | 0.413 | 0 | NeutrophiR3hdm4 |
| 0 | 1.573841 | 0.435 | 0.059 | 0 | NeutrophiItgam |

| | | | | | |
|---|---|---|---|---|---|
| 0 | 1.569291 | 0.443 | 0.095 | 0 | NeutrophiSyk |
| 0 | 1.569044 | 0.531 | 0.2 | 0 | NeutrophiFmnl1 |
| 0 | 1.5683 | 0.984 | 0.943 | 0 | NeutrophiH3f3a |
| 0 | 1.560017 | 0.315 | 0.025 | 0 | NeutrophiCd177 |
| 0 | 1.558255 | 0.788 | 0.637 | 0 | NeutrophiSdcbp |
| 0 | 1.548206 | 0.715 | 0.55 | 0 | NeutrophiPtp4a1 |
| 0 | 1.545441 | 0.427 | 0.164 | 0 | NeutrophiDgat1 |
| 0 | 1.539779 | 0.422 | 0.049 | 0 | NeutrophiDok3 |
| 0 | 1.536782 | 0.42 | 0.095 | 0 | NeutrophiCfap43 |
| 0 | 1.520741 | 0.534 | 0.264 | 0 | NeutrophiGda |
| 0 | 1.516456 | 0.454 | 0.092 | 0 | NeutrophiPtpn6 |
| 0 | 1.514048 | 0.504 | 0.136 | 0 | NeutrophiItgal |
| 0 | 1.50152 | 0.512 | 0.315 | 0 | NeutrophiKpna4 |
| 0 | 1.492836 | 0.816 | 0.65 | 0 | NeutrophiArpc5 |
| 0 | 1.477905 | 0.296 | 0.009 | 0 | NeutrophiIl1f9 |
| 0 | 1.471874 | 0.307 | 0.061 | 0 | NeutrophiOsgin1 |
| 0 | 1.471268 | 0.303 | 0.02 | 0 | NeutrophiCd300c2 |
| 0 | 1.466003 | 0.323 | 0.031 | 0 | NeutrophiMirt1 |
| 0 | 1.460676 | 0.357 | 0.045 | 0 | NeutrophiSt3gal5 |
| 0 | 1.455177 | 0.834 | 0.674 | 0 | NeutrophiYpel3 |
| 0 | 1.449589 | 0.336 | 0.046 | 0 | NeutrophiCsf2rb |
| 0 | 1.44553 | 0.339 | 0.022 | 0 | NeutrophiSlc2a6 |
| 0 | 1.443796 | 0.998 | 0.991 | 0 | NeutrophiActb |
| 0 | 1.442841 | 0.424 | 0.179 | 0 | NeutrophiTnfrsf1b |
| 0 | 1.436888 | 0.481 | 0.231 | 0 | NeutrophiCcpg1 |
| 0 | 1.430331 | 0.473 | 0.262 | 0 | NeutrophiAntxr2 |
| 0 | 1.429879 | 0.557 | 0.285 | 0 | NeutrophiLrrfip1 |
| 0 | 1.417777 | 0.368 | 0.038 | 0 | NeutrophiClec4a2 |
| 0 | 1.409424 | 0.69 | 0.488 | 0 | NeutrophiDazap2 |
| 0 | 1.401437 | 0.651 | 0.49 | 0 | NeutrophiTnfrsf1a |
| 0 | 1.395075 | 0.859 | 0.791 | 0 | NeutrophiArpc3 |
| 0 | 1.391711 | 0.289 | 0.028 | 0 | NeutrophiMpzl3 |
| 0 | 1.386552 | 0.96 | 0.837 | 0 | NeutrophiS100a6 |
| 0 | 1.381219 | 0.53 | 0.268 | 0 | NeutrophiGrk2 |
| 0 | 1.375838 | 0.632 | 0.28 | 0 | NeutrophiLimd2 |
| 0 | 1.374406 | 0.45 | 0.176 | 0 | NeutrophiActn1 |
| 0 | 1.371734 | 0.382 | 0.093 | 0 | NeutrophiRetreg1 |
| 0 | 1.367727 | 0.328 | 0.02 | 0 | NeutrophiRasgrp4 |
| 0 | 1.361998 | 0.745 | 0.571 | 0 | NeutrophiIer5 |
| 0 | 1.351048 | 0.494 | 0.229 | 0 | NeutrophiPrkcd |
| 0 | 1.346475 | 0.273 | 0.016 | 0 | Neutrophi1600010M07Rik |
| 0 | 1.344931 | 0.297 | 0.015 | 0 | NeutrophiFcgr4 |
| 0 | 1.342828 | 0.338 | 0.083 | 0 | NeutrophiAdrb2 |
| 0 | 1.331603 | 0.476 | 0.276 | 0 | NeutrophiZcchc6 |
| 0 | 1.32955 | 0.256 | 0.013 | 0 | Neutrophi4833407H14Rik |
| 0 | 1.328975 | 0.299 | 0.014 | 0 | NeutrophiA530064D06Rik |
| 0 | 1.325792 | 0.548 | 0.36 | 0 | NeutrophiMyh9 |
| 0 | 1.324889 | 0.408 | 0.135 | 0 | NeutrophiChd7 |
| 0 | 1.324486 | 0.615 | 0.411 | 0 | NeutrophiFosl2 |
| 0 | 1.321838 | 0.409 | 0.106 | 0 | NeutrophiCd84 |
| 0 | 1.317287 | 0.324 | 0.089 | 0 | NeutrophiSgms2 |
| 0 | 1.316326 | 0.91 | 0.868 | 0 | NeutrophiGnai2 |
| 0 | 1.312819 | 0.282 | 0.008 | 0 | NeutrophiCeacam10 |
| 0 | 1.301758 | 0.289 | 0.01 | 0 | NeutrophiKlra17 |

| | | | | | |
|---|---|---|---|---|---|
| 0 | 1.296013 | 0.59 | 0.201 | 0 | NeutrophiLtb |
| 0 | 1.288084 | 0.862 | 0.727 | 0 | NeutrophiIfitm2 |
| 0 | 1.28637 | 0.838 | 0.778 | 0 | NeutrophiPkm |
| 0 | 1.282873 | 0.592 | 0.262 | 0 | NeutrophiFam49b |
| 0 | 1.277248 | 0.653 | 0.208 | 0 | NeutrophiLgals3 |
| 0 | 1.276243 | 0.289 | 0.014 | 0 | NeutrophiPilrb2 |
| 0 | 1.275953 | 0.25 | 0.011 | 0 | NeutrophiSlfn4 |
| 0 | 1.261843 | 0.301 | 0.052 | 0 | NeutrophiDck |
| 0 | 1.261597 | 0.728 | 0.592 | 0 | NeutrophiH2afj |
| 0 | 1.257301 | 0.281 | 0.017 | 0 | NeutrophiTlr13 |
| 0 | 1.256328 | 0.291 | 0.028 | 0 | NeutrophiIgsf6 |
| 0 | 1.253336 | 0.522 | 0.337 | 0 | NeutrophiGrb2 |
| 0 | 1.251062 | 0.667 | 0.46 | 0 | NeutrophiCnn2 |
| 0 | 1.246994 | 0.378 | 0.142 | 0 | NeutrophiSlc6a6 |
| 0 | 1.246624 | 0.953 | 0.788 | 0 | NeutrophiDusp1 |
| 0 | 1.243756 | 0.581 | 0.369 | 0 | NeutrophiPpp1r18 |
| 0 | 1.243211 | 0.729 | 0.654 | 0 | NeutrophiAtp6v1g1 |
| 0 | 1.235746 | 0.43 | 0.193 | 0 | NeutrophiAtp11b |
| 0 | 1.233754 | 0.282 | 0.016 | 0 | NeutrophiNfe2 |
| 0 | 1.230342 | 0.302 | 0.102 | 0 | NeutrophiSlc22a15 |
| 0 | 1.228992 | 0.286 | 0.014 | 0 | NeutrophiPadi4 |
| 0 | 1.22679 | 0.7 | 0.561 | 0 | NeutrophiClk1 |
| 0 | 1.225364 | 0.42 | 0.166 | 0 | NeutrophiSlc9a3r1 |
| 0 | 1.224734 | 0.935 | 0.856 | 0 | NeutrophiGabarap |
| 0 | 1.213486 | 0.27 | 0.062 | 0 | NeutrophiCcl3 |
| 0 | 1.213451 | 0.751 | 0.664 | 0 | NeutrophiUbe2b |
| 0 | 1.197885 | 0.734 | 0.316 | 0 | NeutrophiLaptm5 |
| 0 | 1.195034 | 0.363 | 0.143 | 0 | NeutrophiPnkp |
| 0 | 1.193876 | 0.307 | 0.053 | 0 | NeutrophiMir142hg |
| 0 | 1.191094 | 0.257 | 0.008 | 0 | Neutrophi9830107B12Rik |
| 0 | 1.178984 | 0.278 | 0.035 | 0 | NeutrophiSirpb1b |
| 0 | 1.174676 | 0.308 | 0.079 | 0 | NeutrophiMsra |
| 0 | 1.173512 | 0.458 | 0.231 | 0 | NeutrophiAnkrd44 |
| 0 | 1.167092 | 0.977 | 0.915 | 0 | NeutrophiPfn1 |
| 0 | 1.165174 | 0.719 | 0.594 | 0 | NeutrophiActr3 |
| 0 | 1.156208 | 0.34 | 0.11 | 0 | NeutrophiUnc119 |
| 0 | 1.156137 | 0.477 | 0.205 | 0 | NeutrophiCd82 |
| 0 | 1.154806 | 0.848 | 0.799 | 0 | NeutrophiMyl12b |
| 0 | 1.15013 | 0.25 | 0.067 | 0 | NeutrophiAcsl1 |
| 0 | 1.147172 | 0.251 | 0.031 | 0 | NeutrophiDgat2 |
| 0 | 1.131336 | 0.463 | 0.178 | 0 | NeutrophiFyb |
| 0 | 1.126716 | 0.277 | 0.054 | 0 | NeutrophiCybb |
| 0 | 1.126244 | 0.297 | 0.093 | 0 | NeutrophiRnf144a |
| 0 | 1.125291 | 0.252 | 0.016 | 0 | NeutrophiLilra6 |
| 0 | 1.12222 | 0.77 | 0.664 | 0 | NeutrophiGpx1 |
| 0 | 1.118977 | 0.316 | 0.101 | 0 | NeutrophiFes |
| 0 | 1.116089 | 0.861 | 0.822 | 0 | NeutrophiGng5 |
| 0 | 1.102342 | 0.608 | 0.229 | 0 | NeutrophiHcst |
| 0 | 1.10223 | 0.262 | 0.012 | 0 | NeutrophiAlox5 |
| 0 | 1.092289 | 0.865 | 0.796 | 0 | NeutrophiArpc2 |
| 0 | 1.089892 | 0.281 | 0.082 | 0 | NeutrophiAtg2a |
| 0 | 1.086258 | 0.366 | 0.127 | 0 | NeutrophiInpp5d |
| 0 | 1.079788 | 0.296 | 0.076 | 0 | NeutrophiPtpre |
| 0 | 1.072945 | 0.334 | 0.092 | 0 | NeutrophiPstpip1 |

| | | | | | |
|---|---|---|---|---|---|
| 0 | 1.067772 | 0.252 | 0.014 | 0 | NeutrophiMctp1 |
| 0 | 1.059818 | 0.29 | 0.062 | 0 | NeutrophiSema4d |
| 0 | 1.05809 | 0.848 | 0.742 | 0 | NeutrophiSat1 |
| 0 | 1.051294 | 0.255 | 0.023 | 0 | NeutrophiTarm1 |
| 0 | 1.050443 | 0.606 | 0.193 | 0 | NeutrophiTgfbi |
| 0 | 1.038948 | 0.27 | 0.047 | 0 | NeutrophiPlbd1 |
| 0 | 1.023086 | 0.323 | 0.097 | 0 | NeutrophiArhgap9 |
| 0 | 1.020478 | 0.87 | 0.725 | 0 | NeutrophiAnxa2 |
| 0 | 1.005996 | 0.305 | 0.092 | 0 | NeutrophiMap4k2 |
| 0 | 1.001766 | 0.3 | 0.087 | 0 | NeutrophiSema4a |
| 0 | -1.0013 | 0.042 | 0.517 | 0 | NeutrophiPhb2 |
| 0 | -1.00332 | 0.003 | 0.33 | 0 | NeutrophiThbs2 |
| 0 | -1.00346 | 0.004 | 0.395 | 0 | NeutrophiTubb6 |
| 0 | -1.00706 | 0.066 | 0.556 | 0 | NeutrophiCct7 |
| 0 | -1.00726 | 0.161 | 0.68 | 0 | NeutrophiSrsf3 |
| 0 | -1.00842 | 0.004 | 0.383 | 0 | NeutrophiAkap12 |
| 0 | -1.00862 | 0.013 | 0.455 | 0 | NeutrophiPolr2m |
| 0 | -1.00886 | 0.018 | 0.477 | 0 | NeutrophiTomm5 |
| 0 | -1.00908 | 0.009 | 0.437 | 0 | NeutrophiCnpy2 |
| 0 | -1.0095 | 0.015 | 0.465 | 0 | NeutrophiTcp1 |
| 0 | -1.01038 | 0.004 | 0.365 | 0 | NeutrophiVcan |
| 0 | -1.01073 | 0.112 | 0.617 | 0 | NeutrophiPolr1d |
| 0 | -1.01084 | 0.035 | 0.479 | 0 | NeutrophiDek |
| 0 | -1.01176 | 0.003 | 0.392 | 0 | NeutrophiRbpms |
| 0 | -1.01257 | 0.037 | 0.51 | 0 | NeutrophiPsma4 |
| 0 | -1.01293 | 0.007 | 0.412 | 0 | NeutrophiBmp1 |
| 0 | -1.01524 | 0.045 | 0.54 | 0 | NeutrophiEmc7 |
| 0 | -1.01596 | 0.005 | 0.399 | 0 | NeutrophiKdelr3 |
| 0 | -1.01722 | 0.163 | 0.692 | 0 | NeutrophiSmdt1 |
| 0 | -1.01741 | 0.022 | 0.466 | 0 | NeutrophiFkbp3 |
| 0 | -1.01847 | 0.022 | 0.443 | 0 | NeutrophiBola2 |
| 0 | -1.01985 | 0.042 | 0.526 | 0 | NeutrophiSsb |
| 0 | -1.01993 | 0.113 | 0.624 | 0 | NeutrophiArl6ip5 |
| 0 | -1.02025 | 0.033 | 0.495 | 0 | NeutrophiRomo1 |
| 0 | -1.02139 | 0.018 | 0.379 | 0 | NeutrophiAblim1 |
| 0 | -1.02277 | 0.011 | 0.395 | 0 | NeutrophiTmem64 |
| 0 | -1.02333 | 0.004 | 0.393 | 0 | NeutrophiC1ra |
| 0 | -1.02414 | 0.003 | 0.385 | 0 | NeutrophiAbi3bp |
| 0 | -1.02416 | 0.033 | 0.475 | 0 | NeutrophiAtp1a1 |
| 0 | -1.02492 | 0.011 | 0.458 | 0 | NeutrophiTmem109 |
| 0 | -1.02558 | 0.013 | 0.458 | 0 | NeutrophiAkr1b3 |
| 0 | -1.0263 | 0.044 | 0.473 | 0 | NeutrophiHnrnpa1 |
| 0 | -1.02828 | 0.006 | 0.397 | 0 | NeutrophiF2r |
| 0 | -1.02908 | 0.005 | 0.427 | 0 | NeutrophiSgce |
| 0 | -1.03349 | 0.221 | 0.749 | 0 | NeutrophiAtp5c1 |
| 0 | -1.03363 | 0.002 | 0.393 | 0 | NeutrophiMmp14 |
| 0 | -1.03453 | 0.003 | 0.387 | 0 | NeutrophiAr |
| 0 | -1.03462 | 0.003 | 0.42 | 0 | NeutrophiMaged1 |
| 0 | -1.03601 | 0.04 | 0.526 | 0 | NeutrophiCct8 |
| 0 | -1.03627 | 0.241 | 0.775 | 0 | NeutrophiCox7a2l |
| 0 | -1.03644 | 0.005 | 0.39 | 0 | NeutrophiMatn2 |
| 0 | -1.03823 | 0.169 | 0.721 | 0 | NeutrophiNdufa6 |
| 0 | -1.03972 | 0.172 | 0.703 | 0 | NeutrophiPsma7 |
| 0 | -1.04069 | 0.005 | 0.378 | 0 | NeutrophiTbx20 |

| | | | | | |
|---|---|---|---|---|---|
| 0 | -1.0412 | 0.005 | 0.265 | 0 | NeutrophiMeox1 |
| 0 | -1.04217 | 0.013 | 0.458 | 0 | NeutrophiFkbp2 |
| 0 | -1.04551 | 0.025 | 0.49 | 0 | NeutrophiPsmd7 |
| 0 | -1.0457 | 0.211 | 0.735 | 0 | NeutrophiSec62 |
| 0 | -1.04584 | 0.059 | 0.55 | 0 | NeutrophiCanx |
| 0 | -1.05024 | 0.026 | 0.488 | 0 | NeutrophiImpdh2 |
| 0 | -1.05044 | 0.857 | 0.961 | 0 | NeutrophiJund |
| 0 | -1.05066 | 0.025 | 0.505 | 0 | NeutrophiDdrgk1 |
| 0 | -1.0517 | 0.004 | 0.413 | 0 | NeutrophiColec12 |
| 0 | -1.05445 | 0.006 | 0.39 | 0 | NeutrophiAldh1a1 |
| 0 | -1.05459 | 0.181 | 0.647 | 0 | NeutrophiRrbp1 |
| 0 | -1.05619 | 0.091 | 0.624 | 0 | NeutrophiRnf187 |
| 0 | -1.05836 | 0.004 | 0.414 | 0 | NeutrophiPlpp1 |
| 0 | -1.05908 | 0.03 | 0.532 | 0 | NeutrophiPdcd5 |
| 0 | -1.06037 | 0.014 | 0.447 | 0 | NeutrophiP4ha1 |
| 0 | -1.06068 | 0.092 | 0.626 | 0 | NeutrophiSpcs2 |
| 0 | -1.06162 | 0.104 | 0.522 | 0 | NeutrophiH2-Q4 |
| 0 | -1.06407 | 0.017 | 0.483 | 0 | NeutrophiBcl7c |
| 0 | -1.06696 | 0.016 | 0.313 | 0 | NeutrophiHk2 |
| 0 | -1.06704 | 0.017 | 0.325 | 0 | NeutrophiBcl2 |
| 0 | -1.06956 | 0.485 | 0.834 | 0 | NeutrophiDnaja1 |
| 0 | -1.06977 | 0.003 | 0.371 | 0 | NeutrophiCp |
| 0 | -1.07252 | 0.01 | 0.466 | 0 | NeutrophiMrpl23 |
| 0 | -1.07417 | 0.466 | 0.903 | 0 | NeutrophiChchd2 |
| 0 | -1.07536 | 0.017 | 0.478 | 0 | NeutrophiHexb |
| 0 | -1.07569 | 0.476 | 0.915 | 0 | NeutrophiCox4i1 |
| 0 | -1.07653 | 0.003 | 0.406 | 0 | NeutrophiPpic |
| 0 | -1.07729 | 0.004 | 0.43 | 0 | NeutrophiNpdc1 |
| 0 | -1.07983 | 0.099 | 0.514 | 0 | NeutrophiGlul |
| 0 | -1.08059 | 0.004 | 0.443 | 0 | NeutrophiGpx8 |
| 0 | -1.08152 | 0.003 | 0.418 | 0 | NeutrophiSteap3 |
| 0 | -1.08495 | 0.005 | 0.337 | 0 | NeutrophiVcam1 |
| 0 | -1.08561 | 0.085 | 0.606 | 0 | NeutrophiCope |
| 0 | -1.08582 | 0.163 | 0.684 | 0 | NeutrophiNcl |
| 0 | -1.08695 | 0.011 | 0.485 | 0 | NeutrophiFundc2 |
| 0 | -1.087 | 0.058 | 0.558 | 0 | NeutrophiSelenos |
| 0 | -1.08826 | 0.009 | 0.431 | 0 | NeutrophiCyth3 |
| 0 | -1.08972 | 0.099 | 0.633 | 0 | NeutrophiEif2s2 |
| 0 | -1.09552 | 0.005 | 0.43 | 0 | NeutrophiLpar1 |
| 0 | -1.09769 | 0.005 | 0.439 | 0 | NeutrophiTpst1 |
| 0 | -1.09898 | 0.003 | 0.388 | 0 | NeutrophiMaged2 |
| 0 | -1.10065 | 0.005 | 0.425 | 0 | NeutrophiNdn |
| 0 | -1.10142 | 0.027 | 0.439 | 0 | NeutrophiBst2 |
| 0 | -1.10184 | 0.009 | 0.434 | 0 | NeutrophiClic4 |
| 0 | -1.10268 | 0.004 | 0.418 | 0 | NeutrophiGng11 |
| 0 | -1.10612 | 0.005 | 0.427 | 0 | NeutrophiSnhg18 |
| 0 | -1.10721 | 0.032 | 0.517 | 0 | NeutrophiAtraid |
| 0 | -1.11061 | 0.003 | 0.38 | 0 | NeutrophiFrzb |
| 0 | -1.11081 | 0.079 | 0.6 | 0 | NeutrophiOstc |
| 0 | -1.11245 | 0.018 | 0.475 | 0 | NeutrophiTceal9 |
| 0 | -1.11588 | 0.003 | 0.382 | 0 | NeutrophiLoxl2 |
| 0 | -1.11593 | 0.004 | 0.371 | 0 | NeutrophiCol14a1 |
| 0 | -1.11598 | 0.243 | 0.748 | 0 | NeutrophiArf4 |
| 0 | -1.1191 | 0.094 | 0.637 | 0 | NeutrophiSpcs1 |

| | | | | | |
|---|---|---|---|---|---|
| 0 | -1.11944 | 0.005 | 0.461 | 0 | NeutrophiPrdx4 |
| 0 | -1.12001 | 0.012 | 0.436 | 0 | NeutrophiNrip1 |
| 0 | -1.12643 | 0.027 | 0.521 | 0 | NeutrophiHdgf |
| 0 | -1.1271 | 0.011 | 0.428 | 0 | NeutrophiHeg1 |
| 0 | -1.12728 | 0.052 | 0.558 | 0 | NeutrophiErgic3 |
| 0 | -1.12873 | 0.005 | 0.439 | 0 | NeutrophiVat1 |
| 0 | -1.1299 | 0.141 | 0.7 | 0 | Neutrophi2010107E04Rik |
| 0 | -1.13125 | 0.038 | 0.533 | 0 | NeutrophiNop10 |
| 0 | -1.13232 | 0.013 | 0.461 | 0 | NeutrophiThra |
| 0 | -1.13494 | 0.024 | 0.522 | 0 | NeutrophiTimm13 |
| 0 | -1.1354 | 0.007 | 0.482 | 0 | NeutrophiCapn2 |
| 0 | -1.13959 | 0.007 | 0.385 | 0 | NeutrophiScara5 |
| 0 | -1.13965 | 0.033 | 0.521 | 0 | NeutrophiTbrg1 |
| 0 | -1.14448 | 0.017 | 0.504 | 0 | NeutrophiLrpap1 |
| 0 | -1.14591 | 0.018 | 0.3 | 0 | NeutrophiTnfaip6 |
| 0 | -1.14611 | 0.129 | 0.655 | 0 | NeutrophiS100a13 |
| 0 | -1.14742 | 0.014 | 0.414 | 0 | NeutrophiNop58 |
| 0 | -1.15005 | 0.003 | 0.32 | 0 | NeutrophiMafb |
| 0 | -1.15011 | 0.023 | 0.528 | 0 | NeutrophiNdufc1 |
| 0 | -1.15111 | 0.035 | 0.53 | 0 | NeutrophiPura |
| 0 | -1.15463 | 0.066 | 0.614 | 0 | NeutrophiNdufb10 |
| 0 | -1.15539 | 0.041 | 0.547 | 0 | NeutrophiAtp5g3 |
| 0 | -1.1562 | 0.003 | 0.441 | 0 | NeutrophiEng |
| 0 | -1.15752 | 0.043 | 0.546 | 0 | NeutrophiSet |
| 0 | -1.15772 | 0.01 | 0.52 | 0 | NeutrophiGlrx3 |
| 0 | -1.15783 | 0.003 | 0.314 | 0 | NeutrophiPgf |
| 0 | -1.15991 | 0.139 | 0.716 | 0 | NeutrophiPsma3 |
| 0 | -1.16124 | 0.015 | 0.428 | 0 | NeutrophiTubb2a |
| 0 | -1.16217 | 0.01 | 0.476 | 0 | NeutrophiIl6st |
| 0 | -1.16267 | 0.086 | 0.612 | 0 | NeutrophiVcp |
| 0 | -1.16346 | 0.148 | 0.686 | 0 | NeutrophiCapns1 |
| 0 | -1.16391 | 0.071 | 0.601 | 0 | NeutrophiCycs |
| 0 | -1.16492 | 0.005 | 0.472 | 0 | NeutrophiLman1 |
| 0 | -1.16492 | 0.006 | 0.455 | 0 | NeutrophiP3h3 |
| 0 | -1.16634 | 0.318 | 0.869 | 0 | NeutrophiUqcrh |
| 0 | -1.1707 | 0.01 | 0.518 | 0 | NeutrophiNdufa5 |
| 0 | -1.17203 | 0.074 | 0.604 | 0 | NeutrophiP4hb |
| 0 | -1.17282 | 0.034 | 0.572 | 0 | NeutrophiNdufb5 |
| 0 | -1.1749 | 0.006 | 0.471 | 0 | NeutrophiFkbp7 |
| 0 | -1.17566 | 0.005 | 0.458 | 0 | NeutrophiCnn3 |
| 0 | -1.18228 | 0.026 | 0.413 | 0 | NeutrophiPla1a |
| 0 | -1.18262 | 0.008 | 0.344 | 0 | NeutrophiSdc4 |
| 0 | -1.18322 | 0.004 | 0.368 | 0 | NeutrophiGas6 |
| 0 | -1.18508 | 0.002 | 0.42 | 0 | NeutrophiNdrg2 |
| 0 | -1.18544 | 0.031 | 0.561 | 0 | NeutrophiCct2 |
| 0 | -1.18553 | 0.005 | 0.443 | 0 | NeutrophiMmp23 |
| 0 | -1.18913 | 0.098 | 0.636 | 0 | NeutrophiHnrnpa3 |
| 0 | -1.19075 | 0.014 | 0.399 | 0 | NeutrophiHsph1 |
| 0 | -1.19179 | 0.008 | 0.479 | 0 | NeutrophiTmem256 |
| 0 | -1.19266 | 0.221 | 0.798 | 0 | NeutrophiHmgb1 |
| 0 | -1.19509 | 0.076 | 0.603 | 0 | NeutrophiOat |
| 0 | -1.19602 | 0.035 | 0.561 | 0 | NeutrophiCct4 |
| 0 | -1.19796 | 0.004 | 0.433 | 0 | NeutrophiVwa1 |
| 0 | -1.19933 | 0.013 | 0.504 | 0 | NeutrophiComt |

| | | | | | |
|---|---|---|---|---|---|
| 0 | -1.19987 | 0.036 | 0.564 | 0 | NeutrophiEif3m |
| 0 | -1.2005 | 0.024 | 0.54 | 0 | NeutrophiAnapc5 |
| 0 | -1.20212 | 0.006 | 0.475 | 0 | NeutrophiRcn1 |
| 0 | -1.20342 | 0.049 | 0.601 | 0 | NeutrophiCct5 |
| 0 | -1.20452 | 0.026 | 0.554 | 0 | NeutrophiSptssa |
| 0 | -1.20529 | 0.013 | 0.517 | 0 | NeutrophiRanbp1 |
| 0 | -1.20539 | 0.005 | 0.436 | 0 | NeutrophiDpysl3 |
| 0 | -1.20603 | 0.021 | 0.537 | 0 | NeutrophiRexo2 |
| 0 | -1.20732 | 0.007 | 0.472 | 0 | NeutrophiFgfr1 |
| 0 | -1.20773 | 0.317 | 0.706 | 0 | NeutrophiRhob |
| 0 | -1.20886 | 0.07 | 0.618 | 0 | NeutrophiAnp32b |
| 0 | -1.2096 | 0.004 | 0.346 | 0 | NeutrophiFmo2 |
| 0 | -1.21051 | 0.005 | 0.391 | 0 | NeutrophiFxyd6 |
| 0 | -1.21192 | 0.043 | 0.563 | 0 | NeutrophiDnajc3 |
| 0 | -1.2123 | 0.017 | 0.549 | 0 | NeutrophiNdufa11 |
| 0 | -1.21255 | 0.079 | 0.614 | 0 | NeutrophiAplp2 |
| 0 | -1.21321 | 0.007 | 0.456 | 0 | NeutrophiCavin1 |
| 0 | -1.21633 | 0.201 | 0.726 | 0 | NeutrophiCtsb |
| 0 | -1.21783 | 0.01 | 0.501 | 0 | NeutrophiPea15a |
| 0 | -1.2236 | 0.018 | 0.336 | 0 | NeutrophiThy1 |
| 0 | -1.22487 | 0.009 | 0.479 | 0 | NeutrophiSlc1a5 |
| 0 | -1.22682 | 0.005 | 0.376 | 0 | NeutrophiFibin |
| 0 | -1.2273 | 0.897 | 0.99 | 0 | NeutrophiRps27 |
| 0 | -1.22985 | 0.005 | 0.418 | 0 | NeutrophiCol4a2 |
| 0 | -1.23156 | 0.007 | 0.451 | 0 | NeutrophiIl11ra1 |
| 0 | -1.23615 | 0.008 | 0.418 | 0 | NeutrophiKcnq1ot1 |
| 0 | -1.23757 | 0.847 | 0.99 | 0 | NeutrophiRps27a |
| 0 | -1.24245 | 0.008 | 0.37 | 0 | NeutrophiAdamts1 |
| 0 | -1.24898 | 0.08 | 0.67 | 0 | NeutrophiSec11a |
| 0 | -1.2497 | 0.31 | 0.719 | 0 | NeutrophiPtms |
| 0 | -1.2526 | 0.009 | 0.508 | 0 | NeutrophiTspan3 |
| 0 | -1.25294 | 0.141 | 0.743 | 0 | NeutrophiPsmb1 |
| 0 | -1.25416 | 0.082 | 0.639 | 0 | NeutrophiNdufv3 |
| 0 | -1.25665 | 0.04 | 0.586 | 0 | NeutrophiDpm3 |
| 0 | -1.26284 | 0.024 | 0.546 | 0 | NeutrophiSlc50a1 |
| 0 | -1.26674 | 0.062 | 0.636 | 0 | NeutrophiPsmb6 |
| 0 | -1.26847 | 0.003 | 0.396 | 0 | NeutrophiSfrp1 |
| 0 | -1.26969 | 0.014 | 0.54 | 0 | NeutrophiSnrpf |
| 0 | -1.26987 | 0.005 | 0.474 | 0 | NeutrophiMfge8 |
| 0 | -1.27132 | 0.041 | 0.601 | 0 | NeutrophiEmc10 |
| 0 | -1.27442 | 0.062 | 0.648 | 0 | NeutrophiSnu13 |
| 0 | -1.27693 | 0.182 | 0.793 | 0 | NeutrophiAtp5d |
| 0 | -1.27725 | 0.038 | 0.529 | 0 | NeutrophiDap |
| 0 | -1.27726 | 0.022 | 0.571 | 0 | NeutrophiMdh1 |
| 0 | -1.27944 | 0.065 | 0.654 | 0 | NeutrophiPark7 |
| 0 | -1.28317 | 0.086 | 0.656 | 0 | NeutrophiSwi5 |
| 0 | -1.28364 | 0.07 | 0.658 | 0 | NeutrophiCox7b |
| 0 | -1.2876 | 0.095 | 0.639 | 0 | NeutrophiMbnl1 |
| 0 | -1.29125 | 0.039 | 0.556 | 0 | NeutrophiKdelr2 |
| 0 | -1.29157 | 0.038 | 0.601 | 0 | NeutrophiSnrpe |
| 0 | -1.29349 | 0.023 | 0.563 | 0 | NeutrophiSsr2 |
| 0 | -1.29711 | 0.005 | 0.399 | 0 | NeutrophiCol5a3 |
| 0 | -1.29804 | 0.008 | 0.498 | 0 | NeutrophiDpysl2 |
| 0 | -1.30525 | 0.12 | 0.69 | 0 | NeutrophiAkr1a1 |

| | | | | | |
|---|---|---|---|---|---|
| 0 | -1.30558 | 0.003 | 0.447 | 0 | NeutrophiAebp1 |
| 0 | -1.30812 | 0.102 | 0.717 | 0 | NeutrophiSnrpb |
| 0 | -1.30942 | 0.019 | 0.545 | 0 | NeutrophiNme1 |
| 0 | -1.31295 | 0.013 | 0.562 | 0 | NeutrophiNdufc2 |
| 0 | -1.31693 | 0.031 | 0.612 | 0 | NeutrophiSnrpd2 |
| 0 | -1.31946 | 0.011 | 0.492 | 0 | NeutrophiRora |
| 0 | -1.32025 | 0.126 | 0.712 | 0 | NeutrophiYwhae |
| 0 | -1.32453 | 0.285 | 0.853 | 0 | NeutrophiCox6c |
| 0 | -1.32536 | 0.041 | 0.563 | 0 | NeutrophiMat2a |
| 0 | -1.32579 | 0.003 | 0.48 | 0 | NeutrophiFermt2 |
| 0 | -1.32764 | 0.005 | 0.357 | 0 | NeutrophiTppp3 |
| 0 | -1.33044 | 0.23 | 0.764 | 0 | NeutrophiIfngr1 |
| 0 | -1.33168 | 0.005 | 0.461 | 0 | NeutrophiLama4 |
| 0 | -1.33777 | 0.026 | 0.608 | 0 | NeutrophiEif3i |
| 0 | -1.3398 | 0.007 | 0.467 | 0 | NeutrophiNav1 |
| 0 | -1.34031 | 0.032 | 0.547 | 0 | NeutrophiMif |
| 0 | -1.34259 | 0.134 | 0.743 | 0 | NeutrophiAtp5a1 |
| 0 | -1.34385 | 0.096 | 0.555 | 0 | NeutrophiRhoc |
| 0 | -1.34509 | 0.133 | 0.727 | 0 | NeutrophiYwhaq |
| 0 | -1.34943 | 0.084 | 0.568 | 0 | NeutrophiGm11808 |
| 0 | -1.35131 | 0.008 | 0.437 | 0 | NeutrophiTm4sf1 |
| 0 | -1.35182 | 0.008 | 0.285 | 0 | NeutrophiIrf8 |
| 0 | -1.35463 | 0.069 | 0.65 | 0 | NeutrophiSfr1 |
| 0 | -1.355 | 0.061 | 0.403 | 0 | NeutrophiUgdh |
| 0 | -1.35511 | 0.026 | 0.539 | 0 | NeutrophiHspd1 |
| 0 | -1.35577 | 0.008 | 0.5 | 0 | NeutrophiFkbp9 |
| 0 | -1.35666 | 0.01 | 0.402 | 0 | NeutrophiBag3 |
| 0 | -1.35979 | 0.004 | 0.476 | 0 | NeutrophiMxra7 |
| 0 | -1.3622 | 0.004 | 0.446 | 0 | NeutrophiAngptl2 |
| 0 | -1.3667 | 0.023 | 0.498 | 0 | NeutrophiNfia |
| 0 | -1.36886 | 0.051 | 0.628 | 0 | NeutrophiRps27l |
| 0 | -1.37761 | 0.006 | 0.429 | 0 | NeutrophiHtra1 |
| 0 | -1.38198 | 0.133 | 0.768 | 0 | NeutrophiEif3k |
| 0 | -1.38331 | 0.262 | 0.771 | 0 | NeutrophiEmp3 |
| 0 | -1.38441 | 0.017 | 0.521 | 0 | NeutrophiRras |
| 0 | -1.38466 | 0.233 | 0.842 | 0 | NeutrophiAtp5h |
| 0 | -1.38661 | 0.049 | 0.656 | 0 | NeutrophiAtp5o.1 |
| 0 | -1.38806 | 0.331 | 0.602 | 0 | NeutrophiCrispld2 |
| 0 | -1.38869 | 0.241 | 0.747 | 0 | NeutrophiLamp1 |
| 0 | -1.39179 | 0.066 | 0.661 | 0 | Neutrophimt-Nd5 |
| 0 | -1.399 | 0.009 | 0.501 | 0 | NeutrophiPkd2 |
| 0 | -1.3992 | 0.008 | 0.493 | 0 | NeutrophiCald1 |
| 0 | -1.40327 | 0.055 | 0.376 | 0 | NeutrophiEts1 |
| 0 | -1.4053 | 0.005 | 0.465 | 0 | NeutrophiMedag |
| 0 | -1.40666 | 0.015 | 0.511 | 0 | NeutrophiPpp1r14b |
| 0 | -1.4089 | 0.007 | 0.531 | 0 | NeutrophiVkorc1 |
| 0 | -1.41523 | 0.006 | 0.401 | 0 | NeutrophiGsta3 |
| 0 | -1.41954 | 0.007 | 0.477 | 0 | NeutrophiEmilin1 |
| 0 | -1.42146 | 0.007 | 0.477 | 0 | NeutrophiSocs2 |
| 0 | -1.42314 | 0.063 | 0.674 | 0 | NeutrophiRan |
| 0 | -1.42387 | 0.09 | 0.705 | 0 | NeutrophiSsr4 |
| 0 | -1.42592 | 0.023 | 0.585 | 0 | NeutrophiMrpl52 |
| 0 | -1.4321 | 0.043 | 0.533 | 0 | NeutrophiNfic |
| 0 | -1.43339 | 0.006 | 0.45 | 0 | NeutrophiPpp1r14a |

| | | | | | |
|---|---|---|---|---|---|
| 0 | -1.43436 | 0.011 | 0.505 | 0 | NeutrophiItgb5 |
| 0 | -1.43687 | 0.006 | 0.509 | 0 | NeutrophiS100a16 |
| 0 | -1.43767 | 0.137 | 0.548 | 0 | NeutrophiPhlda1 |
| 0 | -1.44548 | 0.008 | 0.447 | 0 | NeutrophiSerpine2 |
| 0 | -1.44622 | 0.215 | 0.614 | 0 | NeutrophiFosb |
| 0 | -1.44634 | 0.007 | 0.501 | 0 | NeutrophiPdgfra |
| 0 | -1.45374 | 0.006 | 0.46 | 0 | NeutrophiSelenbp1 |
| 0 | -1.45713 | 0.007 | 0.474 | 0 | NeutrophiSod3 |
| 0 | -1.45772 | 0.007 | 0.34 | 0 | NeutrophiAckr3 |
| 0 | -1.45857 | 0.087 | 0.636 | 0 | NeutrophiDbi |
| 0 | -1.45929 | 0.041 | 0.67 | 0 | NeutrophiRtraf |
| 0 | -1.46412 | 0.039 | 0.607 | 0 | NeutrophiArl1 |
| 0 | -1.47205 | 0.01 | 0.541 | 0 | NeutrophiCalu |
| 0 | -1.47368 | 0.007 | 0.416 | 0 | NeutrophiEln |
| 0 | -1.4756 | 0.057 | 0.659 | 0 | NeutrophiNsa2 |
| 0 | -1.47718 | 0.008 | 0.488 | 0 | NeutrophiCol5a2 |
| 0 | -1.47741 | 0.009 | 0.5 | 0 | NeutrophiFhl1 |
| 0 | -1.48842 | 0.044 | 0.68 | 0 | NeutrophiTbca |
| 0 | -1.49461 | 0.005 | 0.474 | 0 | NeutrophiAce |
| 0 | -1.49574 | 0.204 | 0.824 | 0 | NeutrophiNpc2 |
| 0 | -1.5071 | 0.748 | 0.988 | 0 | NeutrophiRpl37 |
| 0 | -1.50892 | 0.006 | 0.498 | 0 | NeutrophiEntpd2 |
| 0 | -1.51175 | 0.011 | 0.421 | 0 | NeutrophiGfpt2 |
| 0 | -1.52421 | 0.012 | 0.604 | 0 | NeutrophiPrdx2 |
| 0 | -1.53101 | 0.162 | 0.815 | 0 | NeutrophiSumo2 |
| 0 | -1.53373 | 0.006 | 0.524 | 0 | NeutrophiSlc29a1 |
| 0 | -1.5385 | 0.092 | 0.747 | 0 | NeutrophiNdufa4 |
| 0 | -1.54434 | 0.005 | 0.47 | 0 | NeutrophiFbn1 |
| 0 | -1.5453 | 0.449 | 0.949 | 0 | NeutrophiRps25 |
| 0 | -1.54757 | 0.006 | 0.494 | 0 | NeutrophiAdamts2 |
| 0 | -1.54888 | 0.009 | 0.501 | 0 | NeutrophiCol5a1 |
| 0 | -1.54985 | 0.006 | 0.488 | 0 | NeutrophiFbln5 |
| 0 | -1.55014 | 0.283 | 0.908 | 0 | NeutrophiBtf3 |
| 0 | -1.552 | 0.187 | 0.829 | 0 | NeutrophiCox7c |
| 0 | -1.55489 | 0.008 | 0.558 | 0 | NeutrophiTxndc5 |
| 0 | -1.558 | 0.352 | 0.67 | 0 | NeutrophiCd63 |
| 0 | -1.55839 | 0.167 | 0.854 | 0 | NeutrophiCnbp |
| 0 | -1.55935 | 0.024 | 0.467 | 0 | NeutrophiUap1 |
| 0 | -1.57489 | 0.012 | 0.521 | 0 | NeutrophiDdah2 |
| 0 | -1.57991 | 0.073 | 0.728 | 0 | NeutrophiSerbp1 |
| 0 | -1.58789 | 0.398 | 0.814 | 0 | NeutrophiH2-K1 |
| 0 | -1.59842 | 0.288 | 0.89 | 0 | NeutrophiYbx1 |
| 0 | -1.60155 | 0.012 | 0.512 | 0 | NeutrophiItm2a |
| 0 | -1.60386 | 0.008 | 0.411 | 0 | NeutrophiNbl1 |
| 0 | -1.60926 | 0.031 | 0.683 | 0 | NeutrophiAtp5g1 |
| 0 | -1.60954 | 0.01 | 0.565 | 0 | NeutrophiFcgrt |
| 0 | -1.61384 | 0.007 | 0.517 | 0 | NeutrophiLama2 |
| 0 | -1.61805 | 0.067 | 0.722 | 0 | NeutrophiTmed9 |
| 0 | -1.61823 | 0.006 | 0.543 | 0 | NeutrophiFxyd1 |
| 0 | -1.62036 | 0.027 | 0.678 | 0 | NeutrophiEif3e |
| 0 | -1.62693 | 0.026 | 0.646 | 0 | NeutrophiRpn2 |
| 0 | -1.64165 | 0.037 | 0.685 | 0 | NeutrophiUqcrb |
| 0 | -1.64348 | 0.049 | 0.625 | 0 | NeutrophiTmed3 |
| 0 | -1.64704 | 0.018 | 0.628 | 0 | NeutrophiHmgn1 |

| | | | | | |
|---|---|---|---|---|---|
| 0 | -1.65596 | 0.005 | 0.531 | 0 | NeutrophiBicc1 |
| 0 | -1.6627 | 0.039 | 0.543 | 0 | NeutrophiPnp |
| 0 | -1.66663 | 0.008 | 0.471 | 0 | NeutrophiCol6a3 |
| 0 | -1.67412 | 0.074 | 0.766 | 0 | NeutrophiAtp5g2 |
| 0 | -1.67502 | 0.03 | 0.642 | 0 | NeutrophiItm2c |
| 0 | -1.67873 | 0.03 | 0.642 | 0 | NeutrophiItgb1 |
| 0 | -1.6834 | 0.008 | 0.524 | 0 | NeutrophiPlxdc2 |
| 0 | -1.68392 | 0.033 | 0.631 | 0 | NeutrophiSmc6 |
| 0 | -1.6871 | 0.007 | 0.461 | 0 | NeutrophiPcolce2 |
| 0 | -1.69014 | 0.009 | 0.523 | 0 | NeutrophiNupr1 |
| 0 | -1.69505 | 0.02 | 0.484 | 0 | NeutrophiAtf5 |
| 0 | -1.69935 | 0.006 | 0.526 | 0 | NeutrophiCavin3 |
| 0 | -1.69953 | 0.023 | 0.339 | 0 | NeutrophiCd3e |
| 0 | -1.70786 | 0.013 | 0.473 | 0 | NeutrophiGem |
| 0 | -1.70917 | 0.01 | 0.505 | 0 | NeutrophiPam |
| 0 | -1.71091 | 0.008 | 0.508 | 0 | NeutrophiRamp2 |
| 0 | -1.71297 | 0.009 | 0.524 | 0 | NeutrophiPtgis |
| 0 | -1.71665 | 0.032 | 0.651 | 0 | NeutrophiManf |
| 0 | -1.71901 | 0.007 | 0.51 | 0 | NeutrophiOaf |
| 0 | -1.72793 | 0.019 | 0.677 | 0 | NeutrophiPebp1 |
| 0 | -1.74373 | 0.012 | 0.433 | 0 | NeutrophiAspn |
| 0 | -1.75155 | 0.008 | 0.427 | 0 | NeutrophiSpon2 |
| 0 | -1.75182 | 0.04 | 0.719 | 0 | NeutrophiGlo1 |
| 0 | -1.75524 | 0.049 | 0.759 | 0 | NeutrophiDad1 |
| 0 | -1.75539 | 0.212 | 0.865 | 0 | NeutrophiDynll1 |
| 0 | -1.75576 | 0.008 | 0.487 | 0 | NeutrophiAdamts5 |
| 0 | -1.77488 | 0.013 | 0.341 | 0 | NeutrophiCtgf |
| 0 | -1.79592 | 0.009 | 0.549 | 0 | NeutrophiCpq |
| 0 | -1.80878 | 0.128 | 0.756 | 0 | NeutrophiBsg |
| 0 | -1.81142 | 0.028 | 0.585 | 0 | NeutrophiNedd4 |
| 0 | -1.81547 | 0.057 | 0.614 | 0 | NeutrophiRnase4 |
| 0 | -1.81862 | 0.05 | 0.672 | 0 | NeutrophiZfp36l1 |
| 0 | -1.82397 | 0.012 | 0.538 | 0 | NeutrophiOlfml3 |
| 0 | -1.82701 | 0.051 | 0.767 | 0 | NeutrophiSnrpg |
| 0 | -1.83198 | 0.011 | 0.574 | 0 | NeutrophiCamk2n1 |
| 0 | -1.83203 | 0.016 | 0.665 | 0 | NeutrophiDdost |
| 0 | -1.84191 | 0.019 | 0.574 | 0 | NeutrophiEmp1 |
| 0 | -1.84388 | 0.007 | 0.53 | 0 | NeutrophiTcf21 |
| 0 | -1.85271 | 0.007 | 0.474 | 0 | NeutrophiCol8a1 |
| 0 | -1.85744 | 0.008 | 0.562 | 0 | NeutrophiRhoj |
| 0 | -1.86804 | 0.19 | 0.734 | 0 | NeutrophiLaptm4a |
| 0 | -1.869 | 0.007 | 0.464 | 0 | NeutrophiTimp3 |
| 0 | -1.86977 | 0.038 | 0.656 | 0 | NeutrophiPdia6 |
| 0 | -1.87068 | 0.19 | 0.613 | 0 | NeutrophiDnajb1 |
| 0 | -1.8707 | 0.012 | 0.542 | 0 | NeutrophiEbf1 |
| 0 | -1.87111 | 0.149 | 0.715 | 0 | NeutrophiUba52 |
| 0 | -1.87443 | 0.254 | 0.895 | 0 | NeutrophiEef1b2 |
| 0 | -1.88284 | 0.125 | 0.821 | 0 | NeutrophiSec61g |
| 0 | -1.88422 | 0.067 | 0.806 | 0 | NeutrophiHint1 |
| 0 | -1.88473 | 0.088 | 0.433 | 0 | NeutrophiGm12840 |
| 0 | -1.89397 | 0.019 | 0.622 | 0 | NeutrophiLmna |
| 0 | -1.90048 | 0.016 | 0.375 | 0 | NeutrophiMt2 |
| 0 | -1.91037 | 0.1 | 0.835 | 0 | NeutrophiEif3h |
| 0 | -1.92347 | 0.008 | 0.516 | 0 | NeutrophiIslr |

| | | | | | |
|---|---|---|---|---|---|
| 0 | -1.9238 | 0.009 | 0.53 | 0 | NeutrophiSpry1 |
| 0 | -1.92858 | 0.027 | 0.556 | 0 | NeutrophiErrfi1 |
| 0 | -1.93529 | 0.013 | 0.599 | 0 | NeutrophiCd302 |
| 0 | -1.93886 | 0.014 | 0.292 | 0 | NeutrophiMfap4 |
| 0 | -1.94036 | 0.073 | 0.818 | 0 | NeutrophiEef1d |
| 0 | -1.94176 | 0.008 | 0.571 | 0 | NeutrophiNfib |
| 0 | -1.95411 | 0.008 | 0.554 | 0 | NeutrophiGpm6b |
| 0 | -1.95489 | 0.061 | 0.787 | 0 | NeutrophiEef1g |
| 0 | -1.95739 | 0.603 | 0.984 | 0 | NeutrophiRpl17 |
| 0 | -1.96068 | 0.017 | 0.567 | 0 | NeutrophiAbca8a |
| 0 | -1.97695 | 0.101 | 0.784 | 0 | NeutrophiSelenof |
| 0 | -1.9799 | 0.011 | 0.587 | 0 | NeutrophiRcn3 |
| 0 | -1.99141 | 0.009 | 0.574 | 0 | NeutrophiMxra8 |
| 0 | -1.99613 | 0.012 | 0.512 | 0 | NeutrophiOgn |
| 0 | -2.00715 | 0.026 | 0.741 | 0 | NeutrophiKrtcap2 |
| 0 | -2.00832 | 0.013 | 0.581 | 0 | NeutrophiSdc2 |
| 0 | -2.00904 | 0.01 | 0.536 | 0 | NeutrophiLamb1 |
| 0 | -2.01582 | 0.771 | 0.996 | 0 | NeutrophiRpl41 |
| 0 | -2.02035 | 0.025 | 0.666 | 0 | NeutrophiYbx3 |
| 0 | -2.02165 | 0.186 | 0.902 | 0 | NeutrophiEif3f |
| 0 | -2.03015 | 0.026 | 0.737 | 0 | NeutrophiFkbp1a |
| 0 | -2.03316 | 0.044 | 0.748 | 0 | NeutrophiTubb5 |
| 0 | -2.03468 | 0.038 | 0.748 | 0 | NeutrophiHspe1 |
| 0 | -2.03577 | 0.014 | 0.562 | 0 | NeutrophiTnxb |
| 0 | -2.03581 | 0.012 | 0.623 | 0 | NeutrophiNenf |
| 0 | -2.04575 | 0.744 | 0.992 | 0 | NeutrophiRps29 |
| 0 | -2.04579 | 0.018 | 0.575 | 0 | NeutrophiSlc43a3 |
| 0 | -2.04783 | 0.029 | 0.603 | 0 | NeutrophiSerpinb6a |
| 0 | -2.0734 | 0.073 | 0.708 | 0 | NeutrophiRpl13a |
| 0 | -2.07643 | 0.009 | 0.555 | 0 | NeutrophiPlpp3 |
| 0 | -2.07673 | 0.013 | 0.566 | 0 | NeutrophiPrelp |
| 0 | -2.08374 | 0.68 | 0.991 | 0 | NeutrophiRpl18a |
| 0 | -2.0928 | 0.032 | 0.723 | 0 | NeutrophiTuba1b |
| 0 | -2.09833 | 0.267 | 0.941 | 0 | NeutrophiRpl27 |
| 0 | -2.10104 | 0.877 | 0.997 | 0 | NeutrophiTpt1 |
| 0 | -2.10423 | 0.771 | 0.994 | 0 | NeutrophiRps16 |
| 0 | -2.11227 | 0.014 | 0.608 | 0 | NeutrophiTcf4 |
| 0 | -2.11298 | 0.158 | 0.621 | 0 | NeutrophiSocs3 |
| 0 | -2.11486 | 0.011 | 0.571 | 0 | NeutrophiLoxl1 |
| 0 | -2.11973 | 0.09 | 0.665 | 0 | NeutrophiMtch1 |
| 0 | -2.13639 | 0.091 | 0.764 | 0 | NeutrophiPdia3 |
| 0 | -2.13676 | 0.093 | 0.839 | 0 | NeutrophiTagln2 |
| 0 | -2.14021 | 0.37 | 0.921 | 0 | NeutrophiGnas |
| 0 | -2.14697 | 0.011 | 0.549 | 0 | NeutrophiPcsk6 |
| 0 | -2.15355 | 0.02 | 0.562 | 0 | NeutrophiEcm1 |
| 0 | -2.15495 | 0.012 | 0.595 | 0 | NeutrophiLamc1 |
| 0 | -2.15513 | 0.018 | 0.616 | 0 | NeutrophiKlf9 |
| 0 | -2.16273 | 0.121 | 0.875 | 0 | NeutrophiRpl31 |
| 0 | -2.16333 | 0.048 | 0.821 | 0 | NeutrophiRpl36al |
| 0 | -2.17201 | 0.061 | 0.829 | 0 | Neutrophimt-Nd3 |
| 0 | -2.1828 | 0.066 | 0.707 | 0 | NeutrophiCyb5a |
| 0 | -2.18586 | 0.601 | 0.961 | 0 | NeutrophiHspa8 |
| 0 | -2.18766 | 0.616 | 0.98 | 0 | NeutrophiRps10 |
| 0 | -2.18886 | 0.696 | 0.989 | 0 | NeutrophiRpl9 |

| | | | | | |
|---|---|---|---|---|---|
| 0 | -2.19102 | 0.105 | 0.71 | 0 | NeutrophiSelenop |
| 0 | -2.19193 | 0.01 | 0.589 | 0 | NeutrophiCrip2 |
| 0 | -2.20863 | 0.028 | 0.721 | 0 | NeutrophiAhnak |
| 0 | -2.21587 | 0.066 | 0.844 | 0 | NeutrophiNpm1 |
| 0 | -2.22071 | 0.016 | 0.528 | 0 | NeutrophiCkb |
| 0 | -2.2228 | 0.013 | 0.587 | 0 | NeutrophiRbp1 |
| 0 | -2.23328 | 0.023 | 0.727 | 0 | NeutrophiSlc25a4 |
| 0 | -2.23578 | 0.012 | 0.576 | 0 | NeutrophiHspg2 |
| 0 | -2.23832 | 0.011 | 0.562 | 0 | NeutrophiCol4a1 |
| 0 | -2.23912 | 0.01 | 0.586 | 0 | NeutrophiNfix |
| 0 | -2.24153 | 0.014 | 0.594 | 0 | NeutrophiAxl |
| 0 | -2.2513 | 0.664 | 0.984 | 0 | NeutrophiRps12 |
| 0 | -2.25577 | 0.038 | 0.667 | 0 | NeutrophiSptbn1 |
| 0 | -2.26085 | 0.488 | 0.981 | 0 | NeutrophiRps13 |
| 0 | -2.27045 | 0.405 | 0.979 | 0 | NeutrophiRplp2 |
| 0 | -2.29042 | 0.013 | 0.601 | 0 | NeutrophiPmp22 |
| 0 | -2.29067 | 0.014 | 0.631 | 0 | NeutrophiSelenom |
| 0 | -2.29148 | 0.011 | 0.62 | 0 | NeutrophiLrp1 |
| 0 | -2.30703 | 0.014 | 0.549 | 0 | NeutrophiGas1 |
| 0 | -2.31347 | 0.452 | 0.985 | 0 | NeutrophiRps14 |
| 0 | -2.31683 | 0.437 | 0.978 | 0 | NeutrophiRpl10 |
| 0 | -2.33308 | 0.12 | 0.854 | 0 | NeutrophiPpib |
| 0 | -2.34701 | 0.013 | 0.399 | 0 | NeutrophiCyr61 |
| 0 | -2.34966 | 0.015 | 0.621 | 0 | NeutrophiLhfp |
| 0 | -2.36256 | 0.16 | 0.934 | 0 | NeutrophiNaca |
| 0 | -2.36798 | 0.545 | 0.685 | 0 | NeutrophiCebpd |
| 0 | -2.36944 | 0.528 | 0.971 | 0 | NeutrophiRpl30 |
| 0 | -2.38012 | 0.602 | 0.989 | 0 | NeutrophiRpl23 |
| 0 | -2.38284 | 0.149 | 0.734 | 0 | NeutrophiAnxa5 |
| 0 | -2.38611 | 0.162 | 0.693 | 0 | NeutrophiCtsl |
| 0 | -2.38696 | 0.016 | 0.584 | 0 | NeutrophiNid1 |
| 0 | -2.39337 | 0.044 | 0.818 | 0 | NeutrophiPrdx1 |
| 0 | -2.39986 | 0.081 | 0.866 | 0 | NeutrophiRps17 |
| 0 | -2.40185 | 0.519 | 0.986 | 0 | NeutrophiRpl34 |
| 0 | -2.40632 | 0.031 | 0.571 | 0 | NeutrophiAW112010 |
| 0 | -2.41371 | 0.525 | 0.991 | 0 | NeutrophiRpl35a |
| 0 | -2.4264 | 0.216 | 0.935 | 0 | NeutrophiRpl23a |
| 0 | -2.43086 | 0.017 | 0.603 | 0 | NeutrophiDpt |
| 0 | -2.43182 | 0.474 | 0.984 | 0 | NeutrophiRps3 |
| 0 | -2.43785 | 0.41 | 0.97 | 0 | NeutrophiEef2 |
| 0 | -2.44315 | 0.02 | 0.639 | 0 | NeutrophiPmepa1 |
| 0 | -2.46695 | 0.331 | 0.975 | 0 | NeutrophiRpl24 |
| 0 | -2.47717 | 0.023 | 0.592 | 0 | NeutrophiFstl1 |
| 0 | -2.47842 | 0.245 | 0.964 | 0 | NeutrophiRps26 |
| 0 | -2.48245 | 0.143 | 0.826 | 0 | NeutrophiCalr |
| 0 | -2.48795 | 0.491 | 0.986 | 0 | NeutrophiRpl37a |
| 0 | -2.50617 | 0.067 | 0.787 | 0 | NeutrophiHsp90b1 |
| 0 | -2.514 | 0.042 | 0.871 | 0 | NeutrophiSelenow |
| 0 | -2.52182 | 0.012 | 0.494 | 0 | NeutrophiDpep1 |
| 0 | -2.52373 | 0.016 | 0.488 | 0 | NeutrophiMeg3 |
| 0 | -2.52802 | 0.535 | 0.989 | 0 | NeutrophiRpl27a |
| 0 | -2.52935 | 0.528 | 0.995 | 0 | Neutrophimt-Co1 |
| 0 | -2.5358 | 0.155 | 0.775 | 0 | NeutrophiTuba1a |
| 0 | -2.53835 | 0.186 | 0.95 | 0 | NeutrophiRpl26 |

| | | | | | |
|---|---|---|---|---|---|
| 0 | -2.56445 | 0.206 | 0.949 | 0 | NeutrophiRps6 |
| 0 | -2.5866 | 0.044 | 0.42 | 0 | NeutrophiCxcl1 |
| 0 | -2.59004 | 0.025 | 0.647 | 0 | NeutrophiGstm1 |
| 0 | -2.60756 | 0.446 | 0.983 | 0 | NeutrophiRpl21 |
| 0 | -2.6116 | 0.221 | 0.957 | 0 | NeutrophiRpl7a |
| 0 | -2.61185 | 0.372 | 0.982 | 0 | NeutrophiRps23 |
| 0 | -2.62317 | 0.425 | 0.986 | 0 | NeutrophiRpl8 |
| 0 | -2.63183 | 0.023 | 0.577 | 0 | NeutrophiCol15a1 |
| 0 | -2.63736 | 0.024 | 0.596 | 0 | NeutrophiId3 |
| 0 | -2.63826 | 0.366 | 0.982 | 0 | NeutrophiRpl18 |
| 0 | -2.64144 | 0.289 | 0.941 | 0 | NeutrophiHsp90ab1 |
| 0 | -2.64825 | 0.426 | 0.978 | 0 | NeutrophiRpl28 |
| 0 | -2.65576 | 0.02 | 0.591 | 0 | NeutrophiFbln2 |
| 0 | -2.67012 | 0.207 | 0.965 | 0 | NeutrophiRps15 |
| 0 | -2.67271 | 0.023 | 0.613 | 0 | NeutrophiMmp2 |
| 0 | -2.6741 | 0.049 | 0.89 | 0 | NeutrophiRpl22l1 |
| 0 | -2.6764 | 0.03 | 0.678 | 0 | NeutrophiZbtb20 |
| 0 | -2.67662 | 0.451 | 0.983 | 0 | NeutrophiRps21 |
| 0 | -2.68225 | 0.115 | 0.935 | 0 | NeutrophiRpl5 |
| 0 | -2.68946 | 0.016 | 0.593 | 0 | NeutrophiCol6a2 |
| 0 | -2.69249 | 0.016 | 0.602 | 0 | NeutrophiSerpinf1 |
| 0 | -2.69295 | 0.026 | 0.608 | 0 | NeutrophiCfh |
| 0 | -2.70034 | 0.346 | 0.982 | 0 | NeutrophiRpl7 |
| 0 | -2.70348 | 0.309 | 0.974 | 0 | Neutrophimt-Nd4 |
| 0 | -2.70468 | 0.201 | 0.857 | 0 | NeutrophiHsp90aa1 |
| 0 | -2.71655 | 0.019 | 0.591 | 0 | NeutrophiCcdc80 |
| 0 | -2.73287 | 0.017 | 0.639 | 0 | NeutrophiRarres2 |
| 0 | -2.73369 | 0.338 | 0.984 | 0 | NeutrophiRpl11 |
| 0 | -2.73768 | 0.027 | 0.534 | 0 | NeutrophiMfap5 |
| 0 | -2.74328 | 0.406 | 0.981 | 0 | NeutrophiRps11 |
| 0 | -2.76056 | 0.319 | 0.985 | 0 | NeutrophiRpl38 |
| 0 | -2.76541 | 0.39 | 0.986 | 0 | NeutrophiRpl39 |
| 0 | -2.77073 | 0.021 | 0.618 | 0 | NeutrophiCd34 |
| 0 | -2.77411 | 0.023 | 0.604 | 0 | NeutrophiCol6a1 |
| 0 | -2.78826 | 0.127 | 0.944 | 0 | NeutrophiRpl4 |
| 0 | -2.81168 | 0.565 | 0.994 | 0 | NeutrophiRps8 |
| 0 | -2.83313 | 0.023 | 0.584 | 0 | NeutrophiSmoc2 |
| 0 | -2.84262 | 0.05 | 0.844 | 0 | NeutrophiS100a10 |
| 0 | -2.8537 | 0.467 | 0.994 | 0 | Neutrophimt-Co2 |
| 0 | -2.86439 | 0.144 | 0.978 | 0 | Neutrophimt-Nd1 |
| 0 | -2.86743 | 0.372 | 0.982 | 0 | NeutrophiRps7 |
| 0 | -2.86949 | 0.343 | 0.88 | 0 | NeutrophiVim |
| 0 | -2.89718 | 0.026 | 0.572 | 0 | NeutrophiLy6a |
| 0 | -2.89827 | 0.368 | 0.986 | 0 | NeutrophiRps3a1 |
| 0 | -2.90495 | 0.13 | 0.664 | 0 | NeutrophiEgr1 |
| 0 | -2.94263 | 0.601 | 0.997 | 0 | Neutrophimt-Co3 |
| 0 | -2.9459 | 0.205 | 0.976 | 0 | NeutrophiRpl36 |
| 0 | -2.96359 | 0.119 | 0.951 | 0 | NeutrophiRack1 |
| 0 | -2.97003 | 0.111 | 0.956 | 0 | NeutrophiRpl22 |
| 0 | -2.98334 | 0.057 | 0.687 | 0 | NeutrophiCd81 |
| 0 | -2.99636 | 0.209 | 0.973 | 0 | NeutrophiRpl15 |
| 0 | -2.99732 | 0.268 | 0.981 | 0 | NeutrophiRpl6 |
| 0 | -3.00221 | 0.157 | 0.973 | 0 | NeutrophiPpia |
| 0 | -3.01167 | 0.142 | 0.947 | 0 | Neutrophimt-Nd2 |

| | | | | | |
|---|---|---|---|---|---|
| 0 | -3.02092 | 0.021 | 0.638 | 0 | NeutrophiCygb |
| 0 | -3.02657 | 0.342 | 0.987 | 0 | NeutrophiRpl19 |
| 0 | -3.02777 | 0.108 | 0.96 | 0 | NeutrophiRpl36a |
| 0 | -3.05345 | 0.479 | 0.992 | 0 | NeutrophiRplp1 |
| 0 | -3.05783 | 0.554 | 0.998 | 0 | Neutrophimt-Atp6 |
| 0 | -3.06579 | 0.327 | 0.989 | 0 | Neutrophimt-Cytb |
| 0 | -3.07607 | 0.206 | 0.977 | 0 | NeutrophiPtma |
| 0 | -3.07616 | 0.139 | 0.974 | 0 | NeutrophiRpl14 |
| 0 | -3.08578 | 0.026 | 0.616 | 0 | NeutrophiKlf4 |
| 0 | -3.10356 | 0.291 | 0.983 | 0 | NeutrophiRps2 |
| 0 | -3.10443 | 0.034 | 0.683 | 0 | NeutrophiIgfbp4 |
| 0 | -3.13508 | 0.105 | 0.944 | 0 | NeutrophiRpl35 |
| 0 | -3.14317 | 0.194 | 0.97 | 0 | NeutrophiRps28 |
| 0 | -3.14524 | 0.065 | 0.846 | 0 | NeutrophiCrip1 |
| 0 | -3.17698 | 0.071 | 0.511 | 0 | NeutrophiApoe |
| 0 | -3.20261 | 0.041 | 0.647 | 0 | NeutrophiLtbp4 |
| 0 | -3.20398 | 0.347 | 0.99 | 0 | NeutrophiRplp0 |
| 0 | -3.20411 | 0.028 | 0.636 | 0 | NeutrophiPcolce |
| 0 | -3.22704 | 0.439 | 0.992 | 0 | NeutrophiRps24 |
| 0 | -3.25903 | 0.111 | 0.959 | 0 | NeutrophiRps18 |
| 0 | -3.26156 | 0.233 | 0.985 | 0 | NeutrophiRps15a |
| 0 | -3.26466 | 0.099 | 0.958 | 0 | NeutrophiRpl12 |
| 0 | -3.26741 | 0.616 | 0.995 | 0 | NeutrophiEef1a1 |
| 0 | -3.28552 | 0.041 | 0.616 | 0 | NeutrophiCol1a1 |
| 0 | -3.29231 | 0.029 | 0.612 | 0 | NeutrophiFbln1 |
| 0 | -3.32485 | 0.035 | 0.521 | 0 | NeutrophiHspb1 |
| 0 | -3.33152 | 0.076 | 0.968 | 0 | NeutrophiRpl10a |
| 0 | -3.3583 | 0.031 | 0.637 | 0 | NeutrophiGpx3 |
| 0 | -3.37327 | 0.117 | 0.954 | 0 | NeutrophiTmsb10 |
| 0 | -3.40207 | 0.229 | 0.988 | 0 | NeutrophiRps5 |
| 0 | -3.40223 | 0.054 | 0.852 | 0 | NeutrophiLgals1 |
| 0 | -3.4171 | 0.023 | 0.621 | 0 | NeutrophiSerpinh1 |
| 0 | -3.42821 | 0.097 | 0.973 | 0 | NeutrophiRpl3 |
| 0 | -3.44439 | 0.411 | 0.996 | 0 | NeutrophiRpl13 |
| 0 | -3.48478 | 0.208 | 0.986 | 0 | NeutrophiRps19 |
| 0 | -3.50514 | 0.185 | 0.987 | 0 | NeutrophiRpl32 |
| 0 | -3.51286 | 0.035 | 0.635 | 0 | NeutrophiCol1a2 |
| 0 | -3.58775 | 0.144 | 0.98 | 0 | NeutrophiRps20 |
| 0 | -3.63859 | 0.052 | 0.653 | 0 | NeutrophiLum |
| 0 | -3.64595 | 0.257 | 0.991 | 0 | NeutrophiRps4x |
| 0 | -3.68607 | 0.063 | 0.626 | 0 | NeutrophiCol3a1 |
| 0 | -3.69418 | 0.025 | 0.652 | 0 | NeutrophiSerping1 |
| 0 | -3.70692 | 0.159 | 0.984 | 0 | NeutrophiRpsa |
| 0 | -3.76022 | 0.04 | 0.604 | 0 | NeutrophiLpl |
| 0 | -3.84133 | 0.063 | 0.664 | 0 | NeutrophiSparcl1 |
| 0 | -3.87911 | 0.037 | 0.648 | 0 | NeutrophiClec3b |
| 0 | -3.88654 | 0.109 | 0.677 | 0 | NeutrophiHspa1a |
| 0 | -4.0249 | 0.123 | 0.656 | 0 | NeutrophiMt1 |
| 0 | -4.02861 | 0.059 | 0.667 | 0 | NeutrophiHtra3 |
| 0 | -4.04477 | 0.099 | 0.727 | 0 | NeutrophiHspa1b |
| 0 | -4.05738 | 0.13 | 0.783 | 0 | NeutrophiJun |
| 0 | -4.19862 | 0.052 | 0.661 | 0 | NeutrophiSparc |
| 0 | -4.31318 | 0.066 | 0.676 | 0 | NeutrophiBgn |
| 0 | -4.46677 | 0.101 | 0.704 | 0 | NeutrophiIgfbp7 |

| | | | | | |
|---|---|---|---|---|---|
| 0 | -4.71633 | 0.116 | 0.696 | 0 | NeutrophiMgp |
| 0 | -4.94699 | 0.609 | 0.816 | 0 | NeutrophiGsn |
| 0 | -5.75959 | 0.253 | 0.752 | 0 | NeutrophiDcn |
| ######## | 1.056544 | 0.268 | 0.082 | ######## | NeutrophiKlhl2 |
| ######## | 1.280501 | 0.454 | 0.26 | ######## | NeutrophiAnxa11 |
| ######## | 1.122724 | 0.341 | 0.136 | ######## | NeutrophiXpo6 |
| ######## | 1.149699 | 0.594 | 0.447 | ######## | NeutrophiVamp8 |
| ######## | 1.142959 | 0.34 | 0.133 | ######## | NeutrophiPag1 |
| ######## | 1.372169 | 0.425 | 0.219 | ######## | NeutrophiHmgn2 |
| ######## | -1.72459 | 0.019 | 0.271 | ######## | NeutrophiS100a4 |
| ######## | -1.06301 | 0.626 | 0.733 | ######## | NeutrophiIfitm3 |
| ######## | 1.10924 | 0.379 | 0.169 | ######## | NeutrophiLbr |
| ######## | 1.059651 | 0.274 | 0.088 | ######## | NeutrophiTtc7 |
| ######## | 1.44284 | 0.594 | 0.451 | ######## | NeutrophiAdgre5 |
| ######## | 1.107328 | 0.508 | 0.316 | ######## | NeutrophiApbb1ip |
| ######## | 1.521339 | 0.609 | 0.524 | ######## | NeutrophiMgst1 |
| ######## | 1.149497 | 0.318 | 0.124 | ######## | NeutrophiSmim3 |
| ######## | 1.000521 | 0.33 | 0.13 | ######## | NeutrophiArrb2 |
| ######## | 1.297298 | 0.487 | 0.314 | ######## | NeutrophiSbno1 |
| ######## | 1.286521 | 0.562 | 0.433 | ######## | NeutrophiHectd1 |
| ######## | 1.209689 | 0.333 | 0.143 | ######## | NeutrophiSp140 |
| ######## | 1.265914 | 0.549 | 0.424 | ######## | NeutrophiCreg1 |
| ######## | 1.09557 | 0.275 | 0.096 | ######## | NeutrophiAbr |
| ######## | 1.146025 | 0.279 | 0.1 | ######## | NeutrophiEll2 |
| ######## | -1.26355 | 0.841 | 0.845 | ######## | NeutrophiCst3 |
| ######## | 1.063999 | 0.612 | 0.506 | ######## | NeutrophiSupt4a |
| ######## | 1.222136 | 0.452 | 0.282 | ######## | NeutrophiRaf1 |
| ######## | 1.351848 | 0.289 | 0.098 | ######## | NeutrophiCcl4 |
| ######## | 1.023119 | 0.712 | 0.729 | ######## | NeutrophiD8Ertd738e |
| ######## | -1.02007 | 0.134 | 0.404 | ######## | NeutrophiGadd45g |
| ######## | 1.074637 | 0.294 | 0.122 | ######## | NeutrophiMtus1 |
| ######## | 1.409103 | 0.317 | 0.142 | ######## | NeutrophiHist1h2bc |
| ######## | 1.36379 | 0.602 | 0.547 | ######## | NeutrophiCtnnb1 |
| ######## | 1.196653 | 0.44 | 0.277 | ######## | NeutrophiHipk1 |
| ######## | 1.378592 | 0.636 | 0.584 | ######## | NeutrophiAnxa1 |
| ######## | 1.269563 | 0.468 | 0.314 | ######## | NeutrophiPer1 |
| ######## | 1.297837 | 0.387 | 0.212 | ######## | NeutrophiPnpla2 |
| ######## | 1.213109 | 0.309 | 0.138 | ######## | NeutrophiBraf |
| ######## | 1.158575 | 0.39 | 0.221 | ######## | NeutrophiPxn |
| ######## | 1.177956 | 0.521 | 0.389 | ######## | NeutrophiPtpn1 |
| ######## | 1.30648 | 0.489 | 0.364 | ######## | NeutrophiSelenon |
| ######## | 1.258481 | 0.45 | 0.306 | ######## | NeutrophiTcn2 |
| ######## | 1.225433 | 0.466 | 0.302 | ######## | NeutrophiGm26532 |
| ######## | 1.174405 | 0.452 | 0.307 | ######## | NeutrophiMax |
| ######## | 1.056218 | 0.525 | 0.398 | ######## | NeutrophiPtbp3 |
| ######## | 1.113103 | 0.558 | 0.502 | ######## | Neutrophi1110008F13Rik |
| ######## | 1.092579 | 0.463 | 0.317 | ######## | NeutrophiCdc42se1 |
| ######## | 1.245456 | 0.56 | 0.453 | ######## | NeutrophiKdm6b |
| ######## | 1.080555 | 0.491 | 0.373 | ######## | NeutrophiRnf130 |
| ######## | 1.034272 | 0.291 | 0.131 | ######## | NeutrophiNotch1 |
| ######## | 1.389754 | 0.367 | 0.2 | ######## | NeutrophiHist1h1c |
| ######## | 1.135191 | 0.347 | 0.191 | ######## | NeutrophiHgsnat |
| ######## | 1.234819 | 0.373 | 0.218 | ######## | NeutrophiIgf1r |
| ######## | 1.102444 | 0.296 | 0.139 | ######## | NeutrophiDusp6 |

| | | | | | | |
|---|---|---|---|---|---|---|
| ######## | 1.166962 | 0.48 | 0.371 | ######## | Neutrophi | Nadk |
| ######## | 1.034784 | 0.403 | 0.254 | ######## | Neutrophi | Hpcal1 |
| ######## | 1.229426 | 0.432 | 0.297 | ######## | Neutrophi | Ddit3 |
| ######## | 1.067056 | 0.376 | 0.223 | ######## | Neutrophi | Kdm7a |
| ######## | -1.2957 | 0.732 | 0.788 | ######## | Neutrophi | Fos |
| ######## | 1.132951 | 0.533 | 0.5 | ######## | Neutrophi | Tkt |
| ######## | -1.54778 | 0.506 | 0.604 | ######## | Neutrophi | Pi16 |
| ######## | 1.050179 | 0.48 | 0.383 | ######## | Neutrophi | Ssu72 |
| ######## | 1.218285 | 0.533 | 0.461 | ######## | Neutrophi | Cox17 |
| ######## | 1.192418 | 0.276 | 0.129 | ######## | Neutrophi | Trf |
| ######## | 1.053783 | 0.447 | 0.344 | ######## | Neutrophi | Map2k3 |
| ######## | 1.06094 | 0.533 | 0.473 | ######## | Neutrophi | Coq10b |
| ######## | 1.081147 | 0.261 | 0.127 | ######## | Neutrophi | St3gal6 |
| ######## | 1.007637 | 0.312 | 0.174 | ######## | Neutrophi | Mboat7 |
| ######## | 1.073853 | 0.36 | 0.227 | ######## | Neutrophi | Notch2 |
| ######## | 1.117517 | 0.261 | 0.121 | ######## | Neutrophi | Ptgs2 |
| ######## | 1.531148 | 0.513 | 0.439 | ######## | Neutrophi | Nfkbiz |
| ######## | 1.088348 | 0.341 | 0.212 | ######## | Neutrophi | Tmem189 |
| ######## | 1.230583 | 0.454 | 0.375 | ######## | Neutrophi | Mapkapk2 |
| ######## | 1.074538 | 0.361 | 0.245 | ######## | Neutrophi | Preb |
| ######## | -1.14485 | 0.225 | 0.408 | ######## | Neutrophi | C3 |
| ######## | 1.028414 | 0.428 | 0.358 | ######## | Neutrophi | Etf1 |
| 2.05E-82 | 1.073467 | 0.42 | 0.375 | 3.69E-78 | Neutrophi | Emd |
| 4.07E-82 | 1.075118 | 0.4 | 0.327 | 7.33E-78 | Neutrophi | Gadd45a |
| 4.80E-76 | 1.65551 | 0.436 | 0.378 | 8.64E-72 | Neutrophi | Thbs1 |
| 8.86E-75 | -2.08193 | 0.647 | 0.675 | 1.60E-70 | Neutrophi | Hbb-bs |
| 2.50E-72 | 1.088426 | 0.382 | 0.329 | 4.50E-68 | Neutrophi | Plin2 |
| 4.83E-36 | 1.493389 | 0.606 | 0.716 | 8.70E-32 | Neutrophi | Nfkbia |
| 2.73E-30 | 1.049703 | 0.444 | 0.487 | 4.91E-26 | Neutrophi | Atp2b1 |
| 2.45E-22 | -2.0963 | 0.479 | 0.506 | 4.41E-18 | Neutrophi | Hba-a1 |
| 0 | 3.169906 | 0.899 | 0.064 | 0 | T cells | Trbc2 |
| 0 | 2.804744 | 0.89 | 0.061 | 0 | T cells | Cd3g |
| 0 | 2.608806 | 0.788 | 0.041 | 0 | T cells | Trac |
| 0 | 2.50985 | 0.724 | 0.053 | 0 | T cells | Cd28 |
| 0 | 2.488974 | 0.795 | 0.087 | 0 | T cells | Ms4a4b |
| 0 | 2.374579 | 0.524 | 0.065 | 0 | T cells | Trbc1 |
| 0 | 2.335249 | 0.906 | 0.141 | 0 | T cells | Cd3d |
| 0 | 2.329106 | 0.704 | 0.095 | 0 | T cells | Gimap6 |
| 0 | 2.2516 | 0.487 | 0.047 | 0 | T cells | Ccr7 |
| 0 | 2.244881 | 0.456 | 0.033 | 0 | T cells | Icos |
| 0 | 2.220824 | 0.763 | 0.092 | 0 | T cells | Ms4a6b |
| 0 | 2.208727 | 0.517 | 0.078 | 0 | T cells | Dusp10 |
| 0 | 2.174123 | 0.867 | 0.234 | 0 | T cells | Ets1 |
| 0 | 2.161649 | 0.725 | 0.084 | 0 | T cells | Cd2 |
| 0 | 2.133503 | 0.725 | 0.052 | 0 | T cells | Lat |
| 0 | 2.076032 | 0.978 | 0.69 | 0 | T cells | H2-K1 |
| 0 | 2.074876 | 0.785 | 0.296 | 0 | T cells | Vps37b |
| 0 | 2.051239 | 0.328 | 0.02 | 0 | T cells | Ctla4 |
| 0 | 2.046058 | 0.882 | 0.192 | 0 | T cells | Cd3e |
| 0 | 1.985146 | 0.301 | 0.006 | 0 | T cells | Tnfrsf4 |
| 0 | 1.965119 | 0.512 | 0.029 | 0 | T cells | Il7r |
| 0 | 1.956397 | 0.987 | 0.741 | 0 | T cells | Rpl12 |
| 0 | 1.952248 | 0.651 | 0.062 | 0 | T cells | Gimap3 |
| 0 | 1.935432 | 0.673 | 0.075 | 0 | T cells | Gimap1 |

| | | | | | | |
|---|---|---|---|---|---|---|
| 0 | 1.915571 | 0.702 | 0.076 | 0 | T cells | Lck |
| 0 | 1.890965 | 0.484 | 0.02 | 0 | T cells | Lef1 |
| 0 | 1.829037 | 0.995 | 0.854 | 0 | T cells | Rps24 |
| 0 | 1.817647 | 0.993 | 0.741 | 0 | T cells | Tmsb10 |
| 0 | 1.768642 | 0.555 | 0.044 | 0 | T cells | Itk |
| 0 | 1.767053 | 0.634 | 0.207 | 0 | T cells | Crlf3 |
| 0 | 1.759156 | 0.532 | 0.151 | 0 | T cells | Satb1 |
| 0 | 1.752392 | 0.832 | 0.205 | 0 | T cells | Ptpn18 |
| 0 | 1.748583 | 0.442 | 0.041 | 0 | T cells | Tnfrsf18 |
| 0 | 1.725825 | 0.632 | 0.058 | 0 | T cells | Skap1 |
| 0 | 1.720368 | 0.552 | 0.137 | 0 | T cells | Gramd3 |
| 0 | 1.716374 | 0.526 | 0.053 | 0 | T cells | Cd27 |
| 0 | 1.710753 | 0.993 | 0.829 | 0 | T cells | Rps7 |
| 0 | 1.709786 | 0.568 | 0.06 | 0 | T cells | Gimap4 |
| 0 | 1.701426 | 0.994 | 0.797 | 0 | T cells | Rps15a |
| 0 | 1.621092 | 0.429 | 0.096 | 0 | T cells | Rgs1 |
| 0 | 1.617557 | 0.993 | 0.778 | 0 | T cells | Rpsa |
| 0 | 1.610745 | 0.993 | 0.792 | 0 | T cells | Rps19 |
| 0 | 1.603731 | 0.99 | 0.771 | 0 | T cells | Rps20 |
| 0 | 1.600024 | 0.996 | 0.83 | 0 | T cells | Rplp0 |
| 0 | 1.597403 | 0.996 | 0.966 | 0 | T cells | Rps27 |
| 0 | 1.590738 | 0.998 | 0.938 | 0 | T cells | Rps16 |
| 0 | 1.573917 | 0.994 | 0.857 | 0 | T cells | Rps13 |
| 0 | 1.570713 | 0.978 | 0.747 | 0 | T cells | Rps18 |
| 0 | 1.569542 | 0.994 | 0.856 | 0 | T cells | Rps3 |
| 0 | 1.559502 | 0.994 | 0.826 | 0 | T cells | Rpl19 |
| 0 | 1.546175 | 0.401 | 0.007 | 0 | T cells | Cd4 |
| 0 | 1.540299 | 0.993 | 0.835 | 0 | T cells | Rplp2 |
| 0 | 1.527523 | 0.995 | 0.787 | 0 | T cells | Rpl32 |
| 0 | 1.527101 | 0.994 | 0.931 | 0 | T cells | Rps29 |
| 0 | 1.506439 | 0.565 | 0.112 | 0 | T cells | H2-Q7 |
| 0 | 1.504502 | 0.459 | 0.039 | 0 | T cells | Gm8369 |
| 0 | 1.50328 | 0.557 | 0.1 | 0 | T cells | Ptprcap |
| 0 | 1.502344 | 0.962 | 0.728 | 0 | T cells | Rpl5 |
| 0 | 1.49862 | 0.416 | 0.025 | 0 | T cells | Cd6 |
| 0 | 1.497819 | 0.989 | 0.889 | 0 | T cells | Rps10 |
| 0 | 1.493399 | 0.992 | 0.838 | 0 | T cells | Rpl39 |
| 0 | 1.492715 | 0.647 | 0.277 | 0 | T cells | Zc3hav1 |
| 0 | 1.479234 | 0.476 | 0.034 | 0 | T cells | Cd247 |
| 0 | 1.47143 | 0.431 | 0.033 | 0 | T cells | 1700097N02Rik |
| 0 | 1.461608 | 0.467 | 0.167 | 0 | T cells | Smc4 |
| 0 | 1.455205 | 0.996 | 0.888 | 0 | T cells | Rpl17 |
| 0 | 1.451373 | 0.995 | 0.837 | 0 | T cells | Rps11 |
| 0 | 1.433621 | 0.663 | 0.413 | 0 | T cells | Saraf |
| 0 | 1.433496 | 0.992 | 0.828 | 0 | T cells | Rpl18 |
| 0 | 1.433396 | 0.611 | 0.14 | 0 | T cells | 1-Sep |
| 0 | 1.429389 | 0.993 | 0.851 | 0 | T cells | Rps14 |
| 0 | 1.428492 | 0.427 | 0.065 | 0 | T cells | Cd69 |
| 0 | 1.42741 | 0.57 | 0.228 | 0 | T cells | Thy1 |
| 0 | 1.415844 | 0.361 | 0.015 | 0 | T cells | Cd5 |
| 0 | 1.414824 | 0.397 | 0.023 | 0 | T cells | Bcl11b |
| 0 | 1.412115 | 0.831 | 0.56 | 0 | T cells | Uba52 |
| 0 | 1.411517 | 0.994 | 0.832 | 0 | T cells | Rps3a1 |
| 0 | 1.395129 | 0.992 | 0.865 | 0 | T cells | Rplp1 |

| | | | | | | |
|---|---|---|---|---|---|---|
| 0 | 1.394247 | 0.996 | 0.913 | 0 | T cells | Rpl18a |
| 0 | 1.390744 | 0.993 | 0.818 | 0 | T cells | Rpl38 |
| 0 | 1.38836 | 0.974 | 0.77 | 0 | T cells | Rpl27 |
| 0 | 1.383016 | 0.932 | 0.731 | 0 | T cells | Eef1b2 |
| 0 | 1.381636 | 0.989 | 0.783 | 0 | T cells | Rpl36 |
| 0 | 1.375898 | 0.965 | 0.763 | 0 | T cells | Rps6 |
| 0 | 1.374129 | 0.895 | 0.644 | 0 | T cells | Npm1 |
| 0 | 1.374073 | 0.529 | 0.102 | 0 | T cells | Itgb7 |
| 0 | 1.371974 | 0.981 | 0.745 | 0 | T cells | Rpl10a |
| 0 | 1.371322 | 0.388 | 0.02 | 0 | T cells | Tcf7 |
| 0 | 1.371105 | 0.732 | 0.383 | 0 | T cells | Psmb8 |
| 0 | 1.370927 | 0.991 | 0.85 | 0 | T cells | Rps21 |
| 0 | 1.364863 | 0.976 | 0.777 | 0 | T cells | Rps28 |
| 0 | 1.362834 | 0.992 | 0.754 | 0 | T cells | Rpl3 |
| 0 | 1.347869 | 0.997 | 0.892 | 0 | T cells | Rpl23 |
| 0 | 1.335985 | 0.966 | 0.786 | 0 | T cells | Rps26 |
| 0 | 1.333663 | 0.994 | 0.829 | 0 | T cells | Rps23 |
| 0 | 1.328462 | 0.526 | 0.096 | 0 | T cells | Ptpn22 |
| 0 | 1.323463 | 0.461 | 0.062 | 0 | T cells | P2ry10 |
| 0 | 1.319856 | 0.444 | 0.061 | 0 | T cells | Gimap9 |
| 0 | 1.319387 | 0.985 | 0.859 | 0 | T cells | Rpl30 |
| 0 | 1.319048 | 0.99 | 0.847 | 0 | T cells | Rpl8 |
| 0 | 1.313577 | 0.994 | 0.822 | 0 | T cells | Rpl11 |
| 0 | 1.306874 | 0.793 | 0.485 | 0 | T cells | Mbnl1 |
| 0 | 1.298762 | 0.888 | 0.667 | 0 | T cells | Shisa5 |
| 0 | 1.297819 | 0.994 | 0.869 | 0 | T cells | Rpl34 |
| 0 | 1.288026 | 0.81 | 0.561 | 0 | T cells | Rps27rt |
| 0 | 1.281244 | 0.594 | 0.19 | 0 | T cells | Tnfaip3 |
| 0 | 1.27695 | 0.43 | 0.053 | 0 | T cells | Txk |
| 0 | 1.275643 | 0.46 | 0.116 | 0 | T cells | Dgka |
| 0 | 1.270408 | 0.553 | 0.204 | 0 | T cells | Itpkb |
| 0 | 1.265337 | 0.992 | 0.799 | 0 | T cells | Rps5 |
| 0 | 1.262248 | 0.994 | 0.862 | 0 | T cells | Rpl37a |
| 0 | 1.262052 | 0.983 | 0.782 | 0 | T cells | Rpl15 |
| 0 | 1.260031 | 0.989 | 0.849 | 0 | T cells | Rpl21 |
| 0 | 1.255715 | 0.966 | 0.742 | 0 | T cells | Rack1 |
| 0 | 1.247501 | 0.992 | 0.803 | 0 | T cells | Rpl6 |
| 0 | 1.245202 | 0.995 | 0.876 | 0 | T cells | Rpl27a |
| 0 | 1.234257 | 0.967 | 0.748 | 0 | T cells | Rpl36a |
| 0 | 1.232397 | 0.383 | 0.028 | 0 | T cells | Klk8 |
| 0 | 1.23237 | 0.942 | 0.736 | 0 | T cells | Rpl35 |
| 0 | 1.228894 | 0.995 | 0.916 | 0 | T cells | Rpl9 |
| 0 | 1.224649 | 0.437 | 0.062 | 0 | T cells | Sh2d2a |
| 0 | 1.217515 | 0.967 | 0.776 | 0 | T cells | Rps15 |
| 0 | 1.214468 | 0.366 | 0.026 | 0 | T cells | Inpp4b |
| 0 | 1.213704 | 0.996 | 0.808 | 0 | T cells | Rps4x |
| 0 | 1.200071 | 0.997 | 0.94 | 0 | T cells | Rpl41 |
| 0 | 1.198164 | 0.306 | 0.047 | 0 | T cells | Gpr183 |
| 0 | 1.194026 | 0.437 | 0.09 | 0 | T cells | H2-Q6 |
| 0 | 1.192319 | 0.973 | 0.744 | 0 | T cells | Rpl22 |
| 0 | 1.190149 | 0.995 | 0.954 | 0 | T cells | Rps27a |
| 0 | 1.188677 | 0.353 | 0.034 | 0 | T cells | Zap70 |
| 0 | 1.177768 | 0.99 | 0.875 | 0 | T cells | Rpl35a |
| 0 | 1.167361 | 0.32 | 0.023 | 0 | T cells | S1pr1 |

| | | | | | | |
|---|---|---|---|---|---|---|
| 0 | 1.158636 | 0.637 | 0.242 | 0 | T cells | Ltb |
| 0 | 1.158202 | 0.339 | 0.029 | 0 | T cells | Grap2 |
| 0 | 1.156105 | 0.303 | 0.046 | 0 | T cells | Traf1 |
| 0 | 1.146128 | 0.37 | 0.039 | 0 | T cells | Gpr132 |
| 0 | 1.137896 | 0.981 | 0.765 | 0 | T cells | Rpl14 |
| 0 | 1.136192 | 0.923 | 0.738 | 0 | T cells | Pabpc1 |
| 0 | 1.132438 | 0.984 | 0.898 | 0 | T cells | B2m |
| 0 | 1.125218 | 0.514 | 0.208 | 0 | T cells | Pdcd4 |
| 0 | 1.124779 | 0.711 | 0.262 | 0 | T cells | Hcst |
| 0 | 1.114807 | 0.987 | 0.811 | 0 | T cells | Rps2 |
| 0 | 1.11012 | 0.992 | 0.823 | 0 | T cells | Rpl7 |
| 0 | 1.106703 | 0.997 | 0.967 | 0 | T cells | Tpt1 |
| 0 | 1.100831 | 0.998 | 0.851 | 0 | T cells | Rpl13 |
| 0 | 1.084581 | 0.389 | 0.063 | 0 | T cells | Rhoh |
| 0 | 1.078705 | 0.954 | 0.74 | 0 | T cells | Rpl4 |
| 0 | 1.071788 | 0.943 | 0.755 | 0 | T cells | Rpl23a |
| 0 | 1.071665 | 0.309 | 0.034 | 0 | T cells | Il21r |
| 0 | 1.064238 | 0.499 | 0.156 | 0 | T cells | Dusp2 |
| 0 | 1.063115 | 0.456 | 0.102 | 0 | T cells | Ikzf1 |
| 0 | 1.06296 | 0.997 | 0.901 | 0 | T cells | Eef1a1 |
| 0 | 1.061607 | 0.948 | 0.74 | 0 | T cells | Naca |
| 0 | 1.047332 | 0.997 | 0.991 | 0 | T cells | Fau |
| 0 | 1.045816 | 0.867 | 0.623 | 0 | T cells | Rpl36al |
| 0 | 1.041642 | 0.993 | 0.888 | 0 | T cells | Rps8 |
| 0 | 1.040069 | 0.431 | 0.08 | 0 | T cells | Il2rg |
| 0 | 1.04 | 0.99 | 0.814 | 0 | T cells | Rpl24 |
| 0 | 1.036102 | 0.595 | 0.196 | 0 | T cells | Fyb |
| 0 | 1.033675 | 0.986 | 0.769 | 0 | T cells | Ppia |
| 0 | 1.030799 | 0.882 | 0.683 | 0 | T cells | Rpl22l1 |
| 0 | 1.029539 | 0.665 | 0.256 | 0 | T cells | Fam107b |
| 0 | 1.023484 | 0.951 | 0.761 | 0 | T cells | Rpl26 |
| 0 | 1.020322 | 0.974 | 0.841 | 0 | T cells | Rpl28 |
| 0 | 1.009426 | 0.35 | 0.081 | 0 | T cells | Frat2 |
| 0 | 1.008668 | 0.346 | 0.05 | 0 | T cells | Gimap5 |
| 0 | 1.004569 | 0.733 | 0.31 | 0 | T cells | Limd2 |
| 0 | 1.000353 | 0.295 | 0.044 | 0 | T cells | Tagap |
| 0 | -1.19081 | 0.627 | 0.905 | 0 | T cells | Gabarap |
| 0 | -1.20756 | 0.008 | 0.422 | 0 | T cells | Fermt2 |
| 0 | -1.23363 | 0.014 | 0.44 | 0 | T cells | Fkbp9 |
| 0 | -1.25239 | 0.018 | 0.439 | 0 | T cells | Selenon |
| 0 | -1.26555 | 0.013 | 0.434 | 0 | T cells | Cald1 |
| 0 | -1.27325 | 0.018 | 0.44 | 0 | T cells | Pkd2 |
| 0 | -1.29236 | 0.014 | 0.447 | 0 | T cells | S100a16 |
| 0 | -1.30022 | 0.015 | 0.444 | 0 | T cells | Itgb5 |
| 0 | -1.31155 | 0.249 | 0.677 | 0 | T cells | Ptms |
| 0 | -1.33508 | 0.014 | 0.441 | 0 | T cells | Pdgfra |
| 0 | -1.33747 | 0.015 | 0.442 | 0 | T cells | Nfia |
| 0 | -1.35034 | 0.049 | 0.473 | 0 | T cells | Nfic |
| 0 | -1.35851 | 0.016 | 0.44 | 0 | T cells | Fhl1 |
| 0 | -1.3813 | 0.011 | 0.438 | 0 | T cells | Entpd2 |
| 0 | -1.39954 | 0.315 | 0.676 | 0 | T cells | Lamp1 |
| 0 | -1.40366 | 0.011 | 0.434 | 0 | T cells | Adamts2 |
| 0 | -1.40571 | 0.163 | 0.627 | 0 | T cells | Ctsz |
| 0 | -1.41348 | 0.103 | 0.571 | 0 | T cells | Dstn |

| | | | | | | |
|---|---|---|---|---|---|---|
| 0 | -1.42072 | 0.009 | 0.429 | 0 | T cells | Fbln5 |
| 0 | -1.42176 | 0.014 | 0.441 | 0 | T cells | Col5a1 |
| 0 | -1.42369 | 0.893 | 0.96 | 0 | T cells | Itm2b |
| 0 | -1.44615 | 0.019 | 0.458 | 0 | T cells | Ddah2 |
| 0 | -1.45878 | 0.018 | 0.476 | 0 | T cells | Fxyd1 |
| 0 | -1.46898 | 0.082 | 0.582 | 0 | T cells | Tnfrsf1a |
| 0 | -1.47114 | 0.015 | 0.455 | 0 | T cells | Lama2 |
| 0 | -1.48429 | 0.022 | 0.496 | 0 | T cells | Fcgrt |
| 0 | -1.5025 | 0.989 | 0.997 | 0 | T cells | Fth1 |
| 0 | -1.50666 | 0.119 | 0.639 | 0 | T cells | Lamp2 |
| 0 | -1.50955 | 0.019 | 0.46 | 0 | T cells | Plxdc2 |
| 0 | -1.50981 | 0.474 | 0.914 | 0 | T cells | S100a6 |
| 0 | -1.51432 | 0.013 | 0.467 | 0 | T cells | Bicc1 |
| 0 | -1.51836 | 0.044 | 0.506 | 0 | T cells | Rhoc |
| 0 | -1.52776 | 0.018 | 0.462 | 0 | T cells | Cavin3 |
| 0 | -1.53661 | 0.475 | 0.802 | 0 | T cells | Sat1 |
| 0 | -1.54347 | 0.018 | 0.459 | 0 | T cells | Nupr1 |
| 0 | -1.55135 | 0.017 | 0.444 | 0 | T cells | Pam |
| 0 | -1.56293 | 0.584 | 0.855 | 0 | T cells | Dusp1 |
| 0 | -1.5754 | 0.013 | 0.461 | 0 | T cells | Ptgis |
| 0 | -1.5863 | 0.017 | 0.448 | 0 | T cells | Oaf |
| 0 | -1.58995 | 0.01 | 0.447 | 0 | T cells | Ramp2 |
| 0 | -1.59678 | 0.038 | 0.557 | 0 | T cells | Myadm |
| 0 | -1.59979 | 0.954 | 0.993 | 0 | T cells | Ftl1 |
| 0 | -1.62286 | 0.01 | 0.429 | 0 | T cells | Adamts5 |
| 0 | -1.63532 | 0.052 | 0.482 | 0 | T cells | Gpm6b |
| 0 | -1.65452 | 0.468 | 0.845 | 0 | T cells | Psap |
| 0 | -1.65515 | 0.014 | 0.483 | 0 | T cells | Cpq |
| 0 | -1.66484 | 0.021 | 0.474 | 0 | T cells | Olfml3 |
| 0 | -1.67453 | 0.03 | 0.517 | 0 | T cells | Nedd4 |
| 0 | -1.67555 | 0.141 | 0.566 | 0 | T cells | Cdkn1a |
| 0 | -1.71185 | 0.015 | 0.494 | 0 | T cells | Rhoj |
| 0 | -1.72214 | 0.01 | 0.466 | 0 | T cells | Tcf21 |
| 0 | -1.72501 | 0.258 | 0.659 | 0 | T cells | Laptm4a |
| 0 | -1.72705 | 0.04 | 0.514 | 0 | T cells | Rcn3 |
| 0 | -1.75152 | 0.057 | 0.543 | 0 | T cells | Nenf |
| 0 | -1.76232 | 0.063 | 0.518 | 0 | T cells | Lamc1 |
| 0 | -1.7648 | 0.063 | 0.546 | 0 | T cells | Rnase4 |
| 0 | -1.77886 | 0.012 | 0.454 | 0 | T cells | Islr |
| 0 | -1.78534 | 0.015 | 0.502 | 0 | T cells | Nfib |
| 0 | -1.79237 | 0.031 | 0.503 | 0 | T cells | Mxra8 |
| 0 | -1.79773 | 0.297 | 0.715 | 0 | T cells | Zfp36 |
| 0 | -1.8056 | 0.167 | 0.586 | 0 | T cells | Mtch1 |
| 0 | -1.81491 | 0.018 | 0.528 | 0 | T cells | Cd302 |
| 0 | -1.82632 | 0.011 | 0.478 | 0 | T cells | Ebf1 |
| 0 | -1.82741 | 0.025 | 0.499 | 0 | T cells | Abca8a |
| 0 | -1.83195 | 0.025 | 0.471 | 0 | T cells | Lamb1 |
| 0 | -1.83725 | 0.245 | 0.822 | 0 | T cells | Anxa2 |
| 0 | -1.8392 | 0.022 | 0.511 | 0 | T cells | Sdc2 |
| 0 | -1.8678 | 0.185 | 0.58 | 0 | T cells | Zbtb20 |
| 0 | -1.87314 | 0.035 | 0.505 | 0 | T cells | Slc43a3 |
| 0 | -1.87729 | 0.013 | 0.452 | 0 | T cells | Ogn |
| 0 | -1.88584 | 0.292 | 0.645 | 0 | T cells | Anxa5 |
| 0 | -1.91712 | 0.247 | 0.668 | 0 | T cells | Rhob |

| | | | | | | |
|---|---|---|---|---|---|---|
| 0 | -1.91737 | 0.02 | 0.498 | 0 | T cells | Prelp |
| 0 | -1.9205 | 0.017 | 0.495 | 0 | T cells | Tnxb |
| 0 | -1.9217 | 0.026 | 0.534 | 0 | T cells | Tcf4 |
| 0 | -1.92583 | 0.044 | 0.54 | 0 | T cells | Klf9 |
| 0 | -1.9276 | 0.019 | 0.502 | 0 | T cells | Loxl1 |
| 0 | -1.93515 | 0.021 | 0.578 | 0 | T cells | Marcks |
| 0 | -1.93991 | 0.03 | 0.604 | 0 | T cells | App |
| 0 | -1.9438 | 0.015 | 0.488 | 0 | T cells | Plpp3 |
| 0 | -1.95036 | 0.048 | 0.519 | 0 | T cells | Axl |
| 0 | -1.95221 | 0.348 | 0.771 | 0 | T cells | Neat1 |
| 0 | -1.99736 | 0.028 | 0.515 | 0 | T cells | Rbp1 |
| 0 | -2.00779 | 0.016 | 0.483 | 0 | T cells | Pcsk6 |
| 0 | -2.01679 | 0.044 | 0.553 | 0 | T cells | Selenom |
| 0 | -2.03632 | 0.025 | 0.515 | 0 | T cells | Nfix |
| 0 | -2.04446 | 0.024 | 0.465 | 0 | T cells | Ckb |
| 0 | -2.04758 | 0.027 | 0.506 | 0 | T cells | Hspg2 |
| 0 | -2.05485 | 0.023 | 0.494 | 0 | T cells | Col4a1 |
| 0 | -2.11643 | 0.023 | 0.528 | 0 | T cells | Pmp22 |
| 0 | -2.12183 | 0.02 | 0.545 | 0 | T cells | Lrp1 |
| 0 | -2.1223 | 0.076 | 0.662 | 0 | T cells | Anxa1 |
| 0 | -2.16144 | 0.019 | 0.483 | 0 | T cells | Gas1 |
| 0 | -2.19843 | 0.022 | 0.547 | 0 | T cells | Lhfp |
| 0 | -2.20719 | 0.02 | 0.609 | 0 | T cells | Mgst1 |
| 0 | -2.22536 | 0.025 | 0.514 | 0 | T cells | Nid1 |
| 0 | -2.24707 | 0.189 | 0.654 | 0 | T cells | Cd9 |
| 0 | -2.25756 | 0.034 | 0.522 | 0 | T cells | Fstl1 |
| 0 | -2.29256 | 0.022 | 0.531 | 0 | T cells | Dpt |
| 0 | -2.30369 | 0.051 | 0.523 | 0 | T cells | Atf3 |
| 0 | -2.33054 | 0.021 | 0.435 | 0 | T cells | Dpep1 |
| 0 | -2.33076 | 0.111 | 0.71 | 0 | T cells | Litaf |
| 0 | -2.35273 | 0.138 | 0.708 | 0 | T cells | Timp2 |
| 0 | -2.37023 | 0.014 | 0.431 | 0 | T cells | Meg3 |
| 0 | -2.45089 | 0.031 | 0.52 | 0 | T cells | Fbln2 |
| 0 | -2.45712 | 0.035 | 0.519 | 0 | T cells | Ccdc80 |
| 0 | -2.46755 | 0.03 | 0.509 | 0 | T cells | Col15a1 |
| 0 | -2.47117 | 0.032 | 0.571 | 0 | T cells | Gstm1 |
| 0 | -2.47697 | 0.023 | 0.522 | 0 | T cells | Col6a2 |
| 0 | -2.47968 | 0.033 | 0.529 | 0 | T cells | Serpinf1 |
| 0 | -2.48862 | 0.039 | 0.471 | 0 | T cells | Mfap5 |
| 0 | -2.49983 | 0.029 | 0.541 | 0 | T cells | Mmp2 |
| 0 | -2.52486 | 0.122 | 0.602 | 0 | T cells | Cd81 |
| 0 | -2.52762 | 0.539 | 0.806 | 0 | T cells | Fos |
| 0 | -2.52955 | 0.038 | 0.536 | 0 | T cells | Cfh |
| 0 | -2.56238 | 0.025 | 0.563 | 0 | T cells | Rarres2 |
| 0 | -2.56247 | 0.033 | 0.544 | 0 | T cells | Cd34 |
| 0 | -2.59389 | 0.146 | 0.631 | 0 | T cells | Ctsl |
| 0 | -2.60687 | 0.028 | 0.533 | 0 | T cells | Col6a1 |
| 0 | -2.62873 | 0.04 | 0.71 | 0 | T cells | Aldh2 |
| 0 | -2.64963 | 0.033 | 0.514 | 0 | T cells | Smoc2 |
| 0 | -2.71388 | 0.543 | 0.877 | 0 | T cells | Cebpb |
| 0 | -2.77947 | 0.056 | 0.54 | 0 | T cells | Klf4 |
| 0 | -2.79251 | 0.162 | 0.833 | 0 | T cells | Ifitm2 |
| 0 | -2.80255 | 0.02 | 0.609 | 0 | T cells | Crispld2 |
| 0 | -2.81609 | 0.03 | 0.562 | 0 | T cells | Cygb |

| | | | | | | |
|---|---|---|---|---|---|---|
| 0 | -2.87499 | 0.17 | 0.778 | 0 | T cells | Ifitm3 |
| 0 | -2.93621 | 0.048 | 0.67 | 0 | T cells | Cd63 |
| 0 | -2.97977 | 0.036 | 0.561 | 0 | T cells | Pcolce |
| 0 | -3.00483 | 0.048 | 0.573 | 0 | T cells | Ltbp4 |
| 0 | -3.01255 | 0.166 | 0.594 | 0 | T cells | Egr1 |
| 0 | -3.06273 | 0.04 | 0.546 | 0 | T cells | Col1a1 |
| 0 | -3.08889 | 0.047 | 0.539 | 0 | T cells | Fbln1 |
| 0 | -3.09771 | 0.037 | 0.547 | 0 | T cells | Serpinh1 |
| 0 | -3.15431 | 0.047 | 0.561 | 0 | T cells | Gpx3 |
| 0 | -3.16005 | 0.416 | 0.899 | 0 | T cells | Cst3 |
| 0 | -3.1769 | 0.045 | 0.561 | 0 | T cells | Col1a2 |
| 0 | -3.19523 | 0.031 | 0.573 | 0 | T cells | G0s2 |
| 0 | -3.2921 | 0.053 | 0.649 | 0 | T cells | Pi16 |
| 0 | -3.33182 | 0.071 | 0.557 | 0 | T cells | Col3a1 |
| 0 | -3.40061 | 0.066 | 0.578 | 0 | T cells | Lum |
| 0 | -3.41337 | 0.176 | 0.584 | 0 | T cells | Mt1 |
| 0 | -3.42678 | 0.041 | 0.574 | 0 | T cells | Serping1 |
| 0 | -3.62021 | 0.053 | 0.571 | 0 | T cells | Clec3b |
| 0 | -3.62269 | 0.041 | 0.536 | 0 | T cells | Lpl |
| 0 | -3.6793 | 0.07 | 0.585 | 0 | T cells | Sparc |
| 0 | -3.72595 | 0.065 | 0.591 | 0 | T cells | Sparcl1 |
| 0 | -3.78678 | 0.169 | 0.622 | 0 | T cells | Igfbp7 |
| 0 | -3.85201 | 0.078 | 0.591 | 0 | T cells | Htra3 |
| 0 | -3.92829 | 0.09 | 0.599 | 0 | T cells | Bgn |
| 0 | -4.35562 | 0.085 | 0.727 | 0 | T cells | Cebpd |
| 0 | -4.45653 | 0.157 | 0.621 | 0 | T cells | Mgp |
| 0 | -5.40185 | 0.424 | 0.815 | 0 | T cells | Gsn |
| 0 | -5.40273 | 0.288 | 0.687 | 0 | T cells | Dcn |
| ######## | -1.77586 | 0.07 | 0.491 | ######## | T cells | Ier3 |
| ######## | -1.31444 | 0.017 | 0.429 | ######## | T cells | Col5a2 |
| ######## | -2.66318 | 0.03 | 0.437 | ######## | T cells | Thbs1 |
| ######## | -1.08973 | 0.011 | 0.42 | ######## | T cells | Ckap4 |
| ######## | -1.22411 | 0.013 | 0.418 | ######## | T cells | Mxra7 |
| ######## | -1.34971 | 0.012 | 0.416 | ######## | T cells | Ace |
| ######## | -1.3025 | 0.014 | 0.417 | ######## | T cells | Sod3 |
| ######## | -1.21426 | 0.01 | 0.411 | ######## | T cells | Nav1 |
| ######## | -1.28352 | 0.017 | 0.418 | ######## | T cells | Emilin1 |
| ######## | 1.027453 | 0.498 | 0.176 | ######## | T cells | Dusp5 |
| ######## | -1.60045 | 0.099 | 0.507 | ######## | T cells | Crip2 |
| ######## | -2.06429 | 0.117 | 0.515 | ######## | T cells | Id3 |
| ######## | -1.10322 | 0.014 | 0.414 | ######## | T cells | Fgfr1 |
| ######## | -1.20307 | 0.05 | 0.456 | ######## | T cells | Rras |
| ######## | -1.51037 | 0.016 | 0.414 | ######## | T cells | Col6a3 |
| ######## | -3.09105 | 0.061 | 0.459 | ######## | T cells | Hspb1 |
| ######## | 1.219925 | 0.575 | 0.288 | ######## | T cells | Peli1 |
| ######## | -1.37674 | 0.015 | 0.412 | ######## | T cells | Fbn1 |
| ######## | -1.6615 | 0.019 | 0.416 | ######## | T cells | Col8a1 |
| ######## | -1.2825 | 0.014 | 0.408 | ######## | T cells | Medag |
| ######## | -1.21345 | 0.011 | 0.405 | ######## | T cells | Lama4 |
| ######## | -1.0536 | 0.017 | 0.413 | ######## | T cells | Fkbp7 |
| ######## | -1.71545 | 0.015 | 0.408 | ######## | T cells | Timp3 |
| ######## | -2.00752 | 0.193 | 0.611 | ######## | T cells | Grina |
| ######## | -2.72262 | 0.415 | 0.668 | ######## | T cells | Jun |
| ######## | -1.24807 | 0.145 | 0.543 | ######## | T cells | Tmed3 |

| | | | | | | |
|---|---|---|---|---|---|---|
| ######## | -1.34629 | 0.008 | 0.396 | ######## | T cells | Ppp1r14a |
| ######## | -1.11765 | 0.011 | 0.401 | ######## | T cells | Cavin1 |
| ######## | -1.05928 | 0.011 | 0.4 | ######## | T cells | P3h3 |
| ######## | -1.67586 | 0.091 | 0.487 | ######## | T cells | Ecm1 |
| ######## | -1.51827 | 0.015 | 0.405 | ######## | T cells | Pcolce2 |
| ######## | -1.76534 | 0.01 | 0.395 | ######## | T cells | Tnfaip2 |
| ######## | -3.28882 | 0.072 | 0.458 | ######## | T cells | Apoe |
| ######## | -1.19249 | 0.009 | 0.392 | ######## | T cells | Aebp1 |
| ######## | 1.212303 | 0.697 | 0.431 | ######## | T cells | Gm11808 |
| ######## | -1.30792 | 0.017 | 0.404 | ######## | T cells | Selenbp1 |
| ######## | -1.52569 | 0.037 | 0.426 | ######## | T cells | Atf5 |
| ######## | -1.23846 | 0.112 | 0.523 | ######## | T cells | Dhrs7 |
| ######## | -2.04903 | 0.026 | 0.411 | ######## | T cells | C3 |
| ######## | -1.01907 | 0.077 | 0.485 | ######## | T cells | Hexa |
| ######## | -1.15507 | 0.116 | 0.524 | ######## | T cells | Sntb2 |
| ######## | -1.13968 | 0.084 | 0.482 | ######## | T cells | Txndc5 |
| ######## | -1.47808 | 0.216 | 0.568 | ######## | T cells | Sptbn1 |
| ######## | -1.04427 | 0.068 | 0.469 | ######## | T cells | Grn |
| ######## | -1.4213 | 0.064 | 0.46 | ######## | T cells | Spry1 |
| ######## | -1.10351 | 0.049 | 0.444 | ######## | T cells | Rnf130 |
| ######## | -1.11419 | 0.017 | 0.396 | ######## | T cells | Il11ra1 |
| ######## | -1.04829 | 0.013 | 0.389 | ######## | T cells | Mmp23 |
| ######## | -1.26733 | 0.011 | 0.384 | ######## | T cells | Tm4sf1 |
| ######## | -1.08709 | 0.01 | 0.383 | ######## | T cells | Dpysl3 |
| ######## | -1.03231 | 0.014 | 0.386 | ######## | T cells | Eng |
| ######## | -1.28827 | 0.018 | 0.393 | ######## | T cells | Serpine2 |
| ######## | -1.08177 | 0.009 | 0.38 | ######## | T cells | Vwa1 |
| ######## | -1.00268 | 0.009 | 0.378 | ######## | T cells | Lpar1 |
| ######## | -1.27058 | 0.011 | 0.377 | ######## | T cells | Htra1 |
| ######## | -1.00219 | 0.239 | 0.642 | ######## | T cells | Bri3 |
| ######## | 1.18356 | 0.559 | 0.289 | ######## | T cells | Smad7 |
| ######## | -1.46757 | 0.286 | 0.614 | ######## | T cells | Selenop |
| ######## | -1.63056 | 0.013 | 0.376 | ######## | T cells | Spon2 |
| ######## | -1.25659 | 0.107 | 0.495 | ######## | T cells | Emp1 |
| ######## | -1.59353 | 0.018 | 0.381 | ######## | T cells | Aspn |
| ######## | -1.22177 | 0.008 | 0.368 | ######## | T cells | Pla1a |
| ######## | 1.018382 | 0.439 | 0.181 | ######## | T cells | Srpk1 |
| ######## | -1.0846 | 0.088 | 0.466 | ######## | T cells | Calu |
| ######## | -1.00011 | 0.038 | 0.412 | ######## | T cells | Zeb2 |
| ######## | -1.11462 | 0.008 | 0.367 | ######## | T cells | Col4a2 |
| ######## | -1.04689 | 0.079 | 0.459 | ######## | T cells | Vkorc1 |
| ######## | -1.40637 | 0.013 | 0.371 | ######## | T cells | Gfpt2 |
| ######## | -1.06526 | 0.24 | 0.637 | ######## | T cells | Ppp1r2 |
| ######## | -1.38513 | 0.026 | 0.388 | ######## | T cells | Jdp2 |
| ######## | -1.51951 | 0.112 | 0.481 | ######## | T cells | Errfi1 |
| ######## | -1.17894 | 0.204 | 0.566 | ######## | T cells | Ybx3 |
| ######## | -1.34703 | 0.013 | 0.365 | ######## | T cells | Eln |
| ######## | -1.28928 | 0.013 | 0.363 | ######## | T cells | Gda |
| ######## | -2.72011 | 0.029 | 0.377 | ######## | T cells | Cxcl1 |
| ######## | -1.45425 | 0.014 | 0.361 | ######## | T cells | Nbl1 |
| ######## | -1.05291 | 0.024 | 0.377 | ######## | T cells | Tcn2 |
| ######## | -1.33362 | 0.161 | 0.517 | ######## | T cells | Serpinb6a |
| ######## | -1.18188 | 0.01 | 0.351 | ######## | T cells | Col5a3 |
| ######## | -1.30012 | 0.011 | 0.353 | ######## | T cells | Gsta3 |

| | | | | | | |
|---|---|---|---|---|---|---|
| ######### | -2.97039 | 0.038 | 0.376 | ######### | T cells | Fcer1g |
| ######### | -1.64411 | 0.469 | 0.839 | ######### | T cells | S100a11 |
| ######### | 1.265286 | 0.75 | 0.545 | ######### | T cells | Rpl13a |
| ######### | -1.1388 | 0.012 | 0.347 | ######### | T cells | Sfrp1 |
| ######### | -1.05494 | 0.006 | 0.34 | ######### | T cells | Scara5 |
| ######### | -1.05044 | 0.369 | 0.649 | ######### | T cells | Bsg |
| ######### | -1.51044 | 0.015 | 0.349 | ######### | T cells | Emilin2 |
| ######### | -1.07533 | 0.096 | 0.45 | ######### | T cells | Slc29a1 |
| ######### | -1.59298 | 0.077 | 0.427 | ######### | T cells | Trib1 |
| ######### | -1.05672 | 0.041 | 0.387 | ######### | T cells | Angptl2 |
| ######### | -1.09811 | 0.011 | 0.344 | ######### | T cells | Fxyd6 |
| ######### | -1.20041 | 0.49 | 0.81 | ######### | T cells | Txn1 |
| ######### | -3.80873 | 0.066 | 0.393 | ######### | T cells | Tyrobp |
| ######### | -1.11495 | 0.126 | 0.491 | ######### | T cells | Camk2n1 |
| ######### | -2.18281 | 0.02 | 0.351 | ######### | T cells | Cyr61 |
| ######### | -3.23865 | 0.172 | 0.507 | ######### | T cells | Msrb1 |
| ######### | -1.12378 | 0.011 | 0.33 | ######### | T cells | Fibin |
| ######### | -1.02627 | 0.128 | 0.492 | ######### | T cells | Creg1 |
| ######### | -1.37298 | 0.231 | 0.594 | ######### | T cells | Zyx |
| ######### | -1.12881 | 0.316 | 0.676 | ######### | T cells | Rab7 |
| ######### | -1.1456 | 0.011 | 0.326 | ######### | T cells | Adamts1 |
| ######### | -1.39125 | 0.328 | 0.683 | ######### | T cells | Prdx5 |
| ######### | -1.04683 | 0.012 | 0.323 | ######### | T cells | Gas6 |
| ######### | -1.21139 | 0.32 | 0.597 | ######### | T cells | Cyb5a |
| ######### | -1.10694 | 0.006 | 0.305 | ######### | T cells | Fmo2 |
| ######### | -1.08918 | 0.008 | 0.302 | ######### | T cells | Sirpa |
| ######### | -1.18038 | 0.015 | 0.313 | ######### | T cells | Tppp3 |
| ######### | -2.61457 | 0.027 | 0.32 | ######### | T cells | Alox5ap |
| ######### | 1.003822 | 0.546 | 0.329 | ######### | T cells | Atp1b3 |
| ######### | -1.25518 | 0.074 | 0.388 | ######### | T cells | Snap23 |
| ######### | -1.32233 | 0.011 | 0.299 | ######### | T cells | Ackr3 |
| ######### | -1.38925 | 0.27 | 0.559 | ######### | T cells | Fosb |
| ######### | -1.13323 | 0.184 | 0.518 | ######### | T cells | Ndel1 |
| ######### | -1.46686 | 0.076 | 0.379 | ######### | T cells | Gadd45g |
| ######### | 1.213994 | 0.45 | 0.232 | ######### | T cells | Bcl2 |
| ######### | -1.6601 | 0.019 | 0.301 | ######### | T cells | Ctgf |
| ######### | -2.16465 | 0.016 | 0.293 | ######### | T cells | Ncf2 |
| ######### | -1.27358 | 0.018 | 0.298 | ######### | T cells | Tgm2 |
| ######### | -1.04096 | 0.176 | 0.507 | ######### | T cells | Ehd1 |
| ######### | -1.07215 | 0.052 | 0.349 | ######### | T cells | Bag3 |
| ######### | -1.72304 | 0.034 | 0.315 | ######### | T cells | Tgfbi |
| ######### | -1.20998 | 0.102 | 0.403 | ######### | T cells | Uap1 |
| ######### | -1.05184 | 0.008 | 0.275 | ######### | T cells | Pgf |
| ######### | -1.90962 | 0.011 | 0.276 | ######### | T cells | Spi1 |
| ######### | 1.007295 | 0.325 | 0.116 | ######### | T cells | Bcl2a1b |
| ######### | -2.53404 | 0.015 | 0.278 | ######### | T cells | Csf3r |
| ######### | -1.447 | 0.188 | 0.485 | ######### | T cells | Ly6a |
| ######### | -1.07887 | 0.642 | 0.777 | ######### | T cells | Vim |
| ######### | -2.3943 | 0.052 | 0.322 | ######### | T cells | Lst1 |
| ######### | -1.18898 | 0.019 | 0.286 | ######### | T cells | Hmox1 |
| ######### | -2.7454 | 0.021 | 0.282 | ######### | T cells | Hp |
| ######### | -2.32133 | 0.056 | 0.326 | ######### | T cells | Slc16a3 |
| ######### | -1.00706 | 0.018 | 0.28 | ######### | T cells | Mafb |
| ######### | -2.91251 | 0.039 | 0.301 | ######### | T cells | Ccl6 |

| | | | | | | |
|---|---|---|---|---|---|---|
| ######## | -2.33994 | 0.012 | 0.266 | ######## | T cells | Clec4d |
| ######## | -1.71714 | 0.313 | 0.546 | ######## | T cells | Socs3 |
| ######## | -1.44802 | 0.054 | 0.327 | ######## | T cells | Mt2 |
| ######## | -1.13306 | 0.012 | 0.267 | ######## | T cells | Tnfaip6 |
| ######## | 1.098713 | 0.355 | 0.163 | ######## | T cells | Ass1 |
| ######## | -2.79143 | 0.019 | 0.27 | ######## | T cells | Hdc |
| ######## | -1.14403 | 0.013 | 0.262 | ######## | T cells | Igfbp6 |
| ######## | -2.01877 | 0.077 | 0.341 | ######## | T cells | Plaur |
| ######## | -3.50613 | 0.041 | 0.29 | ######## | T cells | Il1b |
| ######## | -1.16427 | 0.052 | 0.317 | ######## | T cells | Kctd12 |
| ######## | 1.679255 | 0.391 | 0.193 | ######## | T cells | S100a4 |
| ######## | -1.09436 | 0.307 | 0.57 | ######## | T cells | Igfbp4 |
| ######## | -1.80555 | 0.015 | 0.258 | ######## | T cells | Mfap4 |
| ######## | -1.99937 | 0.024 | 0.264 | ######## | T cells | Ccr1 |
| ######## | -3.0076 | 0.04 | 0.277 | ######## | T cells | Il1r2 |
| ######## | -1.62639 | 0.41 | 0.68 | ######## | T cells | Taldo1 |
| ######## | -1.97232 | 0.03 | 0.264 | ######## | T cells | Ifitm1 |
| ######## | -2.8575 | 0.055 | 0.289 | ######## | T cells | Ccrl2 |
| ######## | -1.1661 | 0.099 | 0.357 | ######## | T cells | Ugdh |
| ######## | -1.05709 | 0.056 | 0.296 | ######## | T cells | Ly6c1 |
| ######## | -4.25942 | 0.107 | 0.335 | ######## | T cells | Lyz2 |
| ######## | -1.05265 | 0.135 | 0.392 | ######## | T cells | Hsd11b1 |
| ######## | -1.18633 | 0.095 | 0.343 | ######## | T cells | Tmcc1 |
| ######## | -1.43997 | 0.065 | 0.292 | ######## | T cells | Tpd52 |
| ######## | -1.16207 | 0.137 | 0.386 | ######## | T cells | Nudt4 |
| ######## | -1.09919 | 0.55 | 0.649 | ######## | T cells | Tuba1a |
| ######## | -6.14933 | 0.273 | 0.492 | ######## | T cells | S100a8 |
| ######## | -1.04706 | 0.214 | 0.46 | ######## | T cells | Pnp |
| ######## | 1.186522 | 0.586 | 0.435 | ######## | T cells | AW112010 |
| ######## | -5.26821 | 0.141 | 0.352 | ######## | T cells | Retnlg |
| ######## | -1.22461 | 0.097 | 0.322 | ######## | T cells | Ninj1 |
| ######## | -6.39315 | 0.317 | 0.526 | ######## | T cells | S100a9 |
| ######## | -1.04153 | 0.153 | 0.39 | ######## | T cells | Fgl2 |
| 2.46E-99 | -1.1562 | 0.43 | 0.609 | 4.43E-95 | T cells | Hspa1b |
| 7.40E-94 | -1.53231 | 0.162 | 0.362 | 1.33E-89 | T cells | Rnf149 |
| 5.12E-92 | -1.3364 | 0.069 | 0.251 | 9.22E-88 | T cells | Lilr4b |
| 1.03E-91 | -1.43489 | 0.149 | 0.346 | 1.85E-87 | T cells | Ets2 |
| 1.05E-85 | -1.36805 | 0.085 | 0.263 | 1.89E-81 | T cells | Stx11 |
| 1.16E-58 | -1.70689 | 0.166 | 0.297 | 2.10E-54 | T cells | Lmnb1 |
| 6.12E-42 | -1.04243 | 0.214 | 0.332 | 1.10E-37 | T cells | Cd44 |
| 1.43E-38 | -1.40741 | 0.197 | 0.295 | 2.58E-34 | T cells | Pglyrp1 |
| 1.83E-27 | -1.85557 | 0.312 | 0.367 | 3.29E-23 | T cells | Mxd1 |
| 1.38E-26 | -1.11576 | 0.179 | 0.279 | 2.49E-22 | T cells | Isg15 |
| 2.11E-09 | -2.39464 | 0.671 | 0.669 | 3.80E-05 | T cells | Hbb-bs |
| 0 | 5.325862 | 0.49 | 0.015 | 0 | Monocytes | Ccl24 |
| 0 | 4.039436 | 0.809 | 0.08 | 0 | Monocytes | Plac8 |
| 0 | 3.754968 | 0.935 | 0.088 | 0 | Monocytes | Ctss |
| 0 | 3.739459 | 0.823 | 0.024 | 0 | Monocytes | Ccl9 |
| 0 | 3.553024 | 0.336 | 0.007 | 0 | Monocytes | Arg1 |
| 0 | 3.322264 | 0.909 | 0.255 | 0 | Monocytes | Tgfbi |
| 0 | 3.134635 | 0.734 | 0.031 | 0 | Monocytes | Fcgr2b |
| 0 | 3.103267 | 0.406 | 0.054 | 0 | Monocytes | H2-Ab1 |
| 0 | 3.073171 | 0.943 | 0.276 | 0 | Monocytes | Lgals3 |
| 0 | 3.033736 | 0.467 | 0.01 | 0 | Monocytes | Chil3 |

| | | | | | | |
|---|---|---|---|---|---|---|
| 0 | 2.974895 | 0.807 | 0.025 | 0 | Monocytes | Ms4a6c |
| 0 | 2.959182 | 0.736 | 0.004 | 0 | Monocytes | Ms4a6d |
| 0 | 2.943074 | 0.433 | 0.057 | 0 | Monocytes | H2-Aa |
| 0 | 2.870181 | 0.705 | 0.165 | 0 | Monocytes | Fn1 |
| 0 | 2.850571 | 0.617 | 0.09 | 0 | Monocytes | Cd74 |
| 0 | 2.766497 | 0.621 | 0.006 | 0 | Monocytes | Gatm |
| 0 | 2.752429 | 0.909 | 0.281 | 0 | Monocytes | Lyz2 |
| 0 | 2.640781 | 0.447 | 0.068 | 0 | Monocytes | Ccl2 |
| 0 | 2.586441 | 0.892 | 0.184 | 0 | Monocytes | S100a4 |
| 0 | 2.371573 | 0.737 | 0.058 | 0 | Monocytes | Ccr2 |
| 0 | 2.36191 | 0.99 | 0.794 | 0 | Monocytes | Psap |
| 0 | 2.352148 | 0.712 | 0.092 | 0 | Monocytes | Ctsc |
| 0 | 2.241866 | 0.519 | 0.019 | 0 | Monocytes | H2-DMb1 |
| 0 | 2.16606 | 0.661 | 0.007 | 0 | Monocytes | Pld4 |
| 0 | 2.126497 | 0.695 | 0.05 | 0 | Monocytes | Wfdc17 |
| 0 | 2.083702 | 0.917 | 0.615 | 0 | Monocytes | Rbm3 |
| 0 | 2.01572 | 0.581 | 0.046 | 0 | Monocytes | H2-DMa |
| 0 | 2.004632 | 0.329 | 0.002 | 0 | Monocytes | Mrc1 |
| 0 | 2.003896 | 0.542 | 0.109 | 0 | Monocytes | Ly6c2 |
| 0 | 1.939564 | 0.452 | 0.002 | 0 | Monocytes | F13a1 |
| 0 | 1.906394 | 0.945 | 0.675 | 0 | Monocytes | Gpx1 |
| 0 | 1.882045 | 0.498 | 0.003 | 0 | Monocytes | F10 |
| 0 | 1.879738 | 0.659 | 0.12 | 0 | Monocytes | Ctsh |
| 0 | 1.872053 | 0.503 | 0.003 | 0 | Monocytes | Aif1 |
| 0 | 1.781763 | 0.661 | 0.083 | 0 | Monocytes | Napsa |
| 0 | 1.776008 | 0.596 | 0.08 | 0 | Monocytes | Cybb |
| 0 | 1.754171 | 0.605 | 0.086 | 0 | Monocytes | Cfp |
| 0 | 1.727696 | 0.311 | 0.005 | 0 | Monocytes | Cdh1 |
| 0 | 1.682083 | 0.614 | 0.065 | 0 | Monocytes | Csf1r |
| 0 | 1.660508 | 0.927 | 0.31 | 0 | Monocytes | Fcer1g |
| 0 | 1.648492 | 0.569 | 0.055 | 0 | Monocytes | Mpeg1 |
| 0 | 1.642724 | 0.998 | 0.871 | 0 | Monocytes | Rpl37a |
| 0 | 1.61685 | 0.575 | 0.03 | 0 | Monocytes | Plekho1 |
| 0 | 1.608394 | 0.607 | 0.078 | 0 | Monocytes | Cd68 |
| 0 | 1.595757 | 0.996 | 0.79 | 0 | Monocytes | Rps28 |
| 0 | 1.583206 | 0.594 | 0.073 | 0 | Monocytes | Plbd1 |
| 0 | 1.572448 | 0.456 | 0.092 | 0 | Monocytes | Casp6 |
| 0 | 1.549045 | 0.595 | 0.141 | 0 | Monocytes | Pycard |
| 0 | 1.544623 | 0.466 | 0.004 | 0 | Monocytes | Clec4a1 |
| 0 | 1.526389 | 0.482 | 0.049 | 0 | Monocytes | Ltb4r1 |
| 0 | 1.525471 | 1 | 0.944 | 0 | Monocytes | Rpl41 |
| 0 | 1.518985 | 0.655 | 0.186 | 0 | Monocytes | Fam96a |
| 0 | 1.46571 | 0.546 | 0.059 | 0 | Monocytes | Irf5 |
| 0 | 1.436955 | 0.973 | 0.811 | 0 | Monocytes | Cox4i1 |
| 0 | 1.424713 | 0.452 | 0.014 | 0 | Monocytes | Ly86 |
| 0 | 1.415909 | 0.747 | 0.198 | 0 | Monocytes | Fcgr3 |
| 0 | 1.400148 | 0.508 | 0.074 | 0 | Monocytes | Ifitm6 |
| 0 | 1.380197 | 0.334 | 0.035 | 0 | Monocytes | Gngt2 |
| 0 | 1.369103 | 0.893 | 0.259 | 0 | Monocytes | Alox5ap |
| 0 | 1.359548 | 0.408 | 0.006 | 0 | Monocytes | Clec4a3 |
| 0 | 1.344341 | 0.545 | 0.113 | 0 | Monocytes | Rpl29 |
| 0 | 1.336634 | 0.385 | 0.015 | 0 | Monocytes | Ms4a4c |
| 0 | 1.269778 | 0.342 | 0.001 | 0 | Monocytes | Msr1 |
| 0 | 1.261177 | 0.587 | 0.121 | 0 | Monocytes | Itgam |

| | | | | | | |
|---|---|---|---|---|---|---|
| 0 | 1.243644 | 0.496 | 0.107 | 0 | Monocytes | Mcub |
| 0 | 1.236558 | 0.347 | 0.008 | 0 | Monocytes | P2ry6 |
| 0 | 1.22516 | 0.384 | 0.039 | 0 | Monocytes | Ccr5 |
| 0 | 1.218931 | 0.274 | 0.001 | 0 | Monocytes | Fcgr1 |
| 0 | 1.171249 | 0.307 | 0.001 | 0 | Monocytes | Trem2 |
| 0 | 1.115527 | 0.293 | 0.008 | 0 | Monocytes | Lair1 |
| 0 | 1.10212 | 0.417 | 0.029 | 0 | Monocytes | Nrros |
| 0 | 1.091362 | 0.478 | 0.07 | 0 | Monocytes | Sirpb1b |
| 0 | 1.074101 | 0.469 | 0.061 | 0 | Monocytes | Sirpb1c |
| 0 | 1.063617 | 0.259 | 0.003 | 0 | Monocytes | Batf3 |
| 0 | 1.044504 | 0.997 | 0.986 | 0 | Monocytes | Rps9 |
| 0 | 1.0295 | 0.338 | 0.004 | 0 | Monocytes | Rassf4 |
| 0 | 1.024591 | 0.322 | 0.005 | 0 | Monocytes | Adgre1 |
| ######## | 1.480032 | 0.836 | 0.311 | ######## | Monocytes | Fam49b |
| ######## | 1.202241 | 0.996 | 0.935 | ######## | Monocytes | Rps29 |
| ######## | 1.577736 | 0.923 | 0.676 | ######## | Monocytes | Npc2 |
| ######## | 2.322149 | 0.661 | 0.231 | ######## | Monocytes | Mafb |
| ######## | 1.044902 | 0.526 | 0.109 | ######## | Monocytes | Cyth4 |
| ######## | 1.438124 | 0.572 | 0.142 | ######## | Monocytes | Trf |
| ######## | 1.552272 | 0.977 | 0.763 | ######## | Monocytes | Rps18 |
| ######## | 1.730475 | 0.748 | 0.308 | ######## | Monocytes | Aprt |
| ######## | 1.128477 | 0.481 | 0.093 | ######## | Monocytes | Clec4a2 |
| ######## | -1.0617 | 0.996 | 0.996 | ######## | Monocytes | Malat1 |
| ######## | 1.396962 | 0.961 | 0.797 | ######## | Monocytes | Atp5l |
| ######## | 1.061513 | 0.385 | 0.063 | ######## | Monocytes | Ifi209 |
| ######## | 1.594192 | 0.655 | 0.228 | ######## | Monocytes | Nme2 |
| ######## | 1.583343 | 0.956 | 0.751 | ######## | Monocytes | Rpl35 |
| ######## | 1.326628 | 0.936 | 0.771 | ######## | Monocytes | Sem1 |
| ######## | 1.609199 | 0.92 | 0.656 | ######## | Monocytes | Sec61g |
| ######## | 2.241062 | 0.626 | 0.223 | ######## | Monocytes | Lgmn |
| ######## | 1.147321 | 0.964 | 0.904 | ######## | Monocytes | Cfl1 |
| ######## | 1.421496 | 0.908 | 0.639 | ######## | Monocytes | Rpl36al |
| ######## | 1.345009 | 0.961 | 0.768 | ######## | Monocytes | Rpl23a |
| ######## | 1.403523 | 0.93 | 0.682 | ######## | Monocytes | Rps17 |
| ######## | 1.125797 | 0.995 | 0.846 | ######## | Monocytes | Rplp2 |
| ######## | 1.858838 | 0.867 | 0.577 | ######## | Monocytes | Uba52 |
| ######## | 1.504832 | 0.852 | 0.557 | ######## | Monocytes | Atox1 |
| ######## | 1.499731 | 0.85 | 0.563 | ######## | Monocytes | Slc25a5 |
| ######## | 1.137263 | 0.964 | 0.854 | ######## | Monocytes | Rpl10 |
| ######## | 1.279272 | 0.397 | 0.089 | ######## | Monocytes | Fam129b |
| ######## | 1.252545 | 0.879 | 0.476 | ######## | Monocytes | Ucp2 |
| ######## | 1.63781 | 0.798 | 0.439 | ######## | Monocytes | Eno1 |
| ######## | 1.525256 | 0.862 | 0.554 | ######## | Monocytes | Rpl13a |
| ######## | 1.002333 | 0.612 | 0.175 | ######## | Monocytes | Lyn |
| ######## | 1.249698 | 0.792 | 0.297 | ######## | Monocytes | Cd44 |
| ######## | 1.427377 | 0.707 | 0.31 | ######## | Monocytes | Gm2a |
| ######## | 1.544086 | 0.586 | 0.22 | ######## | Monocytes | Hebp1 |
| ######## | 1.704699 | 0.789 | 0.446 | ######## | Monocytes | Mrpl52 |
| ######## | 1.293237 | 0.948 | 0.778 | ######## | Monocytes | Rps6 |
| ######## | 1.283937 | 0.883 | 0.579 | ######## | Monocytes | Sub1 |
| ######## | 1.200268 | 0.934 | 0.804 | ######## | Monocytes | Cyba |
| ######## | 1.334538 | 0.619 | 0.229 | ######## | Monocytes | Unc93b1 |
| ######## | 1.265233 | 0.439 | 0.107 | ######## | Monocytes | Dok2 |
| ######## | 1.368819 | 0.815 | 0.498 | ######## | Monocytes | Capza2 |

| | | | | | | |
|---|---|---|---|---|---|---|
| ######## | 1.484811 | 0.669 | 0.288 | ######## | Monocytes | Snx5 |
| ######## | 1.271273 | 0.886 | 0.653 | ######## | Monocytes | Sec61b |
| ######## | 1.736641 | 0.735 | 0.383 | ######## | Monocytes | Cstb |
| ######## | 1.268147 | 0.594 | 0.216 | ######## | Monocytes | Metrnl |
| ######## | 1.255875 | 0.866 | 0.562 | ######## | Monocytes | Ctsz |
| ######## | 1.08029 | 0.917 | 0.806 | ######## | Monocytes | Arpc2 |
| ######## | 1.306132 | 0.814 | 0.508 | ######## | Monocytes | Eif4a1 |
| ######## | 1.30391 | 0.844 | 0.564 | ######## | Monocytes | 2010107E04Rik |
| ######## | 1.645958 | 0.748 | 0.372 | ######## | Monocytes | Id2 |
| ######## | 1.017024 | 0.369 | 0.088 | ######## | Monocytes | Arrdc4 |
| ######## | 1.51553 | 0.87 | 0.598 | ######## | Monocytes | Ctsb |
| ######## | 1.588351 | 0.912 | 0.663 | ######## | Monocytes | Crip1 |
| ######## | 1.186391 | 0.497 | 0.154 | ######## | Monocytes | Hopx |
| ######## | 1.432462 | 0.462 | 0.154 | ######## | Monocytes | Naaa |
| ######## | -2.33016 | 0.077 | 0.557 | ######## | Monocytes | Zbtb20 |
| ######## | -3.53296 | 0.078 | 0.553 | ######## | Monocytes | Sparcl1 |
| ######## | -1.88398 | 0.238 | 0.663 | ######## | Monocytes | Timp2 |
| ######## | 1.340235 | 0.756 | 0.448 | ######## | Monocytes | Cops9 |
| ######## | 1.227658 | 0.825 | 0.575 | ######## | Monocytes | Psma7 |
| ######## | -3.86635 | 0.1 | 0.562 | ######## | Monocytes | Bgn |
| ######## | 1.201333 | 0.862 | 0.675 | ######## | Monocytes | Clta |
| ######## | -2.48469 | 0.094 | 0.563 | ######## | Monocytes | Crispld2 |
| ######## | -2.25737 | 0.286 | 0.655 | ######## | Monocytes | Tuba1a |
| ######## | 1.185034 | 0.77 | 0.447 | ######## | Monocytes | Gm11808 |
| ######## | -3.24594 | 0.061 | 0.535 | ######## | Monocytes | Serping1 |
| ######## | -1.43121 | 0.432 | 0.83 | ######## | Monocytes | Pnrc1 |
| ######## | -2.83786 | 0.058 | 0.535 | ######## | Monocytes | Ltbp4 |
| ######## | -2.65034 | 0.04 | 0.523 | ######## | Monocytes | Cygb |
| ######## | 2.281794 | 0.265 | 0.049 | ######## | Monocytes | H2-Eb1 |
| ######## | 1.264722 | 0.797 | 0.487 | ######## | Monocytes | Rps27l |
| ######## | -3.15561 | 0.046 | 0.524 | ######## | Monocytes | Col1a2 |
| ######## | 1.041595 | 0.875 | 0.67 | ######## | Monocytes | Cox6b1 |
| ######## | 1.025573 | 0.901 | 0.678 | ######## | Monocytes | Cox7c |
| ######## | -3.7933 | 0.089 | 0.547 | ######## | Monocytes | Sparc |
| ######## | -3.08684 | 0.027 | 0.51 | ######## | Monocytes | Col1a1 |
| ######## | 1.31348 | 0.682 | 0.361 | ######## | Monocytes | Tmem256 |
| ######## | -3.2345 | 0.082 | 0.541 | ######## | Monocytes | Lum |
| ######## | 1.147086 | 0.751 | 0.449 | ######## | Monocytes | Pitpna |
| ######## | 1.017523 | 0.721 | 0.341 | ######## | Monocytes | Efhd2 |
| ######## | -3.13286 | 0.039 | 0.51 | ######## | Monocytes | Serpinh1 |
| ######## | -2.83615 | 0.062 | 0.522 | ######## | Monocytes | Pcolce |
| ######## | -1.35699 | 0.847 | 0.943 | ######## | Monocytes | Jund |
| ######## | -2.41179 | 0.034 | 0.504 | ######## | Monocytes | Mmp2 |
| ######## | 1.007965 | 0.802 | 0.464 | ######## | Monocytes | Vamp8 |
| ######## | -3.4499 | 0.085 | 0.533 | ######## | Monocytes | Clec3b |
| ######## | -2.08451 | 0.164 | 0.622 | ######## | Monocytes | Cd9 |
| ######## | 1.257131 | 0.637 | 0.271 | ######## | Monocytes | Prkcd |
| ######## | 1.220309 | 0.753 | 0.455 | ######## | Monocytes | Usmg5 |
| ######## | -3.47033 | 0.129 | 0.552 | ######## | Monocytes | Htra3 |
| ######## | 1.075263 | 0.855 | 0.611 | ######## | Monocytes | H2afj |
| ######## | -2.02266 | 0.05 | 0.508 | ######## | Monocytes | Lhfp |
| ######## | -3.85392 | 0.211 | 0.588 | ######## | Monocytes | Igfbp7 |
| ######## | -2.43177 | 0.052 | 0.507 | ######## | Monocytes | Cd34 |
| ######## | -3.33367 | 0.075 | 0.522 | ######## | Monocytes | Col3a1 |

| | | | | | | |
|---|---|---|---|---|---|---|
| ######### | -2.48737 | 0.043 | 0.497 | ######### | Monocytes | Col6a1 |
| ######### | -4.89508 | 0.362 | 0.655 | ######### | Monocytes | Dcn |
| ######### | -2.94568 | 0.052 | 0.503 | ######### | Monocytes | Fbln1 |
| ######### | -2.2456 | 0.036 | 0.487 | ######### | Monocytes | Fstl1 |
| ######### | -2.12144 | 0.046 | 0.493 | ######### | Monocytes | Dpt |
| ######### | -1.16822 | 0.448 | 0.757 | ######### | Monocytes | Aes |
| ######### | -2.12577 | 0.029 | 0.479 | ######### | Monocytes | Nid1 |
| ######### | -1.63778 | 0.019 | 0.47 | ######### | Monocytes | Camk2n1 |
| ######### | 1.054347 | 0.902 | 0.786 | ######### | Monocytes | Pkm |
| ######### | -2.44935 | 0.04 | 0.484 | ######### | Monocytes | Ccdc80 |
| ######### | -2.36386 | 0.039 | 0.485 | ######### | Monocytes | Fbln2 |
| ######### | -2.36256 | 0.041 | 0.485 | ######### | Monocytes | Col6a2 |
| ######### | -2.56354 | 0.033 | 0.48 | ######### | Monocytes | Smoc2 |
| ######### | -1.71013 | 0.018 | 0.467 | ######### | Monocytes | Nfib |
| ######### | 1.076218 | 0.812 | 0.555 | ######### | Monocytes | Uqcrq |
| ######### | -1.60287 | 0.036 | 0.482 | ######### | Monocytes | Nedd4 |
| ######### | -3.20378 | 0.392 | 0.667 | ######### | Monocytes | Cebpd |
| ######### | 1.089161 | 0.848 | 0.595 | ######### | Monocytes | Ndufa2 |
| ######### | -2.11661 | 0.149 | 0.567 | ######### | Monocytes | Cd81 |
| ######### | -2.46939 | 0.163 | 0.557 | ######### | Monocytes | Igfbp4 |
| ######### | -1.76249 | 0.031 | 0.476 | ######### | Monocytes | Sdc2 |
| ######### | -2.17793 | 0.088 | 0.521 | ######### | Monocytes | Rarres2 |
| ######### | -1.74474 | 0.027 | 0.469 | ######### | Monocytes | Mxra8 |
| ######### | -2.35329 | 0.032 | 0.475 | ######### | Monocytes | Col15a1 |
| ######### | 1.681327 | 0.361 | 0.111 | ######### | Monocytes | Dab2 |
| ######### | -1.85392 | 0.093 | 0.514 | ######### | Monocytes | Selenom |
| ######### | 1.008902 | 0.736 | 0.441 | ######### | Monocytes | Brk1 |
| ######### | 1.149511 | 0.67 | 0.364 | ######### | Monocytes | Atp1a1 |
| ######### | -2.32883 | 0.066 | 0.492 | ######### | Monocytes | Serpinf1 |
| ######### | -1.79372 | 0.032 | 0.471 | ######### | Monocytes | Slc43a3 |
| ######### | -1.8485 | 0.237 | 0.617 | ######### | Monocytes | Cd63 |
| ######### | -2.76925 | 0.109 | 0.522 | ######### | Monocytes | Gpx3 |
| ######### | 1.435375 | 0.704 | 0.412 | ######### | Monocytes | Pgk1 |
| ######### | 1.067862 | 0.804 | 0.568 | ######### | Monocytes | Arpc4 |
| ######### | -1.81954 | 0.032 | 0.467 | ######### | Monocytes | Loxl1 |
| ######### | -1.77994 | 0.054 | 0.486 | ######### | Monocytes | Lamc1 |
| ######### | -1.77703 | 0.027 | 0.463 | ######### | Monocytes | Prelp |
| ######### | 1.007415 | 0.82 | 0.619 | ######### | Monocytes | Eif3k |
| ######### | -1.88674 | 0.038 | 0.471 | ######### | Monocytes | Hspg2 |
| ######### | -3.24703 | 0.081 | 0.498 | ######### | Monocytes | Lpl |
| ######### | -1.57966 | 0.03 | 0.459 | ######### | Monocytes | Rhoj |
| ######### | -1.77027 | 0.016 | 0.444 | ######### | Monocytes | Ebf1 |
| ######### | -1.94145 | 0.034 | 0.46 | ######### | Monocytes | Col4a1 |
| ######### | -1.82622 | 0.027 | 0.453 | ######### | Monocytes | Plpp3 |
| ######### | -2.08167 | 0.024 | 0.45 | ######### | Monocytes | Gas1 |
| ######### | 1.042398 | 0.812 | 0.572 | ######### | Monocytes | Cox6a1 |
| ######### | 2.433839 | 0.531 | 0.258 | ######### | Monocytes | Sdc4 |
| ######### | 1.039757 | 0.87 | 0.701 | ######### | Monocytes | Rpl31 |
| ######### | -1.68766 | 0.077 | 0.495 | ######### | Monocytes | Serpinb6a |
| ######### | 1.260348 | 0.824 | 0.686 | ######### | Monocytes | H2afz |
| ######### | -1.20758 | 0.689 | 0.805 | ######### | Monocytes | Gnas |
| ######### | -1.66871 | 0.028 | 0.452 | ######### | Monocytes | Gpm6b |
| ######### | -1.8006 | 0.073 | 0.49 | ######### | Monocytes | Pmp22 |
| ######### | 1.138734 | 0.685 | 0.428 | ######### | Monocytes | Ctsa |

| | | | | | | |
|---|---|---|---|---|---|---|
| ######## | -1.78295 | 0.068 | 0.48 | ######## | Monocytes | Crip2 |
| ######## | -1.81342 | 0.064 | 0.479 | ######## | Monocytes | Rbp1 |
| ######## | -1.83096 | 0.033 | 0.449 | ######## | Monocytes | Pcsk6 |
| ######## | 1.098216 | 0.997 | 0.992 | ######## | Monocytes | Actb |
| ######## | -1.68891 | 0.019 | 0.434 | ######## | Monocytes | Spry1 |
| ######## | -1.81282 | 0.077 | 0.477 | ######## | Monocytes | Nfix |
| ######## | -1.43429 | 0.022 | 0.434 | ######## | Monocytes | Bicc1 |
| ######## | 1.137451 | 0.62 | 0.33 | ######## | Monocytes | Ap2s1 |
| ######## | -1.76203 | 0.027 | 0.439 | ######## | Monocytes | Lamb1 |
| ######## | -1.64753 | 0.05 | 0.464 | ######## | Monocytes | Abca8a |
| ######## | -1.78431 | 0.075 | 0.484 | ######## | Monocytes | Axl |
| ######## | -1.7159 | 0.013 | 0.422 | ######## | Monocytes | Islr |
| ######## | -1.47645 | 0.02 | 0.43 | ######## | Monocytes | Cavin3 |
| ######## | 1.047829 | 0.746 | 0.505 | ######## | Monocytes | Pomp |
| ######## | -1.66094 | 0.05 | 0.459 | ######## | Monocytes | Tnxb |
| ######## | -1.58083 | 0.024 | 0.432 | ######## | Monocytes | Tcf21 |
| ######## | -1.779 | 0.117 | 0.521 | ######## | Monocytes | Pmepa1 |
| ######## | -1.43365 | 0.062 | 0.472 | ######## | Monocytes | Rhoc |
| ######## | -1.58753 | 0.036 | 0.441 | ######## | Monocytes | Olfml3 |
| ######## | -1.54929 | 0.077 | 0.478 | ######## | Monocytes | Rcn3 |
| ######## | -1.41558 | 0.023 | 0.423 | ######## | Monocytes | Lama2 |
| ######## | -2.4973 | 0.042 | 0.44 | ######## | Monocytes | Mfap5 |
| ######## | -1.32012 | 0.037 | 0.442 | ######## | Monocytes | Fxyd1 |
| ######## | -1.4592 | 0.026 | 0.429 | ######## | Monocytes | Ptgis |
| ######## | -1.19955 | 0.156 | 0.556 | ######## | Monocytes | Ifi27 |
| ######## | -1.25354 | 0.617 | 0.858 | ######## | Monocytes | Btg2 |
| ######## | -2.51754 | 0.295 | 0.595 | ######## | Monocytes | Pi16 |
| ######## | -1.48119 | 0.022 | 0.415 | ######## | Monocytes | Ramp2 |
| ######## | -1.37612 | 0.025 | 0.419 | ######## | Monocytes | Itm2a |
| ######## | 1.156677 | 0.489 | 0.218 | ######## | Monocytes | Cndp2 |
| ######## | -1.25947 | 0.017 | 0.41 | ######## | Monocytes | Pdgfra |
| ######## | 1.211401 | 0.679 | 0.412 | ######## | Monocytes | Grn |
| ######## | -1.34712 | 0.036 | 0.426 | ######## | Monocytes | Ddah2 |
| ######## | 1.016811 | 0.675 | 0.378 | ######## | Monocytes | Tgfb1 |
| ######## | -1.73245 | 0.03 | 0.419 | ######## | Monocytes | Ogn |
| ######## | -1.33183 | 0.023 | 0.41 | ######## | Monocytes | Col5a1 |
| ######## | -1.4602 | 0.067 | 0.447 | ######## | Monocytes | Cpq |
| ######## | -1.38755 | 0.039 | 0.428 | ######## | Monocytes | Plxdc2 |
| ######## | -1.22792 | 0.018 | 0.404 | ######## | Monocytes | Cald1 |
| ######## | -1.27644 | 0.02 | 0.407 | ######## | Monocytes | Entpd2 |
| ######## | -1.26908 | 0.023 | 0.409 | ######## | Monocytes | Fhl1 |
| ######## | -2.52197 | 0.24 | 0.56 | ######## | Monocytes | Egr1 |
| ######## | 1.01621 | 0.541 | 0.267 | ######## | Monocytes | Mrpl54 |
| ######## | -3.83294 | 0.307 | 0.581 | ######## | Monocytes | Mgp |
| ######## | -1.13635 | 0.012 | 0.392 | ######## | Monocytes | Fermt2 |
| ######## | -1.29721 | 0.02 | 0.403 | ######## | Monocytes | Adamts2 |
| ######## | -1.17608 | 0.031 | 0.415 | ######## | Monocytes | S100a16 |
| ######## | -1.25308 | 0.03 | 0.41 | ######## | Monocytes | Nfia |
| ######## | -1.13153 | 0.028 | 0.409 | ######## | Monocytes | Fkbp9 |
| ######## | -2.28734 | 0.024 | 0.401 | ######## | Monocytes | Meg3 |
| ######## | -1.52064 | 0.019 | 0.398 | ######## | Monocytes | Adamts5 |
| ######## | -1.33178 | 0.02 | 0.399 | ######## | Monocytes | Fbln5 |
| ######## | -1.25587 | 0.019 | 0.399 | ######## | Monocytes | Col5a2 |
| ######## | 1.110037 | 0.694 | 0.441 | ######## | Monocytes | Atp5k |

| | | | | | | |
|---|---|---|---|---|---|---|
| ######## | -2.37515 | 0.1 | 0.468 | ######## | Monocytes | Ly6a |
| ######## | -2.07701 | 0.14 | 0.495 | ######## | Monocytes | Cfh |
| ######## | -1.15118 | 0.025 | 0.403 | ######## | Monocytes | Rora |
| ######## | 1.01302 | 0.752 | 0.5 | ######## | Monocytes | Uqcr11 |
| ######## | -1.47109 | 0.014 | 0.386 | ######## | Monocytes | Col6a3 |
| ######## | -1.29545 | 0.54 | 0.819 | ######## | Monocytes | Klf2 |
| ######## | -4.42553 | 0.751 | 0.772 | ######## | Monocytes | Gsn |
| ######## | -1.52302 | 0.137 | 0.501 | ######## | Monocytes | Klf9 |
| ######## | -2.06522 | 0.141 | 0.529 | ######## | Monocytes | G0s2 |
| ######## | 1.160671 | 0.503 | 0.241 | ######## | Monocytes | Syngr2 |
| ######## | -1.21409 | 0.013 | 0.38 | ######## | Monocytes | Medag |
| ######## | 1.265385 | 0.637 | 0.385 | ######## | Monocytes | Sys1 |
| ######## | -1.123 | 0.02 | 0.388 | ######## | Monocytes | Mxra7 |
| ######## | -1.16227 | 0.116 | 0.495 | ######## | Monocytes | Sntb2 |
| ######## | 1.083727 | 0.698 | 0.451 | ######## | Monocytes | Srp9 |
| ######## | -1.32101 | 0.022 | 0.384 | ######## | Monocytes | Fbn1 |
| ######## | -1.59886 | 0.024 | 0.387 | ######## | Monocytes | Col8a1 |
| ######## | -1.24072 | 0.025 | 0.387 | ######## | Monocytes | Sod3 |
| ######## | -1.11021 | 0.016 | 0.376 | ######## | Monocytes | Lama4 |
| ######## | -1.39021 | 0.055 | 0.415 | ######## | Monocytes | Oaf |
| ######## | -1.01203 | 0.015 | 0.372 | ######## | Monocytes | P3h3 |
| ######## | -1.47937 | 0.023 | 0.376 | 6.04E-99 | Monocytes | Pcolce2 |
| ######## | -1.12295 | 0.011 | 0.365 | 9.78E-99 | Monocytes | Aebp1 |
| ######## | -1.45557 | 0.232 | 0.542 | 4.29E-98 | Monocytes | Sptbn1 |
| ######## | -1.11627 | 0.051 | 0.409 | 1.67E-97 | Monocytes | Pkd2 |
| ######## | -1.32969 | 0.054 | 0.411 | 1.34E-96 | Monocytes | Pam |
| ######## | -1.04798 | 0.006 | 0.354 | 1.99E-96 | Monocytes | Vwa1 |
| ######## | -1.05676 | 0.011 | 0.357 | 2.78E-96 | Monocytes | Dpysl3 |
| ######## | -2.13978 | 0.053 | 0.403 | 3.76E-96 | Monocytes | Dpep1 |
| ######## | 2.344463 | 0.276 | 0.081 | 6.17E-96 | Monocytes | Ccl7 |
| ######## | -1.09368 | 0.142 | 0.5 | 1.06E-95 | Monocytes | Tubb4b |
| 1.71E-98 | -1.09703 | 0.385 | 0.724 | 3.07E-94 | Monocytes | Ypel3 |
| 3.24E-98 | -1.56939 | 0.009 | 0.35 | 5.84E-94 | Monocytes | Spon2 |
| 4.54E-98 | 1.122279 | 0.452 | 0.196 | 8.17E-94 | Monocytes | Il4ra |
| 1.89E-97 | -1.5537 | 0.015 | 0.355 | 3.41E-93 | Monocytes | Aspn |
| 2.54E-97 | -1.52228 | 0.031 | 0.379 | 4.57E-93 | Monocytes | Timp3 |
| 7.44E-97 | -1.21843 | 0.013 | 0.351 | 1.34E-92 | Monocytes | Htra1 |
| 9.89E-97 | -1.42278 | 0.041 | 0.387 | 1.78E-92 | Monocytes | Gem |
| 4.42E-96 | -1.07201 | 0.876 | 0.936 | 7.95E-92 | Monocytes | Junb |
| 6.14E-96 | 1.029133 | 0.675 | 0.453 | 1.11E-91 | Monocytes | Rnh1 |
| 3.34E-95 | 1.095395 | 0.658 | 0.419 | 6.02E-91 | Monocytes | Nme1 |
| 1.29E-94 | -1.21019 | 0.027 | 0.366 | 2.32E-90 | Monocytes | Serpine2 |
| 1.32E-94 | 1.024845 | 0.392 | 0.15 | 2.38E-90 | Monocytes | Tmem176b |
| 1.25E-93 | 1.10689 | 0.484 | 0.226 | 2.25E-89 | Monocytes | Hif1a |
| 5.64E-93 | -1.09164 | 0.086 | 0.425 | 1.02E-88 | Monocytes | Slc29a1 |
| 6.74E-93 | -1.32991 | 0.015 | 0.345 | 1.21E-88 | Monocytes | Gfpt2 |
| 1.50E-90 | -1.03088 | 0.048 | 0.389 | 2.71E-86 | Monocytes | Socs2 |
| 3.04E-90 | -1.02305 | 0.017 | 0.341 | 5.47E-86 | Monocytes | Col4a2 |
| 3.15E-90 | 1.027462 | 0.488 | 0.25 | 5.67E-86 | Monocytes | Ap2a2 |
| 3.38E-90 | -1.0389 | 0.07 | 0.406 | 6.08E-86 | Monocytes | Selenon |
| 6.25E-90 | -2.60979 | 0.098 | 0.429 | 1.12E-85 | Monocytes | Hspb1 |
| 8.74E-90 | -1.40073 | 0.015 | 0.336 | 1.57E-85 | Monocytes | Nbl1 |
| 8.74E-90 | -1.16315 | 0.601 | 0.846 | 1.57E-85 | Monocytes | Map1lc3b |
| 2.13E-89 | -1.27233 | 0.018 | 0.34 | 3.84E-85 | Monocytes | Eln |

| | | | | | | |
|---|---|---|---|---|---|---|
| 3.35E-89 | -1.08235 | 0.037 | 0.363 | 6.03E-85 | Monocytes | Angptl2 |
| 8.11E-89 | -1.15953 | 0.041 | 0.367 | 1.46E-84 | Monocytes | Ppp1r14a |
| 1.39E-88 | 1.05963 | 0.68 | 0.43 | 2.50E-84 | Monocytes | BC005537 |
| 2.98E-88 | -1.11498 | 0.114 | 0.44 | 5.36E-84 | Monocytes | Nfic |
| 4.63E-88 | -1.0707 | 0.032 | 0.357 | 8.34E-84 | Monocytes | Tm4sf1 |
| 5.33E-88 | -1.56883 | 0.236 | 0.54 | 9.60E-84 | Monocytes | Fosb |
| 6.58E-88 | -1.12871 | 0.012 | 0.327 | 1.18E-83 | Monocytes | Col5a3 |
| 6.65E-87 | -1.2239 | 0.014 | 0.328 | 1.20E-82 | Monocytes | Gsta3 |
| 3.09E-86 | -1.09232 | 0.013 | 0.323 | 5.56E-82 | Monocytes | Sfrp1 |
| 3.57E-86 | -1.24524 | 0.197 | 0.505 | 6.42E-82 | Monocytes | Rnase4 |
| 5.66E-86 | -1.11892 | 0.157 | 0.492 | 1.02E-81 | Monocytes | Dhrs7 |
| 5.95E-86 | -1.12667 | 0.029 | 0.341 | 1.07E-81 | Monocytes | Pla1a |
| 4.83E-85 | 1.168524 | 0.706 | 0.401 | 8.69E-81 | Monocytes | Apoe |
| 2.34E-84 | -2.33409 | 0.06 | 0.371 | 4.21E-80 | Monocytes | Gm12840 |
| 2.55E-84 | -1.67153 | 0.125 | 0.429 | 4.60E-80 | Monocytes | Ckb |
| 7.03E-83 | -1.06195 | 0.584 | 0.765 | 1.27E-78 | Monocytes | Dnaja1 |
| 1.28E-82 | -1.06609 | 0.077 | 0.387 | 2.31E-78 | Monocytes | Emilin1 |
| 3.38E-82 | 1.065273 | 0.496 | 0.27 | 6.08E-78 | Monocytes | Snx2 |
| 9.30E-82 | -2.09341 | 0.026 | 0.327 | 1.67E-77 | Monocytes | Cyr61 |
| 5.69E-80 | -1.101 | 0.011 | 0.303 | 1.02E-75 | Monocytes | Adamts1 |
| 5.01E-78 | 2.42122 | 0.459 | 0.258 | 9.02E-74 | Monocytes | Tgm2 |
| 1.15E-77 | -1.30385 | 0.077 | 0.376 | 2.08E-73 | Monocytes | Hsd11b1 |
| 2.49E-77 | -1.60273 | 0.29 | 0.52 | 4.48E-73 | Monocytes | Gstm1 |
| 7.88E-77 | -1.20777 | 0.356 | 0.576 | 1.42E-72 | Monocytes | Cyb5a |
| 8.26E-74 | -1.30893 | 0.005 | 0.279 | 1.49E-69 | Monocytes | Ackr3 |
| 4.27E-72 | -1.0962 | 0.253 | 0.499 | 7.69E-68 | Monocytes | Nenf |
| 1.01E-71 | -1.0236 | 0.208 | 0.489 | 1.81E-67 | Monocytes | Tcf4 |
| 2.38E-71 | -1.48066 | 0.041 | 0.318 | 4.29E-67 | Monocytes | Ets1 |
| 2.47E-71 | -1.2658 | 0.349 | 0.547 | 4.45E-67 | Monocytes | Mtch1 |
| 1.74E-70 | -1.92896 | 0.066 | 0.35 | 3.14E-66 | Monocytes | Cxcl1 |
| 3.33E-69 | -1.20317 | 0.113 | 0.395 | 5.99E-65 | Monocytes | Atf5 |
| 8.63E-68 | -1.00767 | 0.296 | 0.536 | 1.55E-63 | Monocytes | Ybx3 |
| 6.67E-65 | -1.51601 | 0.025 | 0.28 | 1.20E-60 | Monocytes | Ctgf |
| 1.04E-63 | 1.903478 | 0.353 | 0.174 | 1.88E-59 | Monocytes | Timp1 |
| 3.71E-63 | -1.01706 | 0.542 | 0.639 | 6.68E-59 | Monocytes | Aldh2 |
| 2.17E-62 | 1.249896 | 0.374 | 0.186 | 3.90E-58 | Monocytes | Id1 |
| 5.42E-62 | -1.54148 | 0.034 | 0.281 | 9.75E-58 | Monocytes | Cd3e |
| 8.50E-62 | 1.370492 | 0.458 | 0.247 | 1.53E-57 | Monocytes | Hmox1 |
| 3.56E-59 | -1.01068 | 0.032 | 0.277 | 6.40E-55 | Monocytes | Thy1 |
| 8.46E-54 | -1.20635 | 0.1 | 0.339 | 1.52E-49 | Monocytes | Ugdh |
| 1.63E-51 | -1.13738 | 0.231 | 0.452 | 2.94E-47 | Monocytes | Ecm1 |
| 6.43E-50 | 1.024259 | 0.508 | 0.335 | 1.16E-45 | Monocytes | Cltc |
| 1.64E-49 | 1.147709 | 0.585 | 0.427 | 2.95E-45 | Monocytes | Mif |
| 2.06E-48 | -1.15973 | 0.138 | 0.368 | 3.72E-44 | Monocytes | Nudt4 |
| 1.32E-47 | -1.04323 | 0.25 | 0.48 | 2.38E-43 | Monocytes | Id3 |
| 4.17E-47 | -1.02908 | 0.157 | 0.379 | 7.50E-43 | Monocytes | Uap1 |
| 4.30E-41 | -1.21551 | 0.333 | 0.528 | 7.74E-37 | Monocytes | Dnajb1 |
| 1.03E-38 | -1.07103 | 0.182 | 0.386 | 1.86E-34 | Monocytes | 2810474O19Rik |
| 3.22E-35 | -1.79571 | 0.192 | 0.358 | 5.80E-31 | Monocytes | Vps37b |
| 6.89E-32 | -1.31549 | 0.392 | 0.571 | 1.24E-27 | Monocytes | Grina |
| 1.06E-30 | -1.22627 | 0.731 | 0.778 | 1.90E-26 | Monocytes | Fos |
| 3.82E-28 | -2.50214 | 0.803 | 0.663 | 6.88E-24 | Monocytes | Hbb-bs |
| 2.57E-26 | -1.05447 | 0.228 | 0.398 | 4.62E-22 | Monocytes | Thbs1 |
| 3.71E-22 | -3.05919 | 0.71 | 0.456 | 6.69E-18 | Monocytes | S100a8 |

| | | | | | | |
|---|---|---|---|---|---|---|
| 3.73E-22 | -1.63883 | 0.17 | 0.289 | 6.72E-18 | Monocytes | Pglyrp1 |
| 3.62E-21 | -2.34846 | 0.635 | 0.494 | 6.52E-17 | Monocytes | Hba-a1 |
| 6.38E-18 | -1.8646 | 0.275 | 0.365 | 1.15E-13 | Monocytes | Mxd1 |
| 1.05E-15 | -1.09694 | 0.204 | 0.29 | 1.89E-11 | Monocytes | Ltb |
| 2.29E-15 | -1.19779 | 0.163 | 0.273 | 4.12E-11 | Monocytes | Isg15 |
| 5.50E-13 | -1.22832 | 0.223 | 0.292 | 9.89E-09 | Monocytes | Samsn1 |
| 2.62E-11 | -2.21301 | 0.191 | 0.253 | 4.72E-07 | Monocytes | Il1r2 |
| 9.53E-11 | -1.49872 | 0.227 | 0.285 | 1.72E-06 | Monocytes | Lmnb1 |
| 0 | 5.269303 | 0.609 | 0.007 | 0 | ILC | Gzma |
| 0 | 3.96232 | 0.877 | 0.087 | 0 | ILC | Ccl5 |
| 0 | 3.626446 | 0.717 | 0.201 | 0 | ILC | Irf8 |
| 0 | 3.494566 | 0.901 | 0.088 | 0 | ILC | Nkg7 |
| 0 | 3.341147 | 0.691 | 0.018 | 0 | ILC | Prf1 |
| 0 | 3.120423 | 0.949 | 0.429 | 0 | ILC | AW112010 |
| 0 | 3.093218 | 0.679 | 0.026 | 0 | ILC | Serpinb6b |
| 0 | 2.952265 | 0.842 | 0.066 | 0 | ILC | Il2rb |
| 0 | 2.812467 | 0.692 | 0.003 | 0 | ILC | Ncr1 |
| 0 | 2.808587 | 0.404 | 0.007 | 0 | ILC | Xcl1 |
| 0 | 2.751081 | 0.767 | 0.029 | 0 | ILC | Klrd1 |
| 0 | 2.656328 | 0.653 | 0.117 | 0 | ILC | Serpinb9 |
| 0 | 2.605837 | 0.745 | 0.156 | 0 | ILC | Ugcg |
| 0 | 2.488341 | 0.596 | 0.044 | 0 | ILC | Ctla2a |
| 0 | 2.448282 | 0.766 | 0.059 | 0 | ILC | Ctsw |
| 0 | 2.400014 | 0.547 | 0.025 | 0 | ILC | Klrc1 |
| 0 | 2.3794 | 0.612 | 0.004 | 0 | ILC | Klre1 |
| 0 | 2.354205 | 0.728 | 0.066 | 0 | ILC | Txk |
| 0 | 2.344169 | 0.494 | 0.039 | 0 | ILC | Gzmb |
| 0 | 2.302625 | 0.552 | 0.028 | 0 | ILC | Cd7 |
| 0 | 2.244881 | 0.502 | 0.006 | 0 | ILC | Eomes |
| 0 | 2.212043 | 0.621 | 0.025 | 0 | ILC | Klrk1 |
| 0 | 2.186182 | 0.669 | 0.146 | 0 | ILC | Nr4a2 |
| 0 | 2.034063 | 0.647 | 0.079 | 0 | ILC | Sh2d2a |
| 0 | 1.972257 | 0.887 | 0.326 | 0 | ILC | Vps37b |
| 0 | 1.952595 | 0.801 | 0.165 | 0 | ILC | 1-Sep |
| 0 | 1.949782 | 0.655 | 0.109 | 0 | ILC | Rgs1 |
| 0 | 1.948775 | 0.734 | 0.169 | 0 | ILC | Dusp2 |
| 0 | 1.941944 | 0.354 | 0.003 | 0 | ILC | Cma1 |
| 0 | 1.86647 | 0.845 | 0.368 | 0 | ILC | Id2 |
| 0 | 1.813667 | 0.333 | 0.003 | 0 | ILC | Trdc |
| 0 | 1.805311 | 0.768 | 0.139 | 0 | ILC | Ms4a4b |
| 0 | 1.797648 | 0.286 | 0.002 | 0 | ILC | Klra3 |
| 0 | 1.785731 | 0.995 | 0.759 | 0 | ILC | Tmsb10 |
| 0 | 1.78077 | 0.657 | 0.097 | 0 | ILC | Dok2 |
| 0 | 1.730154 | 0.432 | 0.002 | 0 | ILC | Klrb1c |
| 0 | 1.697908 | 0.739 | 0.088 | 0 | ILC | Trbc1 |
| 0 | 1.69773 | 0.715 | 0.12 | 0 | ILC | Lck |
| 0 | 1.695521 | 0.466 | 0.068 | 0 | ILC | Stmn1 |
| 0 | 1.675737 | 0.697 | 0.127 | 0 | ILC | Ptprcap |
| 0 | 1.644014 | 0.53 | 0.062 | 0 | ILC | Gimap5 |
| 0 | 1.604139 | 0.449 | 0.029 | 0 | ILC | Fasl |
| 0 | 1.528502 | 0.487 | 0.066 | 0 | ILC | Runx3 |
| 0 | 1.520184 | 0.399 | 0.019 | 0 | ILC | Klrc2 |
| 0 | 1.508413 | 0.811 | 0.282 | 0 | ILC | Ets1 |
| 0 | 1.504929 | 0.425 | 0.025 | 0 | ILC | Tbx21 |

| | | | | | | |
|---|---|---|---|---|---|---|
| 0 | 1.482465 | 0.605 | 0.123 | 0 | ILC | Ptpn22 |
| 0 | 1.448031 | 0.69 | 0.19 | 0 | ILC | Dusp5 |
| 0 | 1.434245 | 0.81 | 0.25 | 0 | ILC | Ptpn18 |
| 0 | 1.410177 | 0.41 | 0.042 | 0 | ILC | Atp1b1 |
| 0 | 1.388321 | 0.532 | 0.098 | 0 | ILC | Gimap4 |
| 0 | 1.346825 | 0.355 | 0.015 | 0 | ILC | Sytl3 |
| 0 | 1.341785 | 0.597 | 0.121 | 0 | ILC | Gimap1 |
| 0 | 1.34118 | 0.562 | 0.103 | 0 | ILC | Skap1 |
| 0 | 1.333893 | 0.27 | 0.007 | 0 | ILC | Klrg1 |
| 0 | 1.328987 | 0.584 | 0.129 | 0 | ILC | B4galnt1 |
| 0 | 1.270882 | 0.439 | 0.064 | 0 | ILC | Stat4 |
| 0 | 1.223855 | 0.322 | 0.004 | 0 | ILC | Klrb1f |
| 0 | 1.192622 | 0.385 | 0.056 | 0 | ILC | Lamb3 |
| 0 | 1.190121 | 0.428 | 0.07 | 0 | ILC | Tnfrsf18 |
| 0 | 1.168428 | 0.351 | 0.034 | 0 | ILC | Il18r1 |
| 0 | 1.145367 | 0.332 | 0.041 | 0 | ILC | Ccr5 |
| 0 | 1.131997 | 0.396 | 0.056 | 0 | ILC | Prkch |
| 0 | 1.119903 | 0.358 | 0.051 | 0 | ILC | Spn |
| 0 | -3.21259 | 0.098 | 0.788 | 0 | ILC | Ifitm2 |
| ######## | -3.62043 | 0.075 | 0.739 | ######## | ILC | Ifitm3 |
| ######## | 1.436621 | 0.924 | 0.374 | ######## | ILC | Selplg |
| ######## | 1.994025 | 0.909 | 0.634 | ######## | ILC | Ifngr1 |
| ######## | 1.10829 | 0.422 | 0.073 | ######## | ILC | Cd48 |
| ######## | 1.287188 | 0.498 | 0.102 | ######## | ILC | Il2rg |
| ######## | 1.203618 | 0.459 | 0.087 | ######## | ILC | Gimap9 |
| ######## | -2.98629 | 0.515 | 0.86 | ######## | ILC | Cst3 |
| ######## | 1.770378 | 0.937 | 0.681 | ######## | ILC | H2afz |
| ######## | 1.825491 | 0.677 | 0.237 | ######## | ILC | Bcl2 |
| ######## | 1.870901 | 0.646 | 0.228 | ######## | ILC | Pik3r1 |
| ######## | 1.750083 | 0.935 | 0.62 | ######## | ILC | Zfp36l2 |
| ######## | 1.318014 | 0.593 | 0.158 | ######## | ILC | Bin2 |
| ######## | 1.394468 | 0.907 | 0.389 | ######## | ILC | Ptprc |
| ######## | 1.126527 | 0.994 | 0.784 | ######## | ILC | Ppia |
| ######## | 1.031551 | 0.995 | 0.848 | ######## | ILC | Rps11 |
| ######## | 1.181318 | 0.565 | 0.137 | ######## | ILC | Cd2 |
| ######## | 1.329291 | 0.972 | 0.711 | ######## | ILC | H2-K1 |
| ######## | -4.35979 | 0.081 | 0.681 | ######## | ILC | Cebpd |
| ######## | 1.261142 | 0.49 | 0.112 | ######## | ILC | Gimap3 |
| ######## | 1.200359 | 0.564 | 0.144 | ######## | ILC | Gimap6 |
| ######## | 1.027393 | 0.399 | 0.073 | ######## | ILC | Ccr2 |
| ######## | 1.172155 | 0.893 | 0.385 | ######## | ILC | Laptm5 |
| ######## | -5.7309 | 0.403 | 0.788 | ######## | ILC | Gsn |
| ######## | 1.16105 | 0.792 | 0.29 | ######## | ILC | Hcst |
| ######## | 1.079003 | 0.376 | 0.072 | ######## | ILC | Il18rap |
| ######## | -2.36264 | 0.083 | 0.66 | ######## | ILC | Aldh2 |
| ######## | 1.084993 | 0.378 | 0.074 | ######## | ILC | S1pr4 |
| ######## | 1.131775 | 0.515 | 0.133 | ######## | ILC | Itgb7 |
| ######## | -2.89365 | 0.057 | 0.626 | ######## | ILC | Cd63 |
| ######## | -1.88241 | 0.495 | 0.882 | ######## | ILC | S100a6 |
| ######## | 1.093179 | 0.467 | 0.115 | ######## | ILC | Tm6sf1 |
| ######## | 1.08158 | 0.964 | 0.44 | ######## | ILC | Rac2 |
| ######## | 1.025743 | 0.592 | 0.176 | ######## | ILC | Satb1 |
| ######## | 1.078034 | 0.965 | 0.785 | ######## | ILC | Rpl27 |
| ######## | -3.21732 | 0.053 | 0.607 | ######## | ILC | Pi16 |

| | | | | | | |
|---|---|---|---|---|---|---|
| ######## | -2.93204 | 0.06 | 0.6 | ######## | ILC | Ctsl |
| ######## | -2.1735 | 0.175 | 0.666 | ######## | ILC | Timp2 |
| ######## | 1.233435 | 0.483 | 0.147 | ######## | ILC | Evl |
| ######## | 1.017314 | 0.407 | 0.093 | ######## | ILC | P2ry10 |
| ######## | 1.380732 | 0.71 | 0.276 | ######## | ILC | Neurl3 |
| ######## | 1.156248 | 0.473 | 0.125 | ######## | ILC | Ccl4 |
| ######## | 1.038951 | 0.993 | 0.926 | ######## | ILC | Pfn1 |
| ######## | 1.105233 | 0.515 | 0.155 | ######## | ILC | Prex1 |
| ######## | -2.74377 | 0.024 | 0.567 | ######## | ILC | Crispld2 |
| ######## | 1.132476 | 0.708 | 0.269 | ######## | ILC | Arhgap45 |
| ######## | -1.90278 | 0.02 | 0.564 | ######## | ILC | App |
| ######## | 1.185418 | 0.862 | 0.58 | ######## | ILC | Sub1 |
| ######## | 1.128083 | 0.941 | 0.656 | ######## | ILC | S100a10 |
| ######## | -2.08959 | 0.02 | 0.567 | ######## | ILC | Mgst1 |
| ######## | -1.33399 | 0.994 | 0.996 | ######## | ILC | Fth1 |
| ######## | 1.166288 | 0.694 | 0.262 | ######## | ILC | Itgb2 |
| ######## | -4.27849 | 0.113 | 0.593 | ######## | ILC | Igfbp7 |
| ######## | 1.147688 | 0.924 | 0.663 | ######## | ILC | Tagln2 |
| ######## | 1.208911 | 0.814 | 0.506 | ######## | ILC | Mbnl1 |
| ######## | 1.60591 | 0.497 | 0.175 | ######## | ILC | Spry2 |
| ######## | -1.89558 | 0.016 | 0.539 | ######## | ILC | Marcks |
| ######## | -3.45103 | 0.027 | 0.537 | ######## | ILC | Serping1 |
| ######## | -2.63245 | 0.094 | 0.602 | ######## | ILC | Ifi27l2a |
| ######## | -2.76754 | 0.065 | 0.562 | ######## | ILC | Igfbp4 |
| ######## | -3.82999 | 0.061 | 0.555 | ######## | ILC | Htra3 |
| ######## | -3.70862 | 0.061 | 0.554 | ######## | ILC | Sparcl1 |
| ######## | 1.053622 | 0.715 | 0.269 | ######## | ILC | Samsn1 |
| ######## | 1.049969 | 0.381 | 0.092 | ######## | ILC | Itga4 |
| ######## | -5.64687 | 0.265 | 0.659 | ######## | ILC | Dcn |
| ######## | -4.67134 | 0.122 | 0.589 | ######## | ILC | Mgp |
| ######## | -3.02299 | 0.02 | 0.525 | ######## | ILC | Pcolce |
| ######## | 1.42409 | 0.521 | 0.189 | ######## | ILC | Crem |
| ######## | -3.95446 | 0.066 | 0.549 | ######## | ILC | Sparc |
| ######## | -4.03844 | 0.093 | 0.563 | ######## | ILC | Bgn |
| ######## | -3.45425 | 0.057 | 0.542 | ######## | ILC | Lum |
| ######## | -1.7154 | 0.09 | 0.598 | ######## | ILC | Ly6e |
| ######## | -2.7814 | 0.027 | 0.524 | ######## | ILC | Cygb |
| ######## | -3.04924 | 0.027 | 0.534 | ######## | ILC | G0s2 |
| ######## | -2.48969 | 0.028 | 0.524 | ######## | ILC | Rarres2 |
| ######## | -2.9767 | 0.049 | 0.535 | ######## | ILC | Ltbp4 |
| ######## | -2.53075 | 0.095 | 0.569 | ######## | ILC | Cd81 |
| ######## | -3.63578 | 0.051 | 0.535 | ######## | ILC | Clec3b |
| ######## | -2.06233 | 0.021 | 0.518 | ######## | ILC | Selenom |
| ######## | -3.26679 | 0.038 | 0.525 | ######## | ILC | Col1a2 |
| ######## | -3.12573 | 0.042 | 0.525 | ######## | ILC | Gpx3 |
| ######## | 1.08026 | 0.59 | 0.219 | ######## | ILC | Tnfaip3 |
| ######## | -2.5555 | 0.021 | 0.508 | ######## | ILC | Cd34 |
| ######## | -2.11449 | 0.208 | 0.624 | ######## | ILC | Anxa5 |
| ######## | -2.04128 | 0.022 | 0.507 | ######## | ILC | Lrp1 |
| ######## | 1.325994 | 0.581 | 0.258 | ######## | ILC | Vgll4 |
| ######## | -3.1202 | 0.034 | 0.511 | ######## | ILC | Serpinh1 |
| ######## | 1.260763 | 0.654 | 0.327 | ######## | ILC | Stk24 |
| ######## | -2.10503 | 0.028 | 0.509 | ######## | ILC | Lhfp |
| ######## | -2.51315 | 0.022 | 0.501 | ######## | ILC | Cfh |

| | | | | | | |
|---|---|---|---|---|---|---|
| ######### | -2.58698 | 0.021 | 0.498 | ######### | ILC | Col6a1 |
| ######### | -2.43064 | 0.03 | 0.504 | ######### | ILC | Mmp2 |
| ######### | -3.07128 | 0.04 | 0.51 | ######### | ILC | Col1a1 |
| ######### | -3.06558 | 0.031 | 0.505 | ######### | ILC | Fbln1 |
| ######### | -2.08673 | 0.014 | 0.493 | ######### | ILC | Pmp22 |
| ######### | -1.84577 | 0.033 | 0.513 | ######### | ILC | Rnase4 |
| ######### | -2.2259 | 0.021 | 0.495 | ######### | ILC | Dpt |
| ######### | -2.47972 | 0.014 | 0.487 | ######### | ILC | Col6a2 |
| ######### | -1.75404 | 0.016 | 0.491 | ######### | ILC | Cd302 |
| ######### | -3.56005 | 0.035 | 0.501 | ######### | ILC | Lpl |
| ######### | -2.42345 | 0.029 | 0.494 | ######### | ILC | Serpinf1 |
| ######### | -2.44629 | 0.02 | 0.486 | ######### | ILC | Fbln2 |
| ######### | -1.83981 | 0.01 | 0.477 | ######### | ILC | Sdc2 |
| ######### | -1.97674 | 0.014 | 0.48 | ######### | ILC | Nfix |
| ######### | -2.27918 | 0.025 | 0.488 | ######### | ILC | Fstl1 |
| ######### | -1.72183 | 0.048 | 0.509 | ######### | ILC | Nenf |
| ######### | -2.02606 | 0.019 | 0.481 | ######### | ILC | Rbp1 |
| ######### | -3.43804 | 0.081 | 0.522 | ######### | ILC | Col3a1 |
| ######### | -2.4592 | 0.029 | 0.485 | ######### | ILC | Ccdc80 |
| ######### | -3.94696 | 0.124 | 0.558 | ######### | ILC | Mt1 |
| ######### | 1.224175 | 0.541 | 0.228 | ######### | ILC | Pdcd4 |
| ######### | -1.73591 | 0.011 | 0.468 | ######### | ILC | Nfib |
| ######### | -1.98188 | 0.032 | 0.486 | ######### | ILC | Axl |
| ######### | -1.36414 | 0.138 | 0.601 | ######### | ILC | Lamp2 |
| ######### | -2.43748 | 0.024 | 0.475 | ######### | ILC | Col15a1 |
| ######### | -1.86385 | 0.017 | 0.472 | ######### | ILC | Slc43a3 |
| ######### | -2.00904 | 0.019 | 0.472 | ######### | ILC | Hspg2 |
| ######### | -2.57832 | 0.029 | 0.48 | ######### | ILC | Smoc2 |
| ######### | -1.85982 | 0.035 | 0.487 | ######### | ILC | Lamc1 |
| ######### | -2.1267 | 0.03 | 0.479 | ######### | ILC | Nid1 |
| ######### | -1.45054 | 0.062 | 0.519 | ######### | ILC | Myadm |
| ######### | -1.88611 | 0.016 | 0.468 | ######### | ILC | Loxl1 |
| ######### | -1.44804 | 0.009 | 0.463 | ######### | ILC | Fcgrt |
| ######### | -1.87737 | 0.013 | 0.464 | ######### | ILC | Prelp |
| ######### | -1.6575 | 0.01 | 0.46 | ######### | ILC | Rhoj |
| ######### | 1.231425 | 0.797 | 0.579 | ######### | ILC | Jak1 |
| ######### | -1.696 | 0.031 | 0.48 | ######### | ILC | Rcn3 |
| ######### | -1.89014 | 0.007 | 0.455 | ######### | ILC | Plpp3 |
| ######### | 1.011489 | 0.851 | 0.684 | ######### | ILC | Shisa5 |
| ######### | -1.77676 | 0.02 | 0.465 | ######### | ILC | Abca8a |
| ######### | -2.3173 | 0.047 | 0.49 | ######### | ILC | Id3 |
| ######### | -1.98905 | 0.016 | 0.461 | ######### | ILC | Col4a1 |
| ######### | -1.74368 | 0.01 | 0.453 | ######### | ILC | Gpm6b |
| ######### | -1.70438 | 0.027 | 0.469 | ######### | ILC | Mxra8 |
| ######### | -1.81123 | 0.018 | 0.461 | ######### | ILC | Tnxb |
| ######### | -1.93294 | 0.011 | 0.45 | ######### | ILC | Pcsk6 |
| ######### | -1.77726 | 0.005 | 0.445 | ######### | ILC | Ebf1 |
| ######### | -1.35338 | 0.092 | 0.539 | ######### | ILC | Dstn |
| ######### | -1.57401 | 0.014 | 0.449 | ######### | ILC | Cpq |
| ######### | -1.83264 | 0.045 | 0.481 | ######### | ILC | Crip2 |
| ######### | -1.43563 | 0.009 | 0.444 | ######### | ILC | Fxyd1 |
| ######### | 1.226035 | 0.604 | 0.302 | ######### | ILC | Ccnd2 |
| ######### | -2.08298 | 0.018 | 0.45 | ######### | ILC | Gas1 |
| ######### | -2.34488 | 0.038 | 0.471 | ######### | ILC | Ly6a |

| | | | | | | |
|---|---|---|---|---|---|---|
| ######## | -1.90108 | 0.029 | 0.462 | ######## | ILC | Ecm1 |
| ######## | -1.80414 | 0.014 | 0.44 | ######## | ILC | Lamb1 |
| ######## | -1.45336 | 0.007 | 0.435 | ######## | ILC | Bicc1 |
| ######## | -1.64973 | 0.009 | 0.433 | ######## | ILC | Tcf21 |
| ######## | -2.85351 | 0.197 | 0.562 | ######## | ILC | Egr1 |
| ######## | -1.50984 | 0.006 | 0.431 | ######## | ILC | Cavin3 |
| ######## | -1.62134 | 0.017 | 0.442 | ######## | ILC | Olfml3 |
| ######## | -1.52547 | 0.009 | 0.429 | ######## | ILC | Ptgis |
| ######## | -1.51193 | 0.011 | 0.428 | ######## | ILC | Nupr1 |
| ######## | -1.84831 | 0.042 | 0.462 | ######## | ILC | Ier3 |
| ######## | -2.23019 | 0.558 | 0.786 | ######## | ILC | Fos |
| ######## | -1.55839 | 0.005 | 0.418 | ######## | ILC | Oaf |
| ######## | -1.71181 | 0.01 | 0.422 | ######## | ILC | Islr |
| ######## | -1.41662 | 0.01 | 0.423 | ######## | ILC | Lama2 |
| ######## | -1.81336 | 0.009 | 0.421 | ######## | ILC | Ogn |
| ######## | -2.5321 | 0.032 | 0.441 | ######## | ILC | Mfap5 |
| ######## | -1.3911 | 0.015 | 0.427 | ######## | ILC | Ddah2 |
| ######## | -1.45238 | 0.018 | 0.429 | ######## | ILC | Plxdc2 |
| ######## | -1.64983 | 0.021 | 0.434 | ######## | ILC | Spry1 |
| ######## | -1.66236 | 0.092 | 0.494 | ######## | ILC | Serpinb6a |
| ######## | 1.078835 | 0.518 | 0.232 | ######## | ILC | Zcchc11 |
| ######## | -1.54803 | 0.205 | 0.583 | ######## | ILC | Cyb5a |
| ######## | -1.25737 | 0.007 | 0.416 | ######## | ILC | S100a16 |
| ######## | 1.074167 | 0.684 | 0.438 | ######## | ILC | Higd1a |
| ######## | -2.0836 | 0.077 | 0.488 | ######## | ILC | Atf3 |
| ######## | -1.51283 | 0.011 | 0.416 | ######## | ILC | Ramp2 |
| ######## | -1.96159 | 0.029 | 0.433 | ######## | ILC | Ckb |
| ######## | -1.20536 | 0.007 | 0.41 | ######## | ILC | Fkbp9 |
| ######## | -1.71212 | 0.302 | 0.635 | ######## | ILC | Rhob |
| ######## | -1.32726 | 0.007 | 0.408 | ######## | ILC | Entpd2 |
| ######## | -1.26266 | 0.012 | 0.414 | ######## | ILC | Itgb5 |
| ######## | -1.27533 | 0.011 | 0.41 | ######## | ILC | Pdgfra |
| ######## | -1.28737 | 0.012 | 0.411 | ######## | ILC | Nfia |
| ######## | -1.3094 | 0.012 | 0.41 | ######## | ILC | Fhl1 |
| ######## | -1.34512 | 0.009 | 0.404 | ######## | ILC | Adamts2 |
| ######## | -1.21052 | 0.013 | 0.41 | ######## | ILC | Pkd2 |
| ######## | -1.86239 | 0.149 | 0.527 | ######## | ILC | Gstm1 |
| ######## | -1.36889 | 0.006 | 0.399 | ######## | ILC | Fbln5 |
| ######## | -1.34204 | 0.016 | 0.41 | ######## | ILC | Col5a1 |
| ######## | 1.060208 | 0.719 | 0.487 | ######## | ILC | Anp32b |
| ######## | -2.30484 | 0.015 | 0.405 | ######## | ILC | Dpep1 |
| ######## | -1.20999 | 0.987 | 0.989 | ######## | ILC | Ftl1 |
| ######## | -1.21167 | 0.012 | 0.404 | ######## | ILC | Cald1 |
| ######## | -1.28888 | 0.01 | 0.4 | ######## | ILC | Col5a2 |
| ######## | -3.04207 | 0.042 | 0.432 | ######## | ILC | Hspb1 |
| ######## | -1.15379 | 0.005 | 0.393 | ######## | ILC | Fermt2 |
| ######## | -1.53527 | 0.011 | 0.399 | ######## | ILC | Adamts5 |
| ######## | -1.29446 | 0.05 | 0.443 | ######## | ILC | Nfic |
| ######## | -1.19951 | 0.092 | 0.496 | ######## | ILC | Sntb2 |
| ######## | -2.31935 | 0.017 | 0.401 | ######## | ILC | Meg3 |
| ######## | -1.19091 | 0.005 | 0.389 | ######## | ILC | Mxra7 |
| ######## | -1.46816 | 0.026 | 0.413 | ######## | ILC | Pam |
| ######## | -1.30446 | 0.006 | 0.388 | ######## | ILC | Sod3 |
| ######## | -1.598 | 0.216 | 0.553 | ######## | ILC | Mtch1 |

| | | | | | | |
|---|---|---|---|---|---|---|
| ######## | -1.30601 | 0.007 | 0.388 | ######## | ILC | Ace |
| ######## | -1.2563 | 0.003 | 0.381 | ######## | ILC | Medag |
| ######## | -1.18394 | 0.005 | 0.383 | ######## | ILC | Nav1 |
| ######## | -1.04304 | 0.01 | 0.391 | ######## | ILC | Ckap4 |
| ######## | -1.17083 | 0.13 | 0.544 | ######## | ILC | Tnfrsf1a |
| ######## | -1.63775 | 0.01 | 0.388 | ######## | ILC | Col8a1 |
| ######## | -1.35112 | 0.009 | 0.385 | ######## | ILC | Fbn1 |
| ######## | -1.05755 | 0.05 | 0.441 | ######## | ILC | Grn |
| ######## | -1.2356 | 0.014 | 0.39 | ######## | ILC | Emilin1 |
| ######## | -1.45617 | 0.011 | 0.386 | ######## | ILC | Col6a3 |
| ######## | -2.06796 | 0.011 | 0.385 | ######## | ILC | C3 |
| ######## | -1.0601 | 0.011 | 0.386 | ######## | ILC | Fgfr1 |
| ######## | -2.48197 | 0.034 | 0.408 | ######## | ILC | Thbs1 |
| ######## | -1.13058 | 0.61 | 0.812 | ######## | ILC | Psap |
| ######## | -1.02451 | 0.012 | 0.385 | ######## | ILC | Fkbp7 |
| ######## | -1.17354 | 0.225 | 0.591 | ######## | ILC | Ctsz |
| ######## | -3.18155 | 0.061 | 0.431 | ######## | ILC | Apoe |
| ######## | -1.64542 | 0.01 | 0.38 | ######## | ILC | Timp3 |
| ######## | -1.49735 | 0.009 | 0.377 | ######## | ILC | Pcolce2 |
| ######## | -1.16164 | 0.009 | 0.377 | ######## | ILC | Lama4 |
| ######## | -1.70564 | 0.029 | 0.404 | ######## | ILC | Trib1 |
| ######## | -1.46721 | 0.122 | 0.493 | ######## | ILC | Tcf4 |
| ######## | -1.01924 | 0.005 | 0.373 | ######## | ILC | P3h3 |
| ######## | -1.33613 | 0.096 | 0.487 | ######## | ILC | Ehd1 |
| ######## | -1.28982 | 0.004 | 0.369 | ######## | ILC | Ppp1r14a |
| ######## | 1.087819 | 0.741 | 0.543 | ######## | ILC | Ankrd11 |
| ######## | -1.06404 | 0.007 | 0.373 | ######## | ILC | Cavin1 |
| ######## | -1.18775 | 0.369 | 0.648 | ######## | ILC | Lamp1 |
| ######## | -1.09851 | 0.009 | 0.369 | ######## | ILC | Il11ra1 |
| ######## | -1.13275 | 0.005 | 0.365 | ######## | ILC | Aebp1 |
| ######## | -1.03392 | 0.004 | 0.363 | ######## | ILC | Mmp23 |
| ######## | -1.48125 | 0.035 | 0.398 | ######## | ILC | Atf5 |
| ######## | -1.15907 | 0.145 | 0.514 | ######## | ILC | Tmed3 |
| ######## | -1.31587 | 0.358 | 0.626 | ######## | ILC | Laptm4a |
| ######## | -1.4564 | 0.263 | 0.591 | ######## | ILC | Selenop |
| ######## | -1.06292 | 0.002 | 0.354 | 1.45E-99 | ILC | Vwa1 |
| ######## | -1.05795 | 0.005 | 0.357 | 4.97E-99 | ILC | Dpysl3 |
| ######## | -1.1651 | 0.011 | 0.364 | 9.82E-99 | ILC | Angptl2 |
| ######## | -1.26631 | 0.013 | 0.366 | 9.88E-99 | ILC | Serpine2 |
| ######## | -1.06954 | 0.057 | 0.426 | 1.74E-98 | ILC | Rras |
| ######## | -1.05043 | 0.066 | 0.432 | 9.37E-98 | ILC | Vkorc1 |
| ######## | -1.21294 | 0.009 | 0.358 | 2.52E-97 | ILC | Tm4sf1 |
| ######## | -1.15 | 0.308 | 0.624 | 1.05E-96 | ILC | Ctsb |
| ######## | -1.21885 | 0.006 | 0.351 | 7.40E-96 | ILC | Htra1 |
| 2.71E-99 | -1.49115 | 0.392 | 0.681 | 4.88E-95 | ILC | Zfp36 |
| 2.97E-99 | -1.27804 | 0.104 | 0.468 | 5.35E-95 | ILC | Emp1 |
| 7.66E-99 | -1.6038 | 0.019 | 0.367 | 1.38E-94 | ILC | Tnfaip2 |
| 1.45E-98 | -1.20136 | 0.028 | 0.376 | 2.61E-94 | ILC | Selenbp1 |
| 1.88E-98 | -1.56272 | 0.01 | 0.35 | 3.39E-94 | ILC | Spon2 |
| 5.56E-98 | -1.55003 | 0.015 | 0.355 | 1.00E-93 | ILC | Aspn |
| 3.72E-97 | -1.3694 | 0.009 | 0.346 | 6.71E-93 | ILC | Gfpt2 |
| 5.28E-96 | -1.45189 | 0.004 | 0.337 | 9.51E-92 | ILC | Nbl1 |
| 5.81E-96 | -1.06665 | 0.007 | 0.342 | 1.05E-91 | ILC | Col4a2 |
| 1.24E-95 | -1.28921 | 0.006 | 0.341 | 2.23E-91 | ILC | Eln |

| | | | | | | |
|---|---|---|---|---|---|---|
| 7.31E-95 | -1.16782 | 0.011 | 0.342 | 1.32E-90 | ILC | Pla1a |
| 6.69E-92 | -1.08125 | 0.083 | 0.425 | 1.21E-87 | ILC | Slc29a1 |
| 7.41E-92 | -1.2528 | 0.005 | 0.329 | 1.33E-87 | ILC | Gsta3 |
| 1.42E-91 | -1.11325 | 0.003 | 0.324 | 2.56E-87 | ILC | Sfrp1 |
| 1.02E-89 | -1.19773 | 0.016 | 0.338 | 1.83E-85 | ILC | Gda |
| 2.80E-89 | -1.11262 | 0.009 | 0.327 | 5.05E-85 | ILC | Col5a3 |
| 1.07E-88 | -1.01531 | 0.003 | 0.316 | 1.92E-84 | ILC | Scara5 |
| 1.90E-87 | -1.13293 | 0.155 | 0.477 | 3.42E-83 | ILC | Nedd4 |
| 1.91E-87 | -1.053 | 0.007 | 0.32 | 3.44E-83 | ILC | Fxyd6 |
| 1.38E-85 | -1.34837 | 0.215 | 0.532 | 2.48E-81 | ILC | Cdkn1a |
| 1.14E-84 | -2.10255 | 0.02 | 0.327 | 2.06E-80 | ILC | Cyr61 |
| 1.49E-84 | -2.55739 | 0.042 | 0.351 | 2.69E-80 | ILC | Cxcl1 |
| 2.68E-84 | -1.04513 | 0.001 | 0.301 | 4.82E-80 | ILC | Gas6 |
| 5.16E-84 | -1.0725 | 0.005 | 0.308 | 9.30E-80 | ILC | Fibin |
| 4.91E-83 | -1.31349 | 0.691 | 0.83 | 8.84E-79 | ILC | Dusp1 |
| 1.34E-82 | -1.0978 | 0.005 | 0.304 | 2.42E-78 | ILC | Adamts1 |
| 5.47E-81 | -1.22942 | 0.185 | 0.499 | 9.84E-77 | ILC | Klf9 |
| 4.69E-79 | -2.05988 | 0.067 | 0.37 | 8.44E-75 | ILC | Gm12840 |
| 1.74E-78 | -3.00037 | 0.175 | 0.483 | 3.13E-74 | ILC | Msrb1 |
| 5.71E-78 | -1.08856 | 0.031 | 0.329 | 1.03E-73 | ILC | Bag3 |
| 1.07E-77 | 1.172158 | 0.631 | 0.45 | 1.93E-73 | ILC | Ubald2 |
| 1.23E-77 | -1.02893 | 0.155 | 0.468 | 2.22E-73 | ILC | Rhoc |
| 1.28E-77 | -1.13265 | 0.542 | 0.776 | 2.31E-73 | ILC | Sat1 |
| 2.48E-77 | -1.06585 | 0.002 | 0.284 | 4.47E-73 | ILC | Fmo2 |
| 2.95E-77 | -1.19884 | 0.277 | 0.54 | 5.31E-73 | ILC | Sptbn1 |
| 4.89E-77 | -1.7926 | 0.266 | 0.531 | 8.80E-73 | ILC | Socs3 |
| 7.33E-77 | -1.68619 | 0.021 | 0.309 | 1.32E-72 | ILC | Mt2 |
| 7.50E-76 | -1.31248 | 0.146 | 0.453 | 1.35E-71 | ILC | Errfi1 |
| 9.17E-75 | -1.13533 | 0.013 | 0.292 | 1.65E-70 | ILC | Tppp3 |
| 7.25E-74 | -1.63126 | 0.007 | 0.281 | 1.31E-69 | ILC | Ctgf |
| 3.10E-73 | -1.71233 | 0.221 | 0.498 | 5.58E-69 | ILC | Klf4 |
| 1.50E-71 | -1.26384 | 0.01 | 0.279 | 2.69E-67 | ILC | Ackr3 |
| 3.55E-71 | -1.01875 | 0.013 | 0.281 | 6.40E-67 | ILC | Sirpa |
| 4.60E-71 | -1.73451 | 0.025 | 0.296 | 8.29E-67 | ILC | Tgfbi |
| 2.29E-70 | -1.09701 | 0.402 | 0.7 | 4.13E-66 | ILC | Gpx1 |
| 2.59E-70 | -1.25664 | 0.321 | 0.545 | 4.66E-66 | ILC | Zbtb20 |
| 2.89E-70 | -2.45248 | 0.033 | 0.299 | 5.20E-66 | ILC | Alox5ap |
| 1.97E-69 | -1.23042 | 0.014 | 0.279 | 3.54E-65 | ILC | Tgm2 |
| 3.90E-69 | -1.16698 | 0.007 | 0.268 | 7.02E-65 | ILC | Hmox1 |
| 1.83E-68 | -1.66383 | 0.6 | 0.806 | 3.30E-64 | ILC | S100a11 |
| 2.84E-68 | -1.27562 | 0.494 | 0.645 | 5.11E-64 | ILC | Tuba1a |
| 5.62E-68 | -2.32175 | 0.041 | 0.304 | 1.01E-63 | ILC | Lst1 |
| 8.74E-68 | -1.14686 | 0.096 | 0.382 | 1.57E-63 | ILC | Uap1 |
| 6.02E-66 | -2.33165 | 0.047 | 0.307 | 1.08E-61 | ILC | Slc16a3 |
| 2.06E-61 | -1.78296 | 0.018 | 0.257 | 3.71E-57 | ILC | Spi1 |
| 2.38E-61 | -2.76905 | 0.04 | 0.282 | 4.29E-57 | ILC | Ccl6 |
| 6.10E-61 | -2.57383 | 0.025 | 0.263 | 1.10E-56 | ILC | Hp |
| 3.32E-60 | -1.62204 | 0.295 | 0.576 | 5.98E-56 | ILC | Grina |
| 9.59E-60 | -2.33374 | 0.024 | 0.259 | 1.73E-55 | ILC | Csf3r |
| 1.60E-59 | -2.79267 | 0.025 | 0.261 | 2.88E-55 | ILC | Il1r2 |
| 1.19E-58 | -2.05488 | 0.55 | 0.643 | 2.15E-54 | ILC | Jun |
| 1.52E-58 | -1.13174 | 0.296 | 0.537 | 2.73E-54 | ILC | Fosb |
| 2.42E-58 | -1.08852 | 0.259 | 0.514 | 4.36E-54 | ILC | Pmepa1 |
| 1.10E-55 | -1.03948 | 0.593 | 0.782 | 1.99E-51 | ILC | Txn1 |

| | | | | | | |
|---|---|---|---|---|---|---|
| 2.01E-55 | -2.58621 | 0.029 | 0.252 | 3.62E-51 | ILC | Hdc |
| 3.24E-55 | -3.16061 | 0.046 | 0.272 | 5.84E-51 | ILC | Il1b |
| 8.30E-55 | -1.05074 | 0.362 | 0.607 | 1.49E-50 | ILC | Anxa1 |
| 4.38E-54 | -1.93788 | 0.051 | 0.272 | 7.89E-50 | ILC | Ncf2 |
| 1.21E-53 | -4.04727 | 0.096 | 0.319 | 2.18E-49 | ILC | Lyz2 |
| 2.69E-53 | -1.86242 | 0.09 | 0.321 | 4.83E-49 | ILC | Plaur |
| 3.57E-53 | -1.19742 | 0.109 | 0.356 | 6.42E-49 | ILC | Gadd45g |
| 3.25E-50 | -1.53089 | 0.116 | 0.35 | 5.86E-46 | ILC | Rnf149 |
| 4.36E-50 | -1.54629 | 0.107 | 0.334 | 7.85E-46 | ILC | Ets2 |
| 4.44E-49 | -1.40632 | 0.063 | 0.275 | 7.99E-45 | ILC | Tpd52 |
| 5.23E-48 | -1.53226 | 0.793 | 0.663 | 9.42E-44 | ILC | Hbb-bs |
| 6.01E-48 | -4.98204 | 0.127 | 0.337 | 1.08E-43 | ILC | Retnlg |
| 2.16E-45 | -1.0746 | 0.103 | 0.325 | 3.88E-41 | ILC | Tmcc1 |
| 3.97E-43 | -5.30271 | 0.281 | 0.476 | 7.15E-39 | ILC | S100a8 |
| 2.98E-40 | -1.0888 | 0.418 | 0.653 | 5.37E-36 | ILC | Prdx5 |
| 3.98E-38 | -1.53762 | 0.649 | 0.493 | 7.16E-34 | ILC | Hba-a1 |
| 5.06E-35 | -1.79312 | 0.731 | 0.845 | 9.10E-31 | ILC | Cebpb |
| 2.22E-32 | -1.80553 | 0.132 | 0.29 | 4.00E-28 | ILC | Lmnb1 |
| 6.31E-31 | -5.26727 | 0.35 | 0.509 | 1.14E-26 | ILC | S100a9 |
| 1.03E-28 | -1.31331 | 0.472 | 0.658 | 1.85E-24 | ILC | Taldo1 |
| 7.59E-22 | -2.32166 | 0.14 | 0.268 | 1.37E-17 | ILC | Ccrl2 |
| 1.59E-15 | -1.24176 | 0.159 | 0.273 | 2.86E-11 | ILC | Isg15 |
| 0 | 6.943457 | 0.985 | 0.049 | 0 | B cells | Igkc |
| 0 | 5.568732 | 0.991 | 0.081 | 0 | B cells | Cd74 |
| 0 | 5.229647 | 0.98 | 0.01 | 0 | B cells | Cd79a |
| 0 | 4.863345 | 0.948 | 0.031 | 0 | B cells | Ighm |
| 0 | 4.462581 | 0.966 | 0.04 | 0 | B cells | H2-Aa |
| 0 | 4.373685 | 0.958 | 0.025 | 0 | B cells | H2-Eb1 |
| 0 | 3.790688 | 0.939 | 0.037 | 0 | B cells | H2-Ab1 |
| 0 | 3.384176 | 0.828 | 0.005 | 0 | B cells | Ighd |
| 0 | 3.241432 | 0.684 | 0.004 | 0 | B cells | Cd79b |
| 0 | 3.17758 | 0.349 | 0.003 | 0 | B cells | Iglc3 |
| 0 | 3.141209 | 0.962 | 0.405 | 0 | B cells | Ebf1 |
| 0 | 3.104077 | 0.757 | 0.004 | 0 | B cells | Fcmr |
| 0 | 2.995951 | 0.52 | 0.003 | 0 | B cells | Iglc2 |
| 0 | 2.992748 | 0.813 | 0.145 | 0 | B cells | Mef2c |
| 0 | 2.887555 | 0.702 | 0.09 | 0 | B cells | Cd83 |
| 0 | 2.860872 | 0.801 | 0.071 | 0 | B cells | Ccr7 |
| 0 | 2.599509 | 0.617 | 0.004 | 0 | B cells | Pou2af1 |
| 0 | 2.430012 | 0.606 | 0.007 | 0 | B cells | Tnfrsf13c |
| 0 | 2.410608 | 0.548 | 0.006 | 0 | B cells | Fcrla |
| 0 | 2.295446 | 0.863 | 0.237 | 0 | B cells | Cd37 |
| 0 | 2.295338 | 0.581 | 0.007 | 0 | B cells | Bank1 |
| 0 | 2.230033 | 0.493 | 0.003 | 0 | B cells | Ms4a1 |
| 0 | 2.195721 | 0.466 | 0.004 | 0 | B cells | Mzb1 |
| 0 | 2.168041 | 0.602 | 0.116 | 0 | B cells | Rel |
| 0 | 2.154162 | 0.674 | 0.088 | 0 | B cells | Napsa |
| 0 | 2.14541 | 0.737 | 0.174 | 0 | B cells | Satb1 |
| 0 | 2.116172 | 0.52 | 0.006 | 0 | B cells | H2-DMb2 |
| 0 | 2.084804 | 0.504 | 0.004 | 0 | B cells | H2-Ob |
| 0 | 1.983413 | 0.569 | 0.051 | 0 | B cells | H2-DMa |
| 0 | 1.965338 | 0.408 | 0.002 | 0 | B cells | Fcer2a |
| 0 | 1.928832 | 0.812 | 0.283 | 0 | B cells | Fam107b |
| 0 | 1.882303 | 0.472 | 0.002 | 0 | B cells | Cd19 |

| | | | | | | |
|---|---|---|---|---|---|---|
| 0 | 1.772923 | 0.438 | 0.032 | 0 | B cells | Ralgps2 |
| 0 | 1.754604 | 0.282 | 0.002 | 0 | B cells | Ly6d |
| 0 | 1.706155 | 0.395 | 0.004 | 0 | B cells | Cxcr5 |
| 0 | 1.689875 | 0.317 | 0.001 | 0 | B cells | Vpreb3 |
| 0 | 1.66113 | 0.715 | 0.175 | 0 | B cells | Dusp2 |
| 0 | 1.579391 | 0.373 | 0.013 | 0 | B cells | H2-Oa |
| 0 | 1.573847 | 0.346 | 0.007 | 0 | B cells | Siglecg |
| 0 | 1.484827 | 0.416 | 0.058 | 0 | B cells | Lamb3 |
| 0 | 1.433841 | 0.314 | 0.002 | 0 | B cells | Pax5 |
| 0 | 1.346167 | 0.308 | 0.014 | 0 | B cells | Cd72 |
| 0 | 1.331468 | 0.26 | 0.002 | 0 | B cells | Spib |
| 0 | 1.323911 | 0.313 | 0.023 | 0 | B cells | Ly86 |
| 0 | 1.300425 | 0.302 | 0.016 | 0 | B cells | Mtss1 |
| 0 | 1.282633 | 1 | 0.991 | 0 | B cells | Fau |
| 0 | 1.22528 | 0.592 | 0.108 | 0 | B cells | Ctss |
| 0 | 1.15057 | 0.256 | 0.002 | 0 | B cells | Gm31243 |
| 0 | -4.24932 | 0.109 | 0.892 | 0 | B cells | S100a6 |
| ######## | -2.06663 | 0.703 | 0.962 | ######## | B cells | Itm2b |
| ######## | 1.547646 | 0.614 | 0.135 | ######## | B cells | Ptprcap |
| ######## | 1.40831 | 1 | 0.865 | ######## | B cells | Rps24 |
| ######## | 1.501471 | 0.996 | 0.788 | ######## | B cells | Rps20 |
| ######## | 1.121402 | 0.301 | 0.031 | ######## | B cells | H2-DMb1 |
| ######## | 2.468643 | 0.862 | 0.486 | ######## | B cells | Smc6 |
| ######## | 1.41868 | 0.999 | 0.968 | ######## | B cells | Rps27 |
| ######## | 1.648543 | 0.428 | 0.071 | ######## | B cells | Stap1 |
| ######## | 1.329393 | 0.999 | 0.808 | ######## | B cells | Rps19 |
| ######## | 1.482205 | 0.354 | 0.049 | ######## | B cells | Gpr171 |
| ######## | 1.532946 | 0.989 | 0.95 | ######## | B cells | H3f3a |
| ######## | -3.42344 | 0.068 | 0.783 | ######## | B cells | Ifitm2 |
| ######## | 1.20263 | 0.997 | 0.844 | ######## | B cells | Rps3a1 |
| ######## | 1.322588 | 0.259 | 0.025 | ######## | B cells | Pou2f2 |
| ######## | -2.71134 | 0.053 | 0.783 | ######## | B cells | Anxa2 |
| ######## | 1.195252 | 0.999 | 0.92 | ######## | B cells | Rpl18a |
| ######## | 1.215516 | 0.996 | 0.842 | ######## | B cells | Rps7 |
| ######## | 1.097179 | 0.999 | 0.884 | ######## | B cells | Rpl35a |
| ######## | 1.158419 | 1 | 0.867 | ######## | B cells | Rps13 |
| ######## | 1.061106 | 0.999 | 0.922 | ######## | B cells | Rpl9 |
| ######## | -2.39366 | 0.105 | 0.802 | ######## | B cells | Ctsd |
| ######## | 1.535726 | 0.462 | 0.094 | ######## | B cells | P2ry10 |
| ######## | -3.55905 | 0.058 | 0.734 | ######## | B cells | Ifitm3 |
| ######## | 1.136769 | 0.999 | 0.935 | ######## | B cells | Rps29 |
| ######## | 1.302176 | 0.332 | 0.05 | ######## | B cells | Trp53i11 |
| ######## | 1.468987 | 0.332 | 0.051 | ######## | B cells | Hvcn1 |
| ######## | 1.106092 | 0.989 | 0.836 | ######## | B cells | Rpl7 |
| ######## | 1.826626 | 0.867 | 0.567 | ######## | B cells | Serp1 |
| ######## | 1.154611 | 0.313 | 0.044 | ######## | B cells | Plekho1 |
| ######## | 1.056097 | 0.999 | 0.858 | ######## | B cells | Rpl8 |
| ######## | 1.084662 | 1 | 0.812 | ######## | B cells | Rps15a |
| ######## | -2.32075 | 0.147 | 0.797 | ######## | B cells | Txn1 |
| ######## | 1.054287 | 0.999 | 0.878 | ######## | B cells | Rpl34 |
| ######## | 1.074552 | 0.999 | 0.867 | ######## | B cells | Rps3 |
| ######## | 1.023242 | 1 | 0.823 | ######## | B cells | Rps4x |
| ######## | 1.05888 | 0.997 | 0.818 | ######## | B cells | Rpl6 |
| ######## | 1.045473 | 0.997 | 0.873 | ######## | B cells | mt-Co2 |

| | | | | | | |
|---|---|---|---|---|---|---|
| ######## | 1.248906 | 0.314 | 0.046 | ######## | B cells | Ppp1r16b |
| ######## | 1.260059 | 0.991 | 0.8 | ######## | B cells | Ptma |
| ######## | 1.186104 | 0.985 | 0.761 | ######## | B cells | Rpl12 |
| ######## | 1.055251 | 0.999 | 0.804 | ######## | B cells | Rpl32 |
| ######## | 1.273063 | 0.582 | 0.148 | ######## | B cells | Gimap6 |
| ######## | 1.042897 | 0.997 | 0.896 | ######## | B cells | Rpl17 |
| ######## | 1.029167 | 0.997 | 0.85 | ######## | B cells | Rpl39 |
| ######## | 1.432071 | 0.469 | 0.106 | ######## | B cells | Il2rg |
| ######## | 1.083692 | 0.995 | 0.897 | ######## | B cells | Rps10 |
| ######## | 1.001102 | 0.997 | 0.839 | ######## | B cells | Rpl19 |
| ######## | 1.162259 | 0.293 | 0.043 | ######## | B cells | Chchd10 |
| ######## | -2.67931 | 0.023 | 0.667 | ######## | B cells | Timp2 |
| ######## | 1.253033 | 0.972 | 0.763 | ######## | B cells | mt-Nd2 |
| ######## | 1.586394 | 0.732 | 0.29 | ######## | B cells | Ets1 |
| ######## | -4.2722 | 0.073 | 0.676 | ######## | B cells | Cebpd |
| ######## | 1.782041 | 0.68 | 0.297 | ######## | B cells | Foxp1 |
| ######## | -2.75399 | 0.256 | 0.817 | ######## | B cells | S100a11 |
| ######## | -2.74379 | 0.127 | 0.697 | ######## | B cells | Lgals1 |
| ######## | -3.01659 | 0.02 | 0.622 | ######## | B cells | Cd63 |
| ######## | 1.025654 | 0.985 | 0.787 | ######## | B cells | mt-Nd1 |
| ######## | 1.016818 | 0.989 | 0.861 | ######## | B cells | Rps21 |
| ######## | 1.758675 | 0.534 | 0.186 | ######## | B cells | Scd1 |
| ######## | -2.79655 | 0.028 | 0.623 | ######## | B cells | Cd9 |
| ######## | 1.684407 | 0.65 | 0.292 | ######## | B cells | Snx5 |
| ######## | -2.47641 | 0.227 | 0.79 | ######## | B cells | Fxyd5 |
| ######## | -2.26189 | 0.027 | 0.617 | ######## | B cells | Anxa1 |
| ######## | 1.191442 | 0.341 | 0.069 | ######## | B cells | Prkcb |
| ######## | -3.24622 | 0.034 | 0.603 | ######## | B cells | Pi16 |
| ######## | 1.233142 | 0.359 | 0.076 | ######## | B cells | Gm8369 |
| ######## | 1.364932 | 0.469 | 0.139 | ######## | B cells | Cyth1 |
| ######## | -5.34528 | 0.416 | 0.784 | ######## | B cells | Gsn |
| ######## | 1.455875 | 0.828 | 0.637 | ######## | B cells | Gdi2 |
| ######## | 1.359818 | 0.763 | 0.335 | ######## | B cells | Vps37b |
| ######## | 1.283539 | 0.456 | 0.132 | ######## | B cells | Ctsh |
| ######## | -2.33288 | 0.101 | 0.624 | ######## | B cells | Anxa5 |
| ######## | -2.70987 | 0.02 | 0.562 | ######## | B cells | Crispld2 |
| ######## | -2.74208 | 0.08 | 0.595 | ######## | B cells | Ctsl |
| ######## | -2.90275 | 0.024 | 0.559 | ######## | B cells | Igfbp4 |
| ######## | -1.85341 | 0.019 | 0.559 | ######## | B cells | App |
| ######## | -1.6792 | 0.007 | 0.545 | ######## | B cells | Tnfrsf1a |
| ######## | 1.63491 | 0.524 | 0.202 | ######## | B cells | Ptp4a3 |
| ######## | -1.83202 | 0.135 | 0.647 | ######## | B cells | Ptms |
| ######## | -4.23098 | 0.089 | 0.589 | ######## | B cells | Igfbp7 |
| ######## | -2.07405 | 0.024 | 0.562 | ######## | B cells | Mgst1 |
| ######## | -2.17496 | 0.072 | 0.596 | ######## | B cells | Selenop |
| ######## | -4.05641 | 0.057 | 0.56 | ######## | B cells | Bgn |
| ######## | 1.533653 | 0.691 | 0.38 | ######## | B cells | Tgfb1 |
| ######## | 1.244314 | 0.858 | 0.685 | ######## | B cells | Shisa5 |
| ######## | 1.13765 | 0.341 | 0.08 | ######## | B cells | Acp5 |
| ######## | -3.40196 | 0.024 | 0.532 | ######## | B cells | Serping1 |
| ######## | -4.55676 | 0.102 | 0.586 | ######## | B cells | Mgp |
| ######## | -3.7622 | 0.054 | 0.551 | ######## | B cells | Htra3 |
| ######## | 1.221854 | 0.362 | 0.09 | ######## | B cells | Rhoh |
| ######## | 1.656975 | 0.706 | 0.408 | ######## | B cells | Ezr |

| | | | | | | |
|---|---|---|---|---|---|---|
| ######## | -3.93747 | 0.052 | 0.545 | ######## | B cells | Sparc |
| ######## | -3.18263 | 0.02 | 0.53 | ######## | B cells | G0s2 |
| ######## | -3.44355 | 0.045 | 0.538 | ######## | B cells | Lum |
| ######## | -2.1324 | 0.431 | 0.774 | ######## | B cells | Vim |
| ######## | -5.45103 | 0.257 | 0.656 | ######## | B cells | Dcn |
| ######## | -1.90794 | 0.442 | 0.838 | ######## | B cells | Dusp1 |
| ######## | -3.57295 | 0.041 | 0.531 | ######## | B cells | Clec3b |
| ######## | -2.95431 | 0.025 | 0.52 | ######## | B cells | Pcolce |
| ######## | -2.91264 | 0.045 | 0.531 | ######## | B cells | Ltbp4 |
| ######## | -3.49106 | 0.076 | 0.549 | ######## | B cells | Sparcl1 |
| ######## | -2.70521 | 0.025 | 0.52 | ######## | B cells | Cygb |
| ######## | -2.06502 | 0.023 | 0.513 | ######## | B cells | Selenom |
| ######## | -1.9062 | 0.012 | 0.509 | ######## | B cells | Rnase4 |
| ######## | 1.187278 | 0.366 | 0.099 | ######## | B cells | Dgkd |
| ######## | -2.41103 | 0.028 | 0.52 | ######## | B cells | Rarres2 |
| ######## | -3.2053 | 0.038 | 0.52 | ######## | B cells | Col1a2 |
| ######## | -2.32024 | 0.04 | 0.528 | ######## | B cells | Gstm1 |
| ######## | -3.00858 | 0.036 | 0.521 | ######## | B cells | Gpx3 |
| ######## | -1.55983 | 0.135 | 0.628 | ######## | B cells | Ctsb |
| ######## | -2.12469 | 0.016 | 0.505 | ######## | B cells | Lhfp |
| ######## | -1.35198 | 0.267 | 0.747 | ######## | B cells | Tspo |
| ######## | -2.54435 | 0.432 | 0.789 | ######## | B cells | Fos |
| ######## | -1.5224 | 0.034 | 0.525 | ######## | B cells | Itm2c |
| ######## | 1.132997 | 0.765 | 0.365 | ######## | B cells | Cytip |
| ######## | -2.04678 | 0.016 | 0.503 | ######## | B cells | Lrp1 |
| ######## | -3.03091 | 0.099 | 0.563 | ######## | B cells | Egr1 |
| ######## | -1.77962 | 0.109 | 0.583 | ######## | B cells | Cyb5a |
| ######## | -1.5656 | 0.2 | 0.691 | ######## | B cells | Crip1 |
| ######## | -1.72536 | 0.041 | 0.533 | ######## | B cells | Marcks |
| ######## | 1.451676 | 0.499 | 0.193 | ######## | B cells | Crem |
| ######## | -2.49446 | 0.028 | 0.504 | ######## | B cells | Cd34 |
| ######## | -3.06654 | 0.037 | 0.506 | ######## | B cells | Serpinh1 |
| ######## | -2.5646 | 0.02 | 0.494 | ######## | B cells | Col6a1 |
| ######## | -3.03305 | 0.027 | 0.501 | ######## | B cells | Fbln1 |
| ######## | 1.225971 | 0.365 | 0.096 | ######## | B cells | Cd69 |
| ######## | -2.4801 | 0.024 | 0.497 | ######## | B cells | Cfh |
| ######## | -3.00121 | 0.041 | 0.506 | ######## | B cells | Col1a1 |
| ######## | -3.41613 | 0.06 | 0.519 | ######## | B cells | Col3a1 |
| ######## | -1.8643 | 0.081 | 0.571 | ######## | B cells | Zyx |
| ######## | -2.04768 | 0.016 | 0.488 | ######## | B cells | Pmp22 |
| ######## | -1.36835 | 0.424 | 0.817 | ######## | B cells | Psap |
| ######## | 1.265102 | 0.797 | 0.581 | ######## | B cells | Rps27rt |
| ######## | -2.00289 | 0.013 | 0.483 | ######## | B cells | Axl |
| ######## | -2.03112 | 0.009 | 0.477 | ######## | B cells | Rbp1 |
| ######## | -1.71394 | 0.016 | 0.487 | ######## | B cells | Cd302 |
| ######## | -2.41601 | 0.025 | 0.49 | ######## | B cells | Serpinf1 |
| ######## | -1.64461 | 0.005 | 0.47 | ######## | B cells | Rhoc |
| ######## | -2.43116 | 0.017 | 0.482 | ######## | B cells | Fbln2 |
| ######## | -1.78049 | 0.011 | 0.477 | ######## | B cells | Rcn3 |
| ######## | -2.13936 | 0.024 | 0.49 | ######## | B cells | Dpt |
| ######## | -2.43109 | 0.02 | 0.482 | ######## | B cells | Col6a2 |
| ######## | -2.31819 | 0.04 | 0.5 | ######## | B cells | Mmp2 |
| ######## | -2.27196 | 0.02 | 0.484 | ######## | B cells | Fstl1 |
| ######## | -2.49336 | 0.02 | 0.481 | ######## | B cells | Ccdc80 |

| | | | | | | |
|---|---|---|---|---|---|---|
| ######## | -2.00216 | 0.013 | 0.476 | ######## | B cells | Nfix |
| ######## | -3.83504 | 0.107 | 0.554 | ######## | B cells | Mt1 |
| ######## | -3.47369 | 0.041 | 0.496 | ######## | B cells | Lpl |
| ######## | -1.42693 | 0.058 | 0.536 | ######## | B cells | Dstn |
| ######## | -1.8342 | 0.029 | 0.493 | ######## | B cells | Serpinb6a |
| ######## | -1.27013 | 0.158 | 0.658 | ######## | B cells | Rbms1 |
| ######## | -1.68684 | 0.042 | 0.505 | ######## | B cells | Nenf |
| ######## | -1.41246 | 0.028 | 0.495 | ######## | B cells | Sntb2 |
| ######## | -2.43762 | 0.016 | 0.472 | ######## | B cells | Col15a1 |
| ######## | -1.65863 | 0.013 | 0.472 | ######## | B cells | Fosl2 |
| ######## | 1.189119 | 0.336 | 0.094 | ######## | B cells | Map3k1 |
| ######## | -2.48901 | 0.085 | 0.536 | ######## | B cells | Socs3 |
| ######## | -1.99101 | 0.013 | 0.468 | ######## | B cells | Hspg2 |
| ######## | -2.10047 | 0.021 | 0.475 | ######## | B cells | Nid1 |
| ######## | -2.57245 | 0.027 | 0.476 | ######## | B cells | Smoc2 |
| ######## | -1.71206 | 0.011 | 0.464 | ######## | B cells | Nfib |
| ######## | -2.15564 | 0.106 | 0.581 | ######## | B cells | Grina |
| ######## | -1.87894 | 0.009 | 0.46 | ######## | B cells | Prelp |
| ######## | -1.87489 | 0.072 | 0.535 | ######## | B cells | Cdkn1a |
| ######## | 1.240926 | 0.786 | 0.585 | ######## | B cells | Sub1 |
| ######## | -1.78622 | 0.117 | 0.554 | ######## | B cells | Mtch1 |
| ######## | -1.65162 | 0.008 | 0.456 | ######## | B cells | Rhoj |
| ######## | -1.73258 | 0.017 | 0.466 | ######## | B cells | Mxra8 |
| ######## | -1.74001 | 0.021 | 0.472 | ######## | B cells | Sdc2 |
| ######## | -1.86608 | 0.017 | 0.464 | ######## | B cells | Loxl1 |
| ######## | -1.84655 | 0.011 | 0.457 | ######## | B cells | Tnxb |
| ######## | -1.57022 | 0.031 | 0.479 | ######## | B cells | Nedd4 |
| ######## | -1.9935 | 0.013 | 0.457 | ######## | B cells | Col4a1 |
| ######## | -1.86923 | 0.013 | 0.459 | ######## | B cells | Ier3 |
| ######## | -1.57775 | 0.021 | 0.466 | ######## | B cells | Camk2n1 |
| ######## | -1.74525 | 0.007 | 0.449 | ######## | B cells | Gpm6b |
| ######## | 1.02945 | 0.451 | 0.145 | ######## | B cells | Cd2 |
| ######## | -2.23291 | 0.041 | 0.485 | ######## | B cells | Atf3 |
| ######## | -1.36014 | 0.038 | 0.493 | ######## | B cells | Dhrs7 |
| ######## | -1.75533 | 0.024 | 0.468 | ######## | B cells | Slc43a3 |
| ######## | -1.35764 | 0.069 | 0.524 | ######## | B cells | Itgb1 |
| ######## | -1.61379 | 0.025 | 0.468 | ######## | B cells | Emp1 |
| ######## | -1.9308 | 0.019 | 0.458 | ######## | B cells | Ecm1 |
| ######## | -1.71905 | 0.021 | 0.461 | ######## | B cells | Abca8a |
| ######## | -1.57653 | 0.012 | 0.446 | ######## | B cells | Cpq |
| ######## | -1.78861 | 0.038 | 0.477 | ######## | B cells | Crip2 |
| ######## | -1.66649 | 0.007 | 0.438 | ######## | B cells | Olfml3 |
| ######## | -2.06872 | 0.015 | 0.447 | ######## | B cells | Gas1 |
| ######## | -1.89637 | 0.016 | 0.446 | ######## | B cells | Pcsk6 |
| ######## | -2.51883 | 0.036 | 0.467 | ######## | B cells | Ly6a |
| ######## | -1.46155 | 0.004 | 0.431 | ######## | B cells | Bicc1 |
| ######## | -1.41009 | 0.011 | 0.44 | ######## | B cells | Fxyd1 |
| ######## | -1.71208 | 0.232 | 0.635 | ######## | B cells | Rhob |
| ######## | -1.79586 | 0.023 | 0.45 | ######## | B cells | Plpp3 |
| ######## | -1.51721 | 0.004 | 0.427 | ######## | B cells | Cavin3 |
| ######## | -1.79158 | 0.012 | 0.436 | ######## | B cells | Lamb1 |
| ######## | 1.215319 | 0.321 | 0.095 | ######## | B cells | Cnp |
| ######## | 1.274227 | 0.336 | 0.105 | ######## | B cells | Nsf |
| ######## | -1.62128 | 0.008 | 0.43 | ######## | B cells | Tcf21 |

| | | | | | | |
|---|---|---|---|---|---|---|
| ######## | -1.36883 | 0.143 | 0.611 | ######## | B cells | Mrpl33 |
| ######## | -1.32192 | 0.076 | 0.514 | ######## | B cells | Tmed3 |
| ######## | -1.69073 | 0.011 | 0.431 | ######## | B cells | Spry1 |
| ######## | -1.47346 | 0.009 | 0.426 | ######## | B cells | Plxdc2 |
| ######## | 1.219236 | 0.52 | 0.236 | ######## | B cells | Unc93b1 |
| ######## | -1.41404 | 0.911 | 0.978 | ######## | B cells | Actg1 |
| ######## | -1.27936 | 0.001 | 0.413 | ######## | B cells | S100a16 |
| ######## | -1.53987 | 0.003 | 0.413 | ######## | B cells | Ramp2 |
| ######## | -1.49385 | 0.012 | 0.426 | ######## | B cells | Ptgis |
| ######## | -1.36442 | 0.023 | 0.44 | ######## | B cells | Nfic |
| ######## | -1.49172 | 0.012 | 0.424 | 1.01E-99 | B cells | Nupr1 |
| ######## | -1.98083 | 0.019 | 0.43 | 2.73E-99 | B cells | Ckb |
| ######## | -1.5462 | 0.005 | 0.414 | 3.12E-99 | B cells | Oaf |
| ######## | -1.66858 | 0.036 | 0.454 | 8.67E-99 | B cells | Errfi1 |
| ######## | -1.25396 | 0 | 0.406 | 9.92E-99 | B cells | Selenon |
| ######## | -1.80443 | 0.009 | 0.417 | 1.41E-98 | B cells | Ogn |
| ######## | -1.69149 | 0.011 | 0.419 | 1.57E-98 | B cells | Islr |
| ######## | -1.59016 | 0.08 | 0.5 | 2.29E-98 | B cells | Klf9 |
| ######## | -1.26525 | 0.038 | 0.457 | 1.94E-97 | B cells | Fcgrt |
| ######## | -2.45427 | 0.032 | 0.437 | 2.87E-97 | B cells | Mfap5 |
| ######## | -1.39176 | 0.013 | 0.42 | 4.44E-97 | B cells | Lama2 |
| ######## | -1.24648 | 0.004 | 0.407 | 4.69E-97 | B cells | Pkd2 |
| ######## | -1.12005 | 0.11 | 0.554 | 1.58E-96 | B cells | Ifi27 |
| ######## | -1.28104 | 0.005 | 0.407 | 4.25E-96 | B cells | Pdgfra |
| ######## | -1.37724 | 0.007 | 0.407 | 5.03E-96 | B cells | Col5a1 |
| ######## | -1.14081 | 0.155 | 0.596 | 6.13E-96 | B cells | Lamp2 |
| ######## | -1.29732 | 0.007 | 0.408 | 7.21E-96 | B cells | Nfia |
| ######## | -1.33355 | 0.005 | 0.404 | 1.31E-95 | B cells | Entpd2 |
| 2.04E-99 | -1.17656 | 0.007 | 0.406 | 3.67E-95 | B cells | Fkbp9 |
| 3.74E-99 | -1.30569 | 0.085 | 0.504 | 6.73E-95 | B cells | Lmna |
| 4.01E-99 | 1.011416 | 0.45 | 0.17 | 7.22E-95 | B cells | Inpp5d |
| 4.50E-99 | -1.26593 | 0.02 | 0.424 | 8.11E-95 | B cells | Slc29a1 |
| 6.12E-99 | 1.218786 | 0.729 | 0.564 | 1.10E-94 | B cells | Ncl |
| 2.23E-98 | -1.49145 | 0.012 | 0.41 | 4.02E-94 | B cells | Pam |
| 2.35E-98 | -1.30662 | 0.009 | 0.406 | 4.23E-94 | B cells | Fhl1 |
| 2.80E-98 | -1.35623 | 0.005 | 0.4 | 5.05E-94 | B cells | Adamts2 |
| 5.77E-98 | -1.5848 | 0.081 | 0.481 | 1.04E-93 | B cells | Lamc1 |
| 9.63E-98 | -1.23971 | 0.013 | 0.41 | 1.73E-93 | B cells | Itgb5 |
| 1.81E-97 | -1.53898 | 0.215 | 0.659 | 3.25E-93 | B cells | Prdx5 |
| 5.86E-97 | -1.18403 | 0.008 | 0.4 | 1.06E-92 | B cells | Rora |
| 6.80E-97 | -1.31435 | 0.005 | 0.396 | 1.22E-92 | B cells | Col5a2 |
| 2.12E-96 | -1.22819 | 0.009 | 0.401 | 3.82E-92 | B cells | Cald1 |
| 2.93E-96 | -2.16592 | 0.061 | 0.467 | 5.27E-92 | B cells | AW112010 |
| 4.95E-96 | -1.10243 | 0.025 | 0.424 | 8.92E-92 | B cells | Snx18 |
| 7.93E-96 | -1.15945 | 0.003 | 0.389 | 1.43E-91 | B cells | Fermt2 |
| 3.46E-95 | 1.360022 | 0.462 | 0.222 | 6.24E-91 | B cells | Pkig |
| 4.14E-95 | -1.27608 | 0.027 | 0.423 | 7.45E-91 | B cells | Ddah2 |
| 3.57E-94 | -1.29943 | 0.178 | 0.583 | 6.43E-90 | B cells | Ahnak |
| 5.42E-94 | -2.24568 | 0.017 | 0.402 | 9.75E-90 | B cells | Dpep1 |
| 6.98E-94 | -2.29669 | 0.015 | 0.398 | 1.26E-89 | B cells | Meg3 |
| 7.02E-94 | -1.65591 | 0.759 | 0.847 | 1.26E-89 | B cells | Cst3 |
| 3.42E-93 | -1.25174 | 0.007 | 0.387 | 6.16E-89 | B cells | Emilin1 |
| 5.28E-93 | -1.17631 | 0.005 | 0.386 | 9.51E-89 | B cells | Mxra7 |
| 6.21E-93 | -1.52837 | 0.013 | 0.395 | 1.12E-88 | B cells | Adamts5 |

| | | | | | | |
|---|---|---|---|---|---|---|
| 9.50E-93 | -3.35187 | 0.092 | 0.483 | 1.71E-88 | B cells | Msrb1 |
| 1.51E-92 | -1.32909 | 0.028 | 0.416 | 2.72E-88 | B cells | Itm2a |
| 2.15E-92 | -1.27518 | 0.012 | 0.396 | 3.87E-88 | B cells | Fbln5 |
| 2.94E-92 | -2.55692 | 0.024 | 0.405 | 5.30E-88 | B cells | Thbs1 |
| 4.14E-92 | -1.07201 | 0.005 | 0.383 | 7.46E-88 | B cells | Fgfr1 |
| 5.45E-92 | -1.03862 | 0.005 | 0.382 | 9.80E-88 | B cells | Fkbp7 |
| 5.52E-92 | -2.91346 | 0.044 | 0.428 | 9.94E-88 | B cells | Hspb1 |
| 2.23E-91 | -1.6448 | 0.009 | 0.384 | 4.01E-87 | B cells | Col8a1 |
| 2.48E-91 | -1.38817 | 0.089 | 0.499 | 4.47E-87 | B cells | Picalm |
| 3.92E-91 | -1.2881 | 0.009 | 0.385 | 7.06E-87 | B cells | Sod3 |
| 4.02E-91 | -2.40395 | 0.527 | 0.851 | 7.24E-87 | B cells | Cebpb |
| 4.04E-91 | -1.48059 | 0.009 | 0.383 | 7.27E-87 | B cells | Col6a3 |
| 6.11E-91 | -1.29508 | 0.001 | 0.374 | 1.10E-86 | B cells | Selenbp1 |
| 8.83E-91 | -1.02927 | 0.044 | 0.438 | 1.59E-86 | B cells | Grn |
| 1.55E-90 | -1.28044 | 0.009 | 0.384 | 2.78E-86 | B cells | Ace |
| 1.86E-90 | -1.52418 | 0.02 | 0.396 | 3.35E-86 | B cells | Atf5 |
| 2.35E-90 | -1.23261 | 0.005 | 0.377 | 4.23E-86 | B cells | Medag |
| 3.25E-90 | -1.66279 | 0.005 | 0.377 | 5.86E-86 | B cells | Timp3 |
| 4.52E-90 | -1.55469 | 0.183 | 0.548 | 8.14E-86 | B cells | Zbtb20 |
| 7.52E-90 | -1.28859 | 0.297 | 0.626 | 1.35E-85 | B cells | Laptm4a |
| 1.14E-89 | -1.09167 | 0.042 | 0.432 | 2.05E-85 | B cells | Pdlim2 |
| 1.53E-89 | -1.3301 | 0.009 | 0.381 | 2.75E-85 | B cells | Fbn1 |
| 1.71E-89 | -1.1554 | 0.008 | 0.379 | 3.09E-85 | B cells | Nav1 |
| 9.97E-89 | -2.02978 | 0.012 | 0.381 | 1.80E-84 | B cells | C3 |
| 1.09E-88 | 1.193162 | 0.337 | 0.122 | 1.95E-84 | B cells | Cerk |
| 3.17E-88 | -1.00603 | 0.015 | 0.385 | 5.70E-84 | B cells | Rcn1 |
| 3.99E-88 | -1.48715 | 0.008 | 0.374 | 7.18E-84 | B cells | Pcolce2 |
| 9.26E-88 | -1.10457 | 0.003 | 0.366 | 1.67E-83 | B cells | Il11ra1 |
| 3.51E-87 | -1.09499 | 0.07 | 0.466 | 6.31E-83 | B cells | Creg1 |
| 3.58E-87 | -1.67794 | 0.003 | 0.365 | 6.44E-83 | B cells | Tnfaip2 |
| 6.95E-87 | -1.06752 | 0.052 | 0.438 | 1.25E-82 | B cells | Calu |
| 7.06E-87 | -1.40327 | 0.082 | 0.471 | 1.27E-82 | B cells | Phlda1 |
| 2.39E-86 | -1.12067 | 0.275 | 0.63 | 4.31E-82 | B cells | Bsg |
| 2.57E-86 | -1.0398 | 0.001 | 0.36 | 4.62E-82 | B cells | Mmp23 |
| 3.71E-86 | -1.12 | 0.003 | 0.362 | 6.68E-82 | B cells | Aebp1 |
| 3.88E-86 | -1.09359 | 0.049 | 0.429 | 6.98E-82 | B cells | Ugp2 |
| 4.02E-86 | -1.11362 | 0.415 | 0.805 | 7.24E-82 | B cells | Pkm |
| 4.79E-86 | -1.14946 | 0.208 | 0.585 | 8.63E-82 | B cells | Slc25a4 |
| 7.30E-86 | -1.02089 | 0.046 | 0.43 | 1.31E-81 | B cells | Vkorc1 |
| 1.13E-85 | -1.12004 | 0.012 | 0.373 | 2.03E-81 | B cells | Lama4 |
| 1.31E-85 | -1.46388 | 0.015 | 0.376 | 2.35E-81 | B cells | Hsd11b1 |
| 1.64E-85 | -1.16371 | 0.021 | 0.387 | 2.96E-81 | B cells | Socs2 |
| 2.90E-85 | -2.20944 | 0.353 | 0.65 | 5.23E-81 | B cells | Jun |
| 4.65E-85 | -1.18373 | 0.005 | 0.361 | 8.38E-81 | B cells | Angptl2 |
| 1.19E-84 | 1.470628 | 0.524 | 0.298 | 2.15E-80 | B cells | Tcp11l2 |
| 3.81E-84 | -1.41275 | 0.007 | 0.36 | 6.86E-80 | B cells | Jdp2 |
| 1.41E-82 | -1.01052 | 0.005 | 0.354 | 2.53E-78 | B cells | Fam129a |
| 1.44E-82 | -1.19619 | 0.005 | 0.355 | 2.58E-78 | B cells | Tm4sf1 |
| 1.86E-82 | -1.39695 | 0.338 | 0.68 | 3.35E-78 | B cells | Zfp36 |
| 1.94E-82 | -1.23204 | 0.013 | 0.365 | 3.49E-78 | B cells | Ppp1r14a |
| 2.25E-82 | -1.04199 | 0.005 | 0.354 | 4.05E-78 | B cells | Dpysl3 |
| 2.33E-82 | -1.0453 | 0.004 | 0.351 | 4.19E-78 | B cells | Vwa1 |
| 3.57E-82 | -1.22935 | 0.003 | 0.348 | 6.42E-78 | B cells | Htra1 |
| 4.82E-82 | -1.57479 | 0.007 | 0.353 | 8.68E-78 | B cells | Aspn |

| | | | | | | |
|---|---|---|---|---|---|---|
| 1.32E-81 | 1.005515 | 0.261 | 0.08 | 2.37E-77 | B cells | Ttpal |
| 1.59E-81 | -1.40992 | 0.088 | 0.469 | 2.87E-77 | B cells | Nfkbiz |
| 5.14E-81 | -1.03693 | 0.122 | 0.513 | 9.26E-77 | B cells | Myadm |
| 8.98E-81 | -1.23315 | 0.016 | 0.363 | 1.62E-76 | B cells | Serpine2 |
| 2.22E-80 | -1.00094 | 0.02 | 0.369 | 4.00E-76 | B cells | Cavin1 |
| 8.67E-80 | 1.182033 | 0.646 | 0.471 | 1.56E-75 | B cells | Kras |
| 1.19E-79 | -1.45374 | 0.399 | 0.735 | 2.14E-75 | B cells | Neat1 |
| 1.92E-79 | 1.262463 | 0.515 | 0.294 | 3.45E-75 | B cells | Cd55 |
| 3.05E-79 | -1.0163 | 0.06 | 0.423 | 5.50E-75 | B cells | Rras |
| 5.23E-79 | -1.75167 | 0.032 | 0.376 | 9.41E-75 | B cells | Fgl2 |
| 6.52E-79 | -1.0199 | 0.004 | 0.34 | 1.17E-74 | B cells | Ndrg2 |
| 2.35E-78 | -1.06698 | 0.004 | 0.339 | 4.23E-74 | B cells | Col4a2 |
| 3.77E-78 | -2.05876 | 0.162 | 0.498 | 6.78E-74 | B cells | Klf4 |
| 3.77E-78 | -2.93488 | 0.016 | 0.35 | 6.79E-74 | B cells | Fcer1g |
| 3.85E-78 | -1.24654 | 0.003 | 0.336 | 6.92E-74 | B cells | Gda |
| 5.38E-78 | -1.1705 | 0.005 | 0.34 | 9.69E-74 | B cells | Pla1a |
| 1.49E-77 | -2.37112 | 0.032 | 0.369 | 2.69E-73 | B cells | Gm12840 |
| 4.20E-77 | -1.52784 | 0.013 | 0.347 | 7.56E-73 | B cells | Spon2 |
| 4.85E-77 | 1.049691 | 0.493 | 0.246 | 8.73E-73 | B cells | Crlf3 |
| 6.23E-77 | -1.29418 | 0.007 | 0.338 | 1.12E-72 | B cells | Eln |
| 2.41E-76 | -1.59598 | 0.057 | 0.4 | 4.33E-72 | B cells | Trib1 |
| 3.88E-76 | -1.10138 | 0.267 | 0.661 | 6.98E-72 | B cells | Ifngr1 |
| 1.18E-75 | -1.31756 | 0.012 | 0.343 | 2.13E-71 | B cells | Gfpt2 |
| 1.28E-75 | -1.43373 | 0.008 | 0.334 | 2.30E-71 | B cells | Nbl1 |
| 7.25E-75 | -1.79036 | 0.267 | 0.601 | 1.31E-70 | B cells | Hspa1b |
| 9.51E-75 | -1.49028 | 0.054 | 0.401 | 1.71E-70 | B cells | Id2 |
| 1.18E-73 | -1.4812 | 0.004 | 0.323 | 2.13E-69 | B cells | Emilin2 |
| 1.64E-73 | -1.10203 | 0.003 | 0.321 | 2.96E-69 | B cells | Sfrp1 |
| 1.92E-73 | 1.119076 | 0.359 | 0.152 | 3.45E-69 | B cells | Nfatc3 |
| 4.39E-73 | -1.21883 | 0.206 | 0.54 | 7.90E-69 | B cells | Sptbn1 |
| 9.30E-73 | -1.2161 | 0.007 | 0.326 | 1.67E-68 | B cells | Gsta3 |
| 3.80E-72 | -1.05907 | 0.188 | 0.538 | 6.85E-68 | B cells | Ybx3 |
| 5.01E-72 | -1.48511 | 0.034 | 0.358 | 9.02E-68 | B cells | Glrx |
| 5.64E-72 | 1.285236 | 0.509 | 0.315 | 1.02E-67 | B cells | Pold4 |
| 6.71E-72 | -1.10872 | 0.025 | 0.352 | 1.21E-67 | B cells | Plin2 |
| 2.91E-71 | -2.5637 | 0.032 | 0.349 | 5.24E-67 | B cells | Cxcl1 |
| 2.94E-71 | -1.10829 | 0.126 | 0.494 | 5.29E-67 | B cells | Ndel1 |
| 3.12E-71 | -1.05623 | 0.159 | 0.528 | 5.62E-67 | B cells | Dbi |
| 7.53E-71 | -1.17859 | 0.013 | 0.327 | 1.36E-66 | B cells | Bag3 |
| 9.55E-71 | -1.06459 | 0.22 | 0.6 | 1.72E-66 | B cells | Ptp4a1 |
| 9.65E-71 | 1.03844 | 0.646 | 0.5 | 1.74E-66 | B cells | Polr1d |
| 1.02E-70 | -1.05756 | 0.007 | 0.317 | 1.84E-66 | B cells | Fxyd6 |
| 4.86E-70 | -2.10391 | 0.015 | 0.325 | 8.75E-66 | B cells | Cyr61 |
| 2.02E-69 | -1.0893 | 0.001 | 0.305 | 3.64E-65 | B cells | Fibin |
| 1.92E-68 | -1.05572 | 0.015 | 0.324 | 3.46E-64 | B cells | Col5a3 |
| 3.02E-68 | -2.52575 | 0.005 | 0.306 | 5.44E-64 | B cells | Slc16a3 |
| 2.26E-66 | -1.74825 | 0.101 | 0.409 | 4.06E-62 | B cells | Selplg |
| 2.26E-66 | -1.02554 | 0.004 | 0.298 | 4.06E-62 | B cells | Gas6 |
| 4.00E-66 | -1.07168 | 0.005 | 0.301 | 7.20E-62 | B cells | Adamts1 |
| 1.76E-64 | -2.48859 | 0.013 | 0.302 | 3.17E-60 | B cells | Lst1 |
| 1.90E-64 | -2.70537 | 0.074 | 0.371 | 3.42E-60 | B cells | Mxd1 |
| 3.39E-64 | -1.08512 | 0.064 | 0.384 | 6.10E-60 | B cells | Card19 |
| 2.16E-63 | 1.082452 | 0.275 | 0.103 | 3.89E-59 | B cells | Dmxl1 |
| 1.01E-62 | -1.14887 | 0.007 | 0.29 | 1.82E-58 | B cells | Tppp3 |

| | | | | | | |
|---|---|---|---|---|---|---|
| 2.97E-62 | -1.5795 | 0.176 | 0.481 | 5.35E-58 | B cells | Id3 |
| 4.11E-62 | -1.66738 | 0.02 | 0.307 | 7.40E-58 | B cells | Mt2 |
| 4.11E-61 | -1.75181 | 0.013 | 0.294 | 7.41E-57 | B cells | Tgfbi |
| 3.07E-60 | -1.03249 | 0.005 | 0.279 | 5.53E-56 | B cells | Sirpa |
| 4.42E-60 | -1.12907 | 0.073 | 0.381 | 7.95E-56 | B cells | Uap1 |
| 5.22E-60 | -1.7337 | 0.057 | 0.35 | 9.39E-56 | B cells | Rnf149 |
| 6.68E-60 | 1.24635 | 0.455 | 0.273 | 1.20E-55 | B cells | Got1 |
| 7.89E-60 | -1.0205 | 0.007 | 0.281 | 1.42E-55 | B cells | Fmo2 |
| 1.02E-59 | -3.64903 | 0.097 | 0.366 | 1.84E-55 | B cells | Tyrobp |
| 1.12E-59 | -1.30352 | 0.239 | 0.537 | 2.01E-55 | B cells | Fosb |
| 1.14E-59 | -2.52194 | 0.024 | 0.297 | 2.05E-55 | B cells | Alox5ap |
| 2.26E-59 | -1.22826 | 0.069 | 0.369 | 4.07E-55 | B cells | Nudt4 |
| 2.87E-59 | 1.128316 | 0.383 | 0.199 | 5.17E-55 | B cells | Egln2 |
| 5.34E-59 | -1.28505 | 0.007 | 0.276 | 9.62E-55 | B cells | Ackr3 |
| 5.05E-58 | -1.15066 | 0.072 | 0.368 | 9.09E-54 | B cells | Efhd2 |
| 6.08E-58 | -1.23008 | 0.008 | 0.276 | 1.09E-53 | B cells | Tgm2 |
| 3.31E-57 | -1.26208 | 0.052 | 0.338 | 5.96E-53 | B cells | Ugdh |
| 4.20E-57 | -1.10634 | 0.004 | 0.266 | 7.55E-53 | B cells | Hmox1 |
| 2.20E-56 | 1.156222 | 0.531 | 0.364 | 3.95E-52 | B cells | 4930523C07Rik |
| 2.22E-56 | -1.58506 | 0.016 | 0.279 | 4.00E-52 | B cells | Cd3e |
| 2.34E-56 | -3.00519 | 0.02 | 0.281 | 4.21E-52 | B cells | Ccl6 |
| 6.15E-56 | -1.58287 | 0.016 | 0.278 | 1.11E-51 | B cells | Ctgf |
| 6.78E-56 | -2.75383 | 0.005 | 0.261 | 1.22E-51 | B cells | Hp |
| 2.50E-55 | -1.08201 | 0.013 | 0.276 | 4.50E-51 | B cells | Thy1 |
| 3.12E-54 | -1.88872 | 0.019 | 0.277 | 5.62E-50 | B cells | Isg15 |
| 5.18E-54 | -2.94698 | 0.017 | 0.272 | 9.33E-50 | B cells | Ccrl2 |
| 1.14E-53 | -2.46266 | 0.008 | 0.257 | 2.06E-49 | B cells | Csf3r |
| 1.00E-52 | 1.012032 | 0.687 | 0.589 | 1.80E-48 | B cells | Hspe1 |
| 3.32E-52 | -1.11854 | 0.024 | 0.278 | 5.98E-48 | B cells | Ly6c1 |
| 4.48E-52 | -3.4649 | 0.024 | 0.271 | 8.06E-48 | B cells | Il1b |
| 4.69E-52 | -1.11872 | 0.267 | 0.562 | 8.43E-48 | B cells | Hspa1a |
| 4.99E-52 | -2.82179 | 0.008 | 0.25 | 8.99E-48 | B cells | Hdc |
| 1.25E-51 | -3.10286 | 0.016 | 0.259 | 2.25E-47 | B cells | Il1r2 |
| 1.09E-50 | -1.34035 | 0.414 | 0.553 | 1.96E-46 | B cells | Cd81 |
| 5.03E-50 | -6.66679 | 0.216 | 0.477 | 9.06E-46 | B cells | S100a8 |
| 5.19E-50 | -1.52129 | 0.345 | 0.661 | 9.34E-46 | B cells | Taldo1 |
| 1.37E-49 | -5.46505 | 0.088 | 0.337 | 2.48E-45 | B cells | Retnlg |
| 5.16E-48 | -6.86536 | 0.245 | 0.512 | 9.29E-44 | B cells | S100a9 |
| 1.90E-47 | -4.10271 | 0.081 | 0.317 | 3.43E-43 | B cells | Lyz2 |
| 3.65E-47 | -2.05444 | 0.054 | 0.291 | 6.56E-43 | B cells | Lmnb1 |
| 9.98E-46 | -1.04565 | 0.033 | 0.264 | 1.80E-41 | B cells | Vsir |
| 5.41E-45 | -1.34166 | 0.099 | 0.337 | 9.74E-41 | B cells | Emb |
| 8.21E-45 | -1.54257 | 0.027 | 0.251 | 1.48E-40 | B cells | Stx11 |
| 1.45E-44 | -1.12857 | 0.034 | 0.265 | 2.61E-40 | B cells | Plk3 |
| 6.64E-44 | -1.46469 | 0.09 | 0.327 | 1.19E-39 | B cells | Cd44 |
| 9.12E-42 | -1.02036 | 0.182 | 0.452 | 1.64E-37 | B cells | Gadd45b |
| 6.54E-39 | 1.073695 | 0.467 | 0.344 | 1.18E-34 | B cells | Tmem123 |
| 3.07E-38 | -1.13771 | 0.119 | 0.353 | 5.52E-34 | B cells | Gadd45g |
| 6.85E-38 | -1.97378 | 0.086 | 0.292 | 1.23E-33 | B cells | Pglyrp1 |
| 1.47E-37 | -1.19736 | 0.106 | 0.32 | 2.65E-33 | B cells | Hcst |
| 1.04E-34 | -1.5465 | 0.11 | 0.313 | 1.87E-30 | B cells | Lgals3 |
| 1.06E-34 | -1.06902 | 0.358 | 0.588 | 1.91E-30 | B cells | Ifi27l2a |
| 2.90E-27 | -1.23287 | 0.145 | 0.331 | 5.22E-23 | B cells | Ets2 |
| 7.04E-24 | -1.2431 | 0.138 | 0.294 | 1.27E-19 | B cells | Samsn1 |

| | | | | | | |
|---|---|---|---|---|---|---|
| 3.14E-23 | -1.7792 | 0.696 | 0.668 | 5.66E-19 | B cells | Hbb-bs |
| 8.99E-23 | 1.012948 | 0.373 | 0.288 | 1.62E-18 | B cells | Foxo1 |
| 5.69E-07 | -1.00855 | 0.361 | 0.392 | 0.010235 | B cells | Lcp1 |
| 0 | 4.866536 | 0.981 | 0.102 | 0 | NKT | Ccl5 |
| 0 | 3.444586 | 0.989 | 0.104 | 0 | NKT | Nkg7 |
| 0 | 3.388602 | 0.845 | 0.016 | 0 | NKT | Cd8a |
| 0 | 3.369274 | 0.638 | 0.046 | 0 | NKT | Gzmb |
| 0 | 3.346079 | 0.85 | 0.022 | 0 | NKT | Cd8b1 |
| 0 | 3.234701 | 0.968 | 0.136 | 0 | NKT | Cd3g |
| 0 | 2.950438 | 0.962 | 0.148 | 0 | NKT | Ms4a4b |
| 0 | 2.688342 | 0.725 | 0.053 | 0 | NKT | Ctla2a |
| 0 | 2.664026 | 0.953 | 0.139 | 0 | NKT | Trbc2 |
| 0 | 2.614352 | 0.826 | 0.074 | 0 | NKT | Ctsw |
| 0 | 2.604025 | 0.907 | 0.252 | 0 | NKT | Thy1 |
| 0 | 2.556567 | 0.549 | 0.034 | 0 | NKT | Pdcd1 |
| 0 | 2.526412 | 0.644 | 0.035 | 0 | NKT | Klrc1 |
| 0 | 2.522349 | 0.843 | 0.179 | 0 | NKT | Dusp2 |
| 0 | 2.496712 | 0.864 | 0.108 | 0 | NKT | Trac |
| 0 | 2.488628 | 0.822 | 0.117 | 0 | NKT | Rgs1 |
| 0 | 2.477297 | 0.657 | 0.038 | 0 | NKT | Cxcr6 |
| 0 | 2.428367 | 0.472 | 0.03 | 0 | NKT | Ifng |
| 0 | 2.34486 | 0.96 | 0.254 | 0 | NKT | Cd3e |
| 0 | 2.327305 | 0.943 | 0.211 | 0 | NKT | Cd3d |
| 0 | 2.314645 | 0.873 | 0.152 | 0 | NKT | Ms4a6b |
| 0 | 2.214397 | 0.839 | 0.111 | 0 | NKT | Lat |
| 0 | 2.20947 | 0.727 | 0.114 | 0 | NKT | Ly6c2 |
| 0 | 2.201438 | 0.799 | 0.148 | 0 | NKT | H2-Q7 |
| 0 | 2.175917 | 0.837 | 0.131 | 0 | NKT | Lck |
| 0 | 2.084132 | 0.739 | 0.114 | 0 | NKT | Cd28 |
| 0 | 1.875322 | 0.566 | 0.04 | 0 | NKT | Klrk1 |
| 0 | 1.874198 | 0.549 | 0.065 | 0 | NKT | Gpr183 |
| 0 | 1.858591 | 0.758 | 0.13 | 0 | NKT | Ptpn22 |
| 0 | 1.851796 | 0.631 | 0.093 | 0 | NKT | Sh2d2a |
| 0 | 1.826719 | 0.528 | 0.025 | 0 | NKT | Klrc2 |
| 0 | 1.743772 | 0.502 | 0.038 | 0 | NKT | Fasl |
| 0 | 1.676626 | 0.559 | 0.069 | 0 | NKT | Icos |
| 0 | 1.664085 | 0.532 | 0.051 | 0 | NKT | Klrd1 |
| 0 | 1.609403 | 0.475 | 0.048 | 0 | NKT | Epsti1 |
| 0 | 1.58578 | 0.506 | 0.059 | 0 | NKT | Cd6 |
| 0 | 1.585528 | 0.494 | 0.034 | 0 | NKT | Cxcr3 |
| 0 | 1.515593 | 0.511 | 0.057 | 0 | NKT | Klk8 |
| 0 | 1.479656 | 0.47 | 0.041 | 0 | NKT | Cst7 |
| ######## | 2.394308 | 0.841 | 0.201 | ######## | NKT | S100a4 |
| ######## | 1.060002 | 0.322 | 0.025 | ######## | NKT | Themis |
| ######## | 1.784496 | 0.735 | 0.139 | ######## | NKT | Ptprcap |
| ######## | 1.519589 | 0.5 | 0.061 | ######## | NKT | Bcl2a1d |
| ######## | 1.6465 | 0.646 | 0.111 | ######## | NKT | Skap1 |
| ######## | 1.832958 | 0.684 | 0.127 | ######## | NKT | Bcl2a1b |
| ######## | 1.02433 | 0.269 | 0.018 | ######## | NKT | Cd226 |
| ######## | 1.539608 | 0.578 | 0.09 | ######## | NKT | Itk |
| ######## | 1.774806 | 0.587 | 0.094 | ######## | NKT | Cd69 |
| ######## | 1.3677 | 0.456 | 0.056 | ######## | NKT | Bcl11b |
| ######## | 2.254469 | 0.718 | 0.156 | ######## | NKT | Nr4a2 |
| ######## | 2.119448 | 0.68 | 0.128 | ######## | NKT | Ccl4 |

| | | | | | | |
|---|---|---|---|---|---|---|
| ######## | 2.00715 | 0.932 | 0.298 | ######## | NKT | Hcst |
| ######## | 1.642521 | 0.725 | 0.143 | ######## | NKT | Cd2 |
| ######## | 1.861899 | 0.905 | 0.261 | ######## | NKT | Ptpn18 |
| ######## | 1.569595 | 0.576 | 0.096 | ######## | NKT | Cd27 |
| ######## | 1.665675 | 0.752 | 0.181 | ######## | NKT | 1-Sep |
| ######## | 1.797359 | 0.697 | 0.157 | ######## | NKT | Hopx |
| ######## | 2.460721 | 0.987 | 0.717 | ######## | NKT | H2-K1 |
| ######## | 1.585037 | 0.606 | 0.118 | ######## | NKT | H2-Q6 |
| ######## | 1.232081 | 0.559 | 0.09 | ######## | NKT | Il2rb |
| ######## | 1.394598 | 0.604 | 0.111 | ######## | NKT | Dok2 |
| ######## | 1.528643 | 0.619 | 0.117 | ######## | NKT | Gimap3 |
| ######## | 2.190082 | 0.949 | 0.441 | ######## | NKT | AW112010 |
| ######## | 1.245512 | 0.417 | 0.056 | ######## | NKT | Grap2 |
| ######## | 1.35803 | 0.479 | 0.077 | ######## | NKT | Gm8369 |
| ######## | 1.53457 | 0.633 | 0.139 | ######## | NKT | Itgb7 |
| ######## | 1.094955 | 0.324 | 0.036 | ######## | NKT | Tbx21 |
| ######## | 1.372536 | 1 | 0.982 | ######## | NKT | H2-D1 |
| ######## | 1.333063 | 0.462 | 0.08 | ######## | NKT | Cd48 |
| ######## | 1.141306 | 0.456 | 0.076 | ######## | NKT | Cd247 |
| ######## | 1.145047 | 0.354 | 0.047 | ######## | NKT | Ccr5 |
| ######## | 1.1948 | 0.403 | 0.062 | ######## | NKT | Zap70 |
| ######## | 1.561697 | 0.744 | 0.224 | ######## | NKT | Tnfaip3 |
| ######## | 1.68462 | 0.831 | 0.274 | ######## | NKT | Ltb |
| ######## | 1.843573 | 0.867 | 0.412 | ######## | NKT | Psmb8 |
| ######## | -2.09003 | 0.922 | 0.99 | ######## | NKT | Ftl1 |
| ######## | 1.672501 | 0.998 | 0.481 | ######## | NKT | Cd52 |
| ######## | 1.12914 | 0.352 | 0.048 | ######## | NKT | Ctla4 |
| ######## | 1.207376 | 0.331 | 0.044 | ######## | NKT | Gimap7 |
| ######## | 1.298884 | 0.288 | 0.034 | ######## | NKT | Serpina3g |
| ######## | 1.249473 | 1 | 0.815 | ######## | NKT | Rps15a |
| ######## | 1.445097 | 0.576 | 0.14 | ######## | NKT | B4galnt1 |
| ######## | 1.166264 | 1 | 0.869 | ######## | NKT | Rps13 |
| ######## | 2.394351 | 0.5 | 0.108 | ######## | NKT | Trbc1 |
| ######## | -3.32554 | 0.089 | 0.772 | ######## | NKT | Ifitm2 |
| ######## | 1.125544 | 0.998 | 0.841 | ######## | NKT | Rpl19 |
| ######## | 1.43686 | 0.82 | 0.294 | ######## | NKT | Ets1 |
| ######## | 1.541379 | 0.97 | 0.662 | ######## | NKT | S100a10 |
| ######## | 1.280462 | 0.992 | 0.905 | ######## | NKT | B2m |
| ######## | 1.391541 | 0.934 | 0.396 | ######## | NKT | Laptm5 |
| ######## | 1.139644 | 1 | 0.867 | ######## | NKT | Rps24 |
| ######## | 1.189544 | 0.439 | 0.08 | ######## | NKT | Ccr2 |
| ######## | 1.27343 | 0.508 | 0.108 | ######## | NKT | Gimap4 |
| ######## | 1.355591 | 0.983 | 0.451 | ######## | NKT | Rac2 |
| ######## | 1.064705 | 1 | 0.869 | ######## | NKT | Rps3 |
| ######## | 1.04436 | 0.375 | 0.065 | ######## | NKT | Rasal3 |
| ######## | 1.157221 | 0.356 | 0.058 | ######## | NKT | Il21r |
| ######## | -3.00324 | 0.475 | 0.853 | ######## | NKT | Cst3 |
| ######## | -3.37004 | 0.093 | 0.724 | ######## | NKT | Ifitm3 |
| ######## | 1.043467 | 1 | 0.898 | ######## | NKT | Rps10 |
| ######## | 1.063443 | 0.998 | 0.851 | ######## | NKT | Rps11 |
| ######## | 1.042527 | 0.379 | 0.068 | ######## | NKT | Traf1 |
| ######## | 1.016478 | 0.28 | 0.039 | ######## | NKT | Phf11b |
| ######## | 1.11661 | 0.388 | 0.071 | ######## | NKT | 1700097N02Rik |
| ######## | 1.713698 | 0.583 | 0.174 | ######## | NKT | Gramd3 |

| | | | | | | |
|---|---|---|---|---|---|---|
| ######## | 1.071327 | 0.331 | 0.053 | ######## | NKT | Gpr171 |
| ######## | 1.205695 | 0.542 | 0.133 | ######## | NKT | Gimap1 |
| ######## | 1.236516 | 0.364 | 0.066 | ######## | NKT | Tagap |
| ######## | 1.144946 | 0.398 | 0.077 | ######## | NKT | Runx3 |
| ######## | 1.388037 | 0.873 | 0.511 | ######## | NKT | Mbnl1 |
| ######## | 1.31214 | 0.65 | 0.202 | ######## | NKT | Dusp5 |
| ######## | 1.132088 | 0.992 | 0.788 | ######## | NKT | Rpl27 |
| ######## | 1.357858 | 0.555 | 0.16 | ######## | NKT | Gng2 |
| ######## | 1.221346 | 0.9 | 0.387 | ######## | NKT | Selplg |
| ######## | -2.62397 | 0.011 | 0.649 | ######## | NKT | Aldh2 |
| ######## | 1.124356 | 0.398 | 0.081 | ######## | NKT | S1pr4 |
| ######## | 1.385747 | 0.926 | 0.686 | ######## | NKT | Shisa5 |
| ######## | 1.065627 | 0.394 | 0.083 | ######## | NKT | Acp5 |
| ######## | -2.58855 | 0.047 | 0.658 | ######## | NKT | Timp2 |
| ######## | 1.189161 | 0.278 | 0.045 | ######## | NKT | Abcb1b |
| ######## | 1.166252 | 1 | 0.764 | ######## | NKT | Tmsb10 |
| ######## | 1.167536 | 0.422 | 0.098 | ######## | NKT | Itga4 |
| ######## | 1.561203 | 0.782 | 0.38 | ######## | NKT | Id2 |
| ######## | -4.1197 | 0.123 | 0.667 | ######## | NKT | Cebpd |
| ######## | 1.319374 | 0.636 | 0.243 | ######## | NKT | Psmb9 |
| ######## | 1.039067 | 0.411 | 0.093 | ######## | NKT | Rhoh |
| ######## | -2.96618 | 0.023 | 0.614 | ######## | NKT | Cd63 |
| ######## | 1.038463 | 0.513 | 0.141 | ######## | NKT | Tbc1d10c |
| ######## | -5.63854 | 0.396 | 0.779 | ######## | NKT | Gsn |
| ######## | 1.451364 | 0.907 | 0.628 | ######## | NKT | Zfp36l2 |
| ######## | 1.090364 | 0.633 | 0.208 | ######## | NKT | Itgal |
| ######## | 1.1197 | 0.934 | 0.755 | ######## | NKT | Pabpc1 |
| ######## | 1.195126 | 0.888 | 0.585 | ######## | NKT | Sub1 |
| ######## | -2.66195 | 0.051 | 0.614 | ######## | NKT | Cd9 |
| ######## | 1.222841 | 0.922 | 0.715 | ######## | NKT | Sh3bgrl3 |
| ######## | -2.95591 | 0.036 | 0.589 | ######## | NKT | Ctsl |
| ######## | 1.126814 | 0.792 | 0.341 | ######## | NKT | Vps37b |
| ######## | -3.2009 | 0.059 | 0.594 | ######## | NKT | Pi16 |
| ######## | -2.35416 | 0.049 | 0.589 | 1.61E-99 | NKT | Selenop |
| ######## | 1.255135 | 0.845 | 0.486 | 3.69E-99 | NKT | Ucp2 |
| ######## | 1.005468 | 0.432 | 0.112 | 6.66E-99 | NKT | Il2rg |
| ######## | -2.22892 | 0.157 | 0.679 | 1.19E-97 | NKT | Zfp36 |
| ######## | -1.92803 | 0.008 | 0.552 | 4.00E-97 | NKT | App |
| ######## | 1.163454 | 0.447 | 0.133 | 6.98E-97 | NKT | Tap1 |
| ######## | -2.14532 | 0.011 | 0.555 | 9.66E-97 | NKT | Mgst1 |
| ######## | 1.243121 | 0.627 | 0.263 | 1.20E-95 | NKT | Zdhhc18 |
| ######## | -2.7283 | 0.021 | 0.555 | 1.70E-95 | NKT | Crispld2 |
| 2.40E-99 | -2.87122 | 0.036 | 0.56 | 4.32E-95 | NKT | Cd81 |
| 6.06E-96 | -2.87543 | 0.038 | 0.552 | 1.09E-91 | NKT | Igfbp4 |
| 1.96E-94 | -1.90054 | 0.006 | 0.527 | 3.53E-90 | NKT | Marcks |
| 4.09E-94 | -4.27754 | 0.106 | 0.582 | 7.36E-90 | NKT | Igfbp7 |
| 7.00E-94 | 1.144344 | 0.456 | 0.146 | 1.26E-89 | NKT | Grap |
| 1.41E-92 | -3.73947 | 0.047 | 0.543 | 2.54E-88 | NKT | Sparcl1 |
| 2.23E-92 | -2.37053 | 0.044 | 0.547 | 4.02E-88 | NKT | Zbtb20 |
| 1.25E-91 | -3.46149 | 0.023 | 0.525 | 2.26E-87 | NKT | Serping1 |
| 1.86E-91 | -4.09732 | 0.07 | 0.553 | 3.35E-87 | NKT | Bgn |
| 2.94E-91 | -2.44279 | 0.015 | 0.521 | 5.30E-87 | NKT | Gstm1 |
| 6.39E-91 | 1.009645 | 0.625 | 0.232 | 1.15E-86 | NKT | Fyb |
| 1.62E-89 | -3.41607 | 0.042 | 0.532 | 2.91E-85 | NKT | Lum |

| | | | | | | |
|---|---|---|---|---|---|---|
| 3.74E-89 | -3.71747 | 0.064 | 0.544 | 6.73E-85 | NKT | Htra3 |
| 8.05E-89 | -3.63784 | 0.036 | 0.524 | 1.45E-84 | NKT | Clec3b |
| 1.25E-88 | -3.93197 | 0.059 | 0.538 | 2.25E-84 | NKT | Sparc |
| 3.24E-88 | -5.57157 | 0.267 | 0.65 | 5.83E-84 | NKT | Dcn |
| 5.34E-88 | -2.47721 | 0.019 | 0.513 | 9.61E-84 | NKT | Rarres2 |
| 1.40E-87 | -3.16791 | 0.025 | 0.514 | 2.52E-83 | NKT | Gpx3 |
| 4.55E-87 | -2.06111 | 0.165 | 0.631 | 8.19E-83 | NKT | Rhob |
| 9.48E-87 | -1.46226 | 0.472 | 0.81 | 1.71E-82 | NKT | Psap |
| 1.87E-86 | -2.73255 | 0.03 | 0.513 | 3.37E-82 | NKT | Cygb |
| 4.12E-86 | -4.55948 | 0.136 | 0.579 | 7.42E-82 | NKT | Mgp |
| 4.75E-86 | -3.0966 | 0.03 | 0.523 | 8.56E-82 | NKT | G0s2 |
| 6.21E-86 | -3.25487 | 0.036 | 0.514 | 1.12E-81 | NKT | Col1a2 |
| 7.42E-86 | -2.14793 | 0.011 | 0.499 | 1.33E-81 | NKT | Lhfp |
| 8.02E-86 | -2.89895 | 0.032 | 0.513 | 1.44E-81 | NKT | Pcolce |
| 8.96E-86 | -1.90752 | 0.119 | 0.607 | 1.61E-81 | NKT | Anxa1 |
| 1.71E-84 | -2.05065 | 0.015 | 0.497 | 3.09E-80 | NKT | Lrp1 |
| 2.52E-84 | -3.14567 | 0.023 | 0.5 | 4.53E-80 | NKT | Col1a1 |
| 3.19E-84 | -1.44583 | 0.102 | 0.591 | 5.73E-80 | NKT | Lamp2 |
| 4.47E-84 | -2.86924 | 0.061 | 0.524 | 8.05E-80 | NKT | Ltbp4 |
| 5.69E-84 | -3.15086 | 0.025 | 0.5 | 1.02E-79 | NKT | Serpinh1 |
| 1.54E-83 | 1.198653 | 0.708 | 0.405 | 2.77E-79 | NKT | Abracl |
| 2.52E-83 | -2.55557 | 0.475 | 0.783 | 4.54E-79 | NKT | Fos |
| 3.75E-83 | -1.89775 | 0.068 | 0.539 | 6.75E-79 | NKT | Sptbn1 |
| 5.51E-83 | -2.51424 | 0.023 | 0.497 | 9.92E-79 | NKT | Cd34 |
| 1.47E-82 | -2.49374 | 0.008 | 0.484 | 2.64E-78 | NKT | Serpinf1 |
| 4.21E-82 | -2.08308 | 0.006 | 0.482 | 7.58E-78 | NKT | Pmp22 |
| 7.92E-82 | -2.56458 | 0.017 | 0.487 | 1.43E-77 | NKT | Col6a1 |
| 1.13E-81 | -1.84928 | 0.03 | 0.502 | 2.03E-77 | NKT | Rnase4 |
| 1.15E-81 | -2.42915 | 0.028 | 0.494 | 2.07E-77 | NKT | Mmp2 |
| 1.62E-81 | -3.57303 | 0.021 | 0.49 | 2.91E-77 | NKT | Lpl |
| 4.27E-81 | -3.49628 | 0.057 | 0.512 | 7.68E-77 | NKT | Col3a1 |
| 8.51E-81 | -1.87906 | 0.019 | 0.487 | 1.53E-76 | NKT | Tcf4 |
| 9.86E-81 | 1.082888 | 0.5 | 0.182 | 1.78E-76 | NKT | D16Ertd472e |
| 1.16E-80 | -3.02512 | 0.03 | 0.494 | 2.09E-76 | NKT | Fbln1 |
| 1.97E-80 | -2.48067 | 0.011 | 0.476 | 3.55E-76 | NKT | Col6a2 |
| 2.06E-80 | -2.46233 | 0.008 | 0.476 | 3.71E-76 | NKT | Fbln2 |
| 3.11E-80 | -1.74121 | 0.013 | 0.481 | 5.60E-76 | NKT | Cd302 |
| 3.16E-80 | -2.51404 | 0.008 | 0.475 | 5.68E-76 | NKT | Ccdc80 |
| 4.78E-80 | -2.45242 | 0.025 | 0.49 | 8.60E-76 | NKT | Cfh |
| 1.48E-79 | -2.30693 | 0.015 | 0.477 | 2.67E-75 | NKT | Fstl1 |
| 6.59E-79 | -2.00481 | 0.008 | 0.47 | 1.19E-74 | NKT | Nfix |
| 3.36E-78 | -2.15816 | 0.028 | 0.484 | 6.04E-74 | NKT | Dpt |
| 7.67E-78 | -2.17054 | 0.013 | 0.469 | 1.38E-73 | NKT | Nid1 |
| 1.52E-77 | -1.99172 | 0.013 | 0.471 | 2.73E-73 | NKT | Rbp1 |
| 1.94E-77 | -2.46273 | 0.011 | 0.465 | 3.50E-73 | NKT | Col15a1 |
| 2.07E-77 | 1.03885 | 0.434 | 0.153 | 3.73E-73 | NKT | 6-Sep |
| 8.85E-77 | -1.78969 | 0.011 | 0.466 | 1.59E-72 | NKT | Sdc2 |
| 2.34E-76 | -1.80518 | 0.517 | 0.831 | 4.22E-72 | NKT | Dusp1 |
| 6.97E-76 | -2.0023 | 0.013 | 0.462 | 1.26E-71 | NKT | Hspg2 |
| 9.49E-76 | 1.236555 | 0.47 | 0.177 | 1.71E-71 | NKT | Sipa1l1 |
| 1.02E-75 | -1.63748 | 0.242 | 0.622 | 1.83E-71 | NKT | Laptm4a |
| 1.26E-75 | -1.83408 | 0.061 | 0.505 | 2.27E-71 | NKT | Selenom |
| 1.58E-75 | -1.43277 | 0.049 | 0.509 | 2.84E-71 | NKT | Myadm |
| 1.70E-75 | 1.175463 | 0.701 | 0.435 | 3.07E-71 | NKT | Saraf |

| | | | | | | |
|---|---|---|---|---|---|---|
| 6.27E-75 | -1.63544 | 0.049 | 0.498 | 1.13E-70 | NKT | Nenf |
| 1.10E-74 | -2.56759 | 0.028 | 0.47 | 1.97E-70 | NKT | Smoc2 |
| 1.10E-74 | -2.3298 | 0.034 | 0.48 | 1.98E-70 | NKT | Atf3 |
| 1.53E-74 | -1.67751 | 0.008 | 0.458 | 2.75E-70 | NKT | Nfib |
| 1.74E-74 | -1.87121 | 0.013 | 0.458 | 3.13E-70 | NKT | Loxl1 |
| 5.37E-74 | -2.01356 | 0.008 | 0.451 | 9.66E-70 | NKT | Col4a1 |
| 5.55E-73 | -1.62872 | 0.011 | 0.45 | 9.98E-69 | NKT | Rhoj |
| 9.43E-73 | -2.88245 | 0.163 | 0.555 | 1.70E-68 | NKT | Egr1 |
| 1.19E-72 | -1.83536 | 0.015 | 0.454 | 2.14E-68 | NKT | Prelp |
| 1.66E-72 | -1.8973 | 0.038 | 0.476 | 3.00E-68 | NKT | Axl |
| 2.27E-72 | -1.68086 | 0.019 | 0.46 | 4.09E-68 | NKT | Mxra8 |
| 2.29E-72 | -1.94543 | 0.004 | 0.44 | 4.13E-68 | NKT | Pcsk6 |
| 2.30E-72 | -1.73913 | 0.006 | 0.443 | 4.14E-68 | NKT | Gpm6b |
| 2.33E-72 | -1.74609 | 0.017 | 0.455 | 4.20E-68 | NKT | Abca8a |
| 9.50E-72 | 1.002124 | 0.843 | 0.642 | 1.71E-67 | NKT | Ifngr1 |
| 3.61E-71 | -1.80589 | 0.021 | 0.451 | 6.49E-67 | NKT | Tnxb |
| 4.69E-71 | -1.83838 | 0.013 | 0.444 | 8.44E-67 | NKT | Plpp3 |
| 6.28E-71 | -1.64795 | 0.038 | 0.47 | 1.13E-66 | NKT | Rcn3 |
| 1.04E-70 | -3.78405 | 0.144 | 0.547 | 1.87E-66 | NKT | Mt1 |
| 1.13E-70 | 1.022167 | 0.767 | 0.566 | 2.03E-66 | NKT | Cox5a |
| 3.59E-70 | -2.08315 | 0.013 | 0.441 | 6.46E-66 | NKT | Gas1 |
| 5.21E-70 | -1.56278 | 0.013 | 0.44 | 9.37E-66 | NKT | Cpq |
| 2.08E-69 | -1.7293 | 0.032 | 0.462 | 3.74E-65 | NKT | Slc43a3 |
| 1.26E-68 | 1.072046 | 0.544 | 0.237 | 2.26E-64 | NKT | Itpkb |
| 1.96E-68 | -1.88043 | 0.03 | 0.452 | 3.53E-64 | NKT | Ecm1 |
| 2.55E-68 | -2.50425 | 0.081 | 0.495 | 4.58E-64 | NKT | Klf4 |
| 3.04E-68 | -1.32326 | 0.095 | 0.528 | 5.47E-64 | NKT | Dstn |
| 3.45E-68 | -1.43082 | 0.004 | 0.425 | 6.21E-64 | NKT | Bicc1 |
| 3.53E-68 | -2.69631 | 0.564 | 0.846 | 6.36E-64 | NKT | Cebpb |
| 6.12E-68 | -1.3481 | 0.028 | 0.452 | 1.10E-63 | NKT | Fcgrt |
| 6.55E-68 | 1.03438 | 0.591 | 0.294 | 1.18E-63 | NKT | Sp100 |
| 6.86E-68 | -1.67093 | 0.07 | 0.494 | 1.23E-63 | NKT | Klf9 |
| 8.45E-68 | -1.77814 | 0.013 | 0.43 | 1.52E-63 | NKT | Lamb1 |
| 1.09E-67 | -1.60619 | 0.013 | 0.432 | 1.95E-63 | NKT | Olfml3 |
| 1.13E-67 | -2.67198 | 0.307 | 0.647 | 2.03E-63 | NKT | Jun |
| 1.21E-67 | 1.055069 | 0.39 | 0.139 | 2.17E-63 | NKT | H2-T22 |
| 1.35E-67 | -2.17462 | 0.059 | 0.479 | 2.43E-63 | NKT | Id3 |
| 1.59E-67 | 1.039593 | 0.612 | 0.312 | 2.87E-63 | NKT | Zc3hav1 |
| 1.98E-67 | -1.44502 | 0 | 0.414 | 3.56E-63 | NKT | Lama2 |
| 2.35E-67 | -1.82816 | 0.112 | 0.527 | 4.23E-63 | NKT | Cdkn1a |
| 2.88E-67 | -1.62437 | 0.008 | 0.424 | 5.19E-63 | NKT | Tcf21 |
| 4.63E-67 | -1.52232 | 0.006 | 0.42 | 8.33E-63 | NKT | Ptgis |
| 1.37E-66 | -1.47603 | 0.008 | 0.42 | 2.47E-62 | NKT | Plxdc2 |
| 2.02E-66 | -1.6426 | 0.017 | 0.435 | 3.63E-62 | NKT | Ebf1 |
| 3.14E-66 | -1.47038 | 0.008 | 0.421 | 5.65E-62 | NKT | Cavin3 |
| 7.92E-66 | -1.39796 | 0.008 | 0.418 | 1.43E-61 | NKT | Ddah2 |
| 1.16E-65 | -1.34726 | 0.019 | 0.434 | 2.08E-61 | NKT | Fxyd1 |
| 2.53E-65 | -1.24894 | 0.163 | 0.584 | 4.55E-61 | NKT | Ctsz |
| 7.12E-65 | -1.69459 | 0.008 | 0.413 | 1.28E-60 | NKT | Islr |
| 1.09E-64 | -1.64254 | 0.068 | 0.476 | 1.97E-60 | NKT | Lamc1 |
| 1.78E-64 | -1.48178 | 0.015 | 0.419 | 3.21E-60 | NKT | Nupr1 |
| 1.96E-64 | -1.41284 | 0.051 | 0.463 | 3.53E-60 | NKT | Rhoc |
| 2.06E-64 | -1.70131 | 0.061 | 0.471 | 3.71E-60 | NKT | Crip2 |
| 2.43E-64 | -1.45491 | 0.419 | 0.773 | 4.37E-60 | NKT | Sat1 |

| | | | | | | |
|---|---|---|---|---|---|---|
| 3.43E-64 | 1.106886 | 0.684 | 0.46 | 6.18E-60 | NKT | H2afy |
| 5.68E-64 | -1.25183 | 0.006 | 0.407 | 1.02E-59 | NKT | S100a16 |
| 8.46E-64 | -1.9637 | 0.023 | 0.424 | 1.52E-59 | NKT | Ckb |
| 8.62E-64 | -1.52217 | 0.008 | 0.408 | 1.55E-59 | NKT | Oaf |
| 1.07E-63 | -1.31042 | 0.004 | 0.403 | 1.93E-59 | NKT | Nfia |
| 3.18E-63 | -1.4945 | 0.008 | 0.407 | 5.72E-59 | NKT | Ramp2 |
| 3.18E-63 | -2.47962 | 0.036 | 0.432 | 5.73E-59 | NKT | Mfap5 |
| 1.03E-62 | -1.36551 | 0.006 | 0.402 | 1.86E-58 | NKT | Col5a1 |
| 1.04E-62 | -1.50577 | 0.008 | 0.405 | 1.87E-58 | NKT | Pam |
| 1.31E-62 | -1.75818 | 0.015 | 0.411 | 2.36E-58 | NKT | Ogn |
| 1.41E-62 | 1.047968 | 0.693 | 0.476 | 2.55E-58 | NKT | Psme1 |
| 2.13E-62 | -1.25382 | 0.008 | 0.405 | 3.83E-58 | NKT | Itgb5 |
| 4.95E-62 | -1.31613 | 0.006 | 0.399 | 8.92E-58 | NKT | Entpd2 |
| 1.00E-61 | -1.2363 | 0.004 | 0.395 | 1.81E-57 | NKT | Cald1 |
| 1.27E-61 | 1.023636 | 0.697 | 0.424 | 2.28E-57 | NKT | H2-Q4 |
| 3.23E-61 | -1.15905 | 0.008 | 0.401 | 5.82E-57 | NKT | Fkbp9 |
| 4.12E-61 | -1.25088 | 0.011 | 0.401 | 7.41E-57 | NKT | Pdgfra |
| 8.50E-61 | -1.35724 | 0.004 | 0.391 | 1.53E-56 | NKT | Fbln5 |
| 1.20E-60 | -1.27297 | 0.011 | 0.401 | 2.15E-56 | NKT | Fhl1 |
| 1.36E-60 | -1.20275 | 0.11 | 0.535 | 2.45E-56 | NKT | Tnfrsf1a |
| 2.61E-60 | -1.29055 | 0.04 | 0.434 | 4.70E-56 | NKT | Nfic |
| 3.33E-60 | -1.33295 | 0.011 | 0.395 | 5.99E-56 | NKT | Adamts2 |
| 4.02E-60 | -1.54965 | 0.006 | 0.39 | 7.24E-56 | NKT | Adamts5 |
| 5.04E-60 | -1.29263 | 0.006 | 0.391 | 9.07E-56 | NKT | Col5a2 |
| 6.10E-60 | -1.54165 | 0.032 | 0.424 | 1.10E-55 | NKT | Spry1 |
| 7.55E-60 | -1.14453 | 0.002 | 0.384 | 1.36E-55 | NKT | Fermt2 |
| 1.06E-59 | -1.18045 | 0.015 | 0.401 | 1.90E-55 | NKT | Pkd2 |
| 3.36E-59 | -1.18339 | 0.002 | 0.381 | 6.04E-55 | NKT | Mxra7 |
| 4.20E-59 | -1.0566 | 0 | 0.377 | 7.56E-55 | NKT | Fkbp7 |
| 5.46E-59 | -1.65829 | 0.002 | 0.379 | 9.83E-55 | NKT | Col8a1 |
| 6.49E-59 | -3.06698 | 0.042 | 0.423 | 1.17E-54 | NKT | Hspb1 |
| 6.80E-59 | -1.0548 | 0.004 | 0.382 | 1.22E-54 | NKT | Ckap4 |
| 1.42E-58 | -1.14808 | 0.996 | 0.996 | 2.56E-54 | NKT | Fth1 |
| 1.54E-58 | -1.18525 | 0 | 0.374 | 2.77E-54 | NKT | Nav1 |
| 1.57E-58 | -2.24019 | 0.019 | 0.396 | 2.83E-54 | NKT | Dpep1 |
| 2.17E-58 | -1.30294 | 0.004 | 0.379 | 3.90E-54 | NKT | Ace |
| 2.43E-58 | -1.64271 | 0.333 | 0.612 | 4.37E-54 | NKT | Anxa5 |
| 2.95E-58 | -1.35517 | 0.002 | 0.376 | 5.31E-54 | NKT | Fbn1 |
| 3.05E-58 | -1.23941 | 0.006 | 0.382 | 5.49E-54 | NKT | Emilin1 |
| 7.64E-58 | -2.27976 | 0.019 | 0.393 | 1.38E-53 | NKT | Meg3 |
| 7.75E-58 | -1.20163 | 0.328 | 0.642 | 1.39E-53 | NKT | Lamp1 |
| 1.07E-57 | -1.0709 | 0.006 | 0.378 | 1.92E-53 | NKT | Fgfr1 |
| 1.79E-57 | -1.4661 | 0.006 | 0.378 | 3.23E-53 | NKT | Col6a3 |
| 2.23E-57 | -1.67293 | 0.002 | 0.372 | 4.02E-53 | NKT | Timp3 |
| 2.53E-57 | -1.23322 | 0.002 | 0.372 | 4.55E-53 | NKT | Medag |
| 7.97E-57 | -1.29078 | 0.002 | 0.369 | 1.43E-52 | NKT | Selenbp1 |
| 1.39E-56 | 1.020365 | 0.415 | 0.172 | 2.51E-52 | NKT | Cbx4 |
| 1.59E-56 | -2.53911 | 0.03 | 0.399 | 2.85E-52 | NKT | Thbs1 |
| 2.39E-56 | -1.02934 | 0.002 | 0.367 | 4.30E-52 | NKT | Cnn3 |
| 4.50E-56 | -3.30021 | 0.057 | 0.422 | 8.10E-52 | NKT | Apoe |
| 5.52E-56 | -1.14769 | 0.004 | 0.369 | 9.94E-52 | NKT | Lama4 |
| 1.07E-55 | -1.01845 | 0.002 | 0.364 | 1.93E-51 | NKT | P3h3 |
| 1.26E-55 | -1.51899 | 0.413 | 0.73 | 2.26E-51 | NKT | Neat1 |
| 1.58E-55 | -1.23742 | 0.013 | 0.379 | 2.84E-51 | NKT | Sod3 |

| | | | | | | |
|---|---|---|---|---|---|---|
| 3.43E-55 | -1.06378 | 0.004 | 0.365 | 6.18E-51 | NKT | Cavin1 |
| 4.47E-55 | -1.47838 | 0.008 | 0.369 | 8.05E-51 | NKT | Pcolce2 |
| 1.53E-54 | -1.70966 | 0.032 | 0.396 | 2.75E-50 | NKT | Trib1 |
| 1.83E-54 | -1.03655 | 0 | 0.355 | 3.29E-50 | NKT | Mmp23 |
| 2.05E-54 | -1.07509 | 0.07 | 0.46 | 3.69E-50 | NKT | Creg1 |
| 2.27E-54 | -1.26326 | 0.004 | 0.361 | 4.08E-50 | NKT | Ppp1r14a |
| 2.59E-54 | -1.13055 | 0.002 | 0.357 | 4.67E-50 | NKT | Aebp1 |
| 6.91E-54 | -1.47192 | 0.03 | 0.39 | 1.24E-49 | NKT | Atf5 |
| 7.10E-54 | -1.07927 | 0.083 | 0.471 | 1.28E-49 | NKT | Lmo4 |
| 1.26E-53 | -3.2999 | 0.11 | 0.477 | 2.27E-49 | NKT | Msrb1 |
| 1.32E-53 | -1.6778 | 0.006 | 0.36 | 2.37E-49 | NKT | Tnfaip2 |
| 1.34E-53 | -1.4794 | 0.227 | 0.546 | 2.40E-49 | NKT | Mtch1 |
| 1.79E-53 | -1.27529 | 0.006 | 0.359 | 3.22E-49 | NKT | Serpine2 |
| 2.12E-53 | -1.32607 | 0.239 | 0.574 | 3.82E-49 | NKT | Cyb5a |
| 2.56E-53 | -1.05877 | 0 | 0.349 | 4.61E-49 | NKT | Dpysl3 |
| 5.69E-53 | -1.24228 | 0.106 | 0.471 | 1.02E-48 | NKT | Nedd4 |
| 1.76E-52 | -1.98303 | 0.025 | 0.376 | 3.17E-48 | NKT | C3 |
| 2.36E-52 | -1.02505 | 0.055 | 0.424 | 4.25E-48 | NKT | Vkorc1 |
| 2.60E-52 | -1.21371 | 0.004 | 0.35 | 4.68E-48 | NKT | Tm4sf1 |
| 3.41E-52 | -1.08546 | 0.057 | 0.418 | 6.13E-48 | NKT | Rras |
| 5.89E-52 | -1.03356 | 0.011 | 0.361 | 1.06E-47 | NKT | Il11ra1 |
| 1.96E-51 | -1.14292 | 0.011 | 0.356 | 3.53E-47 | NKT | Angptl2 |
| 8.94E-51 | -1.1414 | 0.178 | 0.568 | 1.61E-46 | NKT | Ctnnb1 |
| 1.86E-50 | -1.55266 | 0.011 | 0.348 | 3.35E-46 | NKT | Aspn |
| 1.89E-50 | -1.0712 | 0.496 | 0.763 | 3.41E-46 | NKT | Anxa2 |
| 2.30E-50 | -1.0769 | 0 | 0.334 | 4.14E-46 | NKT | Col4a2 |
| 2.97E-50 | -1.55708 | 0.006 | 0.342 | 5.35E-46 | NKT | Spon2 |
| 3.67E-50 | -1.01946 | 0.008 | 0.346 | 6.60E-46 | NKT | Vwa1 |
| 3.75E-50 | -1.19056 | 0.006 | 0.343 | 6.76E-46 | NKT | Htra1 |
| 8.67E-50 | -1.35329 | 0.004 | 0.338 | 1.56E-45 | NKT | Gfpt2 |
| 9.18E-50 | -1.29292 | 0.331 | 0.695 | 1.65E-45 | NKT | Gpx1 |
| 3.30E-49 | -1.16639 | 0.004 | 0.335 | 5.94E-45 | NKT | Pla1a |
| 3.55E-49 | -1.45952 | 0.093 | 0.451 | 6.40E-45 | NKT | Ier3 |
| 7.73E-49 | -1.29383 | 0.004 | 0.333 | 1.39E-44 | NKT | Eln |
| 1.39E-48 | -1.00424 | 0.076 | 0.432 | 2.50E-44 | NKT | Calu |
| 2.90E-48 | -1.01317 | 0.214 | 0.601 | 5.22E-44 | NKT | Ppp1r2 |
| 3.87E-48 | -1.99505 | 0.216 | 0.526 | 6.97E-44 | NKT | Socs3 |
| 6.09E-48 | 1.115502 | 0.655 | 0.465 | 1.10E-43 | NKT | Slc3a2 |
| 6.48E-48 | -1.06876 | 0.04 | 0.381 | 1.17E-43 | NKT | Socs2 |
| 7.89E-48 | -2.64329 | 0.019 | 0.345 | 1.42E-43 | NKT | Cxcl1 |
| 8.38E-48 | -1.26144 | 0 | 0.321 | 1.51E-43 | NKT | Gsta3 |
| 1.25E-47 | -1.41929 | 0.006 | 0.329 | 2.25E-43 | NKT | Nbl1 |
| 2.96E-47 | 1.250841 | 0.644 | 0.454 | 5.33E-43 | NKT | Ubald2 |
| 3.08E-47 | -1.04843 | 0.159 | 0.506 | 5.55E-43 | NKT | Tmed3 |
| 3.99E-47 | -1.20625 | 0.008 | 0.331 | 7.18E-43 | NKT | Gda |
| 1.00E-46 | -2.90865 | 0.028 | 0.345 | 1.81E-42 | NKT | Fcer1g |
| 2.01E-46 | -3.8916 | 0.049 | 0.363 | 3.62E-42 | NKT | Tyrobp |
| 3.69E-46 | -1.08745 | 0.002 | 0.317 | 6.65E-42 | NKT | Sfrp1 |
| 6.67E-46 | -1.21992 | 0.121 | 0.459 | 1.20E-41 | NKT | Emp1 |
| 1.05E-45 | -1.11453 | 0.006 | 0.32 | 1.89E-41 | NKT | Col5a3 |
| 6.58E-45 | -1.05051 | 0.004 | 0.313 | 1.18E-40 | NKT | Fxyd6 |
| 1.30E-44 | -2.12157 | 0.013 | 0.321 | 2.35E-40 | NKT | Cyr61 |
| 3.37E-44 | -1.33654 | 0.114 | 0.447 | 6.06E-40 | NKT | Errfi1 |
| 5.38E-44 | -1.40387 | 0.011 | 0.318 | 9.69E-40 | NKT | Emilin2 |

| | | | | | | |
|---|---|---|---|---|---|---|
| 2.37E-43 | -1.07749 | 0.002 | 0.301 | 4.26E-39 | NKT | Fibin |
| 1.22E-42 | -1.49044 | 0.292 | 0.651 | 2.21E-38 | NKT | Litaf |
| 2.74E-42 | -1.19733 | 0.04 | 0.355 | 4.94E-38 | NKT | Jdp2 |
| 5.91E-42 | -1.08681 | 0.004 | 0.297 | 1.06E-37 | NKT | Adamts1 |
| 6.80E-42 | -2.17666 | 0.021 | 0.318 | 1.22E-37 | NKT | Plaur |
| 8.11E-42 | 1.044775 | 0.331 | 0.137 | 1.46E-37 | NKT | Serpinb9 |
| 1.21E-41 | -1.3877 | 0.451 | 0.643 | 2.18E-37 | NKT | Tuba1a |
| 1.22E-41 | -1.01758 | 0.144 | 0.498 | 2.20E-37 | NKT | Klf3 |
| 1.75E-41 | -1.01121 | 0.004 | 0.294 | 3.15E-37 | NKT | Gas6 |
| 3.55E-41 | -1.20252 | 0.142 | 0.465 | 6.40E-37 | NKT | Phlda1 |
| 4.90E-41 | -1.06633 | 0.04 | 0.347 | 8.83E-37 | NKT | Plin2 |
| 8.37E-41 | -1.11406 | 0.025 | 0.322 | 1.51E-36 | NKT | Bag3 |
| 2.07E-40 | -2.61759 | 0.011 | 0.294 | 3.72E-36 | NKT | Alox5ap |
| 7.24E-40 | -1.69602 | 0.017 | 0.303 | 1.30E-35 | NKT | Mt2 |
| 7.59E-40 | -1.05031 | 0.097 | 0.409 | 1.37E-35 | NKT | Itm2a |
| 2.37E-39 | -1.04495 | 0 | 0.276 | 4.27E-35 | NKT | Sirpa |
| 1.65E-38 | -1.13795 | 0.011 | 0.286 | 2.97E-34 | NKT | Tppp3 |
| 1.71E-38 | -1.0355 | 0.004 | 0.277 | 3.08E-34 | NKT | Fmo2 |
| 2.02E-38 | -1.08142 | 0.159 | 0.488 | 3.63E-34 | NKT | Ndel1 |
| 2.80E-38 | -1.29386 | 0.002 | 0.273 | 5.05E-34 | NKT | Ackr3 |
| 4.02E-38 | -1.1207 | 0.057 | 0.35 | 7.24E-34 | NKT | Gadd45a |
| 4.47E-38 | -1.7379 | 0.015 | 0.29 | 8.05E-34 | NKT | Tgfbi |
| 1.84E-37 | -1.69761 | 0.25 | 0.571 | 3.32E-33 | NKT | Grina |
| 2.17E-37 | -2.41304 | 0.028 | 0.298 | 3.91E-33 | NKT | Lst1 |
| 1.79E-36 | -1.22728 | 0.008 | 0.273 | 3.22E-32 | NKT | Tgm2 |
| 3.53E-36 | -1.15817 | 0.07 | 0.36 | 6.36E-32 | NKT | Snap23 |
| 9.58E-36 | -1.17427 | 0.146 | 0.462 | 1.72E-31 | NKT | Nfkbiz |
| 1.09E-35 | -1.56388 | 0.013 | 0.272 | 1.96E-31 | NKT | Tpd52 |
| 1.09E-35 | -2.35567 | 0.036 | 0.302 | 1.97E-31 | NKT | Slc16a3 |
| 1.90E-35 | -1.57644 | 0.015 | 0.275 | 3.42E-31 | NKT | Ctgf |
| 2.31E-35 | -2.72613 | 0.004 | 0.258 | 4.16E-31 | NKT | Hp |
| 2.74E-35 | -2.075 | 0.013 | 0.268 | 4.93E-31 | NKT | Ncf2 |
| 7.55E-35 | -1.56269 | 0.017 | 0.272 | 1.36E-30 | NKT | Sell |
| 1.40E-34 | -1.22041 | 0.049 | 0.322 | 2.51E-30 | NKT | Tmcc1 |
| 3.96E-34 | -4.39802 | 0.059 | 0.315 | 7.12E-30 | NKT | Lyz2 |
| 5.75E-34 | -1.71472 | 0.066 | 0.33 | 1.03E-29 | NKT | Ets2 |
| 2.12E-33 | -1.82945 | 0.008 | 0.252 | 3.82E-29 | NKT | Spi1 |
| 3.86E-33 | -1.21823 | 0.078 | 0.351 | 6.94E-29 | NKT | Gadd45g |
| 7.92E-33 | -1.09504 | 0.015 | 0.262 | 1.43E-28 | NKT | Hmox1 |
| 1.88E-32 | -2.87778 | 0.034 | 0.277 | 3.38E-28 | NKT | Ccl6 |
| 2.98E-32 | -1.11454 | 0.038 | 0.293 | 5.36E-28 | NKT | Kctd12 |
| 4.81E-32 | -2.8753 | 0.025 | 0.268 | 8.65E-28 | NKT | Ccrl2 |
| 2.01E-31 | -3.04955 | 0.021 | 0.256 | 3.62E-27 | NKT | Il1r2 |
| 2.37E-31 | -5.44644 | 0.087 | 0.333 | 4.26E-27 | NKT | Retnlg |
| 6.93E-31 | -6.44802 | 0.22 | 0.473 | 1.25E-26 | NKT | S100a8 |
| 1.97E-30 | -2.31849 | 0.023 | 0.253 | 3.54E-26 | NKT | Csf3r |
| 2.70E-30 | -3.34447 | 0.036 | 0.267 | 4.86E-26 | NKT | Il1b |
| 4.08E-30 | -1.06906 | 0.11 | 0.375 | 7.35E-26 | NKT | Uap1 |
| 2.31E-29 | 1.145445 | 0.706 | 0.565 | 4.15E-25 | NKT | Rpl13a |
| 2.14E-25 | -6.49038 | 0.278 | 0.508 | 3.85E-21 | NKT | S100a9 |
| 2.72E-24 | -1.14639 | 0.083 | 0.302 | 4.89E-20 | NKT | Ninj1 |
| 4.84E-23 | -1.01897 | 0.136 | 0.363 | 8.71E-19 | NKT | Nudt4 |
| 2.55E-22 | -1.04798 | 0.341 | 0.53 | 4.58E-18 | NKT | Fosb |
| 9.37E-22 | -1.03774 | 0.117 | 0.333 | 1.69E-17 | NKT | Ugdh |

| | | | | | | |
|---|---|---|---|---|---|---|
| 1.93E-19 | -1.38413 | 0.146 | 0.344 | 3.47E-15 | NKT | Rnf149 |
| 2.92E-19 | -1.02206 | 0.411 | 0.593 | 5.25E-15 | NKT | Hspa1b |
| 1.26E-17 | -3.60476 | 0.263 | 0.506 | 2.27E-13 | NKT | Hba-a1 |
| 6.86E-13 | -3.48452 | 0.468 | 0.674 | 1.23E-08 | NKT | Hbb-bs |
| 1.11E-12 | -1.23723 | 0.466 | 0.654 | 2.01E-08 | NKT | Taldo1 |
| 1.16E-08 | -1.52715 | 0.195 | 0.285 | 0.000209 | NKT | Lmnb1 |
| 1.53E-06 | -1.25374 | 0.761 | 0.798 | 0.0275 | NKT | S100a11 |
| 0 | 4.683094 | 0.425 | 0.005 | 0 | Stromal c | Rgs5 |
| 0 | 3.809545 | 0.345 | 0 | 0 | Stromal c | Myh11 |
| 0 | 2.92914 | 0.437 | 0.005 | 0 | Stromal c | Egfl7 |
| 0 | 2.83782 | 0.471 | 0.008 | 0 | Stromal c | Mylk |
| 0 | 2.828387 | 0.724 | 0.024 | 0 | Stromal c | Tinagl1 |
| 0 | 2.687808 | 0.632 | 0.021 | 0 | Stromal c | Bcam |
| 0 | 2.535743 | 0.379 | 0.002 | 0 | Stromal c | Higd1b |
| 0 | 2.519826 | 0.253 | 0 | 0 | Stromal c | Cldn5 |
| 0 | 2.315916 | 0.379 | 0.001 | 0 | Stromal c | Sncg |
| 0 | 2.272503 | 0.425 | 0.004 | 0 | Stromal c | Tcf15 |
| 0 | 2.260343 | 0.414 | 0.002 | 0 | Stromal c | Rgs4 |
| 0 | 2.116942 | 0.299 | 0 | 0 | Stromal c | Emcn |
| 0 | 2.098189 | 0.563 | 0.001 | 0 | Stromal c | Esam |
| 0 | 2.071782 | 0.31 | 0.001 | 0 | Stromal c | Sorbs2 |
| 0 | 1.850429 | 0.368 | 0.003 | 0 | Stromal c | Pcp4l1 |
| 0 | 1.805114 | 0.31 | 0 | 0 | Stromal c | Cdh5 |
| 0 | 1.795516 | 0.402 | 0.006 | 0 | Stromal c | Mcam |
| 0 | 1.786183 | 0.322 | 0.002 | 0 | Stromal c | Lmod1 |
| 0 | 1.601677 | 0.276 | 0.002 | 0 | Stromal c | Apold1 |
| 0 | 1.594506 | 0.322 | 0.004 | 0 | Stromal c | Cspg4 |
| 0 | 1.463109 | 0.253 | 0.001 | 0 | Stromal c | Tie1 |
| 0 | 1.37078 | 0.333 | 0 | 0 | Stromal c | Casq2 |
| 0 | 1.250929 | 0.264 | 0.003 | 0 | Stromal c | F11r |
| 0 | 1.208392 | 0.264 | 0.002 | 0 | Stromal c | Map3k7cl |
| 0 | 1.194773 | 0.253 | 0.001 | 0 | Stromal c | Nrip2 |
| 0 | 1.139661 | 0.253 | 0 | 0 | Stromal c | Olfr558 |
| ######## | 1.158407 | 0.31 | 0.005 | ######## | Stromal c | Ccdc3 |
| ######## | 1.359352 | 0.253 | 0.003 | ######## | Stromal c | Pln |
| ######## | 1.471868 | 0.253 | 0.003 | ######## | Stromal c | Kdr |
| ######## | 1.187803 | 0.345 | 0.007 | ######## | Stromal c | Perp |
| ######## | 1.764818 | 0.425 | 0.011 | ######## | Stromal c | Aoc3 |
| ######## | 2.989101 | 0.69 | 0.031 | ######## | Stromal c | Cav1 |
| ######## | 5.234221 | 0.517 | 0.022 | ######## | Stromal c | Fabp4 |
| ######## | 2.713321 | 0.253 | 0.005 | ######## | Stromal c | Cd36 |
| ######## | 2.32943 | 0.483 | 0.02 | ######## | Stromal c | Gja4 |
| ######## | 1.835275 | 0.448 | 0.018 | ######## | Stromal c | Cav2 |
| ######## | 1.532766 | 0.253 | 0.006 | ######## | Stromal c | Ecscr |
| ######## | 1.559289 | 0.391 | 0.016 | ######## | Stromal c | Atp1b2 |
| ######## | 2.155263 | 0.276 | 0.009 | ######## | Stromal c | Aqp1 |
| ######## | 1.222704 | 0.287 | 0.01 | ######## | Stromal c | Rcan2 |
| ######## | 1.510057 | 0.299 | 0.012 | ######## | Stromal c | Clu |
| ######## | 3.117384 | 0.483 | 0.033 | ######## | Stromal c | Cox4i2 |
| ######## | 1.011597 | 0.299 | 0.014 | ######## | Stromal c | Pcdh1 |
| ######## | 1.248419 | 0.322 | 0.016 | ######## | Stromal c | Itga7 |
| ######## | 4.820997 | 0.54 | 0.051 | 3.41E-97 | Stromal c | Myl9 |
| 7.76E-97 | 1.794536 | 0.31 | 0.016 | 1.40E-92 | Stromal c | Rasd1 |
| 9.36E-95 | 1.027802 | 0.253 | 0.011 | 1.68E-90 | Stromal c | Mrvi1 |

| | | | | | | |
|---|---|---|---|---|---|---|
| 2.52E-92 | 2.837747 | 0.437 | 0.036 | 4.53E-88 | Stromal c | Mustn1 |
| 1.23E-91 | 2.432462 | 0.517 | 0.051 | 2.21E-87 | Stromal c | Notch3 |
| 4.92E-81 | 1.196503 | 0.31 | 0.02 | 8.85E-77 | Stromal c | Rbpms2 |
| 5.65E-81 | 5.630692 | 0.402 | 0.034 | 1.02E-76 | Stromal c | Tagln |
| 4.21E-78 | 2.347195 | 0.402 | 0.035 | 7.59E-74 | Stromal c | Des |
| 3.81E-65 | 3.538175 | 0.552 | 0.082 | 6.87E-61 | Stromal c | Gm13889 |
| 7.59E-65 | 1.718628 | 0.379 | 0.037 | 1.37E-60 | Stromal c | Tcim |
| 1.38E-60 | 1.204668 | 0.31 | 0.027 | 2.48E-56 | Stromal c | Kitl |
| 1.12E-59 | 1.836886 | 0.402 | 0.044 | 2.02E-55 | Stromal c | Cpe |
| 5.78E-57 | 1.541743 | 0.264 | 0.021 | 1.04E-52 | Stromal c | Ptprb |
| 1.61E-55 | 2.039094 | 0.506 | 0.078 | 2.90E-51 | Stromal c | Gucy1a1 |
| 4.25E-53 | 1.647557 | 0.264 | 0.022 | 7.65E-49 | Stromal c | Art3 |
| 3.33E-51 | 2.541856 | 0.471 | 0.07 | 6.00E-47 | Stromal c | Cd200 |
| 3.99E-51 | 1.511528 | 0.414 | 0.055 | 7.18E-47 | Stromal c | Gjc1 |
| 1.30E-50 | 2.93148 | 0.862 | 0.325 | 2.35E-46 | Stromal c | Gng11 |
| 4.63E-50 | 1.118401 | 0.414 | 0.052 | 8.34E-46 | Stromal c | Plekho1 |
| 1.54E-49 | 2.232872 | 0.253 | 0.022 | 2.77E-45 | Stromal c | Abcc9 |
| 8.26E-46 | 2.669974 | 0.483 | 0.086 | 1.49E-41 | Stromal c | Cavin2 |
| 8.68E-46 | 1.502739 | 0.356 | 0.046 | 1.56E-41 | Stromal c | Palld |
| 6.32E-44 | 2.419732 | 0.517 | 0.096 | 1.14E-39 | Stromal c | Igfbp5 |
| 1.32E-43 | 1.928254 | 0.494 | 0.093 | 2.38E-39 | Stromal c | Gucy1b1 |
| 3.73E-37 | 1.086768 | 0.276 | 0.033 | 6.71E-33 | Stromal c | Rapgef5 |
| 1.05E-36 | 4.180363 | 0.575 | 0.159 | 1.90E-32 | Stromal c | Tpm2 |
| 1.54E-36 | 2.299043 | 0.552 | 0.142 | 2.78E-32 | Stromal c | Epas1 |
| 2.12E-35 | 1.676133 | 0.402 | 0.071 | 3.81E-31 | Stromal c | Pdlim3 |
| 2.42E-35 | 1.348697 | 0.402 | 0.072 | 4.35E-31 | Stromal c | Crim1 |
| 4.05E-35 | 1.143838 | 0.287 | 0.037 | 7.30E-31 | Stromal c | Tesc |
| 5.53E-35 | 2.429714 | 0.632 | 0.209 | 9.95E-31 | Stromal c | Tsc22d1 |
| 8.90E-34 | 1.911282 | 0.356 | 0.06 | 1.60E-29 | Stromal c | Tspan7 |
| 3.29E-32 | 1.080229 | 0.345 | 0.058 | 5.92E-28 | Stromal c | Sorbs1 |
| 8.19E-32 | 2.696176 | 0.77 | 0.34 | 1.48E-27 | Stromal c | Tm4sf1 |
| 3.71E-31 | 3.306421 | 0.253 | 0.033 | 6.68E-27 | Stromal c | Kcnj8 |
| 1.79E-30 | 1.969759 | 0.644 | 0.235 | 3.23E-26 | Stromal c | Tns1 |
| 3.32E-30 | 2.024527 | 0.368 | 0.068 | 5.98E-26 | Stromal c | Rasl11a |
| 1.48E-29 | 1.071626 | 0.264 | 0.038 | 2.66E-25 | Stromal c | Tbx2 |
| 5.54E-28 | 1.378234 | 0.379 | 0.08 | 9.98E-24 | Stromal c | Nr2f2 |
| 8.64E-28 | 1.992741 | 0.793 | 0.362 | 1.56E-23 | Stromal c | Timp3 |
| 4.91E-27 | 1.487446 | 0.356 | 0.071 | 8.83E-23 | Stromal c | Myo1b |
| 1.31E-26 | 1.284824 | 0.276 | 0.044 | 2.36E-22 | Stromal c | Hrct1 |
| 1.86E-26 | 1.900462 | 0.69 | 0.305 | 3.34E-22 | Stromal c | Rbpms |
| 3.11E-26 | -2.32192 | 0.08 | 0.713 | 5.60E-22 | Stromal c | Lsp1 |
| 1.69E-25 | 1.078089 | 0.322 | 0.059 | 3.04E-21 | Stromal c | Trp53i11 |
| 3.29E-24 | 2.394323 | 0.575 | 0.212 | 5.91E-20 | Stromal c | Ptp4a3 |
| 4.37E-24 | 1.171574 | 0.287 | 0.051 | 7.86E-20 | Stromal c | Chchd10 |
| 1.41E-23 | 1.11297 | 0.402 | 0.099 | 2.54E-19 | Stromal c | Ehd4 |
| 2.19E-23 | 1.064031 | 0.31 | 0.063 | 3.94E-19 | Stromal c | Col18a1 |
| 8.90E-23 | 1.581763 | 0.46 | 0.137 | 1.60E-18 | Stromal c | Cystm1 |
| 1.22E-22 | 1.866184 | 0.299 | 0.061 | 2.20E-18 | Stromal c | Pecam1 |
| 1.71E-22 | 1.268743 | 0.299 | 0.06 | 3.08E-18 | Stromal c | Btbd3 |
| 2.74E-22 | 1.623505 | 0.667 | 0.294 | 4.94E-18 | Stromal c | Cd151 |
| 4.34E-22 | 1.582927 | 0.644 | 0.297 | 7.81E-18 | Stromal c | Actn4 |
| 1.02E-21 | 1.072419 | 0.299 | 0.062 | 1.83E-17 | Stromal c | Ppp1r12b |
| 1.55E-21 | 2.315711 | 0.529 | 0.196 | 2.78E-17 | Stromal c | Csrp2 |
| 1.80E-20 | -1.88415 | 0.598 | 0.865 | 3.25E-16 | Stromal c | S100a6 |

| | | | | | | |
|---|---|---|---|---|---|---|
| 2.46E-20 | 1.594641 | 0.414 | 0.122 | 4.43E-16 | Stromal c | Itga1 |
| 2.78E-20 | 1.504106 | 0.782 | 0.46 | 5.00E-16 | Stromal c | Crip2 |
| 3.02E-20 | 2.053473 | 0.402 | 0.114 | 5.44E-16 | Stromal c | Mgll |
| 3.92E-20 | -1.4923 | 0.08 | 0.631 | 7.06E-16 | Stromal c | Celf2 |
| 4.54E-20 | -1.54291 | 0.92 | 0.989 | 8.18E-16 | Stromal c | Ftl1 |
| 9.53E-20 | 2.210231 | 0.667 | 0.385 | 1.72E-15 | Stromal c | Cald1 |
| 1.09E-19 | 1.511521 | 0.471 | 0.156 | 1.96E-15 | Stromal c | Ldhb |
| 1.31E-19 | 5.055492 | 0.402 | 0.122 | 2.35E-15 | Stromal c | Acta2 |
| 1.63E-19 | 1.445154 | 0.713 | 0.409 | 2.93E-15 | Stromal c | Rras |
| 1.69E-19 | 1.158036 | 0.264 | 0.053 | 3.04E-15 | Stromal c | Tspan12 |
| 4.25E-19 | 1.404828 | 0.793 | 0.51 | 7.65E-15 | Stromal c | Uqcr11 |
| 7.36E-19 | 1.096687 | 0.448 | 0.144 | 1.33E-14 | Stromal c | Dab2ip |
| 7.82E-19 | 1.213397 | 0.862 | 0.657 | 1.41E-14 | Stromal c | Cox7a2 |
| 8.15E-19 | 1.683794 | 0.92 | 0.913 | 1.47E-14 | Stromal c | Myl6 |
| 1.38E-18 | 1.026888 | 0.299 | 0.068 | 2.49E-14 | Stromal c | Plxnd1 |
| 2.17E-18 | 1.074238 | 0.494 | 0.168 | 3.91E-14 | Stromal c | Mef2c |
| 2.25E-18 | 1.507991 | 0.655 | 0.356 | 4.05E-14 | Stromal c | Cavin1 |
| 4.28E-18 | 1.815336 | 0.667 | 0.37 | 7.70E-14 | Stromal c | Mfge8 |
| 5.98E-18 | 1.034267 | 0.287 | 0.067 | 1.08E-13 | Stromal c | Plce1 |
| 6.40E-18 | 2.748006 | 0.575 | 0.295 | 1.15E-13 | Stromal c | Tpm1 |
| 7.51E-18 | 1.508867 | 0.54 | 0.23 | 1.35E-13 | Stromal c | Pkig |
| 3.56E-17 | 1.160279 | 0.828 | 0.649 | 6.41E-13 | Stromal c | Atp5j |
| 5.63E-17 | 1.039147 | 0.299 | 0.076 | 1.01E-12 | Stromal c | Map3k20 |
| 7.34E-17 | -2.22189 | 0.092 | 0.55 | 1.32E-12 | Stromal c | Cotl1 |
| 9.25E-17 | -1.96868 | 0.299 | 0.772 | 1.67E-12 | Stromal c | Fxyd5 |
| 2.54E-16 | 1.226942 | 0.966 | 0.793 | 4.57E-12 | Stromal c | mt-Nd1 |
| 2.73E-16 | 1.953408 | 0.425 | 0.149 | 4.92E-12 | Stromal c | Fabp5 |
| 3.52E-16 | 1.28648 | 0.759 | 0.507 | 6.34E-12 | Stromal c | Itgb1 |
| 3.69E-16 | 1.337098 | 0.793 | 0.469 | 6.65E-12 | Stromal c | Id3 |
| 4.00E-16 | 1.504628 | 0.506 | 0.207 | 7.20E-12 | Stromal c | Hes1 |
| 9.59E-16 | -3.43582 | 0.046 | 0.494 | 1.73E-11 | Stromal c | Cd52 |
| 1.02E-15 | 1.21769 | 0.517 | 0.219 | 1.84E-11 | Stromal c | Oaz2 |
| 1.36E-15 | 1.265467 | 0.529 | 0.235 | 2.46E-11 | Stromal c | Ctnna1 |
| 1.71E-15 | 1.756203 | 0.483 | 0.193 | 3.08E-11 | Stromal c | Id1 |
| 1.80E-15 | 2.565531 | 0.299 | 0.078 | 3.24E-11 | Stromal c | Rrad |
| 2.07E-15 | -3.71164 | 0.299 | 0.643 | 3.72E-11 | Stromal c | Dcn |
| 2.22E-15 | 2.829229 | 0.506 | 0.227 | 4.00E-11 | Stromal c | Ndufa4l2 |
| 2.62E-15 | 2.379241 | 0.506 | 0.231 | 4.72E-11 | Stromal c | Csrp1 |
| 4.23E-15 | 1.196324 | 0.575 | 0.273 | 7.62E-11 | Stromal c | Ppp1r12c |
| 4.52E-15 | -2.87956 | 0.103 | 0.522 | 8.14E-11 | Stromal c | Lum |
| 5.12E-15 | -2.82613 | 0.034 | 0.465 | 9.22E-11 | Stromal c | Rac2 |
| 8.95E-15 | 1.4297 | 0.414 | 0.156 | 1.61E-10 | Stromal c | Smtn |
| 9.24E-15 | 1.27018 | 0.77 | 0.571 | 1.66E-10 | Stromal c | Slc25a4 |
| 1.04E-14 | 1.036447 | 0.414 | 0.152 | 1.88E-10 | Stromal c | Vcl |
| 1.65E-14 | -3.18255 | 0.149 | 0.535 | 2.98E-10 | Stromal c | Htra3 |
| 2.12E-14 | 1.349677 | 0.563 | 0.304 | 3.81E-10 | Stromal c | Ehd2 |
| 2.54E-14 | -2.59534 | 0.046 | 0.466 | 4.58E-10 | Stromal c | Coro1a |
| 4.15E-14 | -2.29377 | 0.092 | 0.485 | 7.48E-10 | Stromal c | Mmp2 |
| 6.54E-14 | -1.17451 | 0.483 | 0.827 | 1.18E-09 | Stromal c | Tsc22d3 |
| 6.62E-14 | 1.621817 | 0.874 | 0.673 | 1.19E-09 | Stromal c | Crip1 |
| 7.07E-14 | 1.427441 | 0.437 | 0.177 | 1.27E-09 | Stromal c | Filip1l |
| 8.60E-14 | -2.68084 | 0.08 | 0.485 | 1.55E-09 | Stromal c | Fbln1 |
| 1.05E-13 | 1.06974 | 0.862 | 0.688 | 1.89E-09 | Stromal c | Selenow |
| 1.10E-13 | -1.55551 | 0.115 | 0.544 | 1.97E-09 | Stromal c | Mgst1 |

| | | | | | | |
|---|---|---|---|---|---|---|
| 1.14E-13 | 1.352816 | 0.471 | 0.202 | 2.05E-09 | Stromal c | Dlc1 |
| 1.23E-13 | 1.559448 | 0.448 | 0.195 | 2.21E-09 | Stromal c | Map1b |
| 1.27E-13 | -1.80971 | 0.057 | 0.475 | 2.29E-09 | Stromal c | Dpt |
| 3.10E-13 | -1.58153 | 0.023 | 0.424 | 5.57E-09 | Stromal c | Olfml3 |
| 3.33E-13 | 1.31531 | 0.31 | 0.099 | 5.99E-09 | Stromal c | Arhgap31 |
| 4.14E-13 | 1.44124 | 0.678 | 0.457 | 7.45E-09 | Stromal c | Atpif1 |
| 5.72E-13 | -2.25806 | 0.069 | 0.462 | 1.03E-08 | Stromal c | Smoc2 |
| 5.81E-13 | 1.700789 | 0.678 | 0.438 | 1.05E-08 | Stromal c | Errfi1 |
| 8.61E-13 | -1.95984 | 0.011 | 0.41 | 1.55E-08 | Stromal c | Laptm5 |
| 9.68E-13 | -2.23186 | 0.011 | 0.4 | 1.74E-08 | Stromal c | Selplg |
| 1.48E-12 | 1.514255 | 0.575 | 0.301 | 2.67E-08 | Stromal c | Fam162a |
| 1.58E-12 | -1.12901 | 0.184 | 0.579 | 2.84E-08 | Stromal c | Serp1 |
| 1.67E-12 | -2.2525 | 0.034 | 0.414 | 3.01E-08 | Stromal c | Ptprc |
| 1.67E-12 | 1.017265 | 0.954 | 0.843 | 3.01E-08 | Stromal c | mt-Cytb |
| 1.91E-12 | -2.13575 | 0 | 0.381 | 3.44E-08 | Stromal c | Cytip |
| 1.97E-12 | 1.749705 | 0.379 | 0.147 | 3.54E-08 | Stromal c | 4-Sep |
| 2.13E-12 | -1.98073 | 0.103 | 0.474 | 3.84E-08 | Stromal c | Serpinf1 |
| 3.88E-12 | -2.55391 | 0.172 | 0.515 | 6.99E-08 | Stromal c | Clec3b |
| 4.07E-12 | 1.353342 | 0.483 | 0.234 | 7.32E-08 | Stromal c | Pim3 |
| 4.93E-12 | -1.82074 | 0.011 | 0.391 | 8.88E-08 | Stromal c | Cd53 |
| 5.04E-12 | -2.04327 | 0.011 | 0.383 | 9.07E-08 | Stromal c | Gmfg |
| 5.29E-12 | 1.020176 | 0.586 | 0.325 | 9.53E-08 | Stromal c | Mprip |
| 5.90E-12 | 1.090567 | 0.943 | 0.827 | 1.06E-07 | Stromal c | mt-Nd4 |
| 7.64E-12 | -1.63559 | 0.092 | 0.449 | 1.38E-07 | Stromal c | Loxl1 |
| 8.56E-12 | 1.332604 | 0.471 | 0.233 | 1.54E-07 | Stromal c | Pls3 |
| 1.02E-11 | -2.1843 | 0.103 | 0.459 | 1.84E-07 | Stromal c | Stk17b |
| 1.19E-11 | -2.59389 | 0.034 | 0.392 | 2.14E-07 | Stromal c | Lcp1 |
| 1.21E-11 | 1.117948 | 0.598 | 0.374 | 2.18E-07 | Stromal c | Fermt2 |
| 1.30E-11 | -1.17108 | 0.023 | 0.394 | 2.34E-07 | Stromal c | Pdgfra |
| 1.33E-11 | 1.017486 | 0.782 | 0.58 | 2.40E-07 | Stromal c | Fkbp1a |
| 1.40E-11 | 1.675353 | 0.368 | 0.148 | 2.53E-07 | Stromal c | Lims2 |
| 2.18E-11 | -1.08899 | 0.287 | 0.648 | 3.92E-07 | Stromal c | Ifngr1 |
| 2.62E-11 | -2.01915 | 0.149 | 0.505 | 4.72E-07 | Stromal c | Gpx3 |
| 2.70E-11 | -1.22519 | 0.034 | 0.391 | 4.85E-07 | Stromal c | Entpd2 |
| 4.62E-11 | -1.32975 | 0.08 | 0.435 | 8.31E-07 | Stromal c | Gpm6b |
| 4.96E-11 | -1.78314 | 0.241 | 0.584 | 8.92E-07 | Stromal c | Pi16 |
| 6.20E-11 | -2.09654 | 0.161 | 0.49 | 1.12E-06 | Stromal c | Col1a1 |
| 6.43E-11 | -1.94816 | 0.034 | 0.389 | 1.16E-06 | Stromal c | Dpep1 |
| 9.17E-11 | 1.929728 | 0.517 | 0.29 | 1.65E-06 | Stromal c | Adamts1 |
| 1.20E-10 | 1.077714 | 0.586 | 0.339 | 2.16E-06 | Stromal c | Lims1 |
| 1.23E-10 | -1.24147 | 0.057 | 0.394 | 2.21E-06 | Stromal c | Col5a1 |
| 1.37E-10 | 1.020954 | 0.264 | 0.084 | 2.47E-06 | Stromal c | Tjp1 |
| 1.98E-10 | 1.024678 | 0.299 | 0.107 | 3.56E-06 | Stromal c | Pde3a |
| 2.68E-10 | 1.162401 | 0.471 | 0.235 | 4.83E-06 | Stromal c | Actn1 |
| 2.70E-10 | 1.004074 | 0.724 | 0.542 | 4.86E-06 | Stromal c | Uqcrb |
| 2.75E-10 | 1.071632 | 0.299 | 0.099 | 4.95E-06 | Stromal c | Postn |
| 2.89E-10 | 1.092123 | 0.586 | 0.369 | 5.21E-06 | Stromal c | Hcfc1r1 |
| 3.01E-10 | 1.489693 | 0.575 | 0.369 | 5.42E-06 | Stromal c | Ppp1r12a |
| 3.07E-10 | 1.227269 | 0.517 | 0.298 | 5.53E-06 | Stromal c | Mast4 |
| 3.30E-10 | -1.23689 | 0.023 | 0.364 | 5.95E-06 | Stromal c | Hsd11b1 |
| 3.95E-10 | -1.17449 | 0.08 | 0.417 | 7.12E-06 | Stromal c | Bicc1 |
| 4.09E-10 | -3.97716 | 0.69 | 0.771 | 7.36E-06 | Stromal c | Gsn |
| 5.02E-10 | -1.30419 | 0.138 | 0.477 | 9.04E-06 | Stromal c | Gpsm3 |
| 5.91E-10 | -1.79312 | 0.241 | 0.56 | 1.06E-05 | Stromal c | Pim1 |

| | | | | | | |
|---|---|---|---|---|---|---|
| 6.07E-10 | 1.071829 | 0.264 | 0.087 | 1.09E-05 | Stromal c | Bcr |
| 6.20E-10 | -1.55667 | 0.115 | 0.432 | 1.12E-05 | Stromal c | Pcsk6 |
| 6.66E-10 | -1.223 | 0.08 | 0.42 | 1.20E-05 | Stromal c | Ezr |
| 6.71E-10 | 1.826533 | 0.437 | 0.22 | 1.21E-05 | Stromal c | Pdgfrb |
| 6.76E-10 | 1.085442 | 0.552 | 0.324 | 1.22E-05 | Stromal c | Macf1 |
| 7.00E-10 | -1.11732 | 0.908 | 0.976 | 1.26E-05 | Stromal c | Actg1 |
| 7.57E-10 | -1.70201 | 0 | 0.314 | 1.36E-05 | Stromal c | Hcst |
| 7.62E-10 | 1.166522 | 0.437 | 0.215 | 1.37E-05 | Stromal c | 2010111I01Rik |
| 8.51E-10 | -3.064 | 0.299 | 0.559 | 1.53E-05 | Stromal c | Srgn |
| 8.63E-10 | -1.02609 | 0.034 | 0.365 | 1.55E-05 | Stromal c | Medag |
| 9.42E-10 | -1.32981 | 0.092 | 0.416 | 1.70E-05 | Stromal c | Tcf21 |
| 1.08E-09 | -3.68989 | 0.046 | 0.357 | 1.94E-05 | Stromal c | Tyrobp |
| 1.24E-09 | -1.50406 | 0.471 | 0.774 | 2.23E-05 | Stromal c | Mcl1 |
| 1.27E-09 | -1.82203 | 0.161 | 0.456 | 2.29E-05 | Stromal c | Col15a1 |
| 1.93E-09 | 1.32648 | 0.437 | 0.217 | 3.48E-05 | Stromal c | Prss23 |
| 2.15E-09 | -1.02218 | 0.839 | 0.802 | 3.87E-05 | Stromal c | Rpsa |
| 2.87E-09 | -1.10705 | 0.08 | 0.401 | 5.17E-05 | Stromal c | Oaf |
| 2.91E-09 | -1.43161 | 0.011 | 0.312 | 5.24E-05 | Stromal c | Emilin2 |
| 3.27E-09 | 1.122626 | 0.517 | 0.298 | 5.88E-05 | Stromal c | Lamb2 |
| 3.54E-09 | -1.5398 | 0.264 | 0.581 | 6.37E-05 | Stromal c | Ifi27l2a |
| 4.06E-09 | 1.037799 | 0.874 | 0.547 | 7.30E-05 | Stromal c | Cd81 |
| 4.12E-09 | -1.24495 | 0.046 | 0.345 | 7.42E-05 | Stromal c | Gadd45a |
| 4.37E-09 | -1.42915 | 0.034 | 0.336 | 7.87E-05 | Stromal c | Spon2 |
| 4.53E-09 | -1.28714 | 0.138 | 0.442 | 8.16E-05 | Stromal c | Tnxb |
| 4.79E-09 | -1.69316 | 0.034 | 0.329 | 8.63E-05 | Stromal c | Emb |
| 4.98E-09 | -2.84647 | 0.069 | 0.362 | 8.97E-05 | Stromal c | Mxd1 |
| 4.98E-09 | 1.1733 | 0.667 | 0.495 | 8.97E-05 | Stromal c | Aplp2 |
| 6.67E-09 | -1.80199 | 0.115 | 0.424 | 0.00012 | Stromal c | Mfap5 |
| 7.85E-09 | -1.18995 | 0.149 | 0.431 | 0.000141 | Stromal c | Cpq |
| 7.97E-09 | 1.401061 | 0.46 | 0.261 | 0.000144 | Stromal c | Lpp |
| 7.99E-09 | -1.05133 | 0.092 | 0.403 | 0.000144 | Stromal c | Itm2a |
| 9.04E-09 | -1.48894 | 0 | 0.283 | 0.000163 | Stromal c | Itgb2 |
| 9.33E-09 | -1.18315 | 0.057 | 0.362 | 0.000168 | Stromal c | Pcolce2 |
| 1.07E-08 | -1.34662 | 0.115 | 0.405 | 0.000193 | Stromal c | Islr |
| 1.37E-08 | 1.026676 | 0.276 | 0.105 | 0.000247 | Stromal c | Cdc42ep4 |
| 1.47E-08 | -1.30345 | 0.011 | 0.29 | 0.000264 | Stromal c | Arhgap45 |
| 1.53E-08 | -1.22305 | 0.046 | 0.332 | 0.000275 | Stromal c | Gfpt2 |
| 1.57E-08 | -1.68714 | 0.379 | 0.65 | 0.000284 | Stromal c | Taldo1 |
| 1.74E-08 | -1.57227 | 0.08 | 0.369 | 0.000313 | Stromal c | C3 |
| 1.78E-08 | 1.077459 | 0.713 | 0.582 | 0.000321 | Stromal c | Cox6a1 |
| 1.89E-08 | -1.69092 | 0.011 | 0.288 | 0.000341 | Stromal c | Ltb |
| 2.26E-08 | -1.51 | 0.069 | 0.353 | 0.000408 | Stromal c | Tnfaip2 |
| 2.44E-08 | 1.054458 | 0.264 | 0.1 | 0.000439 | Stromal c | Cbfa2t3 |
| 3.10E-08 | -1.10556 | 0.046 | 0.325 | 0.000558 | Stromal c | Gda |
| 3.14E-08 | -1.19256 | 0.161 | 0.447 | 0.000565 | Stromal c | Abca8a |
| 3.22E-08 | -2.11904 | 0.264 | 0.503 | 0.00058 | Stromal c | Col3a1 |
| 3.28E-08 | 1.089282 | 0.736 | 0.648 | 0.000591 | Stromal c | Atp5b |
| 3.41E-08 | -6.63387 | 0.184 | 0.468 | 0.000614 | Stromal c | S100a8 |
| 3.84E-08 | -1.52717 | 0 | 0.264 | 0.000691 | Stromal c | Ncf4 |
| 4.33E-08 | -1.16324 | 0.138 | 0.42 | 0.00078 | Stromal c | Rhog |
| 4.50E-08 | -1.91509 | 0.023 | 0.29 | 0.000809 | Stromal c | Samsn1 |
| 4.93E-08 | 1.009915 | 0.31 | 0.13 | 0.000888 | Stromal c | Ndrg1 |
| 5.01E-08 | -1.02306 | 0.161 | 0.453 | 0.000901 | Stromal c | R3hdm4 |
| 5.03E-08 | -1.31467 | 0 | 0.261 | 0.000906 | Stromal c | Cd37 |

| | | | | | | |
|---|---|---|---|---|---|---|
| 5.71E-08 | 1.084516 | 0.598 | 0.406 | 0.001028 | Stromal c | Ndufa5 |
| 6.25E-08 | 1.108414 | 0.356 | 0.169 | 0.001124 | Stromal c | Alad |
| 6.44E-08 | 1.602164 | 0.356 | 0.18 | 0.00116 | Stromal c | Wtip |
| 8.17E-08 | -1.80383 | 0.046 | 0.307 | 0.001472 | Stromal c | Slfn2 |
| 8.38E-08 | -1.23167 | 0.069 | 0.336 | 0.001508 | Stromal c | Fam49b |
| 9.35E-08 | -1.0748 | 0.046 | 0.315 | 0.001683 | Stromal c | Gsta3 |
| 9.91E-08 | -1.19371 | 0.218 | 0.451 | 0.001784 | Stromal c | Mxra8 |
| 1.12E-07 | -1.53198 | 0.011 | 0.267 | 0.002022 | Stromal c | Sell |
| 1.18E-07 | 1.662656 | 0.402 | 0.219 | 0.002126 | Stromal c | Cxcl12 |
| 1.20E-07 | -2.41582 | 0.08 | 0.339 | 0.002159 | Stromal c | Fcer1g |
| 1.35E-07 | -1.68445 | 0.31 | 0.565 | 0.002434 | Stromal c | Grina |
| 2.27E-07 | 1.549988 | 0.379 | 0.2 | 0.004089 | Stromal c | Slc9a3r2 |
| 2.37E-07 | -1.24851 | 0.092 | 0.358 | 0.00426 | Stromal c | Limd2 |
| 2.63E-07 | -1.29501 | 0.023 | 0.264 | 0.004726 | Stromal c | Hcls1 |
| 2.95E-07 | -1.05048 | 0.241 | 0.487 | 0.005303 | Stromal c | Lrp1 |
| 3.03E-07 | -1.14588 | 0.115 | 0.372 | 0.005453 | Stromal c | Col8a1 |
| 3.08E-07 | 2.000779 | 0.609 | 0.518 | 0.005541 | Stromal c | Dstn |
| 3.32E-07 | -1.76989 | 0.322 | 0.504 | 0.005982 | Stromal c | Col1a2 |
| 3.46E-07 | -5.46394 | 0.08 | 0.329 | 0.006222 | Stromal c | Retnlg |
| 3.74E-07 | -2.31358 | 0.046 | 0.288 | 0.006729 | Stromal c | Alox5ap |
| 3.81E-07 | -1.10964 | 0.195 | 0.466 | 0.006868 | Stromal c | Fbln2 |
| 4.13E-07 | 1.01115 | 0.632 | 0.459 | 0.007428 | Stromal c | Ndufb7 |
| 4.55E-07 | -1.18529 | 0.195 | 0.513 | 0.008195 | Stromal c | G0s2 |
| 4.78E-07 | -1.49707 | 0.563 | 0.841 | 0.008604 | Stromal c | Cebpb |
| 5.15E-07 | -1.4614 | 0.023 | 0.265 | 0.009265 | Stromal c | Cxcr4 |
| 6.24E-07 | 1.611184 | 0.494 | 0.352 | 0.011237 | Stromal c | Ppp1r14a |
| 7.04E-07 | -2.60291 | 0.023 | 0.253 | 0.012681 | Stromal c | Hp |
| 7.07E-07 | -1.5988 | 0.057 | 0.296 | 0.012737 | Stromal c | Neurl3 |
| 7.17E-07 | -1.82075 | 0.103 | 0.34 | 0.012911 | Stromal c | Rnf149 |
| 8.22E-07 | 1.029889 | 0.356 | 0.178 | 0.014795 | Stromal c | Tspan4 |
| 9.08E-07 | 1.013583 | 0.563 | 0.386 | 0.016342 | Stromal c | Ndufa12 |
| 1.12E-06 | -3.24798 | 0.034 | 0.263 | 0.020085 | Stromal c | Il1b |
| 1.13E-06 | 1.007083 | 0.402 | 0.24 | 0.02038 | Stromal c | Marveld1 |
| 1.20E-06 | 1.057638 | 0.494 | 0.339 | 0.021518 | Stromal c | Sh3bgrl |
| 1.29E-06 | -4.14559 | 0.08 | 0.31 | 0.023199 | Stromal c | Lyz2 |
| 1.37E-06 | -1.53725 | 0.034 | 0.256 | 0.024687 | Stromal c | Sorl1 |
| 1.45E-06 | -2.69188 | 0.046 | 0.272 | 0.026119 | Stromal c | Ccl6 |
| 1.50E-06 | -1.05687 | 0.149 | 0.403 | 0.026933 | Stromal c | Ogn |
| 1.58E-06 | -1.28673 | 0.253 | 0.467 | 0.02838 | Stromal c | Col6a2 |
| 1.75E-06 | -1.98739 | 0.057 | 0.285 | 0.031417 | Stromal c | Pglyrp1 |
| 1.78E-06 | -1.31143 | 0.149 | 0.389 | 0.031998 | Stromal c | Trib1 |
| 2.33E-06 | 1.153535 | 0.31 | 0.153 | 0.041921 | Stromal c | Tshz2 |
| 2.38E-06 | -1.48545 | 0.345 | 0.503 | 0.042883 | Stromal c | Pcolce |
| 0 | 5.133017 | 0.957 | 0.002 | 0 | Basophils | Cyp11a1 |
| 0 | 4.928959 | 0.87 | 0.001 | 0 | Basophils | Mcpt8 |
| 0 | 4.848309 | 0.435 | 0.002 | 0 | Basophils | Il4 |
| 0 | 4.010514 | 0.87 | 0.002 | 0 | Basophils | Gata2 |
| 0 | 3.980787 | 0.783 | 0.001 | 0 | Basophils | Cpa3 |
| 0 | 3.176374 | 0.761 | 0.001 | 0 | Basophils | Cd200r3 |
| 0 | 2.866436 | 0.457 | 0.003 | 0 | Basophils | Il13 |
| 0 | 2.736414 | 0.696 | 0 | 0 | Basophils | Fcer1a |
| 0 | 2.452967 | 0.674 | 0 | 0 | Basophils | Ms4a2 |
| 0 | 1.992254 | 0.543 | 0.001 | 0 | Basophils | Csrp3 |
| 0 | 1.44215 | 0.261 | 0 | 0 | Basophils | Slc6a4 |

| | | | | | | |
|---|---|---|---|---|---|---|
| 0 | 1.282541 | 0.348 | 0 | 0 | Basophils | Alox15 |
| 0 | 1.254257 | 0.304 | 0.002 | 0 | Basophils | Npl |
| 0 | 1.187841 | 0.261 | 0.001 | 0 | Basophils | Slc18a2 |
| 0 | 1.177799 | 0.283 | 0 | 0 | Basophils | Grm6 |
| ######## | 2.463187 | 0.522 | 0.009 | ######## | Basophils | Hgf |
| ######## | 3.335507 | 0.674 | 0.022 | ######## | Basophils | Csf2rb2 |
| ######## | 1.862434 | 0.413 | 0.01 | ######## | Basophils | C3ar1 |
| ######## | 5.006119 | 0.913 | 0.058 | ######## | Basophils | Ccl9 |
| ######## | 2.760116 | 0.804 | 0.043 | ######## | Basophils | Lat2 |
| ######## | 2.074567 | 0.478 | 0.017 | ######## | Basophils | Klf5 |
| ######## | 2.1662 | 0.609 | 0.029 | ######## | Basophils | Sytl3 |
| ######## | 1.629313 | 0.478 | 0.017 | ######## | Basophils | Cdh1 |
| 2.33E-94 | 1.429545 | 0.478 | 0.022 | 4.20E-90 | Basophils | Matk |
| 3.45E-92 | 3.844019 | 0.891 | 0.089 | 6.21E-88 | Basophils | Il6 |
| 1.01E-90 | 6.958724 | 0.935 | 0.106 | 1.83E-86 | Basophils | Ccl3 |
| 3.98E-87 | 1.605731 | 0.348 | 0.013 | 7.16E-83 | Basophils | Fosl1 |
| 1.89E-83 | 1.648989 | 0.348 | 0.014 | 3.41E-79 | Basophils | Itga2b |
| 3.87E-78 | 6.733433 | 0.978 | 0.138 | 6.96E-74 | Basophils | Ccl4 |
| 5.24E-72 | 3.394832 | 0.739 | 0.078 | 9.43E-68 | Basophils | Slc7a5 |
| 3.45E-68 | 3.788886 | 0.913 | 0.125 | 6.22E-64 | Basophils | Osm |
| 1.76E-62 | 1.810694 | 0.565 | 0.047 | 3.17E-58 | Basophils | Il18r1 |
| 3.41E-54 | 2.19852 | 0.543 | 0.05 | 6.13E-50 | Basophils | Cd7 |
| 7.29E-53 | 4.063803 | 1 | 0.24 | 1.31E-48 | Basophils | Hdc |
| 4.69E-51 | 3.078176 | 0.609 | 0.071 | 8.45E-47 | Basophils | Serpinb1a |
| 8.78E-51 | 1.103374 | 0.304 | 0.017 | 1.58E-46 | Basophils | Specc1 |
| 1.16E-50 | 1.702497 | 0.391 | 0.028 | 2.09E-46 | Basophils | Adora2b |
| 3.55E-50 | 4.163903 | 0.717 | 0.102 | 6.40E-46 | Basophils | Cks2 |
| 3.72E-50 | 4.233626 | 1 | 0.236 | 6.70E-46 | Basophils | Ifitm1 |
| 7.61E-50 | 1.582505 | 0.413 | 0.032 | 1.37E-45 | Basophils | Tec |
| 3.51E-49 | 3.400082 | 0.978 | 0.229 | 6.31E-45 | Basophils | Lilr4b |
| 2.06E-48 | 2.811774 | 0.739 | 0.109 | 3.71E-44 | Basophils | Csf2rb |
| 3.93E-48 | 1.735876 | 0.478 | 0.045 | 7.08E-44 | Basophils | Tbc1d4 |
| 6.84E-48 | 1.342553 | 0.261 | 0.013 | 1.23E-43 | Basophils | Slc7a8 |
| 8.18E-47 | 1.640636 | 0.543 | 0.058 | 1.47E-42 | Basophils | Atp1b1 |
| 1.20E-44 | 4.00087 | 0.783 | 0.14 | 2.17E-40 | Basophils | Acod1 |
| 8.34E-44 | 1.701203 | 0.457 | 0.043 | 1.50E-39 | Basophils | Aqp9 |
| 5.75E-43 | 4.201494 | 0.522 | 0.058 | 1.04E-38 | Basophils | Gzmb |
| 8.01E-43 | 1.625213 | 0.478 | 0.048 | 1.44E-38 | Basophils | Lilrb4a |
| 4.19E-42 | 1.28822 | 0.37 | 0.029 | 7.55E-38 | Basophils | Cd244 |
| 1.13E-41 | 2.744674 | 0.826 | 0.16 | 2.04E-37 | Basophils | Trf |
| 9.38E-41 | 1.911006 | 0.674 | 0.1 | 1.69E-36 | Basophils | Itk |
| 6.48E-40 | 1.352299 | 0.326 | 0.025 | 1.17E-35 | Basophils | Padi2 |
| 1.08E-39 | 1.584439 | 0.457 | 0.048 | 1.94E-35 | Basophils | Dapp1 |
| 2.39E-39 | 1.268241 | 0.391 | 0.035 | 4.29E-35 | Basophils | L1cam |
| 5.94E-39 | 1.475783 | 0.435 | 0.044 | 1.07E-34 | Basophils | Mapkapk3 |
| 8.56E-39 | 2.988065 | 0.891 | 0.218 | 1.54E-34 | Basophils | Plek |
| 1.57E-38 | 3.296592 | 0.761 | 0.151 | 2.83E-34 | Basophils | Ptgs2 |
| 8.50E-38 | 3.101927 | 0.652 | 0.112 | 1.53E-33 | Basophils | Ero1l |
| 1.99E-37 | 1.994383 | 0.739 | 0.139 | 3.59E-33 | Basophils | Tnfaip8 |
| 2.44E-36 | 1.075738 | 0.283 | 0.02 | 4.40E-32 | Basophils | Mboat1 |
| 1.36E-35 | 1.679745 | 0.587 | 0.083 | 2.44E-31 | Basophils | Il7r |
| 8.15E-35 | 2.779082 | 0.63 | 0.106 | 1.47E-30 | Basophils | Nlrp3 |
| 1.11E-34 | 1.956059 | 0.761 | 0.148 | 2.00E-30 | Basophils | Itgb7 |
| 5.49E-34 | 3.16303 | 0.696 | 0.132 | 9.89E-30 | Basophils | Rgs1 |

| | | | | | | |
|---|---|---|---|---|---|---|
| 5.37E-33 | 1.531496 | 0.5 | 0.066 | 9.66E-29 | Basophils | Alox5 |
| 1.82E-30 | 1.219176 | 0.435 | 0.053 | 3.28E-26 | Basophils | Rab44 |
| 2.24E-30 | 1.940423 | 0.783 | 0.176 | 4.03E-26 | Basophils | Ncf1 |
| 6.89E-30 | 2.102585 | 0.957 | 0.286 | 1.24E-25 | Basophils | Alox5ap |
| 9.22E-30 | 4.161165 | 0.957 | 0.442 | 1.66E-25 | Basophils | Ier3 |
| 2.55E-29 | 2.464707 | 0.804 | 0.217 | 4.60E-25 | Basophils | Cyp4f18 |
| 5.78E-29 | 3.637104 | 0.565 | 0.107 | 1.04E-24 | Basophils | Gm20186 |
| 1.12E-26 | 1.854711 | 0.935 | 0.305 | 2.02E-22 | Basophils | Lgals3 |
| 1.55E-26 | 1.406543 | 0.391 | 0.053 | 2.78E-22 | Basophils | Stx3 |
| 1.26E-25 | 1.266072 | 0.37 | 0.047 | 2.27E-21 | Basophils | Hist1h1e |
| 4.27E-25 | 1.539522 | 0.587 | 0.111 | 7.68E-21 | Basophils | Plac8 |
| 5.54E-25 | 2.360946 | 0.848 | 0.31 | 9.98E-21 | Basophils | Emilin2 |
| 6.11E-25 | 3.225477 | 0.804 | 0.27 | 1.10E-20 | Basophils | Ccl6 |
| 1.14E-24 | 1.358701 | 0.37 | 0.048 | 2.06E-20 | Basophils | Tespa1 |
| 1.30E-24 | 2.304173 | 0.783 | 0.234 | 2.33E-20 | Basophils | Tnfaip3 |
| 3.04E-24 | 1.020705 | 0.326 | 0.039 | 5.47E-20 | Basophils | Cish |
| 1.15E-23 | 2.68891 | 0.913 | 0.591 | 2.06E-19 | Basophils | Sub1 |
| 3.34E-23 | 1.307566 | 0.391 | 0.056 | 6.01E-19 | Basophils | Pmaip1 |
| 3.29E-22 | 2.730568 | 0.87 | 0.497 | 5.92E-18 | Basophils | Tax1bp1 |
| 1.01E-21 | 2.221058 | 0.761 | 0.256 | 1.81E-17 | Basophils | Plk3 |
| 1.02E-21 | 1.212981 | 0.478 | 0.085 | 1.83E-17 | Basophils | Il18rap |
| 1.40E-21 | 1.537801 | 0.87 | 0.287 | 2.52E-17 | Basophils | Samsn1 |
| 1.51E-21 | 3.005349 | 0.717 | 0.25 | 2.71E-17 | Basophils | Csf1 |
| 3.49E-21 | 1.781981 | 0.935 | 0.389 | 6.29E-17 | Basophils | Cd53 |
| 4.15E-21 | 2.320744 | 0.717 | 0.241 | 7.47E-17 | Basophils | Ddit4 |
| 5.73E-21 | 1.198703 | 0.543 | 0.108 | 1.03E-16 | Basophils | Napsa |
| 1.04E-20 | 1.482927 | 0.978 | 0.336 | 1.87E-16 | Basophils | Fcer1g |
| 4.29E-20 | 1.537265 | 0.348 | 0.052 | 7.73E-16 | Basophils | Gcnt1 |
| 1.14E-19 | 2.211403 | 0.478 | 0.104 | 2.05E-15 | Basophils | Hilpda |
| 1.95E-19 | 1.652289 | 0.413 | 0.078 | 3.51E-15 | Basophils | Nabp1 |
| 2.15E-19 | 2.461252 | 0.957 | 0.723 | 3.87E-15 | Basophils | Neat1 |
| 6.32E-19 | 2.589799 | 0.717 | 0.271 | 1.14E-14 | Basophils | Furin |
| 1.11E-18 | 1.695285 | 0.413 | 0.075 | 1.99E-14 | Basophils | Gpr183 |
| 1.50E-18 | 1.170161 | 0.543 | 0.123 | 2.70E-14 | Basophils | Ptpre |
| 1.80E-18 | -2.99574 | 0.37 | 0.865 | 3.25E-14 | Basophils | S100a6 |
| 2.19E-18 | 1.700115 | 0.435 | 0.092 | 3.94E-14 | Basophils | Frmd4b |
| 3.47E-18 | 1.965684 | 0.848 | 0.52 | 6.25E-14 | Basophils | Mpc2 |
| 5.02E-18 | 1.770924 | 0.957 | 0.769 | 9.04E-14 | Basophils | Fxyd5 |
| 5.21E-18 | 1.097932 | 0.37 | 0.064 | 9.38E-14 | Basophils | Spn |
| 5.85E-18 | 1.136113 | 0.37 | 0.063 | 1.05E-13 | Basophils | Inpp4b |
| 5.85E-18 | 1.922996 | 0.87 | 0.454 | 1.05E-13 | Basophils | Nfkbiz |
| 6.60E-18 | 2.166219 | 0.478 | 0.105 | 1.19E-13 | Basophils | Cd69 |
| 8.70E-18 | 1.038808 | 0.326 | 0.05 | 1.57E-13 | Basophils | Cst7 |
| 1.26E-16 | 1.419772 | 0.522 | 0.146 | 2.27E-12 | Basophils | Nt5c3 |
| 1.49E-16 | 1.016692 | 0.326 | 0.054 | 2.68E-12 | Basophils | Itgb3 |
| 1.72E-16 | 1.313696 | 0.478 | 0.123 | 3.10E-12 | Basophils | Coq8b |
| 1.82E-16 | 1.277128 | 0.37 | 0.071 | 3.27E-12 | Basophils | Orai2 |
| 2.15E-16 | 1.450274 | 1 | 0.557 | 3.87E-12 | Basophils | Srgn |
| 2.59E-16 | 1.761517 | 0.935 | 0.674 | 4.67E-12 | Basophils | Tagln2 |
| 3.00E-16 | 1.690385 | 0.783 | 0.301 | 5.41E-12 | Basophils | Fam107b |
| 3.45E-16 | 1.371116 | 0.478 | 0.119 | 6.20E-12 | Basophils | Jak2 |
| 1.42E-15 | 1.554057 | 0.457 | 0.113 | 2.56E-11 | Basophils | Klhl6 |
| 1.69E-15 | 1.581057 | 0.652 | 0.221 | 3.04E-11 | Basophils | Dgat1 |
| 2.04E-15 | 2.061218 | 0.891 | 0.601 | 3.68E-11 | Basophils | Cd9 |

| | | | | | | |
|---|---|---|---|---|---|---|
| 2.62E-15 | -2.09202 | 0.304 | 0.804 | 4.73E-11 | Basophils | Psap |
| 9.03E-15 | 2.21486 | 0.913 | 0.691 | 1.63E-10 | Basophils | Nfkbia |
| 1.72E-14 | 1.616559 | 0.522 | 0.148 | 3.10E-10 | Basophils | Baz1a |
| 1.91E-14 | 1.143036 | 0.5 | 0.13 | 3.45E-10 | Basophils | Hacd4 |
| 2.45E-14 | 1.535075 | 0.652 | 0.273 | 4.41E-10 | Basophils | Pfkp |
| 4.25E-14 | 2.310946 | 0.457 | 0.131 | 7.65E-10 | Basophils | Hs3st1 |
| 7.99E-14 | 1.312365 | 0.478 | 0.142 | 1.44E-09 | Basophils | Rfc2 |
| 8.47E-14 | 1.335993 | 0.717 | 0.263 | 1.53E-09 | Basophils | Cxcr4 |
| 9.56E-14 | 1.328301 | 0.935 | 0.812 | 1.72E-09 | Basophils | Pnrc1 |
| 1.03E-13 | 1.622261 | 0.63 | 0.212 | 1.86E-09 | Basophils | Dusp5 |
| 1.54E-13 | 2.667237 | 0.783 | 0.532 | 2.78E-09 | Basophils | Ifrd1 |
| 1.87E-13 | 1.200742 | 0.37 | 0.087 | 3.37E-09 | Basophils | Tle1 |
| 3.82E-13 | 1.17793 | 0.804 | 0.327 | 6.88E-09 | Basophils | Emb |
| 5.58E-13 | 1.067616 | 0.978 | 0.838 | 1.00E-08 | Basophils | Rpl38 |
| 8.35E-13 | 1.691589 | 0.717 | 0.377 | 1.50E-08 | Basophils | Rab11a |
| 1.10E-12 | 1.299346 | 0.761 | 0.311 | 1.99E-08 | Basophils | Hcst |
| 1.27E-12 | -1.91831 | 0 | 0.582 | 2.29E-08 | Basophils | Fkbp1a |
| 1.43E-12 | -3.10961 | 0 | 0.581 | 2.57E-08 | Basophils | Ifi27l2a |
| 2.22E-12 | 1.180139 | 0.652 | 0.24 | 4.00E-08 | Basophils | Fyb |
| 2.46E-12 | 1.231272 | 0.304 | 0.065 | 4.43E-08 | Basophils | Cables1 |
| 2.73E-12 | 1.10219 | 0.587 | 0.193 | 4.92E-08 | Basophils | 1-Sep |
| 4.86E-12 | 1.23122 | 1 | 0.989 | 8.76E-08 | Basophils | Ftl1 |
| 6.88E-12 | -3.86952 | 0.174 | 0.656 | 1.24E-07 | Basophils | Cebpd |
| 8.85E-12 | 1.258343 | 0.826 | 0.446 | 1.59E-07 | Basophils | Vasp |
| 1.03E-11 | 1.01633 | 0.652 | 0.222 | 1.86E-07 | Basophils | Fcgr3 |
| 1.29E-11 | -2.39672 | 0.043 | 0.578 | 2.33E-07 | Basophils | Selenop |
| 1.40E-11 | 1.410197 | 0.522 | 0.189 | 2.53E-07 | Basophils | Zc3h12a |
| 1.84E-11 | 1.621124 | 0.652 | 0.315 | 3.32E-07 | Basophils | Clint1 |
| 1.94E-11 | -2.79751 | 0 | 0.544 | 3.48E-07 | Basophils | Crispld2 |
| 1.96E-11 | -2.11643 | 0.13 | 0.645 | 3.53E-07 | Basophils | Timp2 |
| 1.97E-11 | 1.156038 | 0.891 | 0.76 | 3.55E-07 | Basophils | Rap1b |
| 1.99E-11 | 1.393789 | 0.717 | 0.398 | 3.58E-07 | Basophils | Rnf130 |
| 2.34E-11 | 1.946016 | 0.826 | 0.558 | 4.21E-07 | Basophils | Pim1 |
| 2.42E-11 | -1.93854 | 0 | 0.541 | 4.35E-07 | Basophils | App |
| 3.14E-11 | -1.80681 | 0.261 | 0.711 | 5.66E-07 | Basophils | Ifitm3 |
| 3.56E-11 | 1.251401 | 0.543 | 0.204 | 6.41E-07 | Basophils | Mboat7 |
| 3.59E-11 | 1.362725 | 1 | 0.976 | 6.47E-07 | Basophils | Actg1 |
| 5.78E-11 | 1.095087 | 0.5 | 0.166 | 1.04E-06 | Basophils | Smim3 |
| 5.89E-11 | -2.25706 | 0.652 | 0.845 | 1.06E-06 | Basophils | Cst3 |
| 6.07E-11 | -1.67137 | 0 | 0.527 | 1.09E-06 | Basophils | Tnfrsf1a |
| 6.95E-11 | 1.101904 | 0.696 | 0.322 | 1.25E-06 | Basophils | Man2b1 |
| 7.49E-11 | 1.520758 | 0.674 | 0.299 | 1.35E-06 | Basophils | Rgs2 |
| 7.52E-11 | 1.295345 | 0.522 | 0.189 | 1.35E-06 | Basophils | D16Ertd472e |
| 8.49E-11 | -3.64526 | 0 | 0.522 | 1.53E-06 | Basophils | Lum |
| 9.82E-11 | -3.93958 | 0.022 | 0.534 | 1.77E-06 | Basophils | Htra3 |
| 1.21E-10 | -1.91473 | 0 | 0.516 | 2.19E-06 | Basophils | Marcks |
| 1.30E-10 | 1.337669 | 0.348 | 0.096 | 2.35E-06 | Basophils | Stk19 |
| 1.37E-10 | 1.197079 | 0.391 | 0.109 | 2.47E-06 | Basophils | Tmem71 |
| 1.56E-10 | -3.237 | 0 | 0.513 | 2.80E-06 | Basophils | G0s2 |
| 2.26E-10 | -1.66868 | 0.065 | 0.573 | 4.07E-06 | Basophils | Slc25a4 |
| 2.39E-10 | -1.80584 | 0.022 | 0.526 | 4.31E-06 | Basophils | Ybx3 |
| 2.63E-10 | -3.27809 | 0 | 0.504 | 4.74E-06 | Basophils | Gpx3 |
| 2.66E-10 | -1.53732 | 0.065 | 0.576 | 4.78E-06 | Basophils | Ctsz |
| 2.68E-10 | -2.2687 | 0 | 0.504 | 4.83E-06 | Basophils | Pmepa1 |

| | | | | | | |
|---|---|---|---|---|---|---|
| 2.87E-10 | -1.5957 | 0.043 | 0.539 | 5.17E-06 | Basophils | Ifi27 |
| 2.94E-10 | 1.145755 | 0.87 | 0.72 | 5.30E-06 | Basophils | Sh3bgrl3 |
| 3.20E-10 | -4.07242 | 0.043 | 0.528 | 5.77E-06 | Basophils | Sparc |
| 4.35E-10 | -2.10645 | 0 | 0.497 | 7.84E-06 | Basophils | Selenom |
| 4.84E-10 | -2.70608 | 0.022 | 0.52 | 8.71E-06 | Basophils | Socs3 |
| 5.39E-10 | -3.74203 | 0.065 | 0.533 | 9.71E-06 | Basophils | Sparcl1 |
| 5.41E-10 | -2.69185 | 0.065 | 0.541 | 9.74E-06 | Basophils | Igfbp4 |
| 5.69E-10 | -1.93619 | 0.043 | 0.544 | 1.02E-05 | Basophils | Mgst1 |
| 6.00E-10 | -5.39814 | 0.239 | 0.643 | 1.08E-05 | Basophils | Dcn |
| 6.47E-10 | 1.519057 | 0.261 | 0.058 | 1.16E-05 | Basophils | Slco2b1 |
| 6.48E-10 | -3.21852 | 0 | 0.49 | 1.17E-05 | Basophils | Col1a1 |
| 6.68E-10 | -1.49064 | 0.565 | 0.794 | 1.20E-05 | Basophils | mt-Nd1 |
| 6.76E-10 | 1.078848 | 0.326 | 0.085 | 1.22E-05 | Basophils | Runx1 |
| 7.24E-10 | -3.98008 | 0.087 | 0.543 | 1.30E-05 | Basophils | Bgn |
| 7.46E-10 | -2.96432 | 0.022 | 0.503 | 1.34E-05 | Basophils | Pcolce |
| 7.67E-10 | 1.262436 | 0.413 | 0.129 | 1.38E-05 | Basophils | Ripor2 |
| 7.82E-10 | -3.38626 | 0.043 | 0.515 | 1.41E-05 | Basophils | Serping1 |
| 7.91E-10 | -2.73904 | 0.022 | 0.503 | 1.42E-05 | Basophils | Cygb |
| 7.93E-10 | -2.09344 | 0 | 0.487 | 1.43E-05 | Basophils | Lrp1 |
| 8.86E-10 | -1.54162 | 0.043 | 0.523 | 1.59E-05 | Basophils | Ddost |
| 1.03E-09 | 2.066228 | 0.63 | 0.294 | 1.85E-05 | Basophils | Neurl3 |
| 1.06E-09 | -1.00964 | 0.261 | 0.779 | 1.91E-05 | Basophils | Sem1 |
| 1.16E-09 | -3.03597 | 0.022 | 0.504 | 2.09E-05 | Basophils | Col1a2 |
| 1.16E-09 | -2.55962 | 0 | 0.481 | 2.10E-05 | Basophils | Cfh |
| 1.48E-09 | 1.487577 | 0.652 | 0.286 | 2.66E-05 | Basophils | Ltb |
| 1.53E-09 | 1.213959 | 0.848 | 0.642 | 2.75E-05 | Basophils | Litaf |
| 1.54E-09 | 1.98325 | 0.739 | 0.517 | 2.77E-05 | Basophils | Sqstm1 |
| 1.60E-09 | -2.24092 | 0.087 | 0.536 | 2.88E-05 | Basophils | Zbtb20 |
| 1.68E-09 | -2.26497 | 0 | 0.475 | 3.02E-05 | Basophils | Dpt |
| 1.81E-09 | -3.10027 | 0.022 | 0.49 | 3.26E-05 | Basophils | Serpinh1 |
| 1.82E-09 | 1.03125 | 0.348 | 0.103 | 3.27E-05 | Basophils | Mob3c |
| 1.82E-09 | 1.407518 | 0.587 | 0.297 | 3.27E-05 | Basophils | Nfil3 |
| 1.85E-09 | -3.58439 | 0.043 | 0.503 | 3.33E-05 | Basophils | Col3a1 |
| 1.85E-09 | 1.170075 | 0.739 | 0.38 | 3.34E-05 | Basophils | Gmfg |
| 1.94E-09 | -2.10607 | 0 | 0.472 | 3.49E-05 | Basophils | Pmp22 |
| 2.32E-09 | -1.76806 | 0.022 | 0.489 | 4.18E-05 | Basophils | Nenf |
| 2.34E-09 | -1.78122 | 0.109 | 0.567 | 4.21E-05 | Basophils | Cyb5a |
| 2.46E-09 | -3.09066 | 0.022 | 0.485 | 4.44E-05 | Basophils | Fbln1 |
| 2.49E-09 | -2.43595 | 0.022 | 0.484 | 4.48E-05 | Basophils | Mmp2 |
| 2.69E-09 | -2.49609 | 0 | 0.467 | 4.85E-05 | Basophils | Col6a2 |
| 2.95E-09 | -4.41297 | 0.152 | 0.569 | 5.31E-05 | Basophils | Mgp |
| 3.12E-09 | -1.40524 | 0.022 | 0.494 | 5.61E-05 | Basophils | Hmgn1 |
| 3.12E-09 | -3.61566 | 0.022 | 0.481 | 5.63E-05 | Basophils | Lpl |
| 3.22E-09 | -2.479 | 0.13 | 0.549 | 5.79E-05 | Basophils | Cd81 |
| 3.50E-09 | 2.082495 | 0.5 | 0.231 | 6.30E-05 | Basophils | Prkd3 |
| 3.60E-09 | -2.55924 | 0.022 | 0.477 | 6.48E-05 | Basophils | Col6a1 |
| 3.99E-09 | -2.20886 | 0 | 0.46 | 7.19E-05 | Basophils | Nid1 |
| 4.42E-09 | -1.97919 | 0 | 0.458 | 7.95E-05 | Basophils | Phlda1 |
| 4.77E-09 | -2.4373 | 0.022 | 0.474 | 8.59E-05 | Basophils | Serpinf1 |
| 5.23E-09 | 1.187802 | 0.652 | 0.362 | 9.42E-05 | Basophils | Plp2 |
| 5.57E-09 | -1.92196 | 0.022 | 0.489 | 0.0001 | Basophils | Lhfp |
| 5.62E-09 | 1.63761 | 0.848 | 0.595 | 0.000101 | Basophils | Anxa1 |
| 5.75E-09 | -2.72138 | 0.087 | 0.515 | 0.000104 | Basophils | Ltbp4 |
| 5.83E-09 | -3.35211 | 0.087 | 0.514 | 0.000105 | Basophils | Clec3b |

| | | | | | |
|---|---|---|---|---|---|
| 6.02E-09 | -2.0353 | 0 | 0.453 | 0.000108 | BasophilsHspg2 |
| 6.04E-09 | -1.30537 | 0.087 | 0.543 | 0.000109 | BasophilsUqcrb |
| 6.07E-09 | -1.88549 | 0 | 0.453 | 0.000109 | BasophilsSlc43a3 |
| 6.34E-09 | -1.50357 | 0.174 | 0.611 | 0.000114 | BasophilsCtsb |
| 6.41E-09 | -2.42062 | 0.022 | 0.471 | 0.000115 | BasophilsId3 |
| 6.52E-09 | -1.46985 | 0.022 | 0.478 | 0.000117 | BasophilsDhrs7 |
| 6.66E-09 | -2.14023 | 0.043 | 0.503 | 0.00012 | BasophilsRarres2 |
| 6.70E-09 | -1.4508 | 0.196 | 0.647 | 0.000121 | BasophilsIfngr1 |
| 6.82E-09 | -2.77404 | 0.043 | 0.487 | 0.000123 | BasophilsKlf4 |
| 6.91E-09 | -1.7867 | 0 | 0.451 | 0.000124 | BasophilsMxra8 |
| 7.09E-09 | -2.38369 | 0.022 | 0.47 | 0.000128 | BasophilsAtf3 |
| 7.72E-09 | -1.91653 | 0 | 0.449 | 0.000139 | BasophilsLoxl1 |
| 8.32E-09 | -1.65293 | 0.326 | 0.676 | 0.00015 | BasophilsCalr |
| 8.43E-09 | -1.9781 | 0.022 | 0.467 | 0.000152 | BasophilsAxl |
| 8.46E-09 | -1.57674 | 0.022 | 0.478 | 0.000152 | BasophilsAtp2b1 |
| 9.01E-09 | 1.17783 | 0.5 | 0.21 | 0.000162 | BasophilsIkbkb |
| 1.02E-08 | -2.33535 | 0.022 | 0.466 | 0.000184 | BasophilsFbln2 |
| 1.06E-08 | -1.45312 | 0 | 0.443 | 0.00019 | BasophilsFcgrt |
| 1.14E-08 | -1.63718 | 0.022 | 0.471 | 0.000205 | BasophilsCd302 |
| 1.14E-08 | -2.03004 | 0 | 0.442 | 0.000205 | BasophilsCol4a1 |
| 1.15E-08 | -1.13112 | 0.196 | 0.627 | 0.000206 | BasophilsMrfap1 |
| 1.19E-08 | -1.65652 | 0 | 0.441 | 0.000213 | BasophilsRhoj |
| 1.42E-08 | -2.3173 | 0.022 | 0.466 | 0.000256 | BasophilsCcdc80 |
| 1.44E-08 | -1.3715 | 0 | 0.438 | 0.00026 | BasophilsTxndc5 |
| 1.47E-08 | 1.232514 | 0.652 | 0.373 | 0.000265 | BasophilsEtf1 |
| 1.53E-08 | 1.267772 | 0.326 | 0.101 | 0.000275 | BasophilsBri3bp |
| 1.57E-08 | -1.80043 | 0.239 | 0.639 | 0.000282 | BasophilsTuba1a |
| 1.60E-08 | -1.5499 | 0.609 | 0.77 | 0.000288 | Basophilsmt-Nd2 |
| 1.62E-08 | 1.380457 | 0.957 | 0.971 | 0.000292 | BasophilsH3f3b |
| 1.64E-08 | -1.87818 | 0 | 0.436 | 0.000295 | BasophilsPlpp3 |
| 1.73E-08 | -2.35157 | 0.022 | 0.456 | 0.000312 | BasophilsCol15a1 |
| 1.74E-08 | -2.70086 | 0.022 | 0.452 | 0.000313 | BasophilsLy6a |
| 1.75E-08 | -1.75312 | 0 | 0.434 | 0.000315 | BasophilsGpm6b |
| 1.79E-08 | -1.86297 | 0.022 | 0.46 | 0.000322 | BasophilsNfix |
| 1.83E-08 | -1.38366 | 0.13 | 0.569 | 0.000329 | BasophilsAhnak |
| 1.85E-08 | -2.31668 | 0.065 | 0.488 | 0.000333 | BasophilsCd34 |
| 1.92E-08 | -1.5761 | 0.435 | 0.774 | 0.000345 | BasophilsTxn1 |
| 1.96E-08 | -1.42168 | 0.087 | 0.536 | 0.000353 | BasophilsZfp36l1 |
| 2.05E-08 | -1.23318 | 0.761 | 0.831 | 0.000368 | BasophilsRps2 |
| 2.06E-08 | -2.18601 | 0.043 | 0.468 | 0.000372 | BasophilsFstl1 |
| 2.21E-08 | -1.78448 | 0.13 | 0.539 | 0.000398 | BasophilsMtch1 |
| 2.28E-08 | -1.7869 | 0.022 | 0.462 | 0.00041 | BasophilsCrip2 |
| 2.46E-08 | -1.5699 | 0.022 | 0.451 | 0.000443 | BasophilsCamk2n1 |
| 2.53E-08 | 1.607006 | 0.37 | 0.119 | 0.000456 | BasophilsCtsc |
| 2.62E-08 | -1.17938 | 0.739 | 0.807 | 0.000472 | BasophilsPtma |
| 2.66E-08 | -1.65347 | 0.022 | 0.448 | 0.000478 | BasophilsNfib |
| 2.73E-08 | -1.76902 | 0 | 0.426 | 0.000492 | BasophilsEbf1 |
| 2.75E-08 | -1.1416 | 0.435 | 0.827 | 0.000495 | BasophilsTsc22d3 |
| 2.81E-08 | 1.048072 | 0.587 | 0.287 | 0.000506 | BasophilsPrkcd |
| 2.97E-08 | -1.77984 | 0.043 | 0.468 | 0.000535 | BasophilsLamc1 |
| 3.12E-08 | -1.17425 | 0.043 | 0.5 | 0.000561 | BasophilsMyadm |
| 3.17E-08 | -2.62204 | 0 | 0.424 | 0.000572 | BasophilsMfap5 |
| 3.19E-08 | -1.65892 | 0 | 0.424 | 0.000575 | BasophilsOlfml3 |
| 3.21E-08 | -1.48736 | 0.022 | 0.455 | 0.000577 | BasophilsRhoc |

| | | | | | | |
|---|---|---|---|---|---|---|
| 3.36E-08 | 1.009983 | 0.87 | 0.668 | 0.000605 | Basophils | S100a10 |
| 3.58E-08 | -1.28 | 0.804 | 0.843 | 0.000644 | Basophils | mt-Cytb |
| 3.58E-08 | -1.81489 | 0 | 0.421 | 0.000645 | Basophils | Lamb1 |
| 3.65E-08 | -1.72468 | 0.043 | 0.457 | 0.000658 | Basophils | Sdc2 |
| 3.77E-08 | -1.11426 | 0.283 | 0.648 | 0.00068 | Basophils | Prdx1 |
| 3.91E-08 | -1.23215 | 0.283 | 0.706 | 0.000703 | Basophils | Rpl22l1 |
| 4.28E-08 | -1.68192 | 0.022 | 0.447 | 0.000771 | Basophils | Abca8a |
| 4.65E-08 | -1.52576 | 0.261 | 0.711 | 0.000837 | Basophils | Lsp1 |
| 4.68E-08 | -1.11707 | 0.022 | 0.442 | 0.000842 | Basophils | Ndufc2 |
| 4.71E-08 | -1.4504 | 0 | 0.416 | 0.000848 | Basophils | Bicc1 |
| 4.73E-08 | -1.72047 | 0 | 0.416 | 0.000852 | Basophils | Spry1 |
| 4.83E-08 | -1.30698 | 0.261 | 0.661 | 0.000869 | Basophils | mt-Nd3 |
| 5.00E-08 | -1.63909 | 0 | 0.415 | 0.0009 | Basophils | Tcf21 |
| 5.05E-08 | -3.24236 | 0 | 0.415 | 0.000909 | Basophils | Hspb1 |
| 5.54E-08 | 1.169983 | 0.63 | 0.392 | 0.000997 | Basophils | Nfkb1 |
| 5.71E-08 | -2.31587 | 0.043 | 0.461 | 0.001028 | Basophils | Smoc2 |
| 5.71E-08 | -1.50209 | 0 | 0.413 | 0.001029 | Basophils | Cavin3 |
| 6.11E-08 | -1.52756 | 0 | 0.412 | 0.0011 | Basophils | Ptgis |
| 6.45E-08 | -1.34076 | 0 | 0.411 | 0.001161 | Basophils | Slc29a1 |
| 6.69E-08 | -2.04693 | 0.022 | 0.432 | 0.001205 | Basophils | Gas1 |
| 6.86E-08 | -1.41387 | 0 | 0.409 | 0.001235 | Basophils | Ddah2 |
| 7.73E-08 | -1.0325 | 0 | 0.407 | 0.001392 | Basophils | Ndufa5 |
| 8.28E-08 | -1.42632 | 0 | 0.406 | 0.001491 | Basophils | Lama2 |
| 8.37E-08 | -1.4984 | 0.283 | 0.615 | 0.001507 | Basophils | Laptm4a |
| 8.56E-08 | -1.64791 | 0.022 | 0.44 | 0.001541 | Basophils | Errfi1 |
| 8.78E-08 | -1.71065 | 0 | 0.405 | 0.001581 | Basophils | Islr |
| 8.84E-08 | -1.76602 | 0.283 | 0.606 | 0.001592 | Basophils | Anxa5 |
| 9.06E-08 | -1.56269 | 0.065 | 0.478 | 0.001631 | Basophils | Tcf4 |
| 9.64E-08 | -1.81376 | 0 | 0.403 | 0.001736 | Basophils | Ogn |
| 1.04E-07 | -1.04876 | 0.283 | 0.703 | 0.001868 | Basophils | Ndufa13 |
| 1.06E-07 | 1.324217 | 0.739 | 0.546 | 0.001917 | Basophils | Ncor1 |
| 1.08E-07 | -1.77073 | 0.022 | 0.432 | 0.001947 | Basophils | Pcsk6 |
| 1.15E-07 | -1.81596 | 0.065 | 0.461 | 0.002073 | Basophils | Rbp1 |
| 1.20E-07 | -1.25778 | 0 | 0.399 | 0.002156 | Basophils | S100a16 |
| 1.21E-07 | -1.51792 | 0 | 0.399 | 0.002178 | Basophils | Ramp2 |
| 1.34E-07 | 1.424118 | 0.761 | 0.517 | 0.002418 | Basophils | Cdkn1a |
| 1.36E-07 | -1.52523 | 0 | 0.397 | 0.002443 | Basophils | Pam |
| 1.40E-07 | -3.50183 | 0.152 | 0.539 | 0.002517 | Basophils | Mt1 |
| 1.50E-07 | -1.97815 | 0.022 | 0.416 | 0.002706 | Basophils | Ckb |
| 1.51E-07 | -1.30382 | 0 | 0.394 | 0.00272 | Basophils | Nfia |
| 1.58E-07 | -1.37404 | 0 | 0.394 | 0.002852 | Basophils | Col5a1 |
| 1.59E-07 | -1.23617 | 0 | 0.394 | 0.002859 | Basophils | Pkd2 |
| 1.60E-07 | -1.65802 | 0.043 | 0.445 | 0.002887 | Basophils | Prelp |
| 1.72E-07 | -1.61363 | 0.087 | 0.477 | 0.003097 | Basophils | Serpinb6a |
| 1.77E-07 | -1.5779 | 0.152 | 0.529 | 0.003188 | Basophils | Sptbn1 |
| 1.83E-07 | -1.32602 | 0 | 0.391 | 0.003301 | Basophils | Entpd2 |
| 1.84E-07 | -1.34007 | 0.022 | 0.431 | 0.003319 | Basophils | Cpq |
| 1.90E-07 | -1.2903 | 0.022 | 0.425 | 0.003417 | Basophils | Fxyd1 |
| 1.93E-07 | -1.36735 | 0.13 | 0.521 | 0.003471 | Basophils | Pdia6 |
| 2.17E-07 | -4.78081 | 0.652 | 0.771 | 0.003906 | Basophils | Gsn |
| 2.19E-07 | -1.23936 | 0 | 0.387 | 0.003941 | Basophils | Cald1 |
| 2.21E-07 | -1.35353 | 0 | 0.387 | 0.00398 | Basophils | Adamts2 |
| 2.29E-07 | -1.33515 | 0.174 | 0.577 | 0.004118 | Basophils | Ctsl |
| 2.39E-07 | -1.03578 | 0.022 | 0.413 | 0.004306 | Basophils | Tm2d2 |

| | | | | | | |
|---|---|---|---|---|---|---|
| 2.46E-07 | -2.34677 | 0 | 0.385 | 0.004423 | Basophils | Meg3 |
| 2.65E-07 | -1.08872 | 0.022 | 0.425 | 0.004765 | Basophils | Calu |
| 2.76E-07 | -1.35802 | 0 | 0.383 | 0.004972 | Basophils | Fbln5 |
| 3.08E-07 | -1.06733 | 0.043 | 0.44 | 0.005544 | Basophils | Hexa |
| 3.53E-07 | -1.01928 | 0.109 | 0.525 | 0.00636 | Basophils | Park7 |
| 3.70E-07 | 1.904356 | 0.348 | 0.118 | 0.006666 | Basophils | Nfkbid |
| 3.82E-07 | -1.37085 | 0.022 | 0.41 | 0.006881 | Basophils | Nupr1 |
| 3.87E-07 | -1.14054 | 0 | 0.376 | 0.006962 | Basophils | Fermt2 |
| 4.20E-07 | -1.02678 | 0 | 0.375 | 0.007554 | Basophils | Il6st |
| 4.22E-07 | -1.39932 | 0.87 | 0.796 | 0.007603 | Basophils | Rps20 |
| 4.32E-07 | -1.24876 | 0 | 0.374 | 0.007775 | Basophils | Emilin1 |
| 4.56E-07 | 1.478731 | 0.739 | 0.585 | 0.008216 | Basophils | Prdx6 |
| 4.76E-07 | -1.29992 | 0 | 0.372 | 0.008576 | Basophils | Sod3 |
| 4.77E-07 | -1.10037 | 0 | 0.372 | 0.008596 | Basophils | Mfge8 |
| 4.82E-07 | -1.65179 | 0 | 0.372 | 0.008679 | Basophils | Col8a1 |
| 4.90E-07 | -1.30156 | 0 | 0.371 | 0.008825 | Basophils | Ace |
| 4.95E-07 | -1.00064 | 0.109 | 0.533 | 0.008916 | Basophils | Pebp1 |
| 5.01E-07 | -1.20038 | 0.196 | 0.593 | 0.009022 | Basophils | Ppp1r2 |
| 5.06E-07 | -1.14478 | 0.043 | 0.417 | 0.009117 | Basophils | Vkorc1 |
| 5.20E-07 | -1.48047 | 0 | 0.37 | 0.009367 | Basophils | Col6a3 |
| 5.24E-07 | -2.59935 | 0.022 | 0.392 | 0.009437 | Basophils | Thbs1 |
| 5.60E-07 | -1.14004 | 0.022 | 0.393 | 0.01009 | Basophils | Fkbp9 |
| 5.63E-07 | -2.0711 | 0 | 0.369 | 0.010131 | Basophils | C3 |
| 5.65E-07 | -1.34875 | 0 | 0.369 | 0.010179 | Basophils | Fbn1 |
| 5.98E-07 | -1.19718 | 0.022 | 0.397 | 0.010768 | Basophils | Itgb5 |
| 6.05E-07 | -1.09887 | 0.087 | 0.483 | 0.010889 | Basophils | Arl1 |
| 6.56E-07 | -1.0864 | 0.022 | 0.391 | 0.011813 | Basophils | Dpysl2 |
| 6.63E-07 | -2.23724 | 0.022 | 0.389 | 0.011935 | Basophils | Dpep1 |
| 6.88E-07 | -1.23061 | 0 | 0.365 | 0.012386 | Basophils | Medag |
| 6.98E-07 | -1.4828 | 0.065 | 0.442 | 0.012558 | Basophils | Tnxb |
| 7.13E-07 | -1.51046 | 0 | 0.364 | 0.012831 | Basophils | Hsd11b1 |
| 7.84E-07 | -1.3185 | 0.065 | 0.452 | 0.014114 | Basophils | Emp1 |
| 8.01E-07 | -1.49566 | 0 | 0.361 | 0.014429 | Basophils | Pcolce2 |
| 8.15E-07 | -1.15494 | 0 | 0.361 | 0.014667 | Basophils | Lama4 |
| 8.34E-07 | 1.253498 | 0.283 | 0.093 | 0.015017 | Basophils | Cebpa |
| 8.97E-07 | -1.01983 | 0 | 0.359 | 0.016142 | Basophils | Cnn3 |
| 9.08E-07 | -1.50626 | 0.022 | 0.382 | 0.016355 | Basophils | Adamts5 |
| 9.13E-07 | -1.48129 | 0.065 | 0.433 | 0.016437 | Basophils | Pnp |
| 9.17E-07 | -1.74221 | 0.478 | 0.776 | 0.016508 | Basophils | Fos |
| 9.64E-07 | -1.06759 | 0 | 0.358 | 0.01735 | Basophils | Cavin1 |
| 1.01E-06 | -2.24147 | 0.283 | 0.583 | 0.01815 | Basophils | Pi16 |
| 1.01E-06 | -1.13731 | 0.022 | 0.393 | 0.018228 | Basophils | Pdgfra |
| 1.05E-06 | -1.23164 | 0.022 | 0.383 | 0.018977 | Basophils | Col5a2 |
| 1.10E-06 | -1.03088 | 0.065 | 0.441 | 0.01972 | Basophils | Ubl3 |
| 1.12E-06 | -1.02054 | 0.739 | 0.78 | 0.020176 | Basophils | Rpl3 |
| 1.17E-06 | -1.48029 | 0.283 | 0.621 | 0.021078 | Basophils | Rhob |
| 1.41E-06 | -1.12219 | 0 | 0.35 | 0.025397 | Basophils | Aebp1 |
| 1.45E-06 | -1.1783 | 0 | 0.349 | 0.026165 | Basophils | Angptl2 |
| 1.49E-06 | -1.41749 | 0.022 | 0.383 | 0.026787 | Basophils | Atf5 |
| 1.57E-06 | -1.02142 | 0 | 0.348 | 0.02828 | Basophils | Mmp23 |
| 1.61E-06 | -1.48616 | 0.022 | 0.372 | 0.028927 | Basophils | Gem |
| 1.69E-06 | -1.11912 | 0.326 | 0.636 | 0.030387 | Basophils | Lamp1 |
| 1.93E-06 | 1.023491 | 0.761 | 0.666 | 0.034719 | Basophils | Gpi1 |
| 1.95E-06 | -1.21061 | 0 | 0.343 | 0.035179 | Basophils | Tm4sf1 |

| | | | | | | |
|---|---|---|---|---|---|---|
| 2.04E-06 | -1.04342 | 0 | 0.342 | 0.036724 | Basophils | Dpysl3 |
| 2.14E-06 | -1.17059 | 0.065 | 0.426 | 0.038527 | Basophils | Nfic |
| 2.34E-06 | -1.03752 | 0 | 0.339 | 0.042043 | Basophils | Vwa1 |
| 2.36E-06 | -1.0369 | 0.152 | 0.51 | 0.042501 | Basophils | Rpn2 |
| 2.60E-06 | -1.09165 | 0.022 | 0.367 | 0.046842 | Basophils | Nav1 |
| 2.65E-06 | -1.21161 | 0 | 0.336 | 0.047777 | Basophils | Htra1 |
| 2.67E-06 | -1.5814 | 0.022 | 0.364 | 0.048106 | Basophils | Timp3 |
