# Supplementary figures and images for: Integrated Single-Cell and RNA Sequencing Analysis Identifies Key Immune Cell and Dendritic Cells Associated Genes Participated in Myocarditis
Source: J Immunol Res. 2022 Oct 3;2022:8655343. doi: 10.1155/2022/8655343 (PMC9550476; doi:10.1155/2022/8655343)

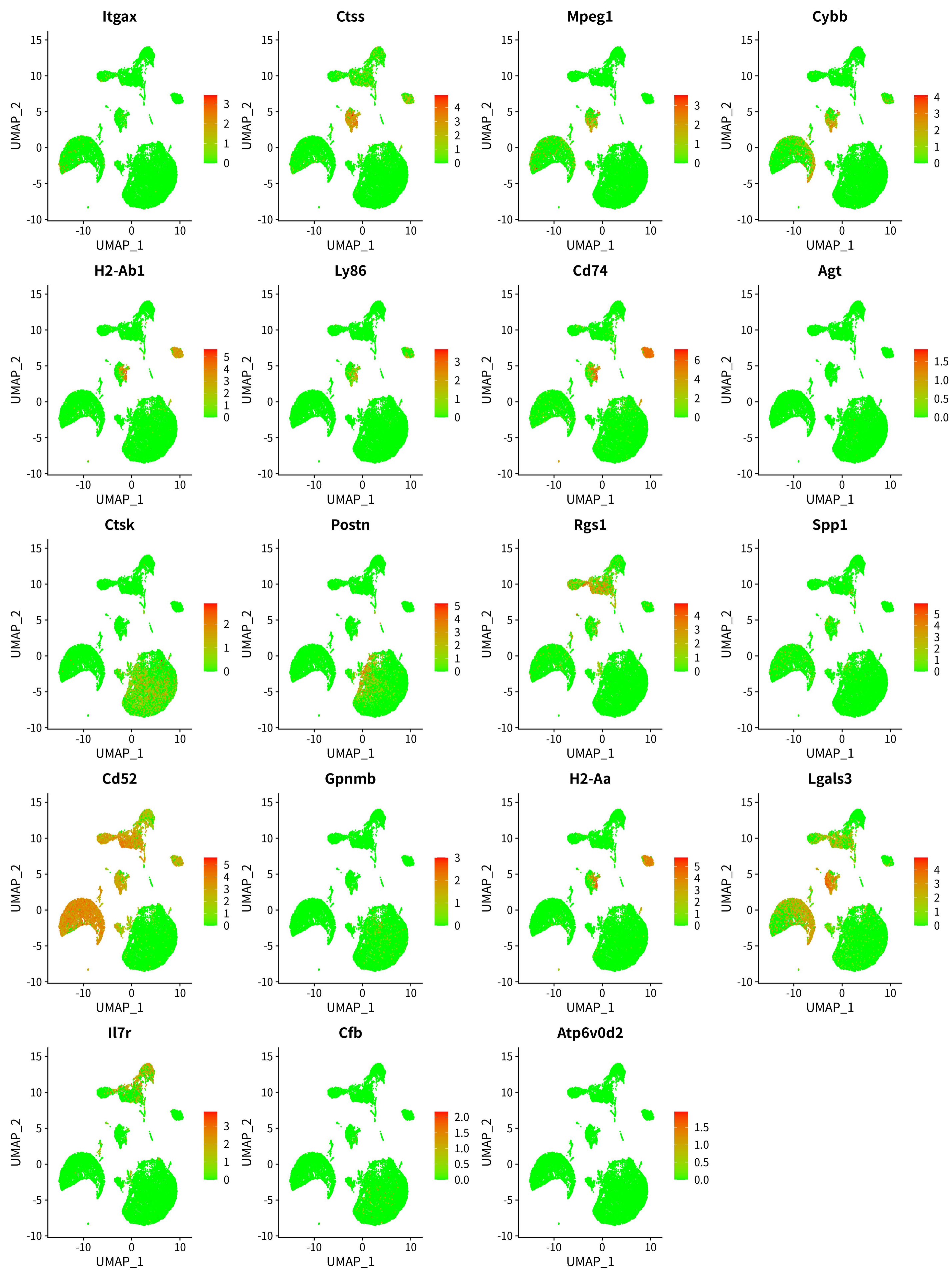

Supplement: Supplementary Materials — See Figures S1-S2 and Table S1in the file of Supplementary Material. [file 8655343.f1.zip › Supplemental Figure 1.pdf]

**Control**

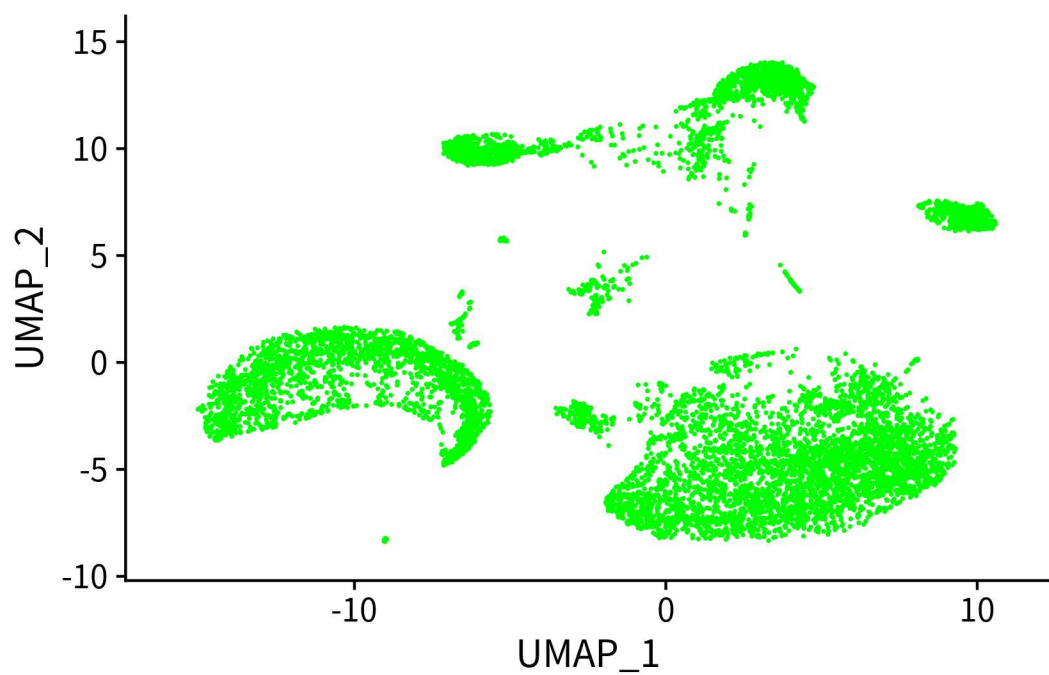

**Myocarditis**

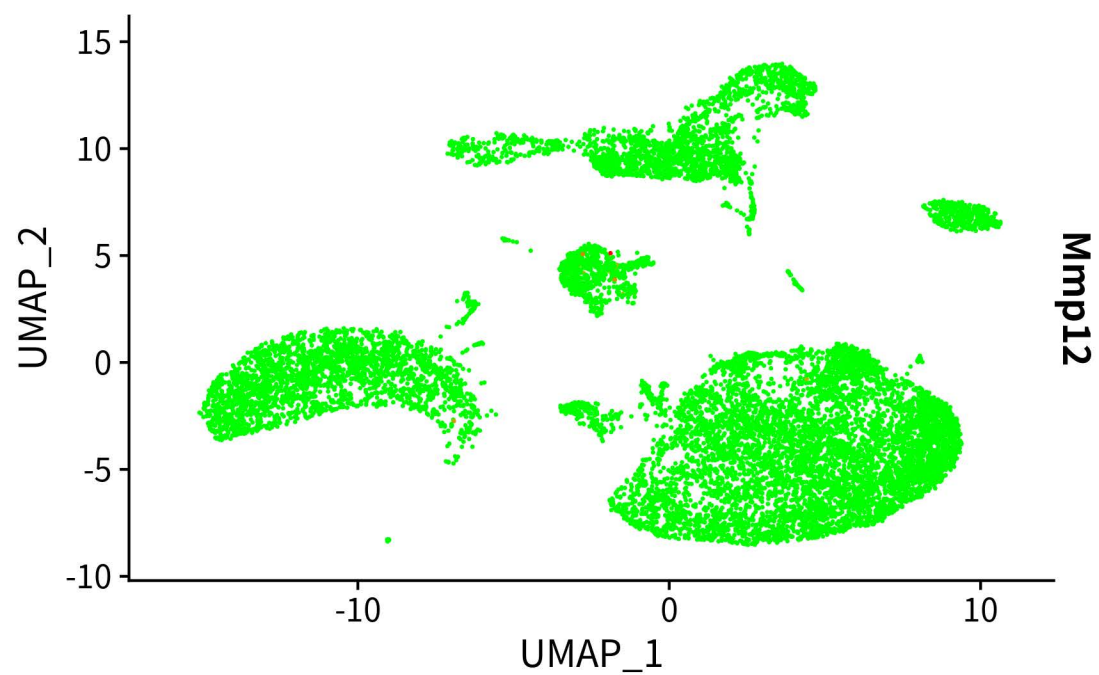

**Mmp12**

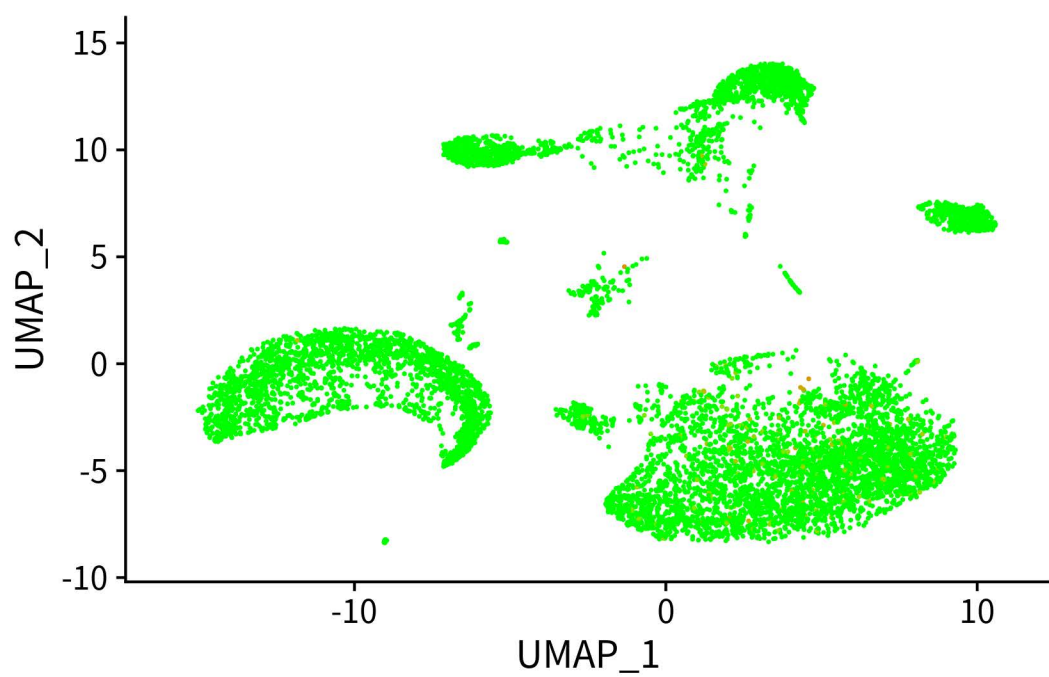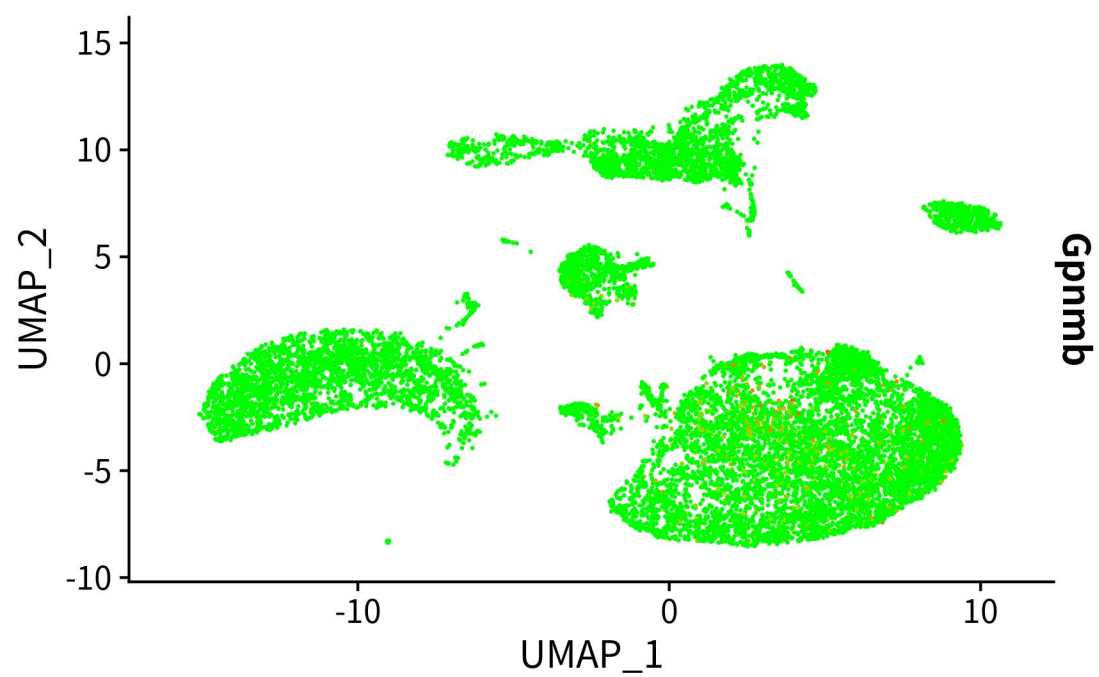

**Gpmmb**

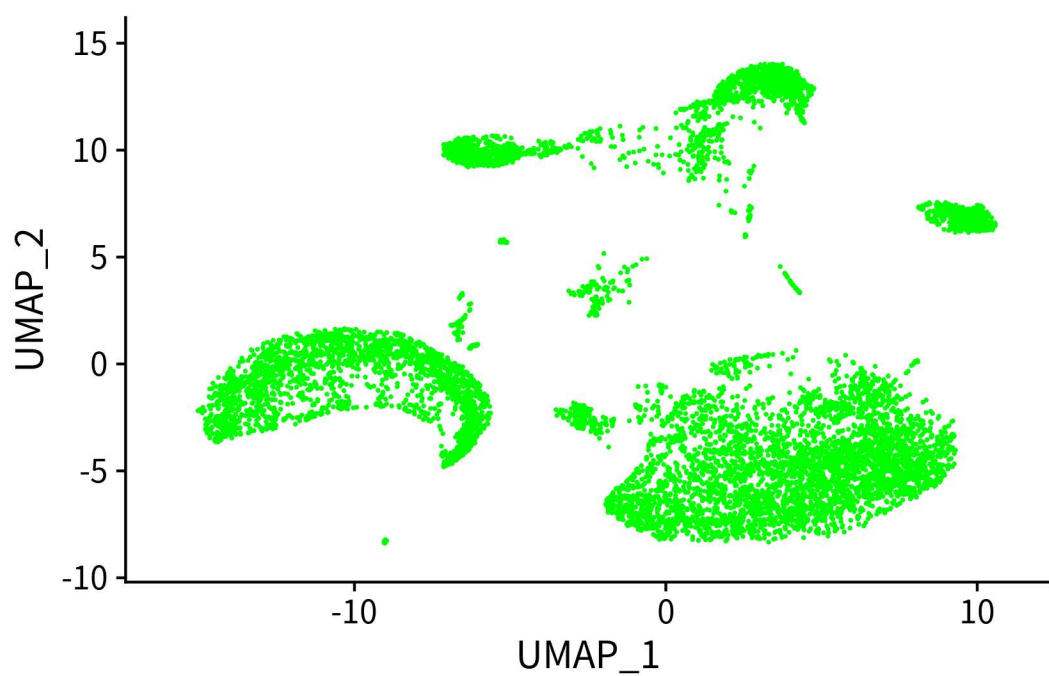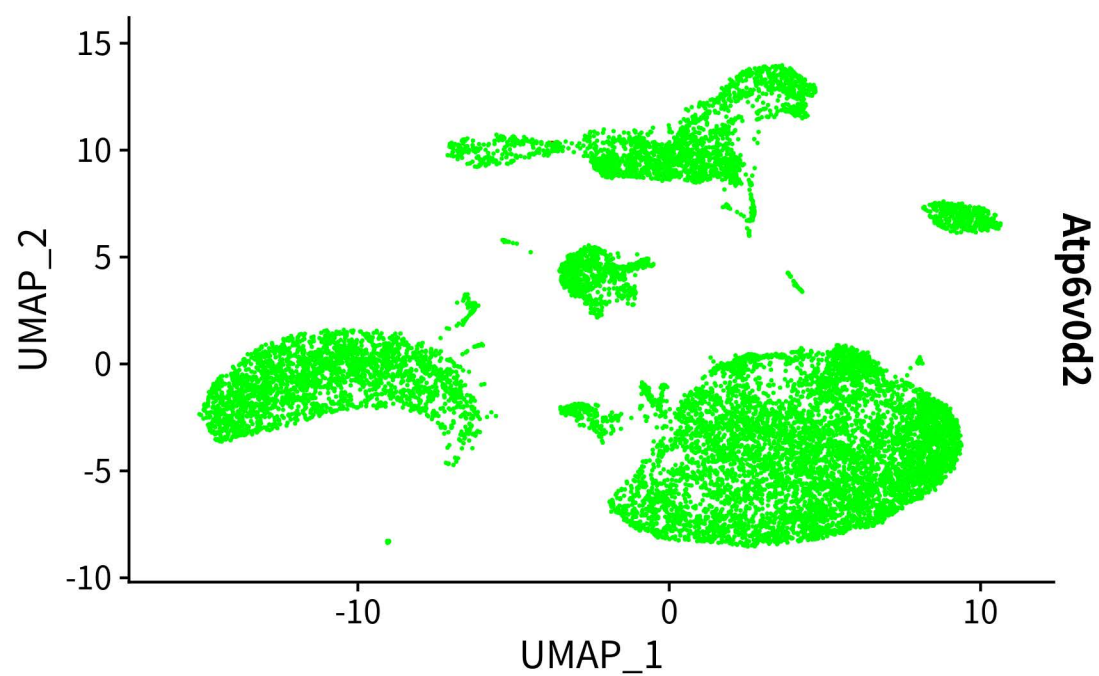

**Atp6v0d2**

Supplement: Supplementary Materials — See Figures S1-S2 and Table S1in the file of Supplementary Material. [file 8655343.f1.zip › Supplemental Figure 2.pdf]
